# ﻿Revision of the Malagasy Camponotus subgenus Myrmosaga (Hymenoptera, Formicidae) using qualitative and quantitative morphology

**DOI:** 10.3897/zookeys.1098.73223

**Published:** 2022-05-03

**Authors:** Jean Claude Rakotonirina, Brian L. Fisher

**Affiliations:** 1 Madagascar Biodiversity Center, BP 6257, Parc Botanique et Zoologique de Tsimbazaza, Antananarivo, Madagascar Madagascar Biodiversity Center Antananarivo Madagascar; 2 Département d’Entomologie-Culture, Elevage, Santé; Faculté des Sciences, BP 906, Université d’Antananarivo, Antananarivo, Madagascar Université d’Antananarivo Antananarivo Madagascar; 3 Entomology, California Academy of Sciences, 55 Music Concourse Drive, San Francisco, CA 94118, USA Entomology, California Academy of Sciences San Francisco United States of America

**Keywords:** Madagascar, morphometry, species delimitation, subgenus *Myrmosaga*, taxonomy

## Abstract

The CamponotussubgenusMyrmosaga**subgen. rev.** from the Malagasy region is revised based on analysis of both qualitative morphological characters and morphometric traits. The multivariate analysis used the Nest Centroid (NC)-clustering method to generate species hypotheses based on 19 continuous morphological traits of minor workers. The proposed species hypotheses were confirmed by cumulative Linear Discriminant Analysis (LDA). Morphometric ratios for the subsets of minor and major workers were used in species descriptions and redefinitions. The present study places the subgenus Myrmopytia**syn. nov.** in synonymy to *Myrmosaga*. It recognizes 38 species, of which 19 are newly described: *C.aina***sp. nov.**, *C.aro***sp. nov.**, *C.asara***sp. nov.**, *C.atimo***sp. nov.**, *C.bemaheva***sp. nov.**, *C.bozaka***sp. nov.**, *C.daraina***sp. nov.**, *C.harenarum***sp. nov.**, *C.joany***sp. nov.**, *C.karsti***sp. nov.**, *C.kelimaso***sp. nov.**, *C.lokobe***sp. nov.**, *C.mahafaly***sp. nov.**, *C.niavo***sp. nov.**, *C.rotrae***sp. nov.**, *C.sambiranoensis***sp. nov.**, *C.tapia***sp. nov.**, *C.tendryi***sp. nov.**, *C.vano***sp. nov.** Eleven species are redescribed: *C.aurosus* Roger, *C.cervicalis* Roger, *C.dufouri* Forel, *C.gibber* Forel, *C.hagensii* Forel, *C.hova* Forel, *C.hovahovoides* Forel, *C.immaculatus* Forel, *C.quadrimaculatus* Forel, *C.roeseli* Forel, *C.strangulatus* Santschi. The following are raised to species and redescribed: *C.becki* Santschi **stat. nov.**, *C.boivini* Forel **stat. rev.**, *C.cemeryi* Özdikmen **stat. rev.**, *C.mixtellus* Forel **stat. nov.**, *C.radamae* Forel **stat. nov.**Camponotusmaculatusst.fairmairei Santschi **syn. nov.**, is synonymized under *C.boivini*. The following are synonymized under *C.cervicalis*: *Camponotuscervicalisgaullei* Santschi, **syn. nov.**; *Camponotusperroti* Forel, **syn. nov.**; *Camponotusperrotiaeschylus* Forel, **syn. nov.**; *Camponotusgerberti* Donisthorpe, **syn. nov.***Camponotusdufouriimerinensis* Forel, **syn. nov.** is a synonym of *C.dufouri*, Camponotushovavar.obscuratus Emery, **syn. nov.** is a synonym of *C.hova*, *Camponotusquadrimaculatusopacata* Emery, **syn. nov.** is a synonym of *C.immaculatus*, Camponotusmaculatusst.legionarium Santschi, **syn. nov.** is a synonym of *C.roeseli*, *Camponotushovamaculatoides* Emery, **syn. nov.** is a synonym of *C.strangulatus*. The following are synonymized under *C.quadrimaculatus*: *Camponotuskelleri* Forel, **syn. nov.**, Camponotuskellerivar.invalidus Forel, **syn. nov.**, *Camponotusquadrimaculatussellaris* Emery, **syn. nov.** As *C.imitator* Forel, *C.liandia* Rakotonirina & Fisher, and *C.lubbocki* Forel have been recently described and redescribed, only diagnoses and taxonomic discussions are provided. This revision also includes an illustrated species identification key, taxonomic discussions, images, and distribution maps for each species superimposed on the ecoregions of Madagascar.

## ﻿Introduction

Given the striking morphological variation of Malagasy *Camponotus*, taxonomic knowledge of this genus has been advanced recently using a combination of traditional morphology-based study and a morphometry-based approach. These techniques and the use of other sources of evidence have helped assign individual specimens to a species, facilitate species recognition, and improve precision of species delimitation in recent revisions (Rakotonirina et al. 2016, 2017; Rasoamanana et al. 2017; Rakotonirina and Fisher 2018). We continue to apply these techniques to revise the subgenus Myrmosaga (Forel 1912) as part of an ongoing project to revise the entire genus in the Malagasy region.

*Myrmosaga* was created by Forel (1912) as a subgenus of *Camponotus*, but later it was synonymized under *Mayria* by Emery (1925) based on a few morphological characters that are shared by both subgenera. However, our observation of the samples collected from the recent extensive inventory of ant fauna in the Malagasy region revealed two separate groups. One group includes the species of *Myrmosaga* and another contains species that remain in the group *Mayria*. In addition, *Camponotusimitator*, the type species of the subgenus Myrmopytia (Emery, 1920a) is grouped with the species of *Myrmosaga*. This grouping is also supported by the molecular analysis using UCE (Ultra-Conserved Element) phylogenomic data (Unpublished data). Species of *Myrmosaga* are remarkably different from those of *Mayria* and therefore the subgenus needs to be revived now from synonymy. *Myrmopytia* is also synonymized under *Myrmosaga* in the present study. The other three species (*C.jodina*, *C.karaha*, and *C.longicolis*) recently described under *Myrmopytia* will be moved to a different subgenus (Rasoamanana in prep.).

Members of *Myrmosaga* are found only in Madagascar and surrounding islands of the southwest Indian Ocean region. They occupy all the terrestrial ecoregions encountered in this region. Morphologically, the worker castes of the subgenus present a wide range of features that are the result of its substantial adaptive radiation across these islands, but particularly in Madagascar. Several species within the subgenus show development of a few or many characters similar to fauna from the Afrotropical region. Our understanding of this observed morphological diversification has triggered taxonomic changes for some of the species that were described and combined previously under different subgenera of *Camponotus* (Table 1).

**Table 1.** Summary of the new subgenus placements for the Malagasy Camponotus (Myrmosaga).

Taxon	Changes	Old subgenus placement	New subgenus placement
*Myrmopytia*	syn. nov.		*Myrmosaga*
*Camponotusaurosus*	subgenus	*Myrmosericus*	*Myrmosaga*
*Camponotusbecki*	subgenus	*Tanaemyrmex*	*Myrmosaga*
*Camponotusboivini*	subgenus	*Tanaemyrmex*	*Myrmosaga*
*Camponotuscemeryi*	subgenus	*Tanaemyrmex*	*Myrmosaga*
*Camponotuscervicalis*	subgenus	*Tanaemyrmex*	*Myrmosaga*
*Camponotusdufouri*	subgenus	*Tanaemyrmex*	*Myrmosaga*
*Camponotusgibber*	subgenus	*Mayria*	*Myrmosaga*
*Camponotusgouldi*	subgenus	*Tanaemyrmex*	*Myrmosaga*
*Camponotushagensii*	subgenus	*Tanaemyrmex*	*Myrmosaga*
*Camponotushova*	subgenus	*Tanaemyrmex*	*Myrmosaga*
*Camponotushovahovoides*	subgenus	*Tanaemyrmex*	*Myrmosaga*
*Camponotusimitator*	subgenus	*Myrmopytia*	*Myrmosaga*
*Camponotusimmaculatus*	subgenus	*Mayria*	*Myrmosaga*
*Camponotusliandia*	subgenus	*Mayria*	*Myrmosaga*
*Camponotuslubbocki*	subgenus	*Mayria*	*Myrmosaga*
*Camponotusmixtellus*	subgenus	*Tanaemyrmex*	*Myrmosaga*
*Camponotusquadrimaculatus*	subgenus	*Mayria*	*Myrmosaga*
*Camponotusradamae*	subgenus	*Tanaemyrmex*	*Myrmosaga*
*Camponotusroeseli*	subgenus	*Tanaemyrmex*	*Myrmosaga*
*Camponotusstrangulatus*	subgenus	*Tanaemyrmex*	*Myrmosaga*

This proposed *Myrmosaga* classification is based on the use of additional morphological features thought to be relevant in defining the subgenus more precisely. Minor workers of *Myrmosaga* are generally characterized by the combination of the following morphological characters: clypeus medially carinate, mandible armed with six teeth, laterodorsal angle of mesosoma never marginate, petiolar node never conical.

The present revision recognizes 38 species in the subgenus Myrmosaga, provides an illustrated species-level identification key, and a description of each species complemented by high-resolution montage images and a geographic distribution map.

## ﻿Materials and methods

### ﻿Abbreviation of depositories

**CAS**California Academy of Sciences, San Francisco, CA, USA.

**MHNG**Musée d’Histoire Naturelle, Geneva, Switzerland.

**MNHN**Musée National d’Histoire Naturelle, Paris, France.

**MSNG**Museo Civico di Storia Naturale “Giacomo Doria”, Genoa, Italy.

**NHMB**Naturhistorisches Museum, Basel, Switzerland.

**NHMUK**NNatural History Museum, London, UK.

**PBZT**Parc Botanique et Zoologique de Tsimbazaza, Antananarivo, Madagascar.

**PSWC** P.S. Ward Collection, University of California at Davis, CA, USA.

**ZMHB** Museum für Naturkunde der Humboldt Universität, Berlin, Germany.

### ﻿Materials

The present study includes all specimens of the subgenus Myrmosaga collected from the arthropod survey project conducted in Madagascar and surrounding islands in the Malagasy region by the members of the Madagascar Biodiversity Center and other ant researchers. Most of these collections were sampled from 1992 through 2018. All pinned specimens examined in this study are available on the AntWeb portal (http://www.antweb.org) and can be accessed using the unique identifying specimen code (CASENTnumber) assigned to each pinned specimen.

A total of 776 specimens from 324 collecting events was measured in this study (see Suppl. material 1). Collection event codes with prefix AND, ANTC, ARA, BLF, MG, or PSW indicate distinct collecting events, and were used as grouping factors in the NC-clustering method. One grouping factor may contain up to three individual specimens for measurements.

### ﻿Methods

Using a Leica MZ12 binocular microscope, qualitative morphological examinations were conducted on minor and major workers to evaluate patterns of morphological discontinuities and phenotypic similarity. Observed discontinuities in morphological space could indicate reproductive boundaries between populations and as such were used to infer potential species limits. Species limits based on qualitative morphological characters were compared with the species hypothesis derived from the quantitative analysis of measurements. Differences between the sizes of the measured worker castes in the present study might result in a conflict between the two approaches. To avoid this conflict any specimen with unusual size class was removed from the analysis.

Digital color images of lateral and dorsal views of the entire body and full-face views of the head of each species were created using a JVC KY-75 or a Leica DFC450 digital camera with a Leica Z16 APO microscope and Leica Application Suite software (v3.8). These images are also available online on AntWeb (www.antweb.org) and are accessible using the unique identifying specimen code.

Distribution maps for all species were generated by importing specimen distribution records into the Diva GIS program (Hijmans et al. 2011). The major ecoregions of Madagascar were superimposed on the distribution of each species. Specimens with inadequate geographic coordinates were excluded from these maps. Information about the biology of each species was based on data obtained from specimen sampling (the nest series and isolated worker specimens) in the field.

Article 74 in the ICZN’s code states that a lectotype may be designated from syntype specimens which directly match the original description of a named species in order to stabilize the nomenclature. Therefore, the phrase “by present designation” is used to indicate a lectotype. As stated in Article 75.1 of the ICZN’s code, neotype designation is necessary for a name with no extant name-bearing type to objectively define the nominal taxon. Thus, a neotype has been selected according to the qualifying conditions specified in Article 75.3.

Etymologies are provided for new species to facilitate name stability. Etymologies indicate if the new name is a non-Latin or Latin (or Latinized) word. For Latin names, we also include the part of speech (nouns, adjectives, participles), gender (masculine, feminine, or neuter), number (singular or plural) and grammatical case (e.g. subjective/nominative, possessive/genitive).

### ﻿Measurements

Morphometric measurements of the minor and major workers were taken using a Leica MZ12 stereomicroscope equipped with a cross-scaled ocular micrometer and an orthogonal pair of micrometers. All measurements and indices are presented as arithmetic means and ranges are shown as minimum and maximum values in parentheses. Body size dimensions are expressed in millimeters (mm) and all values were rounded to the second decimal place.

The following 19 morphometric measurements were evaluated in the present revision:

**CL** (Maximum cephalic length): The maximum midline length of the head in full-face view, measured from the midpoint of the posterior margin to the midpoint of the anterior margin of the clypeus.

**ClyL** (Clypeal length): the maximum midline length of the clypeus measured from the posterior margin to the anterior margin in anterodorsal view, in which the anterior and posterior clypeal margins are aligned to the same focus. Median concavity on either or both margins reduces the length of the clypeus.

**CS** (Cephalic size): The arithmetic mean of CL and CWb. CS is used to indicate the general body size of the ant.

**CW** (Maximum cephalic width): The maximum distance between the lateral margins of the compound eyes in full-face view.

**CWb** (Maximum head capsule width): The maximum width of the head excluding the compound eyes.

**EL** (Eye length): Maximum diameter of the compound eye.

**FR** (Frontal carina distance): The maximum distance between the frontal carinae.

**GPD** (Maximum tentorial pit distance): The longest distance between the centers of the fossae located at or very close to the posterolateral margin of the clypeus.

**HTL** (Maximum hind tibia length): Straight line length of the hind tibia measured from the constriction immediately before its proximal insertion to its distalmost point, excluding the bristles or spines. The measurement of the tibia is taken from any view where the constriction before its proximal insertion is visible.

**ML** (Mesosoma length): The longest median anatomical line that connects the posteriormost point of the propodeal lobe with the anteriormost point of the pronotal collar; preferentially measured in lateral view, but if one of the reference points is not visible, dorsal view may be used.

**MPD** (Mesothoracico-propodeal distance): With the promesonotal suture and the anterior petiolar foramen margin in the same plane of focus in dorsal view, the maximum midline length between the promesonotal suture and the posteriormost point of the propodeal process dorsal to the petiolar insertion (see Rakotonirina et al. 2016: fig. 1).

**MPH** (Mesothoracico-propodeal height): With the mesosoma in lateral view, the length of the line between the anteroventral corner of the mesopleuron dorsal to the insertion of the mesocoxa, and the dorsalmost point of the propodeum that is crossed by the measured line. The line is perpendicular to the diagonal line of the mesosoma that connects the anteriormost point of the pronotal shield and the posteriormost point of the propodeal process dorsal to the petiolar insertion, in lateral view.

**MW** (Mesosoma width): Maximum width of the pronotum in dorsal view, which in the subgenus Myrmosaga is also the maximum mesosomal width (hence “mesosoma width”).

**NOH** (Petiolar node height): The maximum distance between the petiolar spiracle and the dorsalmost point of the petiolar node.

**OMD** (Oculo-mandibular distance): The smallest distance between the anterior margin of the compound eye and the mandibular insertion to the head.

**PEW** (Petiolar width): The maximum width of the petiole in dorsal view.

**PoOc** (Postocular distance): The distance between the posteromedian margin of the head and the level of the posterior margin of the compound eyes measured along the midline of the head in full-face view.

**PrOc** (Preocular distance): The distance between the anteromedian margin of the clypeus and the level of the anterior margin of the compound eyes measured along the midline of the head in full-face view.

**SL** (Scape length): Straight line length of the first antennal segment excluding the basal condyle.

**TCD** (Torular carina distance): The minimum distance between the torular arches that surround the antennal insertion.

### ﻿Multivariate statistical analyses

The datasets assessed in the present study consist of (1) the primary measurement data of the 19 morphological characters and the one calculated character CS (a widely applied size indicator) of each measured specimen (see table of basic measurements of the specimens in the Suppl. material 1), and (2) the ratios (indices) of measurements involving the comparison of one measured trait (variable) over another (CS, CL, or ML) to show the body proportions or shape of the specimen (Table 2). In Table 2, the ratios of measurements for minor and major workers of each species are presented as arithmetic means with standard deviation in the upper line and as ranges in square brackets in the lower line if more than two individual specimens are measured.

**Table 2.** Ratios of morphometric data for minors and majors of the species. Upper line: mean of ratios ± standard deviation, lower line in square brackets: minimum and maximum values. Note: if two or more specimens were available, then minimum, maximum values are given; one value is presented if only one specimen was available.

Species	Castes	CS	CWb/CL	CW/CL	PrOc/CL	PoOc/CL	FR/CS	TCD/CS	ClyL/CL	ClyL/GPD	SL/CS	EL/CS	OMD/CS	MW/ML	PEW/CS	MPD/ML	HTL/CS	ML/CS	MPH/ML	NOH/CS
*aina*	Minor (n= 2)	4.41±0.74	0.72±0.00	0.69±0.01	0.49±0.01	0.26±0.00	0.28±0.01	0.21±0.01	0.29±0.01	0.74±0.07	1.64±0.02	0.24±0.02	0.47±0.03	0.38±0.01	0.21±0.04	0.66±0.05	1.94±0.04	1.91±0.03	0.31±0.09	0.17±0.01
		[3.89, 4.94]	[0.72, 0.72]	[0.68, 0.70]	[0.49, 0.50]	[0.26, 0.26]	[0.27, 0.29]	[0.20, 0.21]	[0.28, 0.29]	[0.69, 0.80]	[1.62, 1.66]	[0.22, 0.26]	[0.46, 0.49]	[0.37, 0.39]	[0.19, 0.24]	[0.63, 0.69]	[1.91, 1.97]	[1.89, 1.93]	[0.25, 0.37]	[0.16, 0.18]
*aro*	Minor (n= 31)	1.78±0.15	0.72±0.05	0.67±0.04	0.50±0.01	0.28±0.01	0.25±0.01	0.23±0.01	0.28±0.01	0.72±0.04	1.59±0.08	0.25±0.01	0.53±0.02	0.33±0.01	0.22±0.01	0.68±0.01	1.96±0.09	1.86±0.06	0.28±0.01	0.23±0.01
		[1.56, 2.09]	[0.62, 0.79]	[0.57, 0.70]	[0.48, 0.53]	[0.27, 0.30]	[0.24, 0.27]	[0.22, 0.25]	[0.24, 0.29]	[0.63, 0.77]	[1.46, 1.71]	[0.23, 0.26]	[0.52, 0.58]	[0.32, 0.35]	[0.20, 0.24]	[0.66, 0.71]	[1.76, 2.08]	[1.73, 1.94]	[0.26, 0.30]	[0.21, 0.24]
	Major (n= 5)	3.10±0.18	0.92±0.01	0.75±0.01	0.55±0.01	0.27±0.01	0.25±0.00	0.22±0.00	0.30±0.00	0.82±0.01	0.99±0.06	0.20±0.01	0.51±0.01	0.59±0.01	0.19±0.01	0.49±0.01	1.33±0.06	0.93±0.04	1.47±0.02	0.18±0.00
		[2.82, 3.29]	[0.90, 0.94]	[0.74, 0.77]	[0.54, 0.56]	[0.25, 0.28]	[0.24, 0.25]	[0.22, 0.23]	[0.29, 0.30]	[0.81, 0.83]	[0.93, 1.07]	[0.19, 0.21]	[0.50, 0.52]	[0.57, 0.60]	[0.19, 0.20]	[0.48, 0.50]	[1.26, 1.41]	[0.91, 1.00]	[1.44, 1.49]	[0.18, 0.19]
*asara*	Minor (n= 19)	1.67±0.13	0.72±0.07	0.67±0.01	0.51±0.02	0.26±0.01	0.26±0.01	0.22±0.01	0.29±0.01	0.72±0.02	1.57±0.08	0.26±0.01	0.52±0.02	0.34±0.01	0.25±0.01	0.71±0.01	1.89±0.09	1.86±0.07	0.34±0.01	0.26±0.02
		[1.49, 2.02]	[0.67, 0.97]	[0.65, 0.70]	[0.49, 0.54]	[0.24, 0.28]	[0.23, 0.27]	[0.19, 0.23]	[0.28, 0.30]	[0.68, 0.76]	[1.38, 1.68]	[0.23, 0.28]	[0.44, 0.55]	[0.33, 0.36]	[0.22, 0.26]	[0.69, 0.73]	[1.67, 1.99]	[1.68, 1.95]	[0.33, 0.38]	[0.23, 0.29]
	Major (n= 4)	3.22±0.15	0.93±0.01	0.77±0.01	0.54±0.01	0.27±0.01	0.24±0.03	0.21±0.02	0.30±0.01	0.86±0.03	0.90±0.11	0.20±0.01	0.49±0.01	0.58±0.01	0.22±0.02	0.53±0.02	1.23±0.05	0.98±0.03	1.38±0.03	0.19±0.01
		[3.03, 3.39]	[0.92, 0.93]	[0.75, 0.78]	[0.53, 0.55]	[0.27, 0.28]	[0.19, 0.26]	[0.18, 0.22]	[0.30, 0.31]	[0.83, 0.89]	[0.74, 0.99]	[0.19, 0.20]	[0.48, 0.49]	[0.56, 0.59]	[0.21, 0.25]	[0.51, 0.55]	[1.18, 1.29]	[0.95, 1.02]	[1.33, 1.41]	[0.18, 0.21]
*atimo*	Minor (n= 31)	1.56±0.12	0.66±0.03	0.66±0.02	0.50±0.01	0.27±0.01	0.23±0.01	0.21±0.01	0.28±0.01	0.74±0.03	1.66±0.09	0.28±0.01	0.51±0.01	0.34±0.02	0.22±0.01	0.69±0.01	1.89±0.09	1.97±0.06	0.33±0.02	0.24±0.02
		[1.28, 1.79]	[0.59, 0.72]	[0.62, 0.68]	[0.47, 0.52]	[0.25, 0.29]	[0.22, 0.25]	[0.20, 0.23]	[0.27, 0.29]	[0.67, 0.81]	[1.48, 1.84]	[0.26, 0.30]	[0.48, 0.53]	[0.30, 0.37]	[0.21, 0.25]	[0.65, 0.71]	[1.72, 2.08]	[1.85, 2.11]	[0.29, 0.36]	[0.20, 0.27]
	Major (n= 8)	3.23±0.34	0.97±0.04	0.77±0.02	0.53±0.01	0.29±0.01	0.24±0.01	0.21±0.00	0.29±0.01	0.86±0.03	0.89±0.06	0.19±0.01	0.47±0.01	0.59±0.02	0.19±0.01	0.55±0.03	1.09±0.05	0.93±0.05	1.41±0.05	0.19±0.01
		[2.80, 3.77]	[0.89, 1.03]	[0.73, 0.79]	[0.52, 0.55]	[0.27, 0.30]	[0.23, 0.24]	[0.20, 0.21]	[0.28, 0.30]	[0.82, 0.89]	[0.80, 1.00]	[0.17, 0.20]	[0.46, 0.48]	[0.56, 0.63]	[0.18, 0.21]	[0.52, 0.62]	[1.02, 1.20]	[0.84, 1.00]	[1.36, 1.51]	[0.17, 0.21]
*aurosus*	Minor (n= 16)	1.37±0.05	0.74±0.02	0.74±0.01	0.54±0.01	0.24±0.01	0.30±0.01	0.25±0.01	0.33±0.01	0.79±0.02	1.33±0.04	0.27±0.01	0.53±0.01	0.39±0.02	0.25±0.02	0.73±0.04	1.48±0.05	1.74±0.08	0.36±0.03	0.25±0.03
		[1.27, 1.45]	[0.71, 0.77]	[0.72, 0.76]	[0.52, 0.56]	[0.22, 0.26]	[0.29, 0.32]	[0.24, 0.26]	[0.32, 0.34]	[0.76, 0.85]	[1.27, 1.40]	[0.25, 0.29]	[0.51, 0.54]	[0.34, 0.41]	[0.22, 0.29]	[0.61, 0.76]	[1.39, 1.54]	[1.67, 2.04]	[0.30, 0.41]	[0.22, 0.30]
	Major (n= 4)	2.24±0.11	0.92±0.03	0.81±0.02	0.57±0.01	0.24±0.01	0.29±0.01	0.26±0.00	0.35±0.01	0.92±0.02	0.93±0.06	0.21±0.02	0.49±0.01	0.60±0.02	0.21±0.01	0.54±0.02	1.02±0.08	0.95±0.05	1.38±0.02	0.23±0.08
		[2.15, 2.37]	[0.87, 0.95]	[0.79, 0.82]	[0.56, 0.59]	[0.22, 0.25]	[0.28, 0.30]	[0.25, 0.26]	[0.34, 0.36]	[0.90, 0.94]	[0.86, 0.99]	[0.19, 0.23]	[0.47, 0.50]	[0.59, 0.64]	[0.20, 0.21]	[0.50, 0.56]	[0.94, 1.12]	[0.90, 1.00]	[1.37, 1.41]	[0.17, 0.35]
*becki*	Minor (n= 11)	1.63±0.18	0.77±0.03	0.75±0.01	0.50±0.01	0.26±0.01	0.27±0.01	0.24±0.01	0.30±0.01	0.72±0.03	1.27±0.05	0.27±0.02	0.49±0.01	0.38±0.02	0.22±0.01	0.72±0.03	1.47±0.05	1.72±0.09	0.37±0.02	0.24±0.06
		[1.34, 1.96]	[0.73, 0.83]	[0.73, 0.77]	[0.48, 0.51]	[0.24, 0.27]	[0.26, 0.29]	[0.22, 0.25]	[0.29, 0.31]	[0.67, 0.77]	[1.15, 1.34]	[0.25, 0.30]	[0.48, 0.50]	[0.35, 0.43]	[0.21, 0.24]	[0.70, 0.78]	[1.38, 1.56]	[1.58, 1.91]	[0.33, 0.42]	[0.2, 0.28]
	Major (n= 3)	2.80±0.23	0.97±0.03	0.83±0.02	0.53±0.01	0.27±0.01	0.26±0.00	0.22±0.00	0.32±0.01	0.85±0.01	0.84±0.03	0.21±0.01	0.46±0.01	0.87±0.43	0.19±0.01	0.55±0.01	1.03±0.03	0.95±0.02	1.39±0.02	0.20±0.02
		[2.54, 2.96]	[0.94, 1.00]	[0.80, 0.84]	[0.51, 0.54]	[0.26, 0.27]	[0.26, 0.26]	[0.22, 0.22]	[0.31, 0.33]	[0.84, 0.87]	[0.81, 0.87]	[0.20, 0.22]	[0.46, 0.47]	[0.62, 1.37]	[0.18, 0.19]	[0.54, 0.56]	[1.00, 1.06]	[0.93, 0.96]	[1.38, 1.41]	[0.18, 0.22]
*bemaheva*	Minor (n= 23)	1.63±0.13	0.64±0.03	0.64±0.01	0.49±0.01	0.26±0.01	0.26±0.01	0.22±0.01	0.29±0.01	0.70±0.02	1.63±0.10	0.30±0.01	0.50±0.02	0.37±0.01	0.28±0.02	0.75±0.01	1.80±0.08	2.02±0.07	0.40±0.02	0.28±0.02
		[1.28, 1.79]	[0.53, 0.68]	[0.62, 0.67]	[0.47, 0.50]	[0.24, 0.28]	[0.25, 0.29]	[0.20, 0.24]	[0.27, 0.29]	[0.66, 0.77]	[1.47, 1.87]	[0.29, 0.34]	[0.48, 0.54]	[0.35, 0.39]	[0.24, 0.31]	[0.72, 0.77]	[1.64, 2.03]	[1.95, 2.24]	[0.37, 0.43]	[0.24, 0.31]
*boivini*	Minor (n= 49)	1.15±0.06	0.67±0.03	0.71±0.03	0.46±0.01	0.27±0.01	0.32±0.01	0.25±0.01	0.29±0.01	0.72±0.04	1.37±0.10	0.32±0.02	0.47±0.02	0.38±0.01	0.27±0.02	0.75±0.01	1.57±0.12	1.83±0.07	0.39±0.01	0.26±0.02
		[1.00, 1.27]	[0.61, 0.73]	[0.65, 0.77]	[0.43, 0.49]	[0.24, 0.30]	[0.30, 0.35]	[0.23, 0.26]	[0.27, 0.31]	[0.63, 0.79]	[1.22, 1.56]	[0.29, 0.36]	[0.44, 0.51]	[0.34, 0.40]	[0.22, 0.30]	[0.71, 0.78]	[1.41, 1.79]	[1.70, 2.00]	[0.37, 0.41]	[0.23, 0.30]
	Major (n= 13)	2.29±0.28	0.91±0.04	0.79±0.02	0.51±0.01	0.29±0.01	0.33±0.01	0.25±0.01	0.32±0.01	0.89±0.02	0.71±0.06	0.22±0.02	0.44±0.01	0.59±0.01	0.22±0.01	0.57±0.02	0.94±0.07	0.93±0.05	1.33±0.02	0.20±0.02
		[1.96, 3.12]	[0.86, 0.99]	[0.77, 0.83]	[0.49, 0.52]	[0.27, 0.33]	[0.32, 0.35]	[0.24, 0.26]	[0.30, 0.34]	[0.85, 0.93]	[0.58, 0.79]	[0.17, 0.24]	[0.42, 0.45]	[0.57, 0.62]	[0.20, 0.25]	[0.54, 0.60]	[0.75, 1.03]	[0.82, 1.00]	[1.29, 1.38]	[0.17, 0.22]
*bozaka*	Minor (n= 12)	1.33±0.13	0.77±0.03	0.78±0.02	0.51±0.01	0.25±0.01	0.28±0.01	0.24±0.01	0.30±0.01	0.74±0.03	1.19±0.05	0.28±0.01	0.46±0.01	0.40±0.01	0.27±0.01	0.74±0.02	1.31±0.06	1.67±0.07	0.40±0.01	0.29±0.02
		[1.16, 1.59]	[0.73, 0.84]	[0.76, 0.81]	[0.47, 0.53]	[0.23, 0.26]	[0.28, 0.29]	[0.23, 0.29]	[0.29, 0.32]	[0.70, 0.82]	[1.08, 1.25]	[0.26, 0.30]	[0.44, 0.47]	[0.38, 0.42]	[0.26, 0.28]	[0.71, 0.77]	[1.21, 1.38]	[1.55, 1.77]	[0.38, 0.43]	[0.27, 0.32]
	Major (n= 6)	2.07±0.18	0.95±0.03	0.86±0.01	0.51±0.02	0.27±0.01	0.28±0.01	0.24±0.01	0.32±0.01	0.85±0.02	0.90±0.04	0.23±0.01	0.44±0.01	0.58±0.02	0.24±0.01	0.57±0.01	1.02±0.04	1.02±0.04	1.33±0.02	0.25±0.02
		[1.91, 2.40]	[0.90, 0.98]	[0.85, 0.88]	[0.49, 0.53]	[0.25, 0.29]	[0.28, 0.30]	[0.23, 0.25]	[0.31, 0.33]	[0.83, 0.88]	[0.86, 0.94]	[0.21, 0.25]	[0.43, 0.45]	[0.56, 0.61]	[0.23, 0.25]	[0.55, 0.58]	[0.98, 1.08]	[0.96, 1.08]	[1.31, 1.38]	[0.22, 0.26]
*cemeryi*	Minor (n= 9)	1.11±0.07	0.67±0.02	0.72±0.02	0.47±0.01	0.25±0.02	0.28±0.01	0.24±0.01	0.27±0.01	0.66±0.03	1.49±0.05	0.33±0.01	0.48±0.01	0.41±0.02	0.29±0.02	0.76±0.02	1.58±0.06	1.88±0.06	0.42±0.02	0.27±0.03
		[0.98, 1.28]	[0.64, 0.71]	[0.67, 0.75]	[0.45, 0.49]	[0.22, 0.29]	[0.26, 0.30]	[0.22, 0.25]	[0.26, 0.29]	[0.62, 0.72]	[1.41, 1.60]	[0.31, 0.35]	[0.47, 0.50]	[0.38, 0.43]	[0.27, 0.34]	[0.72, 0.79]	[1.48, 1.76]	[1.77, 2.01]	[0.39, 0.46]	[0.21, 0.31]
	Major (n= 7)	2.41±0.22	0.87±0.05	0.75±0.03	0.51±0.01	0.29±0.01	0.28±0.01	0.22±0.01	0.30±0.01	0.86±0.03	0.80±0.07	0.21±0.01	0.46±0.02	0.59±0.02	0.23±0.01	0.58±0.01	0.99±0.06	0.97±0.04	1.32±0.03	0.21±0.02
		[2.19, 2.72]	[0.83, 0.94]	[0.71, 0.79]	[0.50, 0.52]	[0.27, 0.30]	[0.26, 0.30]	[0.21, 0.23]	[0.29, 0.31]	[0.83, 0.90]	[0.70, 0.91]	[0.19, 0.23]	[0.43, 0.48]	[0.57, 0.63]	[0.22, 0.25]	[0.56, 0.61]	[0.86, 1.06]	[0.92, 1.02]	[1.29, 1.35]	[0.17, 0.23]
*cervicalis*	Minor (n= 8)	1.94±0.15	0.59±0.03	0.58±0.01	0.47±0.02	0.30±0.01	0.27±0.01	0.23±0.01	0.30±0.01	0.81±0.02	1.81±0.12	0.28±0.01	0.54±0.01	0.34±0.01	0.27±0.01	0.71±0.01	2.15±0.12	2.08±0.08	0.36±0.02	0.29±0.02
		[1.83, 2.18]	[0.56, 0.65]	[0.56, 0.60]	[0.45, 0.49]	[0.29, 0.31]	[0.26, 0.28]	[0.22, 0.24]	[0.28, 0.31]	[0.79, 0.85]	[1.57, 1.94]	[0.26, 0.30]	[0.52, 0.56]	[0.32, 0.36]	[0.25, 0.29]	[0.69, 0.73]	[1.91, 2.33]	[1.96, 2.15]	[0.34, 0.38]	[0.27, 0.32]
	Major (n= 5)	4.17±0.35	0.91±0.03	0.70±0.01	0.53±0.01	0.28±0.01	0.25±0.01	0.21±0.01	0.33±0.01	0.98±0.03	0.95±0.06	0.19±0.01	0.47±0.01	0.56±0.02	0.22±0.01	0.52±0.01	1.23±0.07	1.00±0.04	1.35±0.02	0.21±0.01
		[3.83, 4.63]	[0.85, 0.93]	[0.68, 0.71]	[0.52, 0.54]	[0.28, 0.29]	[0.24, 0.27]	[0.20, 0.23]	[0.31, 0.33]	[0.94, 1.01]	[0.89, 1.04]	[0.18, 0.21]	[0.46, 0.49]	[0.53, 0.58]	[0.21, 0.23]	[0.51, 0.53]	[1.18, 1.35]	[0.97, 1.07]	[1.32, 1.36]	[0.19, 0.22]
*daraina*	Minor (n= 19)	1.87±0.17	0.60±0.02	0.59±0.01	0.48±0.01	0.29±0.01	0.25±0.00	0.22±0.01	0.28±0.01	0.75±0.02	1.72±0.06	0.28±0.01	0.52±0.01	0.34±0.01	0.28±0.01	0.72±0.01	2.04±0.05	2.09±0.04	0.37±0.01	0.30±0.02
		[1.68, 2.31]	[0.57, 0.66]	[0.57, 0.62]	[0.45, 0.50]	[0.28, 0.30]	[0.24, 0.26]	[0.21, 0.23]	[0.27, 0.30]	[0.71, 0.79]	[1.56, 1.82]	[0.26, 0.30]	[0.50, 0.52]	[0.32, 0.36]	[0.26, 0.31]	[0.71, 0.74]	[1.93, 2.15]	[1.95, 2.16]	[0.35, 0.38]	[0.26, 0.32]
	Major (n= 5)	3.54±0.25	0.90±0.04	0.72±0.02	0.53±0.01	0.28±0.01	0.25±0.01	0.21±0.01	0.30±0.01	0.86±0.03	0.96±0.06	0.20±0.01	0.47±0.01	0.56±0.01	0.22±0.01	0.54±0.01	1.23±0.06	1.03±0.03	1.35±0.03	0.20±0.01
		[3.12, 3.76]	[0.85, 0.94]	[0.70, 0.75]	[0.52, 0.55]	[0.28, 0.29]	[0.24, 0.26]	[0.20, 0.21]	[0.30, 0.31]	[0.82, 0.89]	[0.90, 1.04]	[0.18, 0.22]	[0.46, 0.48]	[0.54, 0.58]	[0.21, 0.24]	[0.53, 0.54]	[1.18, 1.31]	[1.00, 1.07]	[1.32, 1.40]	[0.19, 0.21]
*dufouri*	Minor (n= 19)	1.97±0.19	0.56±0.02	0.55±0.02	0.45±0.01	0.33±0.01	0.25±0.01	0.21±0.01	0.27±0.01	0.82±0.03	1.93±0.08	0.28±0.02	0.51±0.01	0.28±0.01	0.24±0.01	0.67±0.01	2.37±0.09	2.15±0.08	0.29±0.01	0.29±0.02
		[1.66, 2.26]	[0.53, 0.59]	[0.53, 0.59]	[0.43, 0.47]	[0.31, 0.36]	[0.24, 0.27]	[0.19, 0.22]	[0.26, 0.28]	[0.77, 0.86]	[1.76, 2.12]	[0.25, 0.31]	[0.49, 0.52]	[0.27, 0.32]	[0.22, 0.27]	[0.65, 0.71]	[2.23, 2.57]	[1.98, 2.31]	[0.27, 0.32]	[0.26, 0.33]
	Major (n= 5)	3.93±0.51	0.88±0.08	0.68±0.03	0.51±0.02	0.32±0.01	0.24±0.01	0.20±0.01	0.30±0.01	0.94±0.02	1.02±0.08	0.19±0.01	0.47±0.01	0.51±0.02	0.18±0.01	0.46±0.02	1.34±0.13	0.99±0.06	1.38±0.04	0.19±0.02
		[3.46, 4.54]	[0.80, 0.97]	[0.65, 0.72]	[0.49, 0.53]	[0.30, 0.33]	[0.23, 0.25]	[0.19, 0.21]	[0.29, 0.31]	[0.93, 0.98]	[0.94, 1.10]	[0.18, 0.20]	[0.46, 0.47]	[0.49, 0.54]	[0.17, 0.19]	[0.44, 0.48]	[1.18, 1.46]	[0.92, 1.06]	[1.33, 1.41]	[0.18, 0.22]
*gibber*	Minor (n= 37)	1.24±0.13	0.85±0.03	0.83±0.02	0.50±0.01	0.25±0.01	0.35±0.01	0.28±0.01	0.31±0.01	0.69±0.04	1.17±0.07	0.27±0.01	0.46±0.01	0.44±0.02	0.29±0.01	0.74±0.02	1.20±0.07	1.57±0.07	0.36±0.02	0.21±0.02
		[1.00, 1.51]	[0.79, 0.93]	[0.79, 0.89]	[0.47, 0.54]	[0.23, 0.28]	[0.33, 0.36]	[0.26, 0.29]	[0.29, 0.33]	[0.62, 0.76]	[0.93, 1.27]	[0.24, 0.30]	[0.44, 0.49]	[0.40, 0.50]	[0.27, 0.32]	[0.71, 0.79]	[1.00, 1.30]	[1.38, 1.67]	[0.32, 0.41]	[0.17, 0.24]
	Major (n= 9)	2.45±0.21	1.05±0.04	0.90±0.04	0.52±0.03	0.28±0.02	0.33±0.02	0.26±0.01	0.33±0.01	0.83±0.03	0.74±0.06	0.21±0.01	0.45±0.02	0.66±0.02	0.25±0.02	0.54±0.01	0.86±0.05	0.88±0.04	1.32±0.03	0.17±0.01
		[2.21, 2.82]	[1.01, 1.10]	[0.86, 0.97]	[0.50, 0.60]	[0.26, 0.31]	[0.31, 0.36]	[0.24, 0.29]	[0.31, 0.36]	[0.76, 0.86]	[0.68, 0.85]	[0.19, 0.22]	[0.43, 0.50]	[0.63, 0.70]	[0.22, 0.27]	[0.52, 0.57]	[0.81, 0.98]	[0.84, 0.97]	[1.28, 1.37]	[0.15, 0.19]
*gouldi*	Minor (n= 18)	2.52±0.17	0.66±0.02	0.62±0.01	0.50±0.01	0.29±0.02	0.26±0.01	0.23±0.01	0.29±0.01	0.77±0.11	1.56±0.06	0.24±0.01	0.49±0.01	0.47±0.01	0.25±0.01	0.49±0.02	2.11±0.08	1.47±0.03	1.36±0.03	0.29±0.02
		[2.29, 2.89]	[0.64, 0.70]	[0.60, 0.65]	[0.49, 0.55]	[0.26, 0.32]	[0.25, 0.27]	[0.21, 0.24]	[0.27, 0.31]	[0.68, 1.16]	[1.44, 1.66]	[0.23, 0.26]	[0.47, 0.50]	[0.44, 0.48]	[0.23, 0.27]	[0.47, 0.53]	[1.97, 2.26]	[1.42, 1.51]	[1.31, 1.41]	[0.24, 0.31]
	Major (n= 6)	5.19±0.23	0.98±0.01	0.76±0.01	0.55±0.01	0.28±0.01	0.24±0.01	0.21±0.00	0.33±0.01	0.93±0.02	0.89±0.05	0.17±0.01	0.47±0.01	0.57±0.01	0.20±0.01	0.53±0.02	1.19±0.05	0.96±0.02	1.34±0.03	0.23±0.02
		[4.89, 5.51]	[0.97, 1.00]	[0.74, 0.76]	[0.54, 0.55]	[0.27, 0.30]	[0.23, 0.25]	[0.20, 0.21]	[0.32, 0.34]	[0.90, 0.95]	[0.84, 0.95]	[0.16, 0.18]	[0.46, 0.48]	[0.57, 0.58]	[0.19, 0.21]	[0.50, 0.55]	[1.16, 1.26]	[0.93, 0.99]	[1.31, 1.38]	[0.20, 0.24]
*hagensii*	Minor (n= 17)	1.45±0.14	0.80±0.03	0.80±0.02	0.48±0.01	0.25±0.01	0.34±0.01	0.27±0.01	0.31±0.01	0.74±0.04	1.24±0.06	0.30±0.01	0.46±0.01	0.41±0.01	0.30±0.01	0.75±0.01	1.37±0.06	1.66±0.04	0.38±0.02	0.24±0.02
		[1.30, 1.68]	[0.74, 0.84]	[0.76, 0.83]	[0.46, 0.50]	[0.23, 0.25]	[0.31, 0.35]	[0.26, 0.29]	[0.30, 0.34]	[0.68, 0.85]	[1.09, 1.36]	[0.28, 0.33]	[0.43, 0.48]	[0.39, 0.42]	[0.27, 0.32]	[0.73, 0.77]	[1.23, 1.48]	[1.56, 1.74]	[0.36, 0.41]	[0.21, 0.27]
	Major (n= 5)	2.37±0.26	0.97±0.05	0.87±0.03	0.50±0.01	0.26±0.02	0.33±0.01	0.27±0.01	0.34±0.01	0.86±0.02	0.86±0.09	0.24±0.02	0.45±0.01	0.61±0.02	0.25±0.02	0.56±0.02	1.03±0.09	0.98±0.06	1.33±0.04	0.19±0.01
		[2.00, 2.70]	[0.92, 1.04]	[0.85, 0.91]	[0.50, 0.51]	[0.24, 0.28]	[0.31, 0.35]	[0.25, 0.27]	[0.33, 0.34]	[0.84, 0.90]	[0.74, 0.97]	[0.22, 0.27]	[0.44, 0.47]	[0.58, 0.64]	[0.22, 0.26]	[0.54, 0.58]	[0.90, 1.12]	[0.92, 1.07]	[1.27, 1.38]	[0.18, 0.21]
*harenarum*	Minor (n= 5)	1.53±0.11	0.79±0.01	0.72±0.01	0.50±0.01	0.28±0.01	0.25±0.00	0.21±0.00	0.29±0.01	0.68±0.03	1.63±0.05	0.25±0.01	0.51±0.01	0.38±0.01	0.26±0.01	0.74±0.01	2.05±0.04	2.00±0.03	0.36±0.01	0.23±0.01
		[1.38, 1.63]	[0.78, 0.80]	[0.72, 0.73]	[0.50, 0.51]	[0.27, 0.29]	[0.24, 0.25]	[0.21, 0.21]	[0.28, 0.30]	[0.64, 0.71]	[1.58, 1.70]	[0.25, 0.26]	[0.50, 0.52]	[0.37, 0.39]	[0.24, 0.28]	[0.73, 0.76]	[2.02, 2.10]	[1.98, 2.05]	[0.36, 0.37]	[0.22, 0.25]
*hova*	Minor (n= 27)	1.55±0.10	0.66±0.03	0.66±0.02	0.49±0.02	0.25±0.02	0.27±0.01	0.23±0.01	0.29±0.01	0.73±0.03	1.53±0.08	0.30±0.01	0.50±0.01	0.37±0.02	0.27±0.02	0.75±0.02	1.68±0.09	1.92±0.06	0.38±0.02	0.25±0.02
		[1.40, 1.74]	[0.63, 0.71]	[0.63, 0.70]	[0.47, 0.52]	[0.22, 0.28]	[0.24, 0.30]	[0.21, 0.25]	[0.27, 0.30]	[0.67, 0.78]	[1.41, 1.83]	[0.28, 0.32]	[0.48, 0.52]	[0.34, 0.41]	[0.23, 0.30]	[0.71, 0.78]	[1.51, 1.86]	[1.80, 2.04]	[0.34, 0.42]	[0.19, 0.29]
	Major (n= 10)	3.17±0.16	0.94±0.03	0.77±0.01	0.53±0.01	0.27±0.01	0.26±0.01	0.22±0.01	0.30±0.01	0.86±0.02	0.87±0.04	0.21±0.01	0.47±0.01	0.58±0.02	0.22±0.01	0.55±0.02	1.06±0.06	0.97±0.04	1.34±0.04	0.18±0.01
		[2.94, 3.48]	[0.89, 1.01]	[0.75, 0.79]	[0.52, 0.55]	[0.26, 0.28]	[0.23, 0.27]	[0.21, 0.23]	[0.29, 0.31]	[0.83, 0.90]	[0.80, 0.92]	[0.19, 0.23]	[0.46, 0.48]	[0.55, 0.62]	[0.21, 0.24]	[0.54, 0.58]	[0.95, 1.12]	[0.91, 1.02]	[1.30, 1.46]	[0.17, 0.19]
*hovahovoides*	Minor (n= 55)	1.56±0.13	0.65±0.02	0.67±0.02	0.47±0.01	0.28±0.01	0.30±0.01	0.25±0.01	0.29±0.01	0.75±0.03	1.54±0.08	0.30±0.01	0.49±0.01	0.35±0.01	0.29±0.02	0.72±0.02	1.77±0.09	1.91±0.06	0.35±0.01	0.30±0.02
		[1.16, 1.83]	[0.59, 0.68]	[0.63, 0.71]	[0.43, 0.49]	[0.25, 0.31]	[0.28, 0.33]	[0.22, 0.26]	[0.27, 0.31]	[0.69, 0.83]	[1.43, 1.75]	[0.27, 0.34]	[0.46, 0.51]	[0.32, 0.38]	[0.25, 0.34]	[0.67, 0.76]	[1.62, 2.00]	[1.79, 2.11]	[0.33, 0.39]	[0.25, 0.35]
	Major (n= 17)	2.66±0.20	0.90±0.04	0.77±0.02	0.50±0.01	0.29±0.01	0.29±0.02	0.23±0.01	0.32±0.01	0.93±0.04	0.92±0.07	0.22±0.01	0.45±0.02	0.56±0.02	0.22±0.01	0.52±0.02	1.14±0.06	1.00±0.04	1.37±0.03	0.21±0.01
		[2.40, 3.07]	[0.85, 0.98]	[0.72, 0.80]	[0.48, 0.51]	[0.27, 0.32]	[0.25, 0.31]	[0.20, 0.24]	[0.30, 0.33]	[0.88, 1.01]	[0.75, 1.01]	[0.20, 0.23]	[0.43, 0.48]	[0.54, 0.60]	[0.20, 0.26]	[0.47, 0.55]	[1.01, 1.21]	[0.91, 1.06]	[1.32, 1.42]	[0.18, 0.23]
*imitator*	Minor (n= 44)	2.42±0.88	0.85±0.10	0.76±0.04	0.54±0.02	0.27±0.02	0.26±0.01	0.23±0.01	0.28±0.02	0.71±0.07	1.21±0.36	0.21±0.03	0.45±0.04	0.35±0.06	0.23±0.02	0.62±0.05	1.69±0.46	1.67±0.34	0.28±0.04	0.22±0.03
		[1.34, 3.94]	[0.72, 1.03]	[0.68, 0.84]	[0.42, 0.57]	[0.22, 0.32]	[0.21, 0.28]	[0.16, 0.25]	[0.23, 0.32]	[0.53, 0.83]	[0.71, 1.73]	[0.16, 0.27]	[0.33, 0.51]	[0.27, 0.46]	[0.16, 0.26]	[0.49, 0.70]	[1.01, 2.39]	[1.17, 2.15]	[0.22, 0.35]	[0.16, 0.27]
*immaculatus*	Minor (n= 12)	1.20±0.13	0.79±0.03	0.78±0.02	0.51±0.01	0.26±0.02	0.32±0.01	0.26±0.01	0.30±0.01	0.70±0.03	1.28±0.05	0.25±0.01	0.48±0.01	0.44±0.01	0.27±0.01	0.74±0.01	1.34±0.05	1.66±0.06	0.35±0.01	0.21±0.02
		[1.01, 1.45]	[0.73, 0.83]	[0.73, 0.80]	[0.48, 0.53]	[0.22, 0.28]	[0.30, 0.33]	[0.24, 0.28]	[0.29, 0.32]	[0.65, 0.74]	[1.19, 1.34]	[0.23, 0.28]	[0.47, 0.50]	[0.41, 0.47]	[0.24, 0.29]	[0.72, 0.76]	[1.26, 1.41]	[1.56, 1.73]	[0.33, 0.37]	[0.18, 0.24]
	Major (n= 7)	2.32±0.12	1.00±0.03	0.85±0.01	0.53±0.01	0.28±0.02	0.32±0.01	0.25±0.01	0.32±0.01	0.84±0.01	0.82±0.03	0.19±0.00	0.47±0.01	0.65±0.01	0.23±0.01	0.52±0.02	0.95±0.05	0.95±0.01	1.33±0.02	0.16±0.01
		[2.13, 2.46]	[0.96, 1.04]	[0.83, 0.86]	[0.52, 0.56]	[0.25, 0.30]	[0.31, 0.33]	[0.24, 0.26]	[0.31, 0.33]	[0.82, 0.86]	[0.76, 0.87]	[0.19, 0.19]	[0.45, 0.48]	[0.63, 0.66]	[0.22, 0.25]	[0.50, 0.55]	[0.85, 1.00]	[0.93, 0.97]	[1.30, 1.36]	[0.14, 0.17]
*joany*	Minor (n= 2)	1.54±0.09	0.73±0.01	0.68±0.01	0.51±0.00	0.26±0.00	0.27±0.00	0.22±0.00	0.28±0.00	0.68±0.02	1.62±0.01	0.27±0.00	0.50±0.00	0.39±0.02	0.28±0.00	0.76±0.00	1.95±0.01	2.00±0.00	0.39±0.00	0.23±0.01
		[1.48, 1.60]	[0.72, 0.73]	[0.68, 0.69]	[0.50, 0.51]	[0.26, 0.26]	[0.27, 0.27]	[0.22, 0.23]	[0.28, 0.29]	[0.67, 0.69]	[1.61, 1.63]	[0.27, 0.28]	[0.49, 0.50]	[0.38, 0.40]	[0.28, 0.28]	[0.76, 0.77]	[1.95, 1.96]	[2.00, 2.01]	[0.39, 0.40]	[0.22, 0.23]
*karsti*	Minor (n= 2)	1.83±0.09	0.70±0.00	0.73±0.01	0.52±0.00	0.26±0.01	0.25±0.00	0.21±0.00	0.30±0.00	0.75±0.01	1.65±0.03	0.26±0.01	0.53±0.00	0.37±0.01	0.21±0.00	0.42±0.01	2.08±0.02	1.93±0.02	0.26±0.00	0.24±0.00
		[1.77, 1.89]	[0.70, 0.70]	[0.72, 0.73]	[0.52, 0.52]	[0.25, 0.27]	[0.25, 0.25]	[0.21, 0.22]	[0.29, 0.30]	[0.75, 0.76]	[1.63, 1.67]	[0.25, 0.26]	[0.53, 0.53]	[0.36, 0.37]	[0.20, 0.21]	[0.41, 0.42]	[2.06, 2.09]	[1.92, 1.94]	[0.26, 0.26]	[0.24, 0.24]
*kelimaso*	Minor (n= 17)	1.68±0.12	0.84±0.02	0.79±0.02	0.54±0.06	0.28±0.01	0.31±0.02	0.26±0.01	0.35±0.01	0.81±0.03	1.19±0.05	0.19±0.01	0.49±0.01	0.42±0.01	0.26±0.02	0.72±0.02	1.27±0.04	1.55±0.06	0.38±0.01	0.25±0.01
		[1.51, 1.88]	[0.80, 0.88]	[0.76, 0.83]	[0.31, 0.57]	[0.27, 0.29]	[0.28, 0.34]	[0.25, 0.27]	[0.33, 0.37]	[0.75, 0.88]	[1.11, 1.27]	[0.16, 0.21]	[0.46, 0.51]	[0.38, 0.44]	[0.22, 0.28]	[0.65, 0.74]	[1.20, 1.38]	[1.45, 1.73]	[0.35, 0.40]	[0.22, 0.27]
	Major (n= 7)	2.83±0.25	0.99±0.04	0.85±0.01	0.54±0.01	0.31±0.01	0.31±0.01	0.25±0.01	0.35±0.01	0.92±0.04	0.82±0.07	0.15±0.01	0.45±0.01	0.67±0.02	0.23±0.02	0.56±0.02	0.93±0.06	0.89±0.06	1.35±0.03	0.20±0.02
		[2.35, 3.15]	[0.92, 1.04]	[0.84, 0.87]	[0.53, 0.56]	[0.30, 0.32]	[0.30, 0.33]	[0.24, 0.27]	[0.33, 0.37]	[0.90, 1.01]	[0.77, 0.96]	[0.14, 0.17]	[0.44, 0.46]	[0.65, 0.70]	[0.21, 0.25]	[0.52, 0.60]	[0.88, 1.06]	[0.84, 1.02]	[1.30, 1.39]	[0.16, 0.24]
*liandia*	Minor (n= 44)	1.04±0.11	0.77±0.02	0.76±0.02	0.51±0.01	0.26±0.02	0.30±0.01	0.24±0.01	0.29±0.01	0.66±0.03	1.22±0.08	0.25±0.02	0.48±0.01	0.44±0.02	0.26±0.01	0.73±0.02	1.27±0.07	1.64±0.07	0.35±0.02	0.21±0.02
		[0.88, 1.53]	[0.73, 0.85]	[0.69, 0.79]	[0.48, 0.54]	[0.23, 0.29]	[0.27, 0.34]	[0.22, 0.27]	[0.27, 0.31]	[0.59, 0.76]	[0.87, 1.33]	[0.20, 0.29]	[0.46, 0.51]	[0.37, 0.48]	[0.23, 0.30]	[0.68, 0.75]	[0.98, 1.36]	[1.38, 1.78]	[0.32, 0.40]	[0.16, 0.24]
	Major (n= 12)	1.95±0.16	0.89±0.01	0.78±0.01	0.53±0.01	0.29±0.01	0.30±0.01	0.23±0.01	0.30±0.00	0.84±0.03	0.75±0.06	0.19±0.01	0.47±0.01	0.66±0.01	0.24±0.01	0.53±0.01	0.92±0.05	0.94±0.01	1.34±0.02	0.18±0.01
		[1.73, 2.15]	[0.85, 0.93]	[0.73, 0.84]	[0.52, 0.55]	[0.27, 0.32]	[0.28, 0.31]	[0.21, 0.25]	[0.29, 0.32]	[0.76, 0.91]	[0.69, 0.86]	[0.17, 0.19]	[0.45, 0.48]	[0.64, 0.70]	[0.21, 0.27]	[0.48, 0.58]	[0.83, 0.99]	[0.92, 0.97]	[1.28, 1.37]	[0.16, 0.19]
*lokobe*	Minor (n= 8)	1.65±0.05	0.64±0.01	0.64±0.01	0.46±0.01	0.29±0.01	0.26±0.01	0.22±0.01	0.26±0.01	0.72±0.02	1.80±0.03	0.30±0.01	0.50±0.01	0.29±0.00	0.21±0.00	0.67±0.01	2.28±0.04	2.11±0.02	0.29±0.01	0.26±0.01
		[1.60, 1.72]	[0.62, 0.66]	[0.63, 0.66]	[0.44, 0.48]	[0.28, 0.31]	[0.25, 0.27]	[0.22, 0.23]	[0.25, 0.27]	[0.69, 0.75]	[1.76, 1.85]	[0.27, 0.31]	[0.49, 0.51]	[0.28, 0.29]	[0.21, 0.22]	[0.65, 0.68]	[2.24, 2.33]	[2.09, 2.14]	[0.29, 0.30]	[0.24, 0.27]
*mahafaly*	Minor (n= 19)	1.05±0.05	0.70±0.02	0.74±0.02	0.50±0.01	0.23±0.01	0.27±0.01	0.23±0.01	0.27±0.01	0.62±0.02	1.40±0.04	0.32±0.01	0.44±0.01	0.38±0.03	0.25±0.01	0.72±0.06	1.56±0.02	1.81±0.12	0.37±0.03	0.25±0.02
		[0.93, 1.13]	[0.65, 0.73]	[0.71, 0.78]	[0.48, 0.52]	[0.20, 0.24]	[0.25, 0.28]	[0.20, 0.24]	[0.26, 0.28]	[0.58, 0.65]	[1.33, 1.45]	[0.30, 0.35]	[0.43, 0.47]	[0.35, 0.50]	[0.23, 0.28]	[0.67, 0.97]	[1.52, 1.60]	[1.35, 1.90]	[0.34, 0.50]	[0.23, 0.29]
*mixtellus*	Minor (n= 25)	1.59±0.10	0.63±0.02	0.64±0.02	0.46±0.01	0.30±0.01	0.28±0.01	0.23±0.01	0.28±0.01	0.76±0.04	1.55±0.07	0.30±0.02	0.49±0.01	0.37±0.01	0.29±0.01	0.75±0.02	1.77±0.08	1.95±0.05	0.39±0.02	0.28±0.02
		[1.36, 1.75]	[0.58, 0.66]	[0.62, 0.68]	[0.44, 0.49]	[0.28, 0.31]	[0.26, 0.30]	[0.21, 0.24]	[0.26, 0.31]	[0.69, 0.85]	[1.40, 1.73]	[0.27, 0.33]	[0.46, 0.51]	[0.34, 0.39]	[0.27, 0.30]	[0.71, 0.78]	[1.64, 1.92]	[1.85, 2.06]	[0.36, 0.42]	[0.23, 0.30]
	Major (n= 8)	2.92±0.15	0.93±0.03	0.77±0.02	0.50±0.01	0.29±0.02	0.28±0.01	0.22±0.01	0.30±0.01	0.93±0.02	0.82±0.05	0.22±0.01	0.44±0.01	0.58±0.01	0.22±0.01	0.54±0.01	1.05±0.05	0.97±0.02	1.32±0.03	0.19±0.01
		[2.62, 3.10]	[0.90, 0.99]	[0.75, 0.80]	[0.48, 0.52]	[0.27, 0.31]	[0.26, 0.29]	[0.20, 0.23]	[0.28, 0.31]	[0.89, 0.96]	[0.77, 0.91]	[0.21, 0.24]	[0.43, 0.48]	[0.56, 0.60]	[0.20, 0.23]	[0.53, 0.56]	[0.98, 1.12]	[0.95, 1.01]	[1.28, 1.37]	[0.17, 0.20]
*niavo*	Minor (n= 8)	2.20±0.15	0.59±0.03	0.61±0.03	0.50±0.03	0.28±0.02	0.25±0.01	0.22±0.01	0.30±0.02	0.81±0.04	1.70±0.11	0.27±0.01	0.51±0.04	0.34±0.03	0.29±0.02	0.72±0.01	2.05±0.17	2.05±0.12	0.37±0.01	0.30±0.02
		[1.98, 2.38]	[0.55, 0.61]	[0.57, 0.65]	[0.46, 0.53]	[0.25, 0.32]	[0.24, 0.28]	[0.21, 0.23]	[0.28, 0.34]	[0.74, 0.87]	[1.59, 1.88]	[0.26, 0.30]	[0.44, 0.56]	[0.28, 0.37]	[0.26, 0.32]	[0.71, 0.74]	[1.87, 2.34]	[1.93, 2.26]	[0.35, 0.38]	[0.28, 0.32]
	Major (n= 7)	4.02±0.35	0.92±0.08	0.73±0.04	0.53±0.01	0.28±0.01	0.24±0.01	0.20±0.01	0.31±0.01	0.90±0.05	0.95±0.12	0.19±0.01	0.48±0.02	0.55±0.02	0.22±0.02	0.54±0.01	1.25±0.16	1.00±0.08	1.34±0.04	0.21±0.02
		[3.69, 4.61]	[0.79, 1.01]	[0.66, 0.76]	[0.52, 0.55]	[0.26, 0.30]	[0.24, 0.26]	[0.20, 0.21]	[0.29, 0.33]	[0.83, 0.97]	[0.81, 1.19]	[0.18, 0.22]	[0.46, 0.51]	[0.52, 0.58]	[0.20, 0.26]	[0.53, 0.55]	[1.11, 1.55]	[0.93, 1.17]	[1.31, 1.41]	[0.18, 0.25]
*quadrimaculatus*	Minor (n= 72)	1.18±0.14	0.84±0.03	0.82±0.02	0.51±0.02	0.25±0.01	0.34±0.01	0.28±0.01	0.30±0.01	0.66±0.04	1.23±0.07	0.26±0.02	0.48±0.01	0.47±0.01	0.28±0.01	0.75±0.02	1.22±0.07	1.58±0.07	0.37±0.02	0.21±0.02
		[0.86, 1.58]	[0.79, 0.93]	[0.79, 0.86]	[0.48, 0.54]	[0.22, 0.28]	[0.31, 0.37]	[0.25, 0.30]	[0.27, 0.35]	[0.58, 0.74]	[1.01, 1.36]	[0.21, 0.30]	[0.44, 0.51]	[0.44, 0.51]	[0.25, 0.31]	[0.62, 0.78]	[1.04, 1.38]	[1.40, 1.70]	[0.33, 0.41]	[0.16, 0.25]
	Major (n= 8)	2.44±0.14	1.04±0.03	0.88±0.02	0.53±0.01	0.28±0.02	0.34±0.00	0.26±0.00	0.32±0.01	0.84±0.04	0.78±0.03	0.20±0.01	0.46±0.01	0.69±0.02	0.24±0.02	0.54±0.03	0.88±0.02	0.87±0.04	1.26±0.14	0.16±0.01
		[2.19, 2.66]	[1.00, 1.08]	[0.85, 0.90]	[0.50, 0.54]	[0.26, 0.31]	[0.34, 0.35]	[0.26, 0.27]	[0.30, 0.33]	[0.77, 0.88]	[0.75, 0.82]	[0.19, 0.21]	[0.44, 0.47]	[0.65, 0.72]	[0.21, 0.28]	[0.51, 0.58]	[0.85, 0.91]	[0.81, 0.93]	[0.95, 1.36]	[0.15, 0.18]
*radamae*	Minor (n= 24)	1.27±0.09	0.65±0.02	0.69±0.02	0.46±0.01	0.29±0.01	0.32±0.01	0.26±0.01	0.29±0.01	0.71±0.03	1.52±0.07	0.31±0.01	0.49±0.01	0.34±0.02	0.25±0.01	0.70±0.01	1.69±0.07	1.87±0.06	0.34±0.01	0.28±0.01
		[1.04, 1.45]	[0.62, 0.69]	[0.67, 0.72]	[0.44, 0.48]	[0.27, 0.31]	[0.30, 0.34]	[0.26, 0.27]	[0.28, 0.31]	[0.66, 0.75]	[1.42, 1.64]	[0.29, 0.34]	[0.46, 0.51]	[0.30, 0.37]	[0.23, 0.29]	[0.67, 0.72]	[1.59, 1.80]	[1.78, 1.99]	[0.31, 0.36]	[0.26, 0.30]
	Major (n= 7)	2.68±0.31	0.95±0.04	0.80±0.02	0.50±0.01	0.30±0.01	0.30±0.01	0.24±0.01	0.30±0.01	0.88±0.03	0.80±0.05	0.21±0.01	0.44±0.01	0.59±0.01	0.21±0.01	0.54±0.02	1.00±0.04	0.88±0.02	1.37±0.02	0.19±0.02
		[2.29, 3.02]	[0.89, 0.99]	[0.77, 0.85]	[0.49, 0.51]	[0.29, 0.32]	[0.27, 0.31]	[0.22, 0.25]	[0.30, 0.32]	[0.82, 0.91]	[0.75, 0.89]	[0.20, 0.22]	[0.42, 0.45]	[0.56, 0.60]	[0.20, 0.22]	[0.52, 0.57]	[0.94, 1.04]	[0.86, 0.92]	[1.34, 1.41]	[0.17, 0.22]
*roeseli*	Minor (n= 29)	2.12±0.16	0.64±0.02	0.60±0.01	0.50±0.01	0.28±0.01	0.24±0.01	0.22±0.01	0.31±0.01	0.77±0.03	1.70±0.08	0.27±0.01	0.52±0.01	0.35±0.02	0.28±0.01	0.75±0.02	1.92±0.08	2.01±0.06	0.39±0.02	0.25±0.02
		[1.82, 2.53]	[0.62, 0.69]	[0.57, 0.63]	[0.47, 0.52]	[0.26, 0.29]	[0.23, 0.27]	[0.21, 0.24]	[0.29, 0.33]	[0.73, 0.84]	[1.50, 1.81]	[0.25, 0.29]	[0.50, 0.54]	[0.32, 0.39]	[0.26, 0.31]	[0.71, 0.79]	[1.77, 2.05]	[1.88, 2.11]	[0.35, 0.43]	[0.20, 0.29]
	Major (n= 10)	4.07±0.19	0.89±0.04	0.69±0.02	0.55±0.01	0.27±0.01	0.23±0.01	0.20±0.00	0.32±0.01	0.88±0.02	0.98±0.05	0.19±0.01	0.48±0.01	0.56±0.02	0.21±0.01	0.54±0.04	1.19±0.08	0.99±0.04	1.35±0.03	0.18±0.01
		[3.81, 4.40]	[0.85, 0.96]	[0.66, 0.73]	[0.53, 0.56]	[0.26, 0.29]	[0.22, 0.24]	[0.20, 0.21]	[0.31, 0.33]	[0.85, 0.91]	[0.91, 1.04]	[0.18, 0.20]	[0.45, 0.50]	[0.53, 0.60]	[0.20, 0.23]	[0.44, 0.56]	[1.07, 1.27]	[0.92, 1.05]	[1.30, 1.40]	[0.16, 0.20]
*rotrae*	Minor (n= 18)	1.29±0.09	0.85±0.05	0.80±0.06	0.53±0.01	0.25±0.01	0.31±0.01	0.25±0.01	0.31±0.01	0.66±0.03	1.19±0.05	0.24±0.01	0.47±0.02	0.44±0.01	0.26±0.01	0.74±0.01	1.25±0.04	1.60±0.07	0.38±0.01	0.20±0.01
		[1.08, 1.42]	[0.81, 1.05]	[0.77, 1.05]	[0.51, 0.55]	[0.23, 0.25]	[0.29, 0.33]	[0.23, 0.26]	[0.29, 0.33]	[0.60, 0.71]	[1.08, 1.27]	[0.22, 0.26]	[0.40, 0.50]	[0.43, 0.47]	[0.23, 0.28]	[0.72, 0.75]	[1.19, 1.31]	[1.43, 1.76]	[0.36, 0.41]	[0.18, 0.24]
	Major (n= 4)	2.29±0.17	1.03±0.01	0.84±0.03	0.56±0.01	0.27±0.02	0.31±0.01	0.24±0.01	0.33±0.01	0.79±0.02	0.75±0.03	0.18±0.01	0.46±0.01	0.67±0.01	0.22±0.01	0.55±0.01	0.88±0.03	0.88±0.04	1.37±0.03	0.14±0.01
		[2.03, 2.42]	[1.02, 1.04]	[0.82, 0.88]	[0.54, 0.57]	[0.24, 0.28]	[0.30, 0.32]	[0.24, 0.25]	[0.32, 0.33]	[0.77, 0.81]	[0.71, 0.79]	[0.17, 0.19]	[0.45, 0.47]	[0.66, 0.67]	[0.21, 0.23]	[0.54, 0.56]	[0.85, 0.91]	[0.85, 0.94]	[1.34, 1.41]	[0.13, 0.16]
*sambiranoensis*	Minor (n= 8)	1.77±0.11	0.60±0.01	0.58±0.01	0.47±0.01	0.32±0.04	0.22±0.01	0.18±0.01	0.28±0.01	0.75±0.03	2.04±0.07	0.28±0.01	0.51±0.01	0.30±0.02	0.23±0.01	0.70±0.02	2.36±0.06	2.22±0.04	0.33±0.03	0.27±0.02
		[1.56, 1.89]	[0.59, 0.62]	[0.56, 0.60]	[0.46, 0.49]	[0.30, 0.42]	[0.21, 0.23]	[0.17, 0.19]	[0.26, 0.30]	[0.70, 0.79]	[1.97, 2.18]	[0.27, 0.29]	[0.49, 0.52]	[0.27, 0.32]	[0.21, 0.26]	[0.67, 0.72]	[2.29, 2.46]	[2.18, 2.30]	[0.28, 0.36]	[0.25, 0.31]
	Major (n= 1)	4.45	0.98	0.74	0.52	0.30	0.25	0.21	0.31	0.88	0.84	0.18	0.46	0.57	0.22	0.53	1.12	0.93	1.29	0.20
*strangulatus*	Minor (n= 18)	1.59±0.11	0.67±0.02	0.66±0.01	0.50±0.01	0.27±0.01	0.25±0.01	0.22±0.01	0.30±0.01	0.80±0.03	1.58±0.05	0.28±0.01	0.52±0.01	0.33±0.01	0.24±0.02	0.68±0.01	1.87±0.05	1.94±0.03	0.33±0.01	0.25±0.02
		[1.41, 1.83]	[0.63, 0.69]	[0.64, 0.69]	[0.48, 0.52]	[0.26, 0.29]	[0.24, 0.27]	[0.21, 0.23]	[0.26, 0.31]	[0.71, 0.85]	[1.50, 1.66]	[0.26, 0.30]	[0.51, 0.56]	[0.31, 0.35]	[0.22, 0.27]	[0.65, 0.69]	[1.80, 2.01]	[1.86, 1.99]	[0.31, 0.36]	[0.19, 0.27]
	Major (n= 6)	3.22±0.36	0.91±0.06	0.75±0.02	0.54±0.02	0.32±0.11	0.25±0.01	0.22±0.01	0.31±0.01	0.89±0.06	0.94±0.11	0.20±0.01	0.49±0.01	0.59±0.03	0.22±0.01	0.56±0.02	1.22±0.12	0.98±0.04	1.42±0.04	0.21±0.02
		[2.73, 3.74]	[0.80, 0.98]	[0.72, 0.78]	[0.51, 0.55]	[0.27, 0.54]	[0.24, 0.27]	[0.21, 0.23]	[0.30, 0.32]	[0.80, 0.93]	[0.82, 1.08]	[0.19, 0.23]	[0.48, 0.50]	[0.55, 0.64]	[0.21, 0.25]	[0.54, 0.59]	[1.06, 1.36]	[0.92, 1.03]	[1.38, 1.48]	[0.20, 0.23]
*tapia*	Minor (n= 17)	1.37±0.14	0.67±0.03	0.69±0.01	0.52±0.01	0.24±0.01	0.25±0.01	0.23±0.01	0.29±0.01	0.73±0.04	1.61±0.08	0.29±0.01	0.52±0.01	0.34±0.02	0.24±0.01	0.69±0.04	1.79±0.07	1.97±0.15	0.33±0.02	0.23±0.02
		[1.17, 1.63]	[0.64, 0.72]	[0.68, 0.71]	[0.49, 0.54]	[0.23, 0.25]	[0.23, 0.27]	[0.21, 0.25]	[0.26, 0.31]	[0.67, 0.79]	[1.48, 1.71]	[0.28, 0.31]	[0.51, 0.53]	[0.25, 0.36]	[0.22, 0.26]	[0.54, 0.72]	[1.67, 1.91]	[1.83, 2.52]	[0.25, 0.36]	[0.19, 0.26]
	Major (n= 5)	3.20±0.21	0.99±0.03	0.81±0.01	0.52±0.01	0.29±0.02	0.26±0.01	0.23±0.01	0.31±0.00	0.88±0.01	0.85±0.06	0.19±0.01	0.46±0.00	0.59±0.02	0.20±0.00	0.55±0.02	1.08±0.05	0.97±0.02	1.35±0.03	0.20±0.01
		[2.84, 3.38]	[0.94, 1.01]	[0.78, 0.82]	[0.50, 0.54]	[0.27, 0.32]	[0.25, 0.28]	[0.22, 0.24]	[0.31, 0.32]	[0.86, 0.89]	[0.81, 0.96]	[0.18, 0.21]	[0.46, 0.47]	[0.56, 0.61]	[0.20, 0.20]	[0.53, 0.59]	[1.04, 1.16]	[0.94, 1.00]	[1.31, 1.38]	[0.18, 0.21]
*tendryi*	Minor (n= 2)	1.57±0.01	0.54±0.04	0.55±0.03	0.44±0.00	0.31±0.02	0.25±0.01	0.20±0.00	0.26±0.02	0.77±0.02	1.95±0.00	0.28±0.01	0.51±0.00	0.28±0.01	0.25±0.02	0.69±0.01	2.28±0.01	2.08±0.06	0.30±0.00	0.28±0.01
		[1.56, 1.58]	[0.52, 0.57]	[0.52, 0.57]	[0.44, 0.44]	[0.30, 0.33]	[0.24, 0.26]	[0.20, 0.21]	[0.24, 0.27]	[0.75, 0.78]	[1.95, 1.96]	[0.27, 0.29]	[0.51, 0.51]	[0.28, 0.29]	[0.24, 0.26]	[0.69, 0.70]	[2.28, 2.29]	[2.04, 2.12]	[0.30, 0.30]	[0.27, 0.29]
*vano*	Minor (n= 2)	1.03±0.01	0.56±0.01	0.62±0.00	0.43±0.01	0.34±0.00	0.29±0.00	0.25±0.01	0.27±0.00	0.74±0.00	1.68±0.03	0.29±0.00	0.47±0.01	0.28±0.00	0.24±0.02	0.68±0.01	1.92±0.06	1.98±0.09	0.29±0.02	0.25±0.01
		[1.02, 1.03]	[0.55, 0.57]	[0.62, 0.62]	[0.42, 0.43]	[0.34, 0.34]	[0.29, 0.30]	[0.24, 0.25]	[0.26, 0.27]	[0.74, 0.74]	[1.66, 1.70]	[0.29, 0.29]	[0.46, 0.49]	[0.28, 0.29]	[0.22, 0.25]	[0.67, 0.69]	[1.88, 1.97]	[1.92, 2.04]	[0.28, 0.31]	[0.24, 0.25]
	Major (n= 1)	1.67	0.64	0.62	0.46	0.34	0.28	0.25	0.30	0.92	1.00	0.24	0.50	0.45	0.21	0.46	1.32	1.11	1.42	0.20

In some species, the existing samples were below the threshold level to apply the quantitative, morphometry-based classification methods and were not included in the analysis. However, the measurement values of the individual specimens were used in the description of each of these species whose delimitations are supported by the qualitative morphological traits and other data from their biology and ecology.

Morphometric data of the major workers are also provided in the description but were excluded in statistical analysis.

As different allometric properties are present in the Malagasy *Camponotus* (see Rakotonirina et al. 2016, 2017), we applied morphometric data obtained from minor workers for multivariate statistical analysis.

Species hypotheses were generated using the Nest Centroid clustering (NC-clustering) technique. The procedure followed Rakotonirina et al. (2016).

To validate the species boundaries and reliability of the clusters recognized by the exploratory analyses, cumulative Linear Discriminant Analysis (LDA) was performed repeatedly until the final classification, showing the highest posterior probability values, was attained.

## ﻿Results and discussion

### ﻿Multivariate statistical analysis of morphometric data

**Figure 1.**
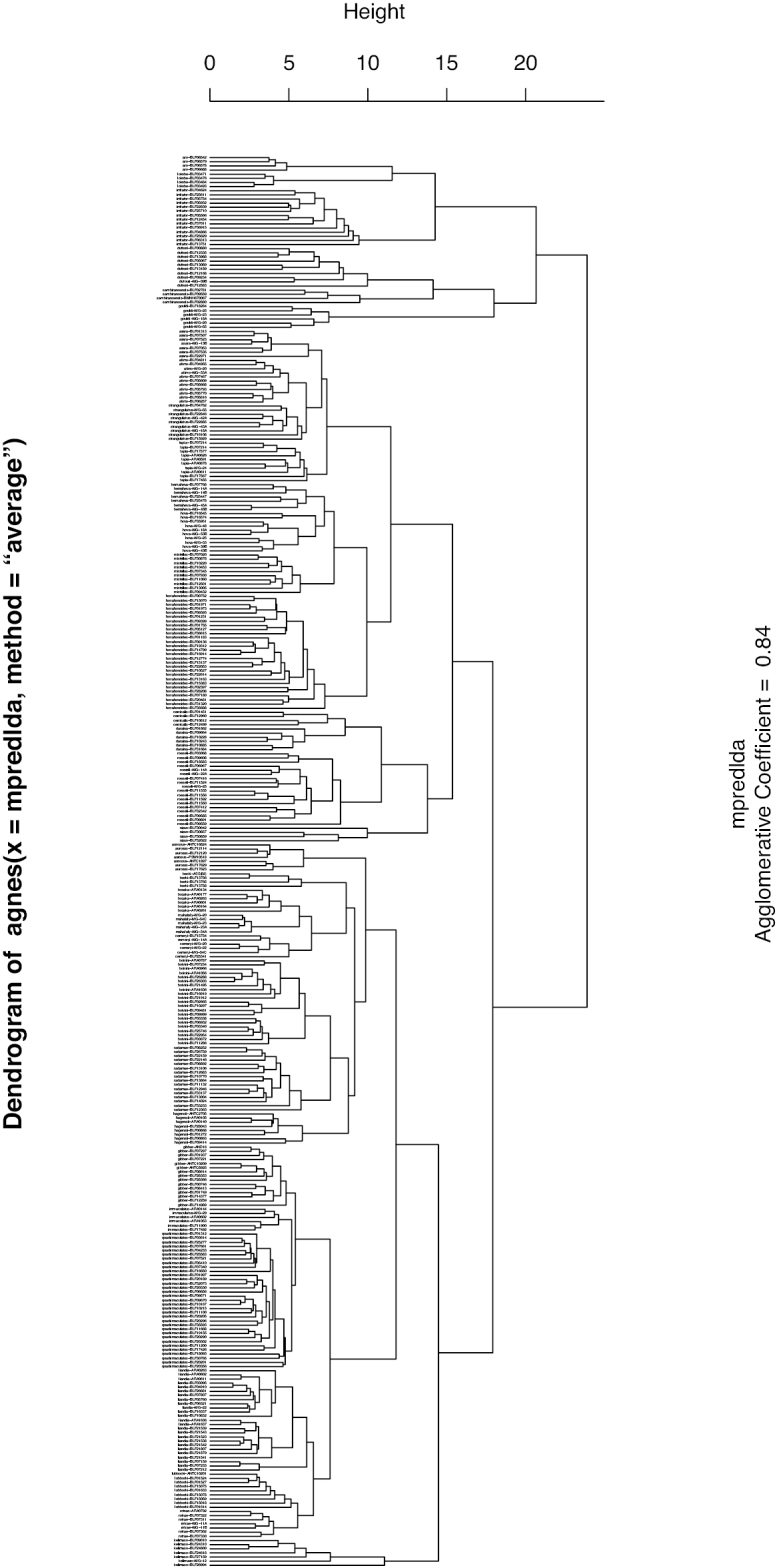
Dendrogram of NC-Clustering of the subgenus Myrmosaga. Label on the tip of the branch indicates the species name followed by the specimen code.

The NC-clustering dendrogram revealed 33 clusters, which are interpreted as 33 species (Fig. 1). The 33 species hypothesis proposed by the exploratory analysis was supported by the cumulative LDA, with 98.2% identification success (Table 3). Few species have conflicting identification features. These misidentifications could be attributed to similar morphology. Morphologically similar species frequently exhibit overlapping ranges of quantitative measurements and share qualitative morphological characters (Table 3).

**Table 3.** Classification matrix of species showing the classification success (percentage), the observed classification (rows) and the predicted classification (columns). Numbers in the matrix are specimen counts.

Observed species	Predicted species																																	
	*aro*	*asara*	*atimo*	*aurosus*	*becki*	*bemaheva*	*boivini*	*bozaka*	*cemeryi*	*cervicalis*	*daraina*	*dufouri*	*gibber*	*gouldi*	*hagensii*	*hova*	*hovahovoides*	*imitator*	*immaculatus*	*kelimaso*	*liandia*	*lokobe*	*lubbocki*	*mahafaly*	*mixtellus*	*niavo*	*quadrimaculatus*	*radamae*	*roeseli*	*rotrae*	*sambiranoensis*	*strangulatus*	*tapia*	Identification success (%)
*aro*	10	0	0	0	0	0	0	0	0	0	0	0	0	0	0	0	0	0	0	0	0	0	0	0	0	0	0	0	0	0	0	0	0	100
*asara*	0	18	0	0	0	0	0	0	0	0	0	0	0	0	0	0	0	0	0	0	0	0	0	0	0	0	0	0	0	0	0	0	0	100
*atimo*	0	0	31	0	0	0	0	0	0	0	0	0	0	0	0	0	0	0	0	0	0	0	0	0	0	0	0	0	0	0	0	0	0	100
*aurosus*	0	0	0	16	0	0	0	0	0	0	0	0	0	0	0	0	0	0	0	0	0	0	0	0	0	0	0	0	0	0	0	0	0	100
*becki*	0	0	0	0	10	0	0	0	0	0	0	0	0	0	0	0	0	0	0	0	0	0	0	0	0	0	0	0	0	0	0	0	1	90.91
*bemaheva*	0	0	0	0	0	22	0	0	0	0	0	0	0	0	0	1	0	0	0	0	0	0	0	0	0	0	0	0	0	0	0	0	0	95.65
*boivini*	0	0	0	0	0	0	49	0	0	0	0	0	0	0	0	0	0	0	0	0	0	0	0	0	0	0	0	0	0	0	0	0	0	100
*bozaka*	0	0	0	0	0	0	0	11	0	0	0	0	0	0	0	0	0	0	0	0	0	0	0	1	0	0	0	0	0	0	0	0	0	91.67
*cemeryi*	0	0	0	0	0	0	0	0	16	0	0	0	0	0	0	0	0	0	0	0	0	0	0	0	0	0	0	0	0	0	0	0	0	100
*cervicalis*	0	0	0	0	0	0	0	0	0	8	0	0	0	0	0	0	0	0	0	0	0	0	0	0	0	0	0	0	0	0	0	0	0	100
*daraina*	0	0	0	0	0	0	0	0	0	0	18	0	0	1	0	0	0	0	0	0	0	0	0	0	0	0	0	0	0	0	0	0	0	94.74
*dufouri*	0	0	0	0	0	0	0	0	0	0	0	19	0	0	0	0	0	0	0	0	0	0	0	0	0	0	0	0	0	0	0	0	0	100
*gibber*	0	0	0	0	0	0	0	0	0	0	0	0	33	0	0	0	0	0	0	0	0	0	0	0	0	0	3	0	0	0	0	0	0	91.67
*gouldi*	0	0	0	0	0	0	0	0	0	0	0	0	0	18	0	0	0	0	0	0	0	0	0	0	0	0	0	0	0	0	0	0	0	100
*hagensii*	0	0	0	0	0	0	0	0	0	0	0	0	1	0	16	0	0	0	0	0	0	0	0	0	0	0	0	0	0	0	0	0	0	94.12
*hova*	0	0	0	0	0	1	0	0	0	0	0	0	0	0	0	26	0	0	0	0	0	0	0	0	0	0	0	0	0	0	0	0	0	96.30
*hovahovoides*	0	0	0	0	0	0	0	0	0	0	0	0	0	0	0	0	55	0	0	0	0	0	0	0	0	0	0	0	0	0	0	0	0	100
*imitator*	0	0	0	0	0	0	0	0	0	0	0	0	0	0	0	0	0	43	0	0	0	0	0	0	0	0	0	0	0	0	0	0	1	97.73
*immaculatus*	0	0	0	0	0	0	0	0	0	0	0	0	0	0	0	0	0	0	11	0	1	0	0	0	0	0	0	0	0	0	0	0	0	91.67
*kelimaso*	0	0	0	0	0	0	0	0	0	0	0	0	0	0	0	0	0	0	0	17	0	0	0	0	0	0	0	0	0	0	0	0	0	100
*liandia*	0	0	0	0	0	0	0	0	0	0	0	0	0	0	0	0	0	0	0	0	57	0	0	0	0	0	0	0	0	0	0	0	0	100
*lokobe*	0	0	0	0	0	0	0	0	0	0	0	0	0	0	0	0	0	0	0	0	0	8	0	0	0	0	0	0	0	0	0	0	0	100
*lubbocki*	0	0	0	0	0	0	0	0	0	0	0	0	0	0	0	0	0	0	0	0	0	0	16	0	0	0	0	0	0	0	0	0	0	100
*mahafaly*	0	0	0	0	0	0	0	0	0	0	0	0	0	0	0	0	0	0	0	0	0	0	0	19	0	0	0	0	0	0	0	0	0	100
*mixtellus*	0	0	0	0	0	0	0	0	0	0	0	0	0	0	0	0	0	0	0	0	0	0	0	0	25	0	0	0	0	0	0	0	0	100
*niavo*	0	0	0	0	0	0	0	0	0	0	0	0	0	0	0	0	0	0	0	0	0	0	0	0	0	8	0	0	0	0	0	0	0	100
*quadrimaculatus*	0	0	0	0	0	0	0	0	0	0	0	0	0	0	0	0	0	0	0	0	0	0	0	0	0	0	69	0	0	1	0	0	0	98.57
*radamae*	0	0	0	0	0	0	0	0	0	0	0	0	0	0	0	0	0	0	0	0	0	0	0	0	0	0	0	24	0	0	0	0	0	100
*roeseli*	0	0	0	0	0	0	0	0	0	0	0	0	0	0	0	0	0	0	0	0	0	0	0	0	0	0	0	0	29	0	0	0	0	100
*rotrae*	0	0	0	0	0	0	0	0	0	0	0	0	0	0	0	0	0	0	0	0	0	0	0	0	0	0	1	0	0	18	0	0	0	94.74
*sambiranoensis*	0	0	0	0	0	0	0	0	0	0	0	0	0	0	0	0	0	0	0	0	0	0	0	0	0	0	0	0	0	0	8	0	0	100
*strangulatus*	0	0	1	0	0	0	0	0	0	0	0	0	0	0	0	0	0	0	0	0	0	0	0	0	0	0	0	0	0	0	0	17	0	94.44
*tapia*	0	0	0	0	0	0	0	0	0	0	0	0	0	0	0	0	0	0	0	0	0	0	0	0	0	0	0	0	0	0	0	0	17	100
Total	10	18	32	16	10	23	49	11	16	8	18	19	34	19	16	27	55	43	11	17	58	8	16	20	25	8	73	24	29	19	8	17	19	98.20

#### Taxonomic synopsis of the subgenus Myrmosaga

*Myrmosaga*Forel 1912: 92 [as subgenus of *Camponotus*]. Type species: *Camponotuskelleri* Forel, 1886b: 186, by subsequent designation of Wheeler 1913: 81, a junior synonym of *Camponotusquadrimaculatus* Forel, 1886a: cii. [As junior synonym of *Mayria* Emery, 1925: 121]. Stat. rev.

*Myrmopytia* Emery, 1920a: 243 [as subgenus of *Camponotus*]. Type species: *Camponotusimitator* Forel, 1891: 209, by monotypy, by original designation. Syn. nov.

##### Morphological diagnosis of the worker caste of Malagasy *Myrmosaga*

As for most *Camponotus* species, minor and major worker castes exist within a colony of the subgenus Myrmosaga. In addition, various worker forms showing continuous morphological variation between these two castes are observed. The following combination of characters can be used to reliably distinguish the two extreme worker castes in the subgenus Myrmosaga from other Malagasy subgenera and species groups of *Camponotus*.

#### Minor worker

1. Head elongate in full-face view, sides either converging or diverging posteriorly towards eye level to approximately parallel to each other and start rounding to posterior margin at ca. posterior 1/3 to posterior 1/5; posterior margin sometimes indistinct from lateral margins.
2. Mandible triangular, apical margin armed with six teeth. In some species, two apical teeth closer to each other than the other teeth.
3. Palp formula: 6,4.
4. Clypeus with median carina, its anteromedian margin mostly broadly convex, sometimes straight (*C.dufouri*, *C.lubbocki*, *C.gibber*, *C.rotrae*, *C.immaculatus*, etc.), projecting into triangular lobe (*C.sambiranoensis*, *C.liandia*); anterior margin rarely excised medially (*C.lokobe*).
5. Antenna with 12 antennomeres, elongate flagellomeres; antennal scape long, generally its distal 1/2 surpassing posterior cephalic margin, either covered or without erect hairs; apical antennomere slightly longer than other flagellomeres;
6. Frontal lobe narrow and partially covering the antennal insertion; frontal carina S-shaped, strongly divergent posteriorly.
7. Compound eye large, most often protruding, its posterior margin usually located at ca. posterior 1/3 to posterior 1/4 of the head.
8. Mesosoma in lateral view, ranging from long and low (*C.dufouri*, *C.lokobe*, *C.atimo*, *C.vano*, etc.) to short and high (*C.cemeryi*); or with pronotum and mesonotum weakly convex, mesonotum and propodeum almost straight (*C.hovahovoides*).
9. Promesonotal suture visible.
10. Pronotum with rounded junction between its dorsum and lateral face; humeral angle rounded.
11. Mesopleuron and propodeal surface together clearly longer than lateral portion of pronotum in lateral view.
12. Metapleural gland absent.
13. Metanotal groove inconspicuous to clearly visible.
14. Procoxa of normal size, maximum width as large as, or smaller than, width of mesopleuron.
15. Middle and hind tibiae with single pectinate spur.
16. Tibia of hind leg either axially rounded or rarely twisted basally.
17. Petiolar node laterally narrow and low.
18. Gaster generally elongate and narrow, anteriorly low and short.
19. Sculpture varying from smooth and shiny through finely and densely imbricate to generally matte.

#### Major worker

Major worker similar to minor worker, but characterized by the following distinctive traits: larger, heart-shaped head; less protruding eye not breaking lateral cephalic margin; more robust mesosoma; stronger mandibles (armed with at least seven teeth and denticles); clypeus with anterolateral angle and straight anterior margin; antennal scape shorter, at most apical 1/3 extending beyond posterior margin of head; metanotum distinctly visible; petiolar node much higher than long (more flattened anteroposteriorly); more erect hairs on promesonotum and the junction of propodeal dorsum and declivity.

#### Comments

Originally Forel (1912) created *Myrmosaga* as a separate subgenus of *Camponotus*. Its synonymy under *Mayria* by Emery (1925) was based on similarity in the form of the clypeus, number of mandibular teeth, and shape of the mesosoma, and the petiole. It also was based on insufficient samples and comparative studies of different species belonging to these two subgenera. In the present study, we revealed many characters (in the Morphological diagnosis above) to distinguish *Myrmosaga* from *Mayria*. Members of *Mayria* are differentiated by the combination of the following characters: the clypeus lacks median carina, its anterolateral corner is rounded; the anterior margin of the pronotum is strongly convex in lateral view, forming a rounded flange and extended laterally to form an obtuse humeral angle; in dorsal view, the pronotal disc is rectangular with distinct lateral margins; the propodeum dorsum is never concave. Thus, *Myrmosaga* is revived from this synonymy here.

Species of the Malagasy *Camponotus* that have been previously placed in the subgenera *Dinomyrmex* and *Myrmoturba* by Forel (1914), in the subgenusTanaemyrmex by Emery (1925), and two species (*C.liandia* and *C.lubbocki*) placed in the subgenus Mayria (Santschi 1914; Rakotonirina and Fisher 2018) are now moved into the subgenus Myrmosaga. This is because our detailed examination of the samples of *Camponotus* from the recent extensive survey of ants in Madagascar revealed that morphological differences between these taxa are due to their ability to adapt to diverse habitats. Similarities among them are the result of convergence by using similar techniques in exploiting similar habitats.

*Camponotusimitator* and the following three species *C.jodina*, *C.karaha*, and *C.longicollis* have morphologically similar minor worker caste and have been grouped together in the subgenus Myrmopytia (Rasoamanana et al. 2017). They are specifically characterized by the elongate mesonotum, which is constricted at midlength, and by the dorsally protruding rounded propodeum. However, *C.imitator* has a medially carinate clypeus with a broad rectangular projection, and a dorsally tapering petiolar node that are considered as some of the strong characteristics of the subgenus Myrmosaga. The other three species have triangular anterior clypeal margin and a lower anterior face and conical petiole. The specific shape of the mesosoma is just a result of the adaptive radiation among these three morphologically similar species across the terrestrial landscapes in Madagascar. Accordingly, *C.imitator* is presently moved to *Myrmosaga* and thus Myrmopytia is synonymized under the same subgenus. The three other species recently described under *Myrmopytia* are moved to a different subgenus (Rasoamanana in prep.).

### ﻿Synoptic list of species of the Malagasy *Myrmosaga*

*aina* sp. nov.

*aro* sp. nov.

*asara* sp. nov.

*atimo* sp. nov.

*aurosus* Roger, 1863

*becki* Santschi, 1923, stat. nov.

*bemaheva* sp. nov.

*boivini* Forel, 1891, stat. rev.

= maculatusst.fairmairei Santschi, 1911a, syn. nov.

*bozaka* sp. nov.

*cemeryi* Özdikmen, 2010, stat. nov.

*cervicalis* Roger, 1863

= *gaullei* Santschi, 1911a, syn. nov.

= *perroti* Forel, 1897, syn. nov.

= *perrotiaeschylus* Forel, 1913, syn. nov.

= *gerberti* Donisthorpe, 1949, syn. nov.

*daraina* Rakotonirina & Fisher, sp. nov.

*dufouri* Forel, 1891

= *dufouriimerinensis* Forel, 1891, syn. nov.

*gibber* Forel, 1891

*gouldi* Forel, 1886a

*hagensii* Forel, 1886a

*harenarum* sp. nov.

*hova* Forel, 1891

= hovavar.obscuratus Emery, 1925, syn. nov.

*hovahovoides* Forel, 1892

= radamaevar.hovoides Dalla Torre, 1893, syn. nov.

*imitator* Forel, 1891

*immaculatus* Forel, 1892

= *quadrimaculatusopacata* Emery, 1925, syn. nov.

*joany* sp. nov.

*karsti* sp. nov.

*kelimaso* sp. nov.

*liandia* Rakotonirina & Fisher, 2018

*lokobe* sp. nov.

*lubbocki* Forel, 1886b

*mahafaly* sp. nov.

*mixtellus* Forel, 1891, stat. nov.

*niavo* sp. nov.

*quadrimaculatus* Forel, 1886a

= *kelleri* Forel, 1886b, syn. nov.

= kellerivar.invalidus Forel, 1897, syn. nov.

= *quadrimaculatussellaris* Emery, 1895, syn. nov.

*radamae* Forel, 1891, stat. rev.

*roeseli* Forel, 1910

= maculatusst.legionarium Santschi, 1911b, syn. nov.

*rotrae* sp. nov.

*sambiranoensis* sp. nov.

*strangulatus* Santschi, 1911a

= *hovamaculatoides* Emery, 1920b, syn. nov.

*tapia* sp. nov.

*tendryi* sp. nov.

*vano* sp. nov.

Most of the species names listed above correspond to different species codes that have been previously used on AntWeb and in publications. These changes are provided in the present study (Table 4).

**Table 4.** List of the species names and their previous corresponding species codes used on AntWeb and in publications.

Species name	Species code
*aina*	MG107
*aro*	MG072
*asara*	MG070
*atimo*	MG062
*becki*	MG071
*bemaheva*	MG064B
*boivini*	MG045, MG049B, MG050
*bozaka*	MG060
*cemeryi*	MG046
*cervicalis*	MG065
*daraina*	MG064
*dufouri*	MG067, MG069
*gibber*	MG014, MG030
*harenarum*	MG105
*hova*	MG056, MG056A, europa_sp1
*hovohovoides*	MG054, MG055
*immaculatus*	MG013
*joany*	MG108
*karsti*	MG106
*kelimaso*	MG056B
*liandia*	MG010, MG012
*lokobe*	MG068
*mahafaly*	MG062B
*mixtellus*	MG053
*niavo*	MG057
*quadrimaculatus*	MG016
*radamae*	MG048
*roeseli*	MG059
*rotrae*	MG015
*sambiranoensis*	MG066
*tapia*	MG061, MG063
*tendryi*	MG052
*vano*	MG125

### ﻿Identification key to minor worker caste of the Malagasy CamponotussubgenusMyrmosaga

**Table:** 

1	With head in full-face view, eyes not breaking lateral cephalic margins (Fig. 2A)	**2**
–	With head in full-face view, eyes breaking lateral cephalic margins (Fig. 2B), if not then lateral cephalic margin diverging posteriorly (Fig. 2C)	**5**
2	Dorsal margin of propodeum entirely straight (Fig. 3A)	**3**
–	Dorsal margin of propodeum with blunt angle at ca. posterior 1/2 (Fig. 3B)	**4**
3	Propodeal dorsum 3 × as long as the declivity; petiolar node with dorsal margin as long as posterior margin (Fig. 4A)	***aina***
–	Propodeal dorsum 2 × as long as the declivity; petiolar node with dorsal margin shorter than posterior margin (Fig. 4B)	***joany***
4	With mesosoma in lateral view, junction of mesonotum and anterior 1/2 of propodeum continuously straight, sloping down to make noticeable angle with posterior 1/2 of propodeum; petiolar node low and long (Fig. 5A)	***karsti***
–	With mesosoma in lateral view, mesonotum and anterior 1/2 of propodeum forming separate convexities; petiolar node ca. as high as long (Fig. 5B)	***harenarum***
5	With head in full-face view, lateral cephalic margins converging posteriorly towards eye level (Fig. 6A)	**6**
–	With head in full-face view, lateral cephalic margin either approximately parallel or diverging posteriorly towards eye level (Fig. 6B, C)	**14**
6	Two apical teeth of mandible normally spaced (Fig. 7A); clypeus with anterolateral corner	**7**
–	Two apical teeth of mandible closely spaced (Fig. 7B); clypeus without anterolateral corner, lateral and anteromedian margin continuously forming broad convexity	**12**
7	With head in full-face view, anteromedian margin of clypeus broadly convex (Fig. 8A)	**8**
–	With head in full-face view, anteromedian margin of clypeus either generally straight (Fig. 8B) or medially excised (Fig. 8C)	**10**
8	With head in full-face view, lateral cephalic margin anterior to eye level without erect hairs (Fig. 9A)	***sambiranoensis***
–	With head in full-face view, lateral cephalic margin anterior to eye level covered with erect hairs (Fig. 9B)	**9**
9	With head in full-face view, level of posterior ocular margin located approximately at posterior 1/4 of the length of head; antennal scape with appressed hairs (Fig. 10A)	***niavo***
–	With head in full-face view, level of posterior ocular margin located at posterior 1/3 of the length of head; antennal scape with suberect hairs (Fig. 10B)	***cervicalis***
10	Anteromedian margin of clypeus noticeably excised medially (Fig. 11A)	***lokobe***
–	Anteromedian margin of clypeus straight (Fig. 11B)	**11**
11	Lateral cephalic margin posterior to eye level covered with erect hairs (Fig. 12A); larger size (CS: 1.97±0.19; 1.66–2.26; ML: 4.23±0.39; 3.56–4.81)	***dufouri***
–	Lateral cephalic margin posterior to eye level without erect hairs (Fig. 12B); smaller size (CS: 1.57±0.01; 1.56–1.58; ML: 3.26±0.01; 3.21–3.31)	***tendryi***
12	Lateral cephalic margin posterior to eye level without erect hairs (Fig. 13A)	***bemaheva***
–	Lateral cephalic margin posterior to eye level covered with erect hairs (Fig. 13B)	**13**
13	Integument of entire body yellowish orange to reddish orange (Fig. 14A)	***daraina***
–	Head and gaster reddish black and mesosoma reddish orange or integument entirely reddish black (Fig. 14B)	***roeseli***
14	In full-face view, lateral margin of head anterior to eye level approximately parallel and covered with erect hairs (Fig. 15A)	**15**
–	In full-face view, lateral margin of head anterior to eye level diverging posteriorly (Fig. 15C), if parallel then lacking erect hairs (Fig. 15B)	**23**
15	With mesosoma in lateral view, mesonotum elongate and constricted at midlength, propodeum broadly convex (Fig. 16A)	***imitator***
–	With mesosoma in lateral view, mesonotum short and lacking constriction at midlength; promesonotum an even convexity; propodeal dorsum almost straight (Fig. 16B)	**12**
16	Two apical teeth of mandible closely spaced (Fig. 17A)	**17**
–	Two apical teeth of mandible normally spaced from each other (Fig. 17B)	**19**
17	Antennal scape covered with erect hairs; lateral cephalic margin posterior to eye level with erect hairs (Fig. 18A); larger in size	***hova***
–	Antennal scape covered with appressed hairs (Fig. 18B); lateral cephalic margin posterior to eye level without erect hairs; smaller in size	**18**
18	In lateral view, mesosoma much higher and short (MPH/ML: 0.34±0.01; 0.31–0.36), propodeal dorsum at most 3 × as long as declivity; petiolar node more or less flattened anteroposteriorly and tapering dorsally (Fig. 19A)	***radamae***
–	In lateral view, mesosoma very low and long (MPH/ML: 0.29±0.02; 0.28–0.31), propodeal dorsum at least four times as long as declivity; petiole much nodelike (Fig. 19B)	***vano***
19	Antennal scape covered with appressed hairs (Fig. 20A)	***mahafaly***
–	Antennal scape covered with erect to suberect hairs (Fig. 20B)	**20**
20	Mesosoma generally short and high, its dorsal outline strongly convex, propodeal dorsum < 2 × height of declivity surface (Fig. 21A)	***cemeryi***
–	Mesosoma low and long, its dorsal outline more or less straight or not strongly convex, propodeal dorsum 2 × or more as long as height of declivity surface (Fig. 21B)	**21**
21	In lateral view, dorsum of mesosoma from mid-mesonotum to posterodorsal corner of propodeum approximately straight, propodeal dorsum ca. 3 × as long as height of declivity surface (Fig. 22A)	***hovahovoides***
–	In lateral view, posterior 1/2 of mesonotum to posterodorsal corner of propodeum slightly convex, propodeal dorsum ca. 2 × as long as height of declivity surface (Fig. 22B)	**22**
22	Antennal scape covered with suberect hairs inclined at ca. 30° (Fig. 23A); larger species (CS: 1.59±0.10; 1.36–1.75; ML: 3.10±0.16; 2.76–3.45)	***mixtellus***
–	Antennal scape covered with suberect hairs inclined at ca. 45° (Fig. 23B); smaller species (CS: 1.15±0.06; 1.00–1.27; ML: 2.11±0.10; 1.87–2.28)	***boivini***
23	With head in full-face view, lateral margins of head anterior to eye level parallel to each other (Fig. 24A)	**24**
–	With head in full-face view, lateral margins of head anterior to eye level diverging posteriorly (Fig. 24B)	**31**
24	In full-face view, clypeus with distinctly visible anterolateral corner (Fig. 25A)	**25**
–	In full-face view, clypeus without visible anterolateral corner, lateral and anteromedian clypeal margin continuously forming broad convexity (Fig. 25B)	**30**
25	Dark body color, or posterior portion of mesosoma pale brown; in lateral view, petiolar node scalelike or more or less compressed anteroposteriorly (Fig. 26A)	**26**
–	Pale yellow to orange body color; in lateral view, petiole nodelike and not compressed anteroposteriorly (Fig. 26B)	**28**
26	With head in full-face view, anteromedian clypeal margin truncate; erect hairs present on lateral cephalic margin behind level of posterior ocular margin (Fig. 27A)	***becki***
–	With head in full-face view, anteromedian clypeal margin broadly convex or triangular; erect hairs absent on lateral cephalic margin behind level of posterior ocular margin (Fig. 27B)	**27**
27	In lateral view, mesosoma low and long, propodeal dorsum ca. 3 × as long as declivity, their junction rounded; petiolar node with dorsal margin inclined posteriorly; integument matte (Fig. 28A)	***asara***
–	In lateral view, mesosoma short and high, length of propodeal dorsum < 2 × height of declivity, their junction angulate; petiolar node scalelike; integument shining (Fig. 28B)	***bozaka***
28	With head in oblique profile, three pairs of erect hairs arranged successively from level of anterior ocular margin towards posterior cephalic margin	***strangulatus***
–	With head in oblique profile, four or more pairs of erect hairs arranged successively from level of anterior ocular margin towards posterior cephalic margin	**29**
29	With mesosoma in profile, junction of propodeal dorsum and declivity surface rounded (Fig. 30A)	***atimo***
–	With mesosoma in profile, junction of propodeal dorsum and declivity surface broadly angulate (Fig. 30B)	***tapia***
30	In full-face view, posterior portion of head extending into broadly short neck (Fig. 31A); in profile, entire propodeal dorsum more or less straight and separated from declivity surface by broad angle	***gouldi***
–	In full-face view, posterior portion of head normally rounded, not extending into a short neck (Fig. 31B); in profile, propodeal dorsum immediately posterior to metanotal groove convex, then concave medially and rounding to declivity surface	***aro***
31	With head in full-face view, lateral margin of head anterior to eye level with erect hairs (Fig. 32A); head and gaster black, mesosoma reddish orange to brown	**32**
–	With head in full-face view, lateral margin of head anterior to eye level without erect hairs (Fig. 32B); body color uniquely black, brown to yellowish orange	**33**
32	With head in full-face view, anterior clypeal margin broadly triangular (Fig. 33A); mesosoma in profile short and high; gastral tergites covered with abundant pubescence	***aurosus***
–	With head in full-face view, anterior clypeal margin truncate (Fig. 33B); mesosoma in profile low and long; gastral tergites without abundant pubescence	***hagensii***
33	With head in full-face view, anterior clypeal margin broadly triangular (Fig. 34A)	***liandia***
–	With head in full-face view, anterior clypeal margin truncate (Fig. 34B)	**34**
34	No white spot on dorsum of second and third abdominal tergites (Fig. 35A)	**35**
–	One pair of white spots on third abdominal tergites (Fig. 35B)or two pairs of white spots present on second and third abdominal tergites (Fig. 35C)	**37**
35	With mesosoma in lateral view, propodeal dorsum broadly concave (Fig. 36A)	***immaculatus***
–	With mesosoma in lateral view, propodeal dorsum approximately straight (Fig. 36B)	**36**
36	Eye small (EL/CL: 0.19±0.01; 0.16–0.21) (Fig. 37A); posterior margin of head approximately straight	***kelimaso***
–	Eye large (EL/CL: 0.24±0.01; 0.23–0.27) (Fig. 37B); posterior margin of head broadly convex	***lubbocki***
37	Pronotum, mesonotum, and propodeum forming separate convexities, metanotal groove depressed; propodeum at lower level than promesonotum; petiolar node with dorsal face rounding to anterior and posterior faces (Fig. 38A)	***gibber***
–	Pronotum, mesonotum, and propodeum not forming separate convexities, metanotal groove not depressed; propodeum immediately in junction with promesonotum; petiolar node with dorsal face joining posterior face into an angle and rounding to anterior face (Fig. 38B)	**38**
38	Propodeal dorsum straight (Fig. 39A)	***rotrae***
–	Propodeal dorsum concave (Fig. 39B)	***quadrimaculatus***

**Figure 2.**
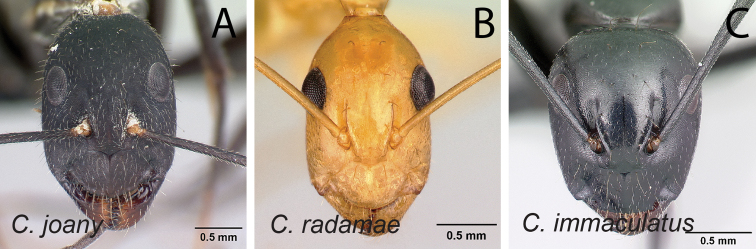
Head in full-face view **A***C.joany* (CASENT0408908) **B***C.radamae* (CASENT0066777) **C***C.immaculatus* (CASENT0179441).

**Figure 3.**
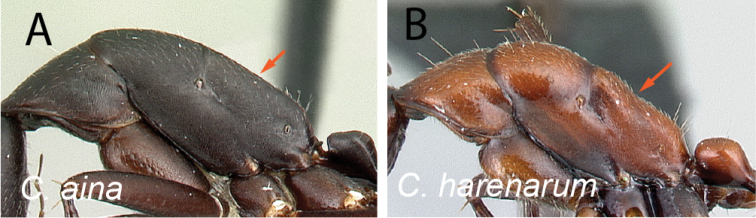
Mesosoma in lateral view **A***C.aina* (CASENT0217291) **B***C.harenarum* (CASENT0499207).

**Figure 4.**
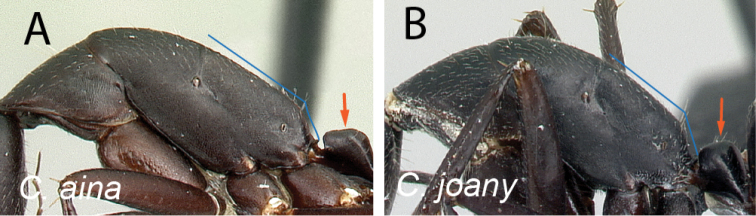
Mesosoma and petiolar node in lateral view **A***C.aina* (CASENT0217291) **B***C.joany* (CASENT0408908).

**Figure 5.**
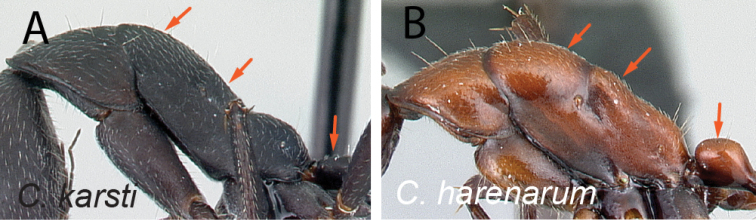
Mesosoma and petiolar node in lateral view **A***C.karsti* (CASENT0217292) **B***C.harenarum* (CASENT0499207).

**Figure 6.**
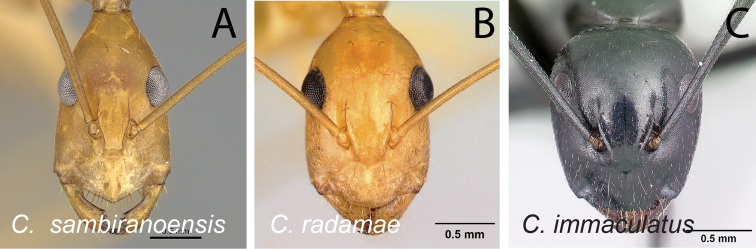
Head in full-face view **A***C.sambiranoensis* (CASENT0231845) **B***C.radamae* (CASENT0066777) **C***C.immaculatus* (CASENT0179441).

**Figure 7.**
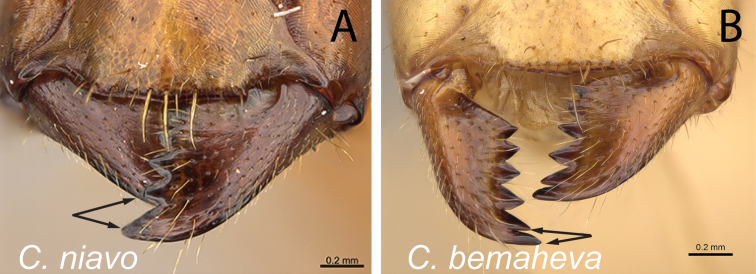
Mandibles in frontal view **A***C.niavo* (CASENT0300086) **B***C.bemaheva* (CASENT0153778).

**Figure 8.**
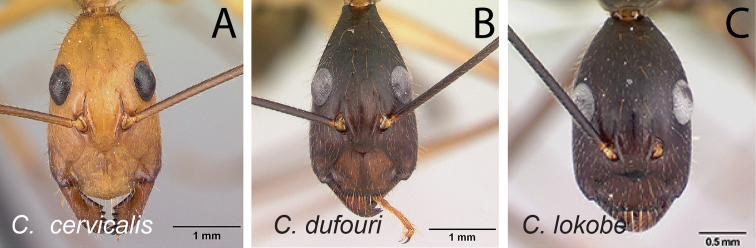
Head in full-face view **A***C.cervicalis* (CASENT0217303) **B***C.dufouri* (CASENT0487727) **C***C.lokobe* (CASENT0436584).

**Figure 9.**
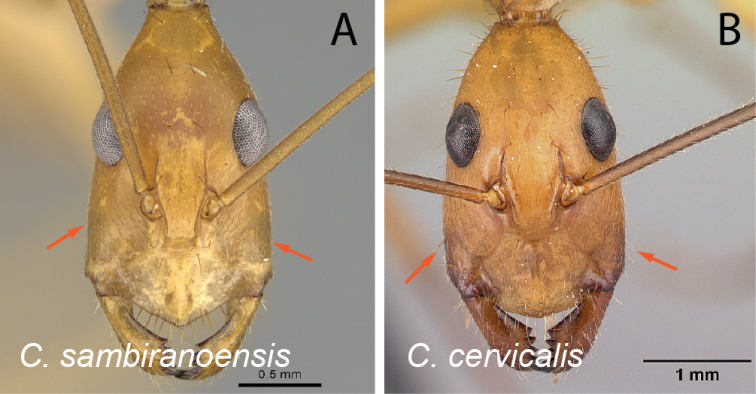
Head in full-face view **A***C.sambiranoensis* (CASENT0231845) **B***C.cervicalis* (CASENT0217303).

**Figure 10.**
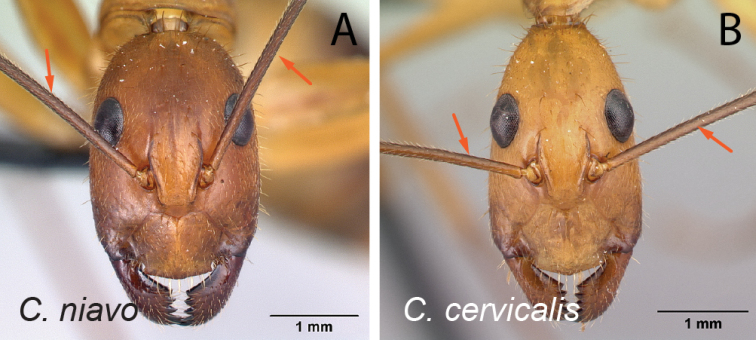
Head in full-face view **A***C.niavo* (CASENT0134004) **B***C.cervicalis* (CASENT0217303).

**Figure 11.**
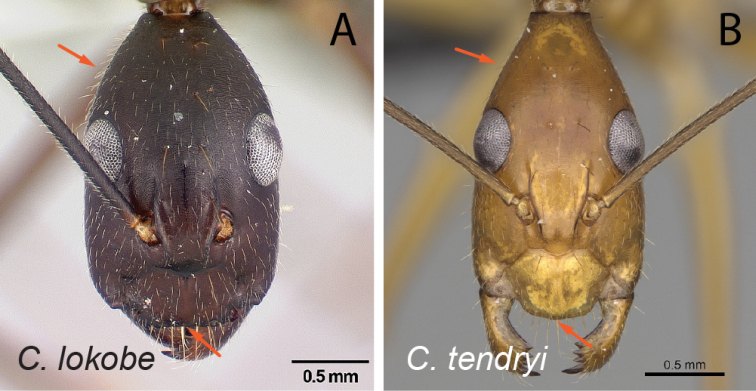
Head in full-face view **A***C.lokobe* (CASENT0436584) **B***C.tendryi* (CASENT0145284).

**Figure 12.**
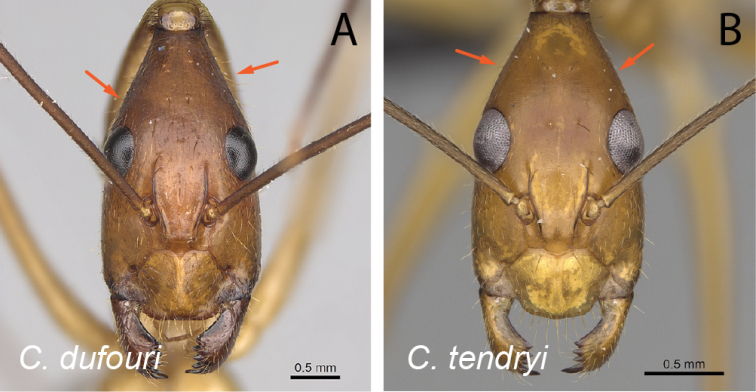
Head in full-face view **A***C.dufouri* (CASENT0274127) **B***C.tendryi* (CASENT0145284).

**Figure 13.**
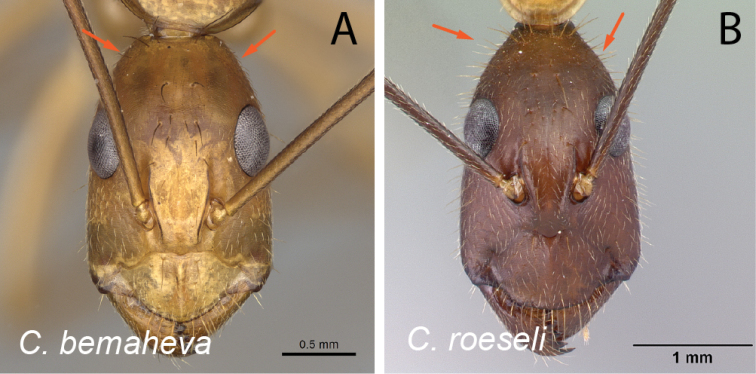
Head in full-face view **A***C.bemaheva* (CASENT0154158) **B***C.roeseli* (CASENT0492473).

**Figure 14.**
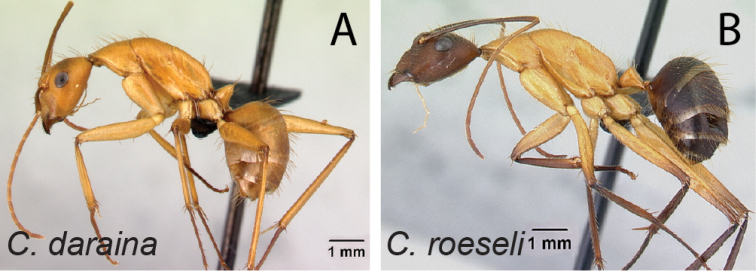
Body in lateral view **A***C.daraina* (CASENT0077433) **B***C.roeseli* (CASENT0492473).

**Figure 15.**
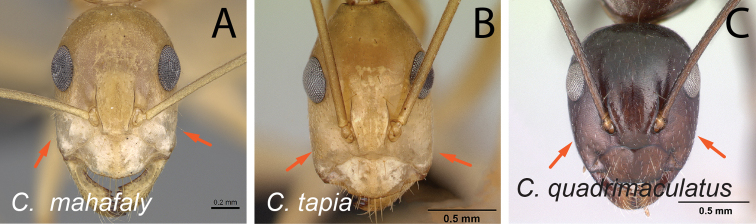
Head in full-face view **A***C.mahafaly* (CASENT0115287) **B***C.tapia* (CASENT0493939) **C***C.quadrimaculatus* (CASENT0096044).

**Figure 16.**
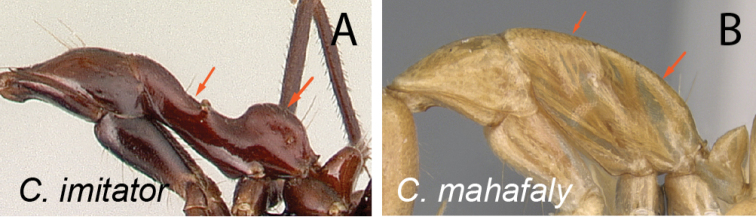
Mesosoma in lateral view **A***C.imitator* (CASENT0452849) **B***C.mahafaly* (CASENT0115287).

**Figure 17.**
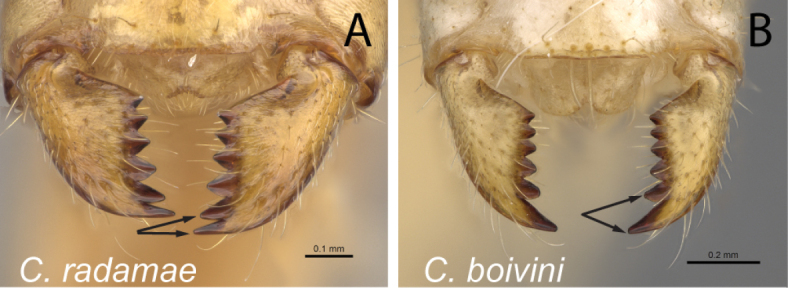
Mandibles in frontal view **A***C.radamae* (CASENT0228250) **B***C.boivini* (CASENT0477264).

**Figure 18.**
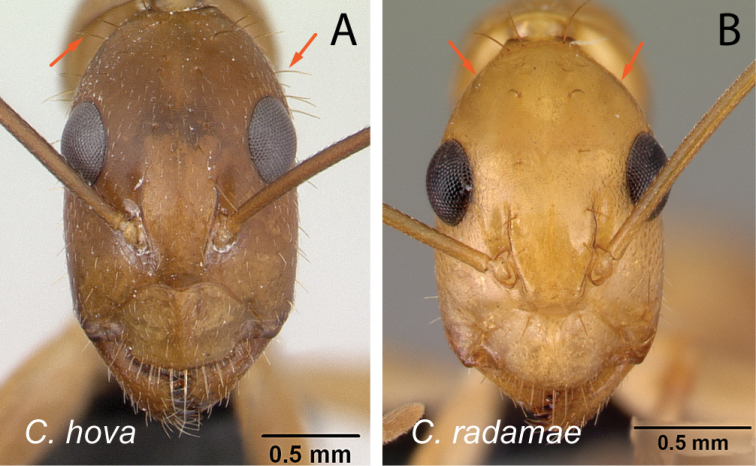
Head in full-face view **A***C.hova* (CASENT0055710) **B***C.radamae* (CASENT0217307).

**Figure 19.**
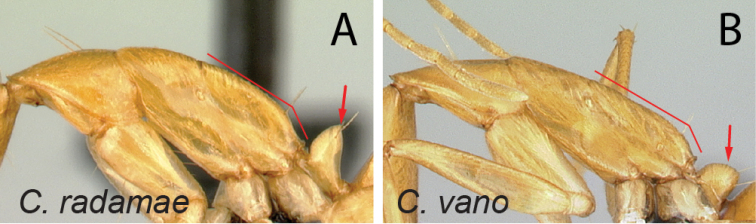
Mesosoma and petiolar node in lateral view **A***C.radamae* (CASENT066777) **B***C.vano* (CASENT0217316).

**Figure 20.**
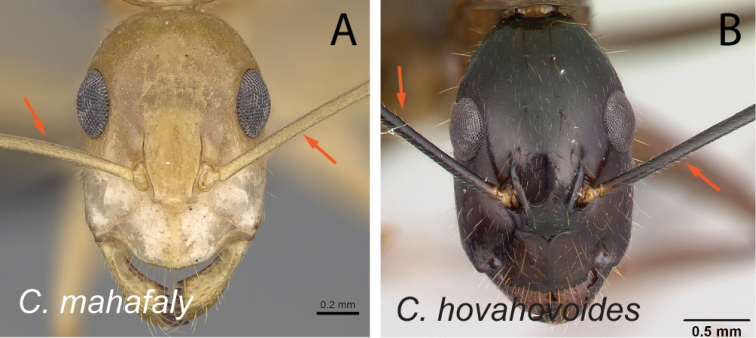
Head in full-face view **A***C.mahafaly* (CASENT0115287) **B***C.hovahovoides* (CASENT0487242).

**Figure 21.**
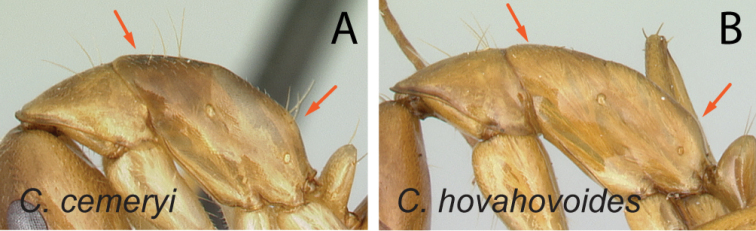
Mesosoma and petiolar node in lateral view **A***C.cemeryi* (CASENT0217306) **B***C.hovahovoides* (CASENT0496908).

**Figure 22.**
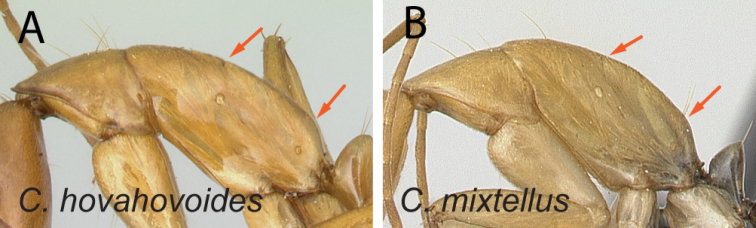
Mesosoma in lateral view **A***C.hovahovoides* (CASENT0496908) **B***C.mixtellus* (CASENT0132606).

**Figure 23.**
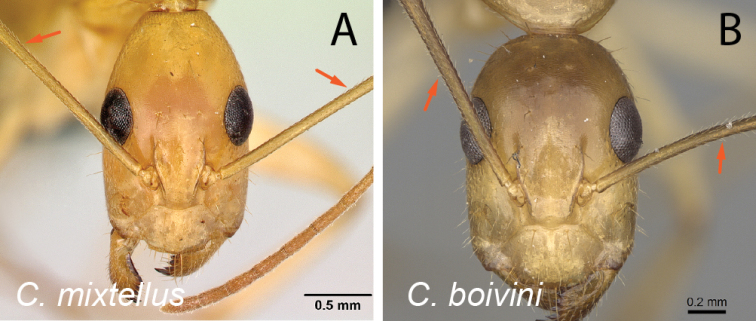
Head in full-face view **A***C.mixtellus* (CASENT0076675) **B***C.boivini* (CASENT0168345).

**Figure 24.**
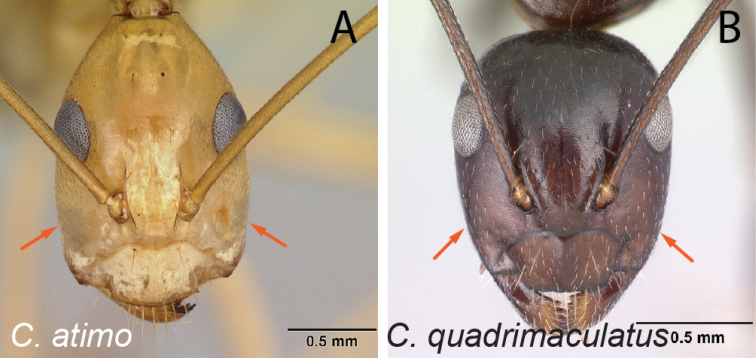
Head in full-face view **A***C.atimo* (CASENT0454042) **B***C.quadrimaculatus* (CASENT0096044).

**Figure 25.**
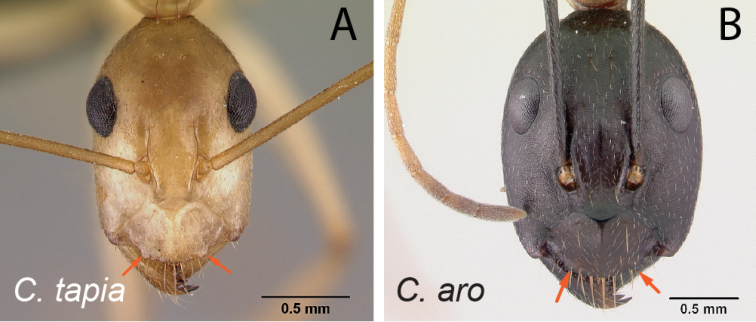
Head in full-face view **A***C.tapia* (CASENT0217310) **B***C.aro* (CASENT0489917).

**Figure 26.**
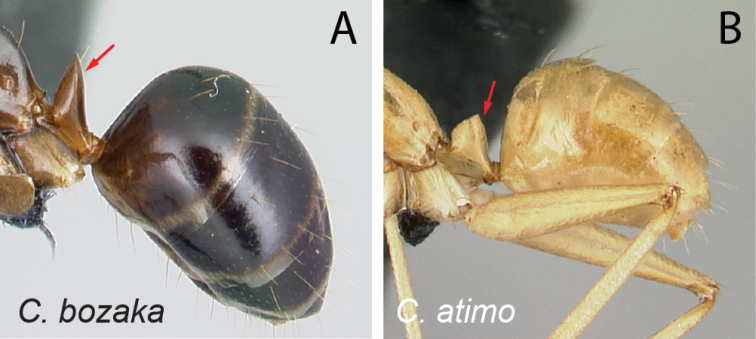
Posterior portion of mesosoma, petiolar node, and gaster in lateral view **A***C.bozaka* (CASENT0217309) **B***C.atimo* (CASENT0454042).

**Figure 27.**
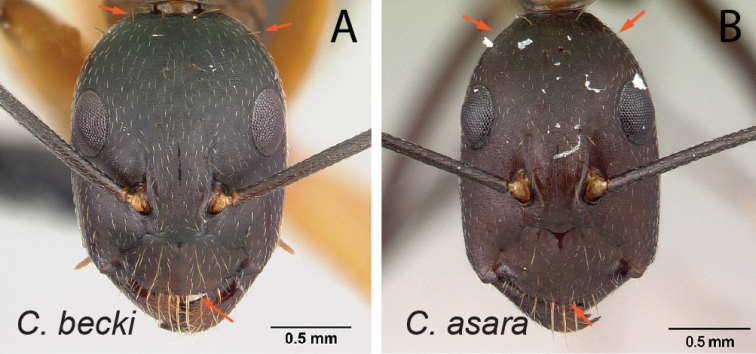
Head in full-face view **A***C.becki* (CASENT0071380) **B***C.asara* (CASENT0493252).

**Figure 28.**
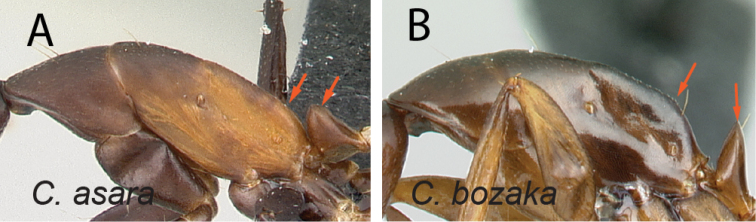
Mesosoma and petiolar node in lateral view **A***C.asara* (CASENT0493252) **B***C.bozaka* (CASENT0217309).

**Figure 29.**
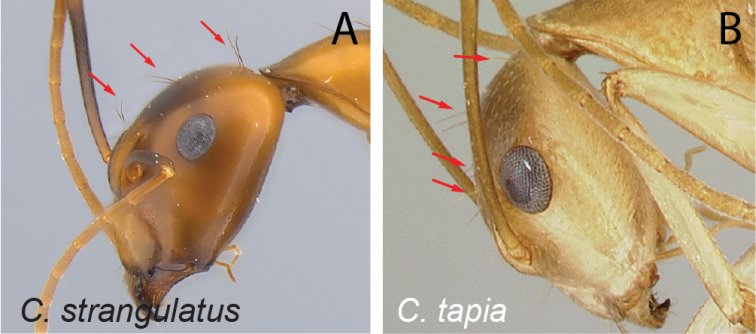
Head in lateral view **A***C.strangulatus* (CASENT0136581) **B***C.tapia* (CASENT0493939).

**Figure 30.**
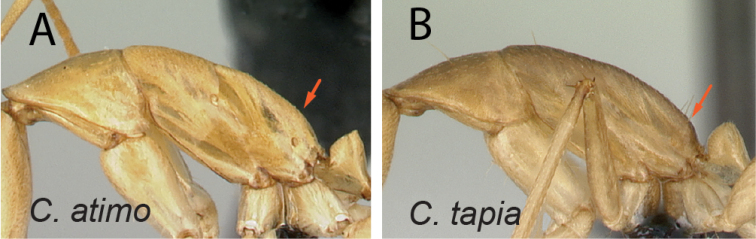
Mesosoma in lateral view **A***C.atimo* (CASENT0454042) **B***C.tapia* (CASENT0217310).

**Figure 31.**
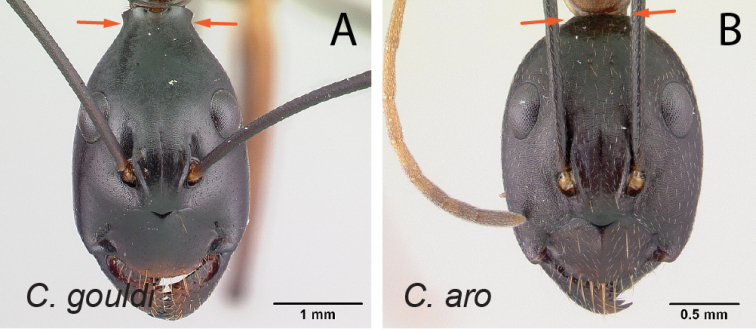
Head in full-face view **A***C.gouldi* (CASENT0121617) **B***C.aro* (CASENT0489917).

**Figure 32.**
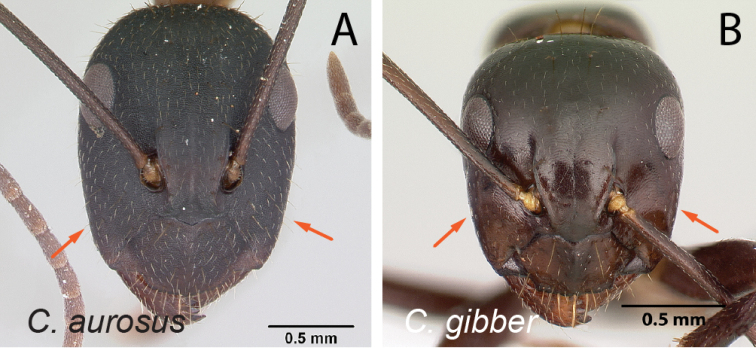
Head in full-face view **A***C.aurosus* (CASENT0064815) **B***C.gibber* (CASENT0188619).

**Figure 33.**
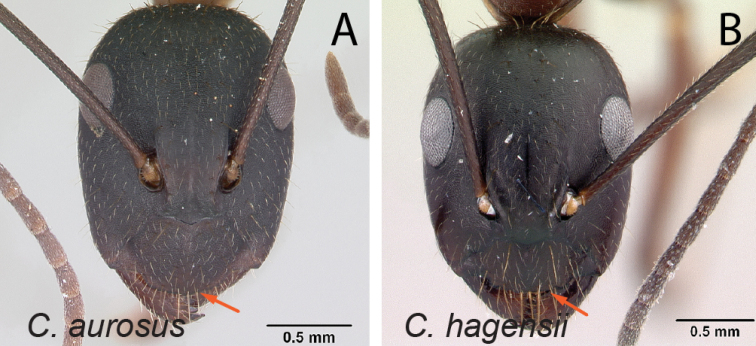
Head in full-face view **A***C.aurosus* (CASENT0064815) **B***C.hagensii* (CASENT0191568).

**Figure 34.**
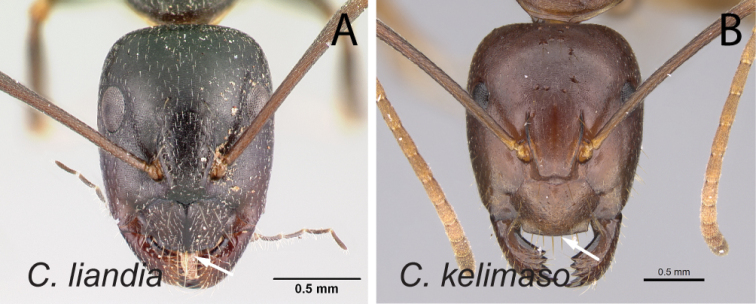
Head in full-face view **A***C.liandia* (CASENT0491145) **B***C.kelimaso* (CASENT0487718).

**Figure 35.**
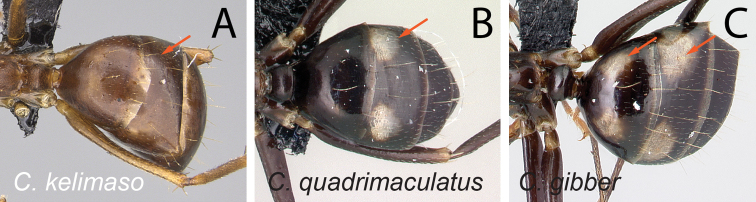
Second and third abdominal tergites in dorsal view **A***C.kelimaso* (CASENT0487718) **B***C.quadrimaculatus* (CASENT0096044) **C***C.gibber* (CASENT0491497).

**Figure 36.**
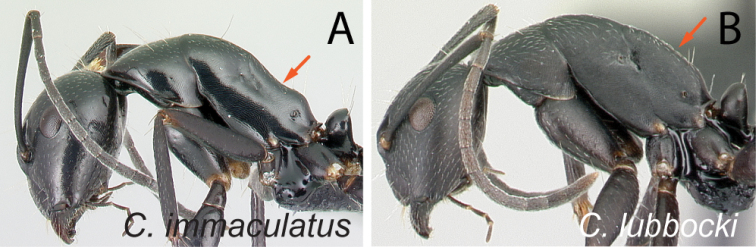
Head, mesosoma and petiolar node in lateral view **A***C.immaculatus* (CASENT0179441) **B***C.lubbocki* (CASENT0486998).

**Figure 37.**
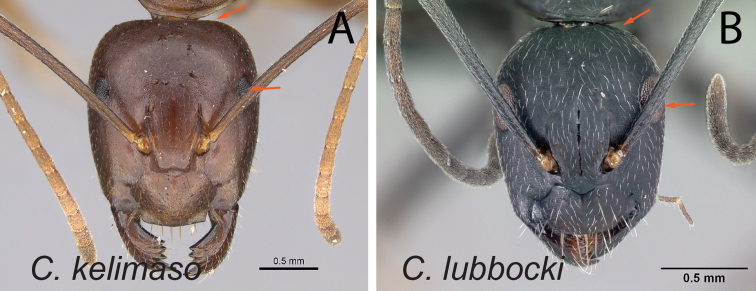
Head in full-face view **A***C.kelimaso* (CASENT0487718) **B***C.lubbocki* (CASENT0486998).

**Figure 38.**
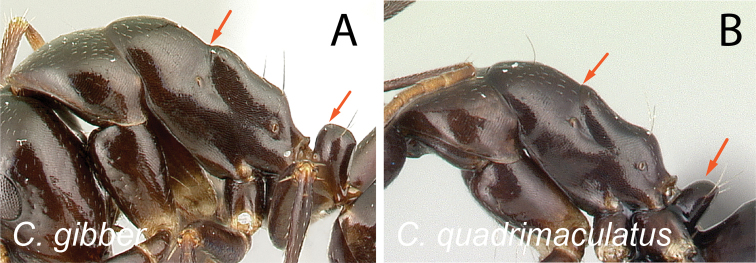
Mesosoma and petiolar node in lateral view **A***C.gibber* (CASENT0188619) **B***C.quadrimaculatus* (CASENT0096044).

**Figure 39.**
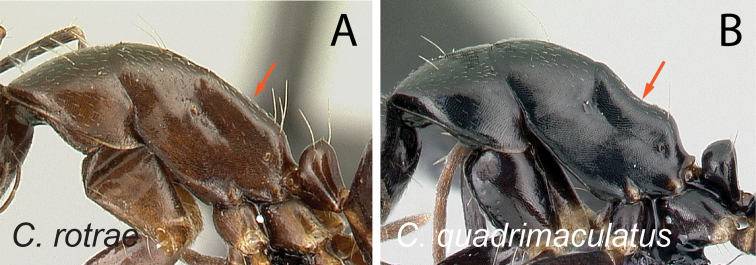
Mesosoma in lateral view **A***C.rotrae* (CASENT0485625) **B***C.quadrimaculatus* (CASENT0121629).

### ﻿Species accounts

#### ﻿ Camponotus aina sp. nov.

Taxon classificationAnimaliaHymenopteraFormicidae

87EE7D7E-8095-5115-8E11-3FC4F95B0881

http://zoobank.org/B622AD1C-1005-44D3-97F5-05E22C8D291B

Figs 3A , 4A , 40

##### Holotype worker.

**Madagascar**: Province **Antsiranana**: RS Ankarana, 7 km SSE Matsaborimanga, -12.91667, 49.1, 150 m, tropical dry forest, on low vegetation, 28 Nov 1990 (P.S. Ward) collection code: PSW11019, specimen code: CASENT0217291 (CAS).

##### Paratype.

1 minor worker with same data as holotype but specimen coded as: CASENT0217290 (CAS).

##### Additional material examined.

**Madagascar: Antsiranana**: RS Ankarana, 7 km SSE Matsaborimanga, -12.91667, 49.1, 150 m, tropical dry forest (P.S. Ward) (CAS).

##### Diagnosis.

With head in full-face view, eye not breaking lateral cephalic margin; mesonotum short and lacking constriction; propodeal dorsum approximately straight, 3 × as long as the declivity; dorsal margin of petiole as long as posterior margin.

##### Description.

**Minor worker.** With head in full-face view, lateral margins anterior to eye level approximately parallel, rounding evenly towards slightly concave posterior margin; eye convex and large (EL/CS: 0.24±0.02; 0.22–0.26), not breaking lateral cephalic margin, location of its posterior margin at posterior 1/4 of head (PoOc/CL: 0.26±0.00; 0.26–0.26); frontal carinae parallel, widely opened posteriorly (FR/CS: 0.28±0.01; 0.27–0.29); clypeus with blunt or poorly defined anterolateral angle, anteromedian margin broadly convex; two apical teeth of mandible normally spaced; antennal scape relatively long (SL/CS: 1.64±0.02; 1.62–1.66). Promesonotum markedly convex, posterior region of mesonotum and propodeal dorsum approximately straight, with metanotal groove visible as suture; propodeal dorsum 3 × as long as declivity, their junction forming blunt angle. Anterior margin of petiolar node ca. 1/2 height of posterior margin and length of dorsal margin, separated from them by an angle and a convexity, respectively.

First and second gastral tergites without a pair of white spots; erect hairs on lateral margin of head present; near posterior margin of head with two elongate, erect hairs; antennal scape covered with erect hairs and lacking appressed hairs; junction of propodeal dorsum and declivity with two pairs of erect hairs.

**Major worker.** Unknown.

##### Distribution and biology.

**Figure 40.**
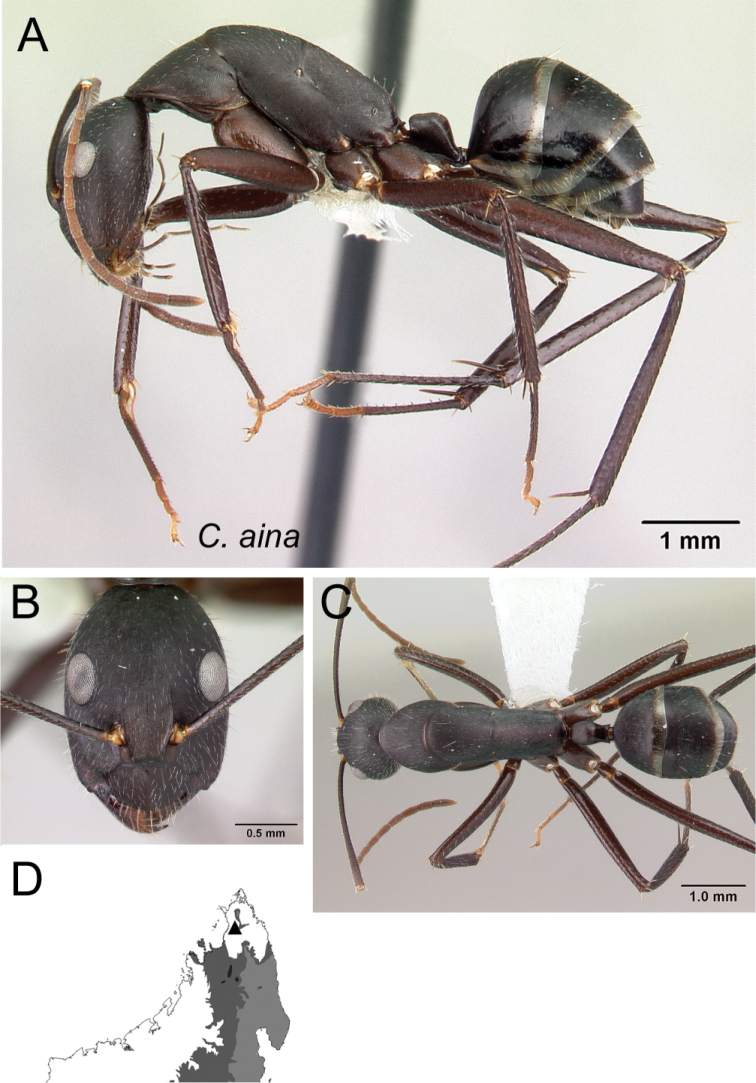
*Camponotusaina***A** lateral view **B** head in full-face view **C** dorsal view of holotype minor worker CASENT0217291**D** distribution map.

*Camponotusaina* is endemic to Madagascar, its distribution restricted to the dry forest and Tsingy of the RS Ankarana in the north of the island (Fig. 40D). Its nest site is unknown, but workers have been found foraging on lower vegetation.

##### Discussion.

*Camponotusaina* looks similar to *C.joany* in that it has a straight propodeal dorsum, but in the latter species the propodeal dorsum is 2 × as long as the declivity and the dorsal margin of the petiolar node is shorter than the posterior margin.

##### Etymology.

The species name *aina* is a non-Latin singular noun and is being used as a noun in apposition.

#### ﻿ Camponotus aro sp. nov.

Taxon classificationAnimaliaHymenopteraFormicidae

D937B665-284D-52DC-B4C9-EBA8B74FDF9F

http://zoobank.org/3C81BF3C-27C7-476A-889D-F2DD66EE7E55

Figs 25B , 31B , 41

##### Holotype worker.

**Madagascar**: Province **Mahajanga**: PN de Namoroka, 17.8 km 329° WNW Vilanandro, -16.37667, 45.32667, 100 m, dry forest, under stone, 08 Nov 2002 (Fisher, Griswold et al.) collection code: BLF06542, specimen code: CASENT0489917 (CAS).

##### Paratypes.

2 minor workers and 1 major worker with same data as holotype but with specimen codes: CASENT0837586, CASENT0837587, CASENT0489918 (NHMUK, MHNG, CAS).

##### Additional material examined.

**Madagascar: Mahajanga**: PN Namoroka, 16.9 km 317° NW Vilanandro, -16.40667, 45.31, 100 m, tropical dry forest (Fisher, Griswold et al.) (CAS); PN Namoroka, 17.8 km 329° WNW Vilanandro, -16.37667, 45.32667, 100 m, tropical dry forest (Fisher, Griswold et al.) (CAS).

##### Diagnosis.

With head in full-face view, lateral margins of head anterior to eye level parallel, lacking erect hairs, lateral cephalic margin rounding to posterior margin, anteromedian clypeal margin continuously forming broad convexity; propodeal dorsum immediately posterior to metanotal groove convex, then concave medially and rounding to declivity surface.

##### Description.

**Minor worker.** In full-face view, head widest at midlength; lateral margins posterior to level of eye, rounding evenly to posterior margin; eye protruding and large (EL/CS: 0.25±0.01; 0.23–0.26), not breaking lateral cephalic margin; level of its posterior border located at ca. posterior 1/3 of head (PoOc/CL: 0.28±0.01; 0.27–0.30); frontal carinae posteriorly parallel (FR/CS: 0.25±0.01; 0.24–0.27); clypeus without anterolateral angle, its anteromedian margin broadly convex; mandible with six teeth, its two apical teeth distantly spaced; antennal scape relatively long (SL/CS: 1.59±0.08; 1.46–1.71). Mesosoma long and low (MPH/ML: 0.28±0.01; 0.26–0.30), promesonotum weakly convex, mesonotum with posterior portion flat immediately anterior to a weakly visible metanotal groove; propodeal dorsum anteriorly convex, with feeble concavity medially, dorsal margin of propodeum rounding to declivity; propodeal dorsum 2 × as long as declivity. Petiolar node nodiform; with dorsal margin inclined posteriorly and forming a blunt angle to anterior face; anterior face of petiolar node 1/2 height of posterior face; femur of hind leg rounded axially and twisted basally.

First and second gastral tergites with a pair of white spots; erect hairs on lateral margin of head absent; two erect hairs present near posterior margin of head; antennal scape only covered with appressed hairs; pronotum with a pair of erect hairs; posterodorsal angle of propodeum with a pair of erect hairs.

**Major worker.** Differing from minor worker in the following characters: enlarged head (CS: 3.10±0.18; 2.82–3.29; CWb/CL: 0.92±0.01; 0.90–0.94) with markedly concave posterior margin; apical 1/3 of antennal scape extending beyond posterior cephalic margin; robust mesosoma with propodeal dorsum slightly concave and its length ca. 2 × height of declivity; petiole tapering dorsally.

##### Distribution and biology.

**Figure 41.**
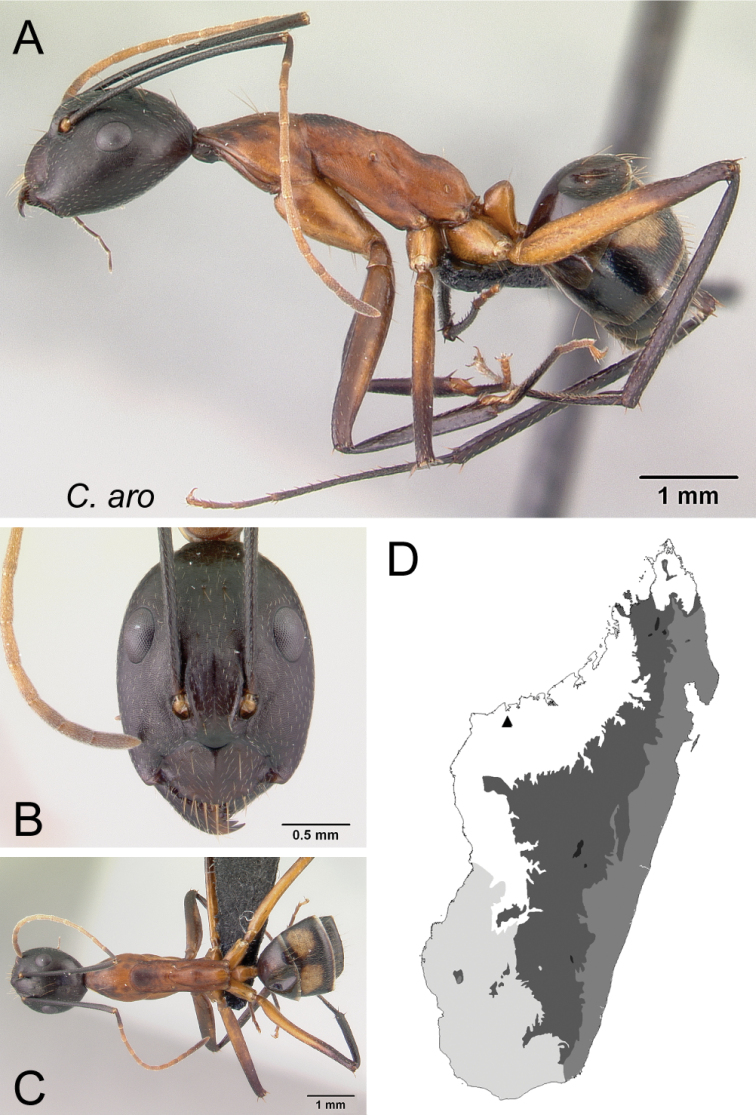
*Camponotusaro***A** lateral view **B** head in full-face view **C** dorsal view of holotype minor worker CASENT0489917**D** distribution map.

Geographically restricted to the PN Namoroka in the western dry forest of Madagascar (Fig. 41D), *C.aro* has been found nesting under stones while workers forage on the ground or through leaf litter and on lower vegetation.

##### Discussion.

*Camponotusaro* is morphologically similar to *C.gouldi* in that both species are characterized by a broadly convex anteromedian margin of clypeus, but the latter species is diagnosed as having a head that extends posteriorly into a broad, short neck and its propodeal dorsum is straight.

The definition for *C.aro* is supported by the congruence between the results of traditional qualitative morphology and the NC-clustering technique. The classification of the samples for the species is at 100% success.

##### Etymology.

The species name *aro* is a non-Latin singular noun used in apposition.

#### ﻿ Camponotus asara sp. nov.

Taxon classificationAnimaliaHymenopteraFormicidae

A22ED62B-F1D9-5FA9-8587-130050582AEB

http://zoobank.org/5C3637B8-D025-44D1-B91D-E49FA3D3CEBC

Figs 27B , 28A , 42

##### Holotype worker.

**Madagascar**: Province **Toliara**: PN Zombitse, 19.8 km 84° E Sakaraha, -22.84333, 44.71, 770 m, tropical dry forest, ex rotten log, 05–09 Feb 2003 (Fisher, Griswold et al.) collection code: BLF07523, specimen code: CASENT0493252 (CAS).

##### Paratypes.

3 workers, same data as holotype but with the following specimen codes: CASENT0493251, CASENT0837576, CASENT0837577 (NHMUK, CAS).

##### Additional material examined.

**Madagascar: Toliara**: 15 km E Sakaraha, -22.9, 44.68333, 760 m, tropical dry forest (P.S. Ward) (CAS); PN Zombitse, 19.8 km 84° E Sakaraha, -22.84333, 44.71, 770 m, tropical dry forest (Fisher, Griswold et al.) (CAS); RS Ambohijanahary, Forêt d’Ankazotsihitafototra, 34.6 km 314° NW Ambaravaranala, -18.26, 45.41833, 1100 m, montane rainforest (Fisher, Griswold et al.) (CAS); RS Ambohijanahary, Forêt d’Ankazotsihitafototra, 35.2 km 312° NW Ambaravaranala, -18.26667, 45.40667, 1050 m, montane rainforest (Fisher, Griswold et al.) (CAS); Southern Isoky-Vohimena Forest, -22.68333, 44.83333, 730 m (Sylvain) (CAS); Vohibasia Forest, 59 km NE Sakaraha, -22.46667, 44.85, 780 m (Sylvain) (CAS); PN Zombitse, 19.8 km 84° E Sakaraha,-22.84333, 44.71, 770 m, tropical dry forest (Fisher, Griswold et al.) (CAS); near ANGAP office, PN Zombitse, -22.8865, 44.69217, 840 m, deciduous spiny forest (R. Harin’Hala) (CAS); near road, PN Zombitse, -22.8405, 44.73117, 825 m, spiny deciduous forest (R. Harin’Hala) (CAS).

##### Diagnosis.

With head in full-face view, lateral margins of head anterior to eye level, parallel, and lacking erect hairs; lateral cephalic margin rounding to posterior margin; anteromedian clypeal margin continuously forming broad convexity; propodeal dorsum more or less straight and separated from declivity surface by broad angle.

##### Description.

**Minor worker.** In full-face view, head sides anterior to level of eye parallel, converging progressively to posterior margin behind eye level; eyes protruding and large (EL/CL: 0.26±0.01; 0.23–0.28), breaking lateral cephalic margin, level of its posterior border approximately located at posterior 1/4 of head (PoOc/CS: 0.26±0.01; 0.24–0.28); frontal carinae posteriorly parallel (FR/CS: 0.26±0.01; 0.23–0.27); clypeus without well-defined anterolateral angle, its anteromedian margin broadly convex; mandible with six teeth, the two apical teeth distantly spaced; antennal scape relatively long (SL/CS: 1.57±0.08; 1.38–1.68). Promesonotum weakly convex, mesopropodeum almost flat; mesonotum flat immediately anterior to weakly visible metanotal groove; propodeal dorsum approximately straight, rounding progressively towards declivity; propodeal declivity ca. 1/3 length of dorsum. Petiolar node flattened anteroposteriorly or short and high, tapering dorsally; femur of hind leg flattened laterally and twisted near base.

First and second gastral tergites with a pair of white spots; erect hairs lacking on lateral margin of head; posterior margin of head with a pair of erect hairs; antennal scape only covered with appressed hairs; pronotum with a pair of erect hairs; posterodorsal angle of propodeum without erect hairs.

**Major worker.** With characteristics of minor worker, except for the following characters: enlarged head (CS: 3.22±0.15; 3.03–3.39; CWb/CL: 0.93±0.01; 0.92–0.93) with noticeable medial excision on posterior margin; straight anterior clypeal margin; apical 1/4 of antennal scape surpassing posterior cephalic margin; propodeum dorsum and declivity the same length.

##### Distribution and biology.

**Figure 42.**
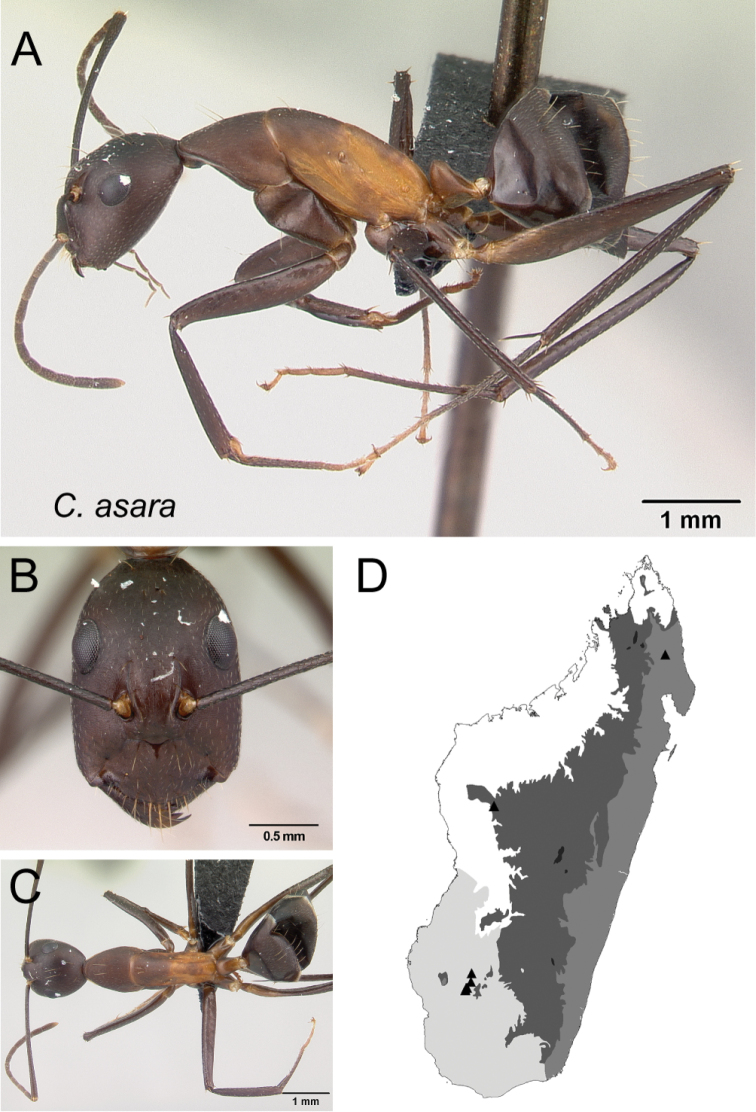
*Camponotusasara***A** lateral view **B** head in full-face view **C** dorsal view of holotype minor worker CASENT0493252**D** distribution map.

*Camponotusasara* is endemic to Madagascar and geographically restricted to the dry forest of the PN Zombitse in the southern high plateau of the island and the relict montane rainforest of the PN Ambohijanahary (Fig. 42D). Colony nests can be in rotten logs and in rot pockets above the ground. Workers have been found foraging in leaf litter and on the ground.

##### Discussion.

*Camponotusasara* is morphologically similar to *C.bozaka* and *C.becki* in that its petiolar node is more or less compressed anteroposteriorly in lateral view and its body color is black to dark brown, or the posterior portion of the mesosoma is pale brown to yellow. However, *C.bozaka* has a scalelike petiolar node, short and high mesosoma, and propodeal dorsum that joins the declivity in a blunt angle with a length < 2 × height of the declivity, the junction being angulate. Regarding *C.becki*, its anteromedian clypeal margin is truncate and a few erect hairs are present on the lateral margin of the head behind the level of the posterior ocular margin.

The cluster of the samples for *C.asara* in the NC-clustering dendrogram is supported by LDA with a classification success of 100%.

##### Etymology.

The species name *asara* is a non-Latin singular noun used in apposition.

#### ﻿ Camponotus atimo sp. nov.

Taxon classificationAnimaliaHymenopteraFormicidae

43B0F225-C982-5322-888E-03A8D277187D

http://zoobank.org/29081C2B-DA66-41BE-9E62-EF67D84D226B

**Figure 43.**
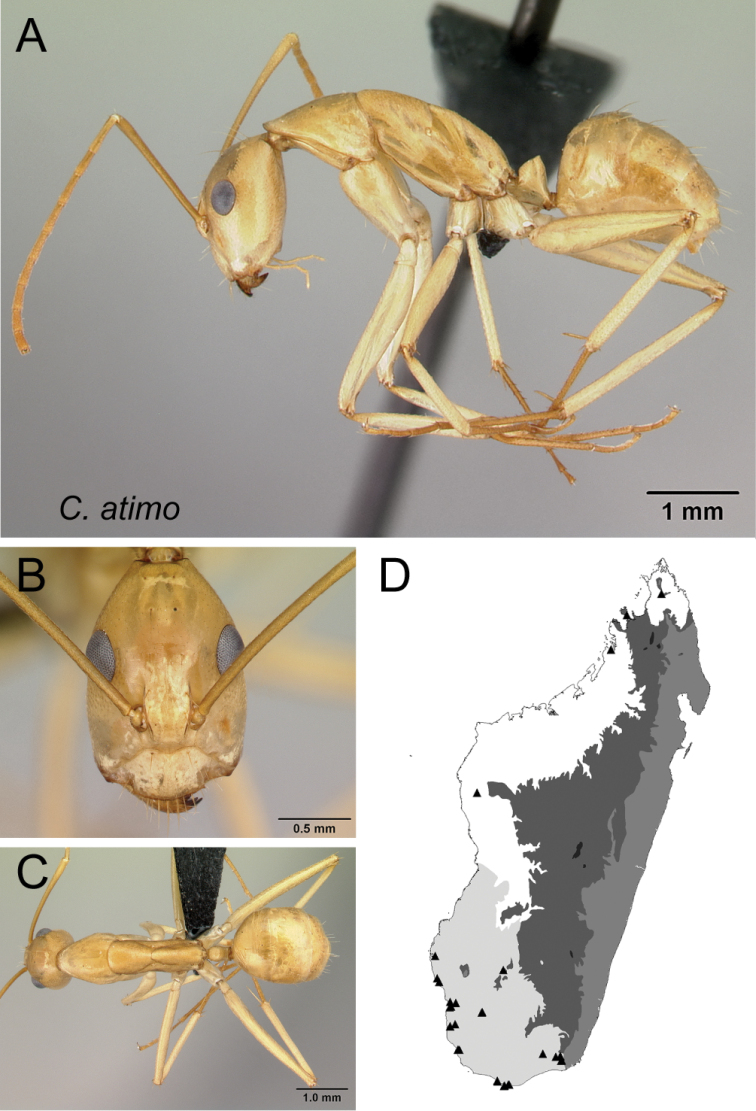
*Camponotusatimo***A** lateral view **B** head in full-face view **C** dorsal view of holotype minor worker CASENT0454042**D** distribution map.

Figs 24A , 26B , 30A , 43

##### Holotype worker.

**Madagascar**: Province **Toliara**: 3.5 km 236° SW Marovato, -25.55389, 45.25583, 230 m, spiny forest/thicket, ex rotten log, 14 Feb 2002 (Fisher, Griswold, and Arthropod Team) collection code: BLF05595, specimen code: CASENT0454042 (CAS).

##### Paratype.

1 major worker same data as holotype but specimen coded as: CASENT0454043 (major) (CAS).

##### Additional material examined.

**Madagascar: Antsiranana**: Nosy Be, RNI Lokobe, 6.3 km 112° ESE Hellville, -13.41933, 48.33117, 30 m, rainforest, (J.-J. Rafanomezantsoa et al.) (CAS); RS Ankarana, 13.6 km 192° SSW Anivorano Nord, -12.86361, 49.22583, 210 m, tropical dry forest (Fisher, Griswold et al.) (CAS); Sahamalaza Peninsula, Forêt d’Anabohazo, 21.6 km 247° WSW Maromandia, -14.30889, 47.91433, 120 m, tropical dry forest (Fisher, Griswold et al.) (CAS). **Fianarantsoa**: Forêt d’Analalava, 29.6 km 280° W Ranohira, -22.59167, 45.12833, 700 m, *Uapaca* woodland (Fisher, Griswold et al.) (CAS). **Mahajanga**: Melaky Region, District of Maintirano, Ampasy 50 km E of Maintirano, -18.004, 44.452, 85 m, dry forest (Mike, Rin’ha) (CAS). **Toliara**: 3.4 km 190° S Marovato, -25.55972, 45.2825, 160 m, spiny forest/thicket (Fisher-Griswold Arthropod Team) (CAS); 3.5 km 236° SW Marovato, -25.55389, 45.25583, 230 m, spiny forest/thicket (Fisher-Griswold Arthropod Team) (CAS); 4.4 km 148° SSE Lavanono, -25.45056, 44.97417, 60 m, spiny forest/thicket (Fisher-Griswold Arthropod Team) (CAS); 7.0 km 156° SSE Lavanono, -25.47111, 44.9885, 50 m, spiny forest/thicket (Fisher-Griswold Arthropod Team) (CAS); Anosy Region, District of Amboasary, PN Andohahela, Parcelle III, Ihazofotsy, 32 km NE Amboasary, -24.83083, 46.53617, 58 m, dry forest, spiny forest (Michael Irwin, Frank Parker, Rin’ha) (CAS); Anosy Region, District of Fort-Dauphin, PN Andohahela, Parcelle II, Tsimela, 42 km W of Fort-Dauphin, -24.93683, 46.62667, 176 m, transition forest (Michael Irwin, Frank Parker, Rin’ha) (CAS); Anosy Region, PN Andohahela, Forêt de Manatalinjo, -24.82466, 46.60111, 100 m, spiny forest/thicket (B.L. Fisher, F.A. Esteves et al.) (CAS); Atsimo Andrefana Region, District of Betioky ; RS Beza Mahafaly Parcelle Belle vue 07 km W of Research Station, -23.68983, 44.5755, 177 m, spiny forest, (Rin’ha) (CAS); Atsimo-Andrefana Region, -23.55275, 43.74471, 45 m, coastal scrub on limestone (B.L. Fisher, F.A. Esteves et al.) (CAS); Atsimo-Andrefana Region, -23.53922, 43.77935, 20 m, riparian scrub (B.L. Fisher, F.A. Esteves et al.) (CAS); Atsimo-Andrefana Region, Antsokay Arboretum, -23.41491, 43.75499, 13 m, spiny forest/thicket (B.L. Fisher, F.A. Esteves et al.) (CAS); Forêt de Beroboka, 5.9 km 131° SE Ankidranoka, -22.23306, 43.36633, 80 m, tropical dry forest (Fisher-Griswold Arthropod Team) (CAS); Forêt de Mahavelo, Isantoria River, -24.75833, 46.15717, 110 m, spiny forest/thicket (Fisher-Griswold Arthropod Team) (CAS); Forêt de Tsinjoriaky, 6.2 km 84° E Tsifota, -22.80222, 43.42067, 70 m, spiny forest/thicket (Fisher-Griswold Arthropod Team) (CAS); Mahafaly Plateau, 6.2 km 74° ENE Itampolo, -24.65361, 43.99667, 80 m, spiny forest/thicket (Fisher-Griswold Arthropod Team) (CAS); PN Andohahela, Forêt d’Ambohibory, 1.7 km 61° ENE Tsimelahy, 36.1 km 308° NW Tolagnaro, -24.93, 46.6455, 300 m, tropical dry forest (Fisher-Griswold Arthropod Team) (CAS); PN Andohahela, Forêt de Manatalinjo, 33.6 km 63° ENE Amboasary, 7.6 km 99° E Hazofotsy, -24.81694, 46.61, 150 m, spiny forest/thicket (Fisher-Griswold Arthropod Team) (CAS); PN Tsimanampetsotsa, Forêt de Bemanateza, 20.7 km 81° E Efoetse, 23.0 km 131° SE Beheloka, -23.99222, 43.88067, 90 m, spiny forest/thicket (Fisher-Griswold Arthropod Team) (CAS); PN Tsimanampetsotsa, Mitoho Cave, 6.4 km 77° ENE Efoetse, 17.4 km 170° S Beheloka, -24.04722, 43.75317, 40 m, spiny forest/thicket (Fisher-Griswold Arthropod Team) (CAS); RS Cap Sainte Marie, 12.3 km 262° W Marovato, -25.58167, 45.16833, 200 m, spiny forest/thicket (Fisher-Griswold Arthropod Team) (CAS); RS Cap Sainte Marie, 14.9 km 261° W Marovato, -25.59444, 45.14683, 160 m, spiny forest/thicket (Fisher-Griswold Arthropod Team) (CAS); 3 km E Itampolo, malaise across path of lower bench of Andrimpano Forest, -24.65783, 43.95617, 45 m, dry forest (M.E. Irwin, Rin’ha) (CAS); 5 km E Itampolo, malaise across path of plateau of Andrimpano Forest, -24.65033, 43.96317, 130 m, dry forest (M.E. Irwin, Rin’ha) (CAS); 5 km N Ampotaka, malaise on trail in Vitambany gallery forest, -24.65033, 43.96317, 86 m, Gallery forest (M.E. Irwin, Rin’ha) (CAS); Ambohimahavelona village 33 km NE of Tulear, Andoharano dry forest, -23.44083, 43.89967, 46 m, dry forest (M.E. Irwin, Rin’ha) (CAS); PN Andohahela, Ihazofotsy - Parcel III, transition forest, -24.83483, 46.48683, 80 m, transition between spiny and dry deciduous forests (M.E. Irwin, F.D. Parker, R. Harin’Hala) (CAS); Mikea Forest, deciduous dry forest, -22.90367, 43.4755, 30 m, deciduous dry forest (R. Harin’Hala) (CAS); Parcel I, RS Beza Mahafaly, near research station, -23.6865, 44.591, 165 m, dry deciduous forest (R. Harin’Hala) (CAS); PN Tsimanampetsotsa, Mitoho Forest, malaise across trail at escarpment base, -24.0485, 43.75233, 120 m, dense dry forest (M.E. Irwin, Rin’ha) (CAS); Tsimelahy - Parcel II, PN Andohahela, transition forest, -24.93683, 46.62667, 180 m, transition forest (M.E. Irwin, F.D. Parker, R. Harin’Hala) (CAS).

##### Diagnosis.

With head in full-face view, lateral margins of head anterior to eye level parallel, lacking erect hairs; in oblique profile, four or more pairs of erect hairs arranged successively from level of anterior ocular margin towards posterior cephalic margin; clypeus with distinct anterolateral corner; in profile, junction of propodeal dorsum to declivity rounded; petiole nodelike and not anteroposteriorly compressed.

##### Description.

**Minor worker.** With head in full-face view, lateral margins anterior to eye level parallel, converging abruptly towards posterior margin behind eye level; eye large and convex (EL/CS: 0.28±0.01; 0.26–0.30), interrupting lateral cephalic border, level of its posterior margin situated approximately at posterior 1/3 of head (PoOc/CL; 0.27±0.01; 0.25–0.29); frontal carinae close to each other, their distance equal to or smaller than their smallest distance to eye (FR/CS: 0.23±0.01; 0.22–0.25); clypeus with anterolateral angle and broadly convex anteromedian margin; mandible with two apical teeth distant from each other; antennal scape relatively long (SL/CS: 1.66±0.09; 1.48–1.84). Promesonotum weakly convex and mesopropodeum feebly convex (MPH/ML: 0.33±0.02; 0.29–0.36), mesonotum flat near weakly visible metanotal groove; propodeal dorsum anteriorly convex, posteriorly flat, rounding to declivity; propodeal dorsum 2 × as long as declivity. Petiole nodiform, its dorsal margin inclined posteriorly, rounding to anterior margin; anterior face 1/2 height of posterior face; femur of hind leg rounded axially, not twisted near base.

First and second gastral tergites without a pair of white spots; lateral margin of head without erect hairs; posterior cephalic margin with a pair of erect hairs; in profile, four pairs of erect hairs arranged from level of anterior margin of eye to posterior cephalic margin; erect hairs lacking from antennal scape, pubescence present; pronotum with a pair of erect hairs; posterodorsal angle of propodeum without erect hairs.

**Major worker.** With characteristics of minor worker, except for the typically broader head (CS: 3.23±0.34; 2.80–3.77; CWb/CL: 0.97±0.04; 0.89–1.03), with broadly concave posterior margin; apical 1/3 of antennal scape extending beyond posterior cephalic margin; robust mesosoma, with propodeal dorsum convex immediately posterior to metanotal groove and < 2 × as long as declivity; petiolar node compressed anteroposteriorly.

##### Distribution and biology.

*Camponotusatimo*, endemic to Madagascar, generally occurs in the southern part of the island (Fig. 43D). Its habitats range from dry forest and coastal scrub on limestone of the southwestern region to the transitional forest in the extreme southeastern region and *Uapaca* woodland in the south-central region through the spiny forest and thicket in the extreme south of the island. Field work during the past 25 years has found this species foraging mostly on the ground and through leaf litter, and very rarely on lower vegetation. It usually nests under stones, in rotten logs and soil layers, and rarely in tree stumps.

##### Discussion.

*Camponotusatimo* may be difficult to distinguish from *C.tapia* in that both species have the same body shape and the dorsum of the head is fringed with four or more pairs of erect hairs arranged successively from the level of the anterior ocular margin towards the posterior cephalic margin. However, in *C.tapia*, the junction of the propodeal dorsum to declivity surface is angulate.

*Camponotusatimo* also may be confounded with *C.strangulatus*, but the latter is characterized by the head having only three pairs of erect hairs arranged successively from the level of anterior ocular margin towards the posterior cephalic margin.

The identity of *C.atimo*, based on traditional qualitative taxonomy, has been confirmed by multivariate morphometrics. The grouping of the samples of *C.atimo* generated by the NC-clustering method is corroborated by cumulative LDA with a classification success of 100%.

##### Etymology.

This new name *atimo* is a singular non-Latin noun used in apposition, derived from the Malagasy word for “south” in reference to the restricted distribution of the species to the southern half of Madagascar.

#### ﻿ Camponotus aurosus

Taxon classificationAnimaliaHymenopteraFormicidae

Roger

CAF5ECED-B923-50F0-AEAF-02C2CD891C89

Figs 32A , 33A , 44

Camponotus aurosus Roger, 1863: 134. Syntype workers, Mauritius (Roger) (ZMHB); one syntype minor worker designated as lectotype, by present designation, AntWeb CASENT0104620 (ZMHB) [examined]. [Combination in Camponotus (Myrmepomis): Emery, 1920a: 258; in Camponotus (Myrmosericus): Forel, 1914: 268; Wheeler 1922: 1044].

##### Additional material examined.

**Mauritius**: [Mauritius, 19161], -20.3, 57.583333, (Roger) (CAS); Bassin Blanc, -20.45463, 57.47587, 481 m: (L. Lach) (CAS); Le Pouce, -20.195, 57.522222 (L. Lach) (CAS); Le Pouce, -20.195, 57.522222 (C. M. Courtois) (CAS); Macchabee Forest, -20.4, 57.45, 600 m, disturbed rainforest (P.S. Ward) (MNHN); Petite Rivière Noire Mt. -20.40883, 57.40767, 750 m, rainforest (B.L. Fisher et al.) (UCDC); Petite Rivière Noire Mt., -20.363889, 57.368333 (Michael, Madl) (CAS). **Reunion**: Mare, Longue, -21.34617, 55.73983, 450 m, rainforest (B.L. Fisher et al.) (ZMHB); Mare Longue, -21.34889, 55.74417, 361 m, Low 1/2wet protected area (J. Casquet) (CAS); Mare Longue, -21.35, 55.75, 150 m, primary forest (F. Blard) (CAS); Mare Longue, -21.363344, 55.753428 (F. Blard) (CAS).

##### Diagnosis.

In full-face view, lateral margin of head anterior to eye level diverging posteriorly and covered with erect hairs; anterior clypeal margin broadly triangular or convex; mesosoma in profile short and high; gastral tergites covered with abundant pubescence; head and gaster black, mesosoma reddish orange to brown.

##### Description.

**Minor worker.** In full-face view, lateral cephalic margins diverging posteriorly, widest at eye level, rounding evenly to posterior margin; eye protruding and large (EL/CS: 0.27±0.01; 0.25–0.29), breaking lateral margin of head, its posterior margin located at ca. posterior 1/4 of head (PoOc/CL: 0.24±0.01; 0.22–0.26); frontal carinae widely opened posteriorly (FR/CS: 0.30±0.01; 0.29–0.32), posteriorly diverging; clypeus without well-defined anterolateral angle, its anteromedian margin broadly convex or triangular; mandible with six teeth, the two apical teeth distant from each other; antennal scape relatively long (SL/CS: 1.33±0.04; 1.27–1.40). Dorsal outline of mesosoma strongly arched, metanotal groove weakly visible; propodeal dorsum almost straight, joining the declivity at a blunt angle; propodeal declivity 3/4 length of the dorsum; petiolar node short, high, and tapering dorsally, its dorsal margin inclined posteriorly and forming a blunt angle with anterior face; anterior face of petiolar node 1/2 height of posterior face; tibia of hind leg rounded axially, without basal twist.

First and second gastral tergites without a pair of white spots; lateral margin of head anterior to level of eyes with a few erect hairs, which are absent behind level of eyes; antennal scape without erect hairs but covered with appressed hairs; pronotum with a pair of erect hairs; posterior cephalic margin with three or more pairs of erect hairs; posterodorsal angle of propodeum with two pairs of erect hairs.

**Major worker.** Differing from minor worker in the following characters: larger head (CS: 2.24±0.11; 2.15–2.37; CWb/CL: 0.92±0.03; 0.87–0.95), with broadly slight medial concavity on posterior margin; apical 1/3 of antennal scape surpassing posterior cephalic margin; with mesosoma in lateral view, length of propodeal dorsum the same as height of propodeal declivity; petiolar node more compressed anteroposteriorly.

##### Distribution and biology.

**Figure 44.**
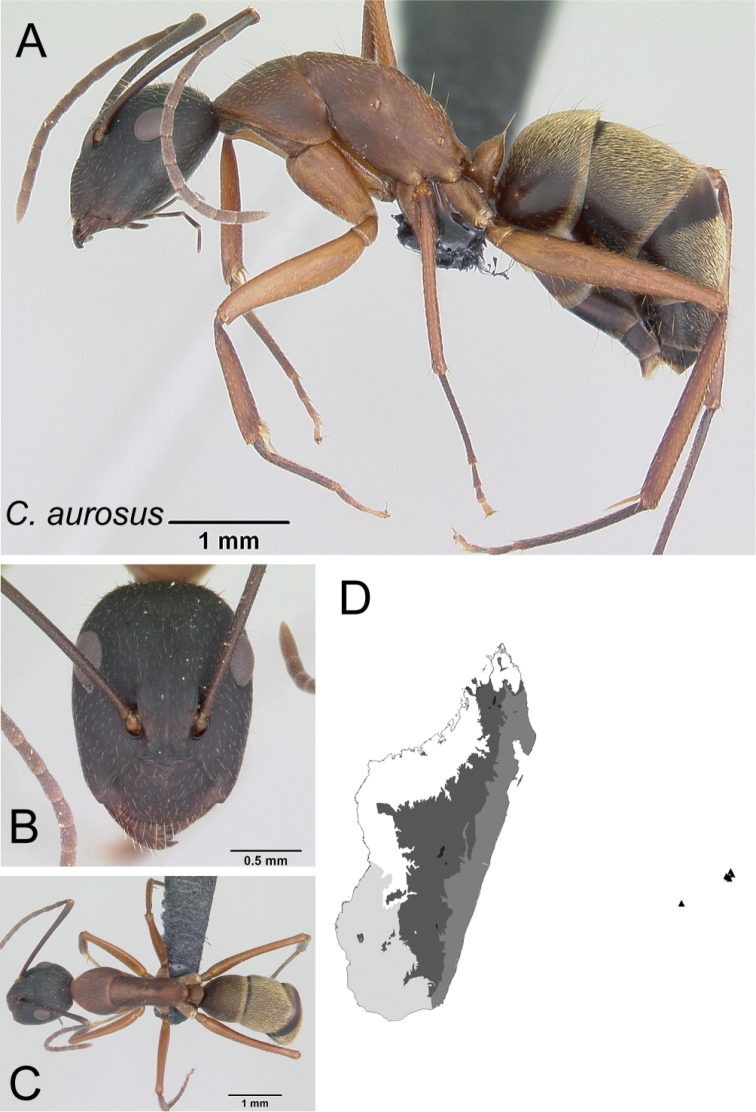
*Camponotusaurosus***A** lateral view **B** head in full-face view **C** dorsal view of minor worker CASENT0064815**D** distribution map.

The distribution of *C.aurosus* is restricted to Mauritius and Reunion islands (Fig. 44D). In these islands, this species occupies primary and disturbed rainforest habitats, where workers have been found foraging on the ground and on lower vegetation and nest sites have been located in rotten logs, in the ground, under stones, and in tree trunks.

##### Discussion.

*Camponotusaurosus* is similar to *C.hagensii* with respect to the shape of the head and the presence of erect hairs on the lateral cephalic margin, but the latter is characterized by the straight anteromedian margin of its clypeus, low and long mesosoma, and the presence of some pubescence on the gastral tergites.

The grouping of the *C.aurosus* samples in the same cluster shown by the dendrogram of multivariate morphometric analysis is corroborated by the cumulative LDA at 100% identification success. This result is congruent with that of the qualitative morphology-based study.

#### ﻿ Camponotus becki

Taxon classificationAnimaliaHymenopteraFormicidae

Santschi stat. nov.

9253523C-2901-5DBD-8373-5D6CF735DAFC

Figs 27A , 45

Camponotus radamae st. becki Santschi, 1923: 292. Syntype workers, Madagascar (R. Beck) (MHNG) [examined]; 1 syntype minor worker designated as lectotype, by present designation, AntWeb CASENT0101992 (MHNG) [examined]. Paralectotype major worker CASENT0101777 (MHNG) [examined]. [First available use of Camponotus (Myrmoturba) maculatus r. radamae var. becki Forel, 1914: 251; unavailable name]. Stat. nov. The worker specimens that are the basis of the unavailable name Camponotus (Myrmoturba) radamae st. becki var altior Santschi, 1923: 292 from Madagascar, Monts Andringitra, point culminant de l’île (Descarpentries) are referred here, AntWeb CASENT0101100 (NHMB) [examined].

##### Additional material examined.

**Madagascar: Fianarantsoa**: PN Andringitra, Plateau d’Andohariana, 35.9 km 205° Ambalavao, -22.15233, 46.89917, 2000 m, ericoid thicket (B.L. Fisher et al.) (CAS); PN Andringitra, Plateau d’Andohariana, 39.8 km 204° Ambalavao, -22.18767, 46.90083, 2150 m, rubicole thicket at base of cliff (B.L. Fisher et al.) (CAS); PN Andringitra, Plateau d’Andohariana, base of Pic d’Ivangomena, -22.2, 46.9, 2050 m, montane rainforest, (Goodman leg.) (CAS); PN Andringitra, 8.5 km SE Antanitotsy, -22.16667, 46.96667, 1990 m, montane rainforest (Sylvain) (CAS).

##### Diagnosis.

With head in full-face view, lateral margins of head anterior to eye level parallel and lacking erect hairs, lateral cephalic margin rounding to posterior margin, anteromedian clypeal margin truncate; propodeal dorsum more or less straight and joining declivity surface at a broad angle; body color black to dark brown.

##### Description.

**Minor worker.** With head in full-face view, lateral margins anterior to level of eye parallel, rounding evenly to a straight posterior margin; eye more or less convex (EL/CS: 0.27±0.02; 0.25–0.30), not breaking lateral cephalic margin, level of its posterior margin located generally at posterior 1/4 of head (PoOc/CL: 0.26±0.01; 0.24–0.27); frontal carinae posteriorly parallel (FR/CS: 0.27±0.01; 0.26–0.29); clypeus without well-defined anterolateral angle and with anteromedian margin broadly convex; mandible with two apical teeth distantly spaced; antennal scape relatively long (SL/CS: 0.27±0.01; 0.26–0.29). Promesonotum weakly convex, mesopropodeum generally flat; mesonotum with posterior portion flat immediately anterior to a weakly visible metanotal groove; propodeal dorsum anteriorly convex and posteriorly flat, joining declivity blunt angle; propodeal dorsum 2 × as long as declivity. Petiolar node short and high, its dorsal margin rounding to anterior face, height of its anterior face 2/3 that of posterior face; femur of hind leg flattened laterally, twisted near base.

First and second gastral tergites usually without white spots; lateral margin of head without erect hairs; two erect hairs near posterior margin of head; antennal scape only covered with appressed hairs. Pronotum with a pair of erect hairs; posterodorsal angle of propodeum with a pair of erect hairs.

**Major worker.** With characteristics of minor worker except: enlarged head (CS: 2.80±0.23; 2.54–2.96; CWb/CL: 0.97±0.03; 0.94–1.00) with slight, medially concave posterior margin; apical 1/3 of antennal scape surpassing posterior cephalic margin; robust mesosoma, metanotum distinctly visible; propodeal dorsum same length as declivity; petiolar node flattened anteroposteriorly.

##### Distribution and biology.

**Figure 45.**
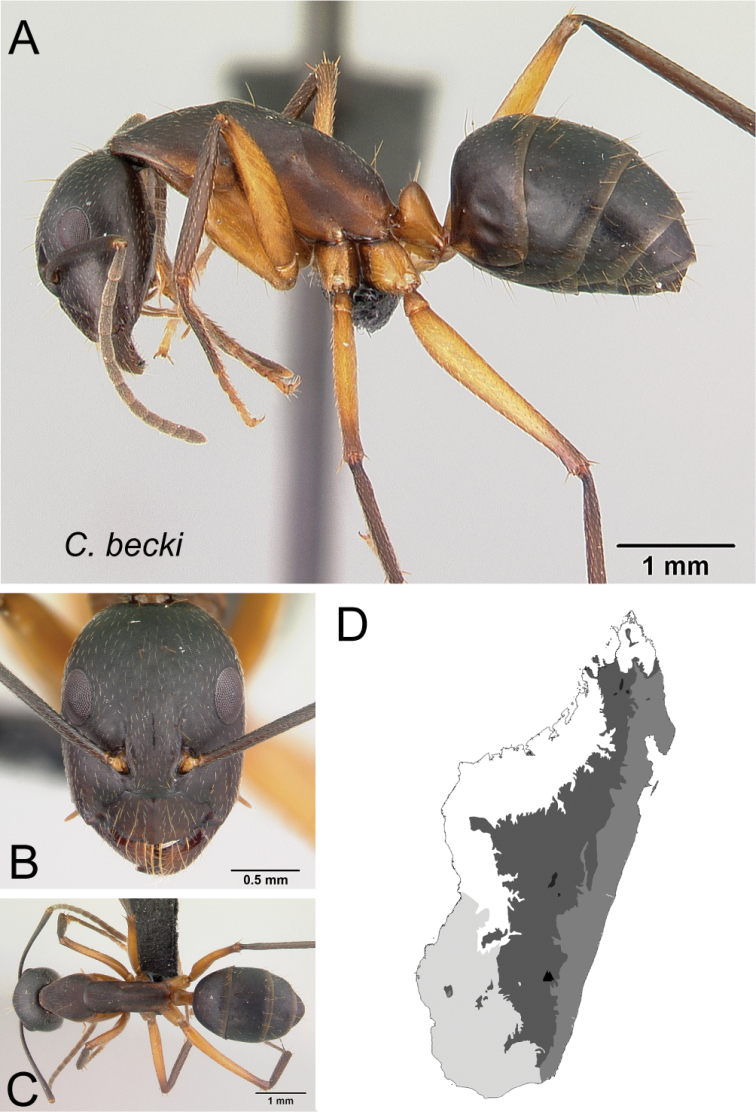
*Camponotusbecki***A** lateral view **B** head in full-face view **C** dorsal view of minor worker CASENT0071380**D** distribution map.

Based on recent collection data, *Camponotusbecki* is restricted to the ericoid thickets and montane rainforests of the PN Andringitra (Fig. 45 D). The species has been found nesting under stones and foraging on the ground.

##### Discussion.

See discussion under *C.asara*. The taxonomic categorization of *C.becki* based on the study of qualitative morphology is supported by the NC-clustering method. This species is identified by LDA at 100% success.

#### ﻿ Camponotus bemaheva sp. nov.

Taxon classificationAnimaliaHymenopteraFormicidae

8D1E409B-9912-598B-A8C2-7167C8601D77

http://zoobank.org/1AF0EE48-7F21-4744-961F-86BCEE7A9029

Figs 7B , 13A , 46

##### Holotype worker.

**Madagascar**: Province **Toliara**: PN Zombitse, 19.8 km 84° E Sakaraha, -22.84333, 44.71, 770 m, tropical dry forest, pitfall trap (Fisher, Griswold et al.) 05–09 Feb 2003, collection code: BLF07507, specimen code: CASENT0050097 (CAS).

##### Additional material examined.

**Madagascar: Antsiranana**: RS Ankarana, 13.6 km 192° SSW Anivorano Nord, -12.86361, 49.22583, 210 m, tropical dry forest (Fisher, Griswold et al.) (CAS). **Fianarantsoa**: Forêt d’Analalava, 29.6 km 280° W Ranohira, -22.59167, 45.12833, 700 m, tropical dry forest (Fisher, Griswold et al.) (CAS); PN Isalo, Sahanafa River, 29.2 km 351° N Ranohira, -22.31333, 45.29167, 500 m, gallery forest (Fisher, Griswold et al.) (CAS). **Mahajanga**: PN Ankarafantsika, Forêt de Tsimaloto, 18.3 km 46° NE de Tsaramandroso, -16.22806, 47.14361, 135 m, tropical dry forest (Fisher, Griswold et al.) (CAS). **Toliara**: 48 km ENE Morondava, -20.06667, 44.65, 30 m, tropical dry forest (D.M. Olson) (PSWC), Androy Region, District of Tsihombe, 74 km S of Tsihombe, RS Cap Ste Marie, -25.58767, 45.163, 36 m, spiny bush (Rin’Ha, Mike) (CAS); Atsimo Andrefana Region, District of Betioky ; RS Beza Mahafaly, Parcelle Belle vue, 07 km W of Research Station, -23.68983, 44.5755, 177 m, spiny forest, (Rin’ha) (CAS); Atsimo Andrefana Region, District of Betioky, 30 km E Betioky, RS Beza Mahafaly (Around Research Station), -23.6865, 44.591, 165 m, Galery dry deciduous forest (Rin’Ha, Mike) (CAS); Atsimo Andrefana Region, District of Sakaraha, PN Zombitse; 60 m E of ANGAP Entrance office, -22.8865, 44.69217, 838 m, deciduous spiny forest (Rin’Ha, Mike) (CAS); Atsimo Andrefana Region, District of Sakaraha, PN Zombitse; 900 m N from ANGAP Entrance office, -22.8405, 44.73117, 823 m, spiny deciduous forest (Rin’Ha, Mike) (CAS); FC Analavelona, 29.2 km 343° NNW Mahaboboka, -22.675, 44.19, 1100 m, montane rainforest (Fisher, Griswold et al.) (CAS); Forét de Kirindy, -20.0671, 44.65723, 50 m (H. Wood & J. Miller) (CAS); Forêt de Kirindy, 15.5 km 64° ENE Marofandilia, -20.06915, 44.66042, 30 m, tropical dry forest (B.L. Fisher et al.) (CAS); Makay Mts., -21.227, 45.33222, 475 m, Gallery forest on sandy soil (B.L. Fisher et al.) (CAS); Manombo, -22.80707, 43.76375, 222 m, gallery forest, TS1 (Frontier Wilderness Project) (CAS); Menabe Region, District of Morondava, Beroboka village 45 km NE of Morondava, Antsarongaza dry forest 07,5 Km E of Beroboka, -19.9775, 44.66633, 50 m, dry forest (M. Irwin, Rin’ha) (CAS); Menabe Region, District of Morondava, Beroboka village, 45 km NE of Morondava, Antsarongaza galery forest, 07 km E of Beroboka, -19.9775, 44.66533, 45 m, Galery forest (M. Irwin, Rin’ha) (CAS); PN Zombitse, 19.8 km 84° E Sakaraha, -22.84333, 44.71, 770 m, tropical dry forest (Fisher, Griswold et al.) (CAS); Vohibasia Forest, 59 km NE Sakaraha, -22.46667, 44.85, 780 m (Sylvain) (CAS); near ANGAP office, PN Zombitse, -22.8865, 44.69217, 840 m, deciduous spiny forest (R. Harin’Hala) (CAS); Parcel I, RS Beza Mahafaly, near research station, -23.6865, 44.591, 165 m, dry deciduous forest (R. Harin’Hala) (CAS).

##### Diagnosis.

In full-face view, lateral cephalic margins converge posteriorly towards eye level and without erect hairs posterior to eye level; two apical teeth of mandible positioned close to each other.

##### Description.

**Minor worker.** In full-face view, head sides converging progressively towards posterior margin; eye convex and large (EL/CL: 0.30±0.01; 0.29–0.34), breaking lateral cephalic borders, level of its posterior margin generally situated at posterior 1/3 to 1/4 of head (PoOc/CL: 0.26±0.01; 0.24–0.28); frontal carinae posteriorly parallel to each other (FR/CS: 0.26±0.01 0.25–0.29); clypeus without well-defined anterolateral angle and with broadly convex anteromedian margin; mandible with six teeth, of which the two apical are close together; antennal scape relatively long (SL/CS: 1.63±0.10; 1.47–1.87) with suberect hairs inclined 30° and pubescence. Promesonotum weakly convex, mesopropodeum almost flat, mesonotum flat immediately anterior to metanotal groove; metanotal groove weakly visible; propodeal dorsum anteriorly convex and posteriorly flat; junction of dorsal margin to declivity rounded; propodeal declivity height 1/2 length of propodeal dorsum; petiole nodiform, its dorsal margin rounding to anterior margin; anterior face 1/2 height of posterior face; tibia of hind leg rounded, not twisted basally.

First and second gastral tergites without a pair of white spots; erect hairs covering lateral margin of head anterior to eye level but absent behind eyes; posterior margin of head with more than six erect hairs; antennal scape with suberect hairs inclined at ca. 30° and pubescence; posterodorsal angle of propodeum with a pair of erect hairs.

**Major worker.** Unknown

##### Distribution and biology.

**Figure 46.**
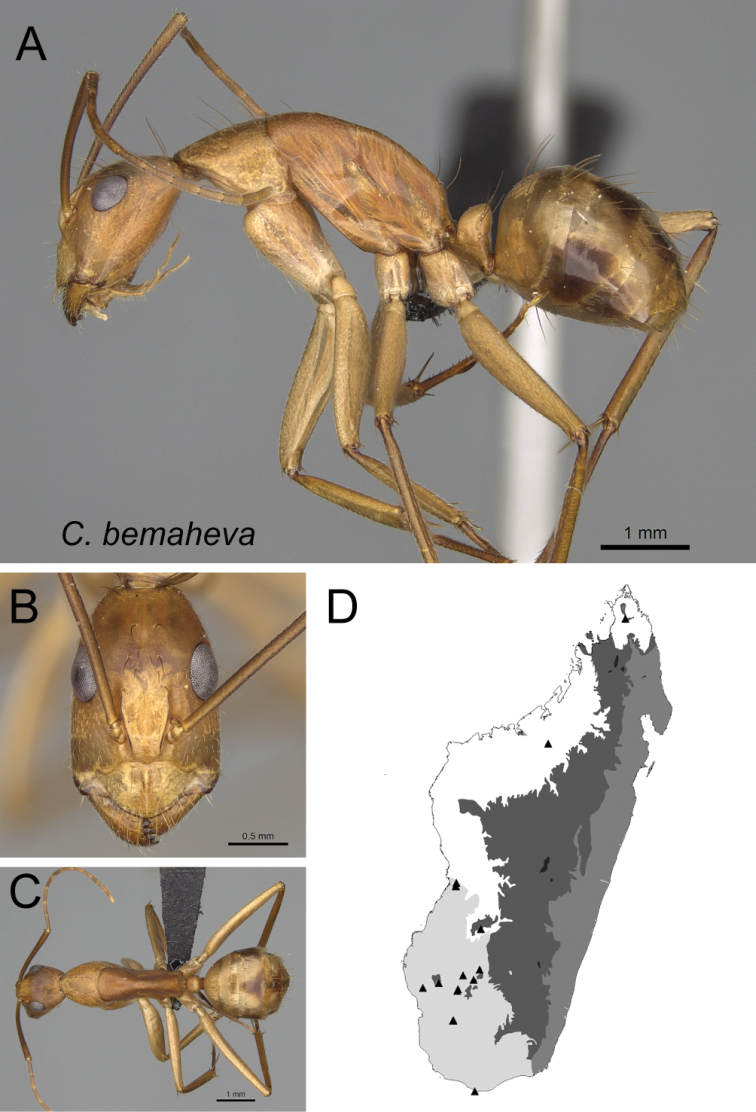
*Camponotusbemaheva***A** lateral view **B** head in full-face view **C** dorsal view of minor worker CASENT0154158**D** distribution map.

*Camponotusbemaheva* is restricted to the spiny bush and thicket of the southern region and the dry forest habitats of western Madagascar (Fig. 46D). The few colony nests that have been found were collected from rotten logs and under stones, but workers have been collected while foraging on the ground or on low vegetation.

##### Discussion.

Workers of *C.bemaheva* look very similar to those of *C.daraina* in that they have closely spaced apical teeth on the mandibles and the lateral margins of their head converge posteriorly towards eye level, but the latter is characterized by the presence of erect hairs on the lateral cephalic margin posterior to eye level.

The alignment of *C.bemaheva* in the same cluster displayed by the dendrogram of multivariate morphometric method is confirmed by the cumulative LDA at 100% identification success, validating the taxonomy hypothesized by qualitative morphology.

##### Etymology.

The new species *bemaheva* is a non-Latin proper noun used in apposition and is named in honor of Fidelis Bemaheva for his help collecting ants in the Malagasy region.

#### ﻿ Camponotus boivini

Taxon classificationAnimaliaHymenopteraFormicidae

Forel stat. rev.

304B6410-4056-56F4-ACC2-535353E167AB

Figs 23B , 47

Camponotus maculatus r. boivini Forel, 1891: 34. Syntype workers, queen and male, Madagascar (Sikora) (MHNG); 1 syntype minor worker designated as lectotype, by present designation, AntWeb CASENT0101343 (MHNG) [examined]. Paralectotypes with same data as lectotype but: 1 minor worker CASENT0101349, 2 major workers CASENT0101342, CASENT0101348 and 1 male CASENT0101607 (MHNG) [examined]. Raised to species by Dalla Torre 1893: 223. As subspecies of Camponotusmaculatus by Emery, 1895: 337; Wheeler 1922: 1040. As subspecies of Camponotushova by Emery, 1920b: 6, 1925: 86. Stat. rev.
Camponotus maculatus st. fairmairei Santschi, 1911a: 130. Syntype workers Madagascar, colony living with larvae of Fulgorids (Fairmaire, 1900) (NHMB); 1 syntype minor worker designated as lectotype, by present designation, AntWeb CASENT0101102 (NHMB) [examined]. [Camponotushovafairmairei: as subspecies of Camponotushova by Emery, 1920b: 6; Emery 1925: 86]. Syn. nov.

##### Note.

One major worker and one male specimen that are labeled respectively with CASENT0104638 and CASENT0104637 (ZMHB), were collected from Mahajanga, and are labeled with an unpublished name “Camponotusmaculatussubsp.hovavar.laticollus” by Forel. In the present study, the two specimens are identified as *Camponotusboivini*.

##### Additional material examined.

**Madagascar: Antananarivo**: Alasora, -18.96245, 47.58925, 1434 m, eucalyptus plantation (B.L. Fisher et al.) (CAS); Andohony I Non Protected Area, 22.62 km SW Antsirabe, -20.06784, 46.99068, 1451 m, Savannah grassland (A. Ravelomanana) (CAS); Andohony II Non Protected Area, 22.71 km SW Antsirabe, -20.06904, 46.99199, 1398 m, Savannah grassland (A. Ravelomanana) (CAS); Ankalalahana, -18.99666, 47.1118, 1353 m, *Uapaca* woodland, (H.E. Marti et al.) (CAS); Ankalalahana, -19.00659, 47.1122, 1375 m, *Uapaca* woodland (B.L. Fisher et al.) (CAS); Antaponimanadala III Non Protected Area, 6.55 km E Manalalondo, -19.25583, 47.17751, 1987 m, Savannah grassland (A. Ravelomanana) (CAS); Antaponimanadala IV Non Protected Area, 6.66 km E Manalalondo, -19.25361, 47.17634, 1951 m, Savannah grassland (A. Ravelomanana) (CAS); Antsirabe, -19.866, 47.0355, 1550 m, urban/garden (B.L. Fisher et al.) (CAS); Beapombo III Non Protected Area, 22.30 km SW Antsirabe, -20.0662, 46.99839, 1612 m, *Uapaca* woodland (A. Ravelomanana) (CAS). **Antsiranana**: Ampasindava, Forêt d’Ambilanivy, 3.9 km 181° S Ambaliha, -13.79861, 48.16167, 600 m, rainforest (Fisher, Griswold et al.) (CAS); Forêt Ambato, 26.6 km 33° Ambanja, -13.4645, 48.55167, 150 m, rainforest (B.L. Fisher) (CAS); Forêt d’Andavakoera, 21.4 km 75° ENE Ambilobe; 4.6 km 356° N Betsiaka, -13.11833, 49.23, 425 m, rainforest (B.L. Fisher) (CAS); Forêt d’Ampondrabe, 26.3 km 10° NNE Daraina, -12.97, 49.7, 175 m, tropical dry forest (B.L. Fisher) (CAS); Forêt de Binara, 7.5 km 230° SW Daraina, -13.255, 49.61667, 375 m, tropical dry forest (B.L. Fisher) (CAS); Forêt d’Orangea, 3.6 km 128° SE Remena, -12.25889, 49.37467, 90 m, littoral rainforest (Fisher, Griswold et al.) (CAS); Galoko chain, Mont Galoko, -13.58745, 48.71419, 380 m, rainforest (B.L. Fisher et al.) (CAS); Montagne des Français, 7.2 km 142° SE Antsiranana (=Diego Suarez), -12.32278, 49.33817, 180 m, tropical dry forest, (J.-J. Rafanomezantsoa et al.) al. (CAS); Nosy Ankao forest, -12.79164, 49.82378, 25 m, tropical dry forest (B.L. Fisher et al.) (CAS); Nosy Be, RNI Lokobe, 6.3 km 112° ESE Hellville, -13.41933, 48.33117, 30 m, rainforest (Fisher, Griswold et al.) (CAS); Réserve Analamerana, 16.7 km 123° Anivorano-Nord, -12.80467, 49.37383, 225 m, tropical dry forest (B.L. Fisher) (CAS); Réserve Analamerana, 28.4 km 99° Anivorano-Nord, -12.74667, 49.49483, 60 m, tropical dry forest (B.L. Fisher) (CAS); RS Ambre, 3.5 km 235° SW Sakaramy, -12.46889, 49.24217, 325 m, tropical dry forest (Fisher, Griswold et al.) (CAS); RS Ankarana, 22.9 km 224° SW Anivorano Nord, -12.90889, 49.10983, 80 m, tropical dry forest (Fisher, Griswold et al.) (CAS); RS Ankarana, Anilotra, -12.90981, 49.11057, 98 m, Dry forest on tsingy (B.L. Fisher et al.) (CAS). **Fianarantsoa**: 28 km SSW Ambositra, Ankazomivady, -20.775, 47.16833, 1670 m, grassland (B.L. Fisher) (CAS); Amoron’i Mania Region, District of Ambositra, Italaviana *Uapaca* forest, 35 km SE of Antsirabe, -20.17333, 47.086, 1359 m, *Uapaca* forest (Rin’Ha, Mike) (CAS); PN Isalo, Ampangabe I Non Protected Area, 21.4 km W Itremo, -20.61111, 46.60688, 1414 m, savannah woodland (A. Ravelomanana) (CAS); Ampangabe IV Non Protected Area, 21.37 km W Itremo, -20.61278, 46.60774, 1417 m, savannah woodland (A. Ravelomanana) (CAS); Antohatsahomby IV Non Protected Area, 22.67 km NW Itremo, -20.56306, 46.58097, 1708 m, *Uapaca* woodland (A. Ravelomanana) (CAS); Antohatsahomby V Non Protected Area, 22.63 km NW Itremo, -20.56722, 46.57923, 1726 m, *Uapaca* woodland (A. Ravelomanana) (CAS); Forêt d’Atsirakambiaty, 7.6 km 285° WNW Itremo, -20.59333, 46.56333, 1550 m, grassland (Fisher, Griswold et al.) (CAS); Manandriana II Non Protected Area, 27.12 km SW Ambositra, -20.7325, 47.09461, 1589 m, Savannah grassland (A. Ravelomanana) (CAS); Soanierenana II Non Protected Area, 25.61 km SW Ambositra, -20.72194, 47.10896, 1732 m, savannah grassland (A. Ravelomanana) (CAS); Soanierenana IV Non Protected Area, 25.22 km SW Ambositra, -20.72389, 47.10705, 1736 m, savannah grassland (A. Ravelomanana) (CAS); Soanierenana IV Non Protected Area, 25.22 km SW Ambositra, -20.72389, 47.10705, 1736 m, savannah grassland (A. Ravelomanana) (CAS). **Mahajanga**: Boeny Region, District of Marovoay, PN Ankarafantsika, Ampijoroa SF, 160 km North of Maevatanana on RN 04, -16.31933, 46.81333, 42 m, deciduous forest (Mike, Rin’ha) (CAS); Boeny Region: PN Ankarafantsika, Ambodimanga, -16.32342, 46.82443, 75 m, dry forest, Canopy Bootcamp, (A. Karunakaran) (CAS). Forêt Ambohimanga, 26.1 km 314° Mampikony, -15.96267, 47.43817, 250 m, tropical dry forest (B.L. Fisher) (CAS). Forêt de Tsimembo, 11.0 km 346° NNW Soatana, -18.99528, 44.4435, 50 m, tropical dry forest (Fisher-Griswold Arthropod Team) (CAS). Mahavavy River, 6.2 km 145° SE Mitsinjo, -16.05167, 45.90833, 20 m, gallery forest (Fisher, Griswold et al.) (CAS). Manerinerina, 76.6 km N Antsohihy, -14.10744, 48.11046, 247 m, disturbed forest (B.L. Fisher et al.) (CAS). PN Ankarafantsika, Ampijoroa SF, 5.4 km 331° NW Andranofasika, -16.29889, 46.813, 70 m, tropical dry forest (Fisher, Griswold et al.) (CAS); PN Ankarafantsika, Ampijoroa SF, 40 km 306° NW Andranofasika, -16.32083, 46.81067, 130 m, tropical dry forest (Fisher, Griswold et al.) (CAS); PN Ankarafantsika, Forêt de Tsimaloto, 18.3 km 46° NE de Tsaramandroso, -16.22806, 47.14361, 135 m, tropical dry forest (Fisher, Griswold et al.) (CAS); PN Baie de Baly, 12.4 km 337° NNW Soalala, -16.01, 45.265, 10 m, tropical dry forest (Fisher, Griswold et al.) (CAS); PN Namoroka, 16.9 km 317° NW Vilanandro, -16.40667, 45.31, 100 m, tropical dry forest (Fisher, Griswold et al.) (CAS); PN Namoroka, 17.8 km 329° WNW Vilanandro, -16.37667, 45.32667, 100 m, tropical dry forest (Fisher, Griswold et al.) (CAS); PN Namoroka, 9.8 km 300° WNW Vilanandro, -16.46667, 45.35, 140 m, tropical dry forest (Fisher, Griswold et al.) (CAS); PN Tsingy de Bemaraha, 10.6 km ESE 123° Antsalova, -18.70944, 44.71817, 150 m, tropical dry forest on Tsingy (Fisher-Griswold Arthropod Team) (CAS); PN Tsingy de Bemaraha, 2.5 km 62° ENE Bekopaka, Ankidrodroa River, -19.13222, 44.81467, 100 m, tropical dry forest on Tsingy (Fisher-Griswold Arthropod Team) (CAS); PN Tsingy de Bemaraha, 3.4 km 93° E Bekopaka, Tombeau Vazimba, -19.14194, 44.828, 50 m, tropical dry forest (Fisher-Griswold Arthropod Team) (CAS); Réserve d’Ankoririka, 10.6 km 13° NE de Tsaramandroso, -16.26722, 47.04861, 210 m, tropical dry forest (Fisher, Griswold et al.) (CAS); Réserve forestière Beanka, 50.7 km E Maintirano, -17.88021, 44.46877, 140 m, tropical dry forest edge (B.L. Fisher et al.) (CAS); Réserve forestière Beanka, 52.7 km E Maintirano, -18.0622, 44.52587, 300 m, tropical dry forest on tsingy (B.L. Fisher et al.) (CAS); Réserve forestière Beanka, 53.6 km E Maintirano, -18.04014, 44.53394, 272 m, tropical dry forest on tsingy (B.L. Fisher et al.) (CAS); RS Bemarivo, 23.8 km 223° SW Besalampy, -16.925, 44.36833, 30 m, tropical dry forest (Fisher, Griswold et al.) (CAS); Sofia Region, District of Port-Berger, Ambovomamy 20 km N of Port-Berger, -15.45117, 47.61333, 86 m, secondary forest on white sandy area (Mike, Frank, Rin’ha) (CAS). **Toamasina**: Ambatovy, 12.4 km NE Moramanga, -18.85813, 48.28488, 1040 m, grassland (B.L. Fisher et al.) (CAS); PN Andasibe, botanic garden near entrance, west of ANGAP office, -18.925172, 48.418651, 1025 m, tropical forest (M.E. Irwin, R. Harin’Hala) (CAS). **Toliara**: Forêt de Beroboka, 5.9 km 131° SE Ankidranoka, -22.23306, 43.36633, 80 m, tropical dry forest (Fisher-Griswold Arthropod Team) (CAS); Forêt de Kirindy, 15.5 km 64° ENE Marofandilia, -20.06855, 44.65956667, 30 m, tropical dry forest (B.L. Fisher) (CAS); Forêt de Kirindy, 15.5 km 64° ENE Marofandilia, -20.045, 44.66222, 100 m, tropical dry forest (Fisher-Griswold Arthropod Team) (CAS); Forêt de Tsinjoriaky, 6.2 km 84° E Tsifota, -22.80222, 43.42067, 70 m, spiny forest/thicket (Fisher-Griswold Arthropod Team) (CAS); Makay Mts., -21.30997, 45.12946, 590 m, dry forest on sandy soil (B.L. Fisher et al.) (CAS); Makay Mts., -21.31664, 45.1296, 620 m, dry forest on sandy soil (B.L. Fisher et al.) (CAS); Makay Mts., -21.25864, 45.16412, 500 m, gallery forest with bamboo (B.L. Fisher et al.) (CAS); Makay Mts., -21.21985, 45.32396, 500 m, gallery forest on sandy soil (B.L. Fisher et al.) (CAS); Makay Mts., -21.20978, 45.34184, 525 m, gallery forest on sandy soil (B.L. Fisher et al.) (CAS); PN Tsimanampetsotsa, Forêt de Bemanateza, 20.7 km 81° E Efoetse, 23.0 km 131° SE Beheloka, -23.99222, 43.88067, 90 m, spiny forest/thicket (Fisher-Griswold Arthropod Team) (CAS); 5 km E Itampolo, Malaise across path of plateau of Andrimpano Forest, -24.65033, 43.96317, 130 m, dry forest (M.E. Irwin, Rin’ha) (CAS); RS Kalambatritra, Ambinanitelo, -23.45373, 46.45773, 1345 m, grassland (B.L. Fisher et al.) (CAS).

##### Diagnosis.

Lateral cephalic margins approximately parallel in full-face view; two apical teeth of mandible normally spaced; antennal scape covered with suberect hairs inclined at ca. 45°; in lateral view, posterior 1/2 of mesonotum to posterodorsal corner of propodeum somewhat convex, propodeal dorsum ca. 2 × as long as the height of declivity surface.

##### Description.

**Minor worker.** In full-face view, lateral cephalic borders anterior to level of eye parallel to each other, converging progressively towards posterior margin behind eye level; eye protruding and large (EL/CS: 0.32±0.02; 0.29–0.36), breaking lateral cephalic margins; head sides behind eye level 1/4 length of head (PoOc/CL: 0.27±0.01; 0.24–0.30); frontal carinae widely diverging posteriorly (FR/CS: 0.32±0.01; 0.30–0.35); clypeus with anterolateral angle, its anteromedian margin with blunt angle or convex; mandible with two apical teeth distant from each other; antennal scape relatively long (SL/CS: 1.37±0.10; 1.22–1.56). Promesonotum weakly convex and mesopropodeum feebly convex, mesonotum with posterior portion flat immediately anterior to metanotal groove, metanotum weakly visible, propodeal dorsum anteriorly convex and posteriorly flat, dorsal margin of propodeum and declivity joining at a blunt angle, height of propodeal declivity 1/2 length of propodeal dorsum. Petiolar node flattened, short, and high, without noticeable dorsal margin; tibia of hind leg rounded axially, without twist in the basal portion.

First and second gastral tergites without a pair of white spots; lateral margin of head anterior to eyes level with erect hairs, which are absent behind eyes; posterior margin of head with a pair of erect hairs; antennal scape covered with erect hairs inclined at 45°; posterodorsal angle of propodeum with a pair of erect hairs.

**Major worker.** Differing from minor worker in having larger head (CS: 2.29±0.28; 1.96–3.12; CWb/CL: 0.91±0.04; 0.86–0.99), short antennal scape barely surpassing posterior cephalic margin; robust mesosoma with distinct metanotum, and petiolar node as high as long. Other characters as in minor worker.

##### Distribution and biology.

**Figure 47.**
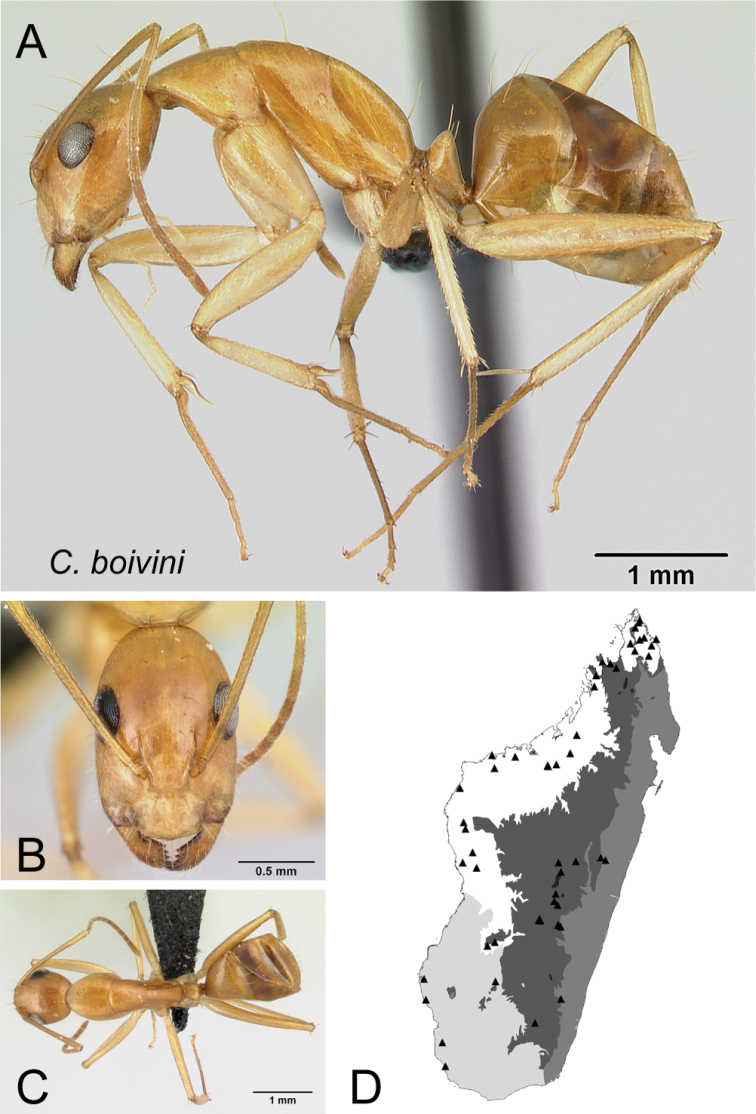
*Camponotusboivini***A** lateral view **B** head in full-face view **C** dorsal view of minor worker CASENT0486575**D** distribution map.

The distribution of *C.boivini* is generally limited to western and the high plateau of Madagascar (Fig. 47D). This species occurs mostly in dry forest habitats in the north through spiny forest areas in the south. Along its north-south range it can be found in gallery forests, disjunct montane rainforest, *Uapaca* woodlands, and savannah grasslands. When nesting in rotten logs, rotting tree stumps, under stones, in dead branches, twigs on the ground, and in the soil, *C.boivini* typically forages in leaf litter and seldom on the forest floor and low vegetation.

##### Discussion.

*Camponotusboivini* may be confused with *C.cemeryi* but the latter is characterized by a short and high mesosoma which shows a strongly convex dorsal outline. *Camponotusboivini* can be confounded with *C.mixtellus* but workers of the latter species have an antennal scape covered with suberect hairs inclined at ca. 30°, are generally larger (CS: 1.56±0.13; 1.16–1.83; ML: 2.76±0.16; 3.10–3.45), and are mostly found in the rainforest of the region.

Santschi (1911a) originally described Camponotusmaculatusst.fairmairei without any comparison of the specimens to those of other species, though his description corresponds to the type specimens of *C.boivini*. The observation of the samples and the distributional data obtained from the recent ant survey in Madagascar combined with this information are sufficiently enough to reasonably synonymize Camponotusmaculatusst.fairmairei here.

The definition of *C.boivinii* based on traditional qualitative morphology is congruent with the results achieved by the exploratory analysis and the cumulative LDA, which has an identification success of 100%.

#### ﻿ Camponotus bozaka sp. nov.

Taxon classificationAnimaliaHymenopteraFormicidae

1556097A-8D56-57FE-A6F4-D209B648FA52

http://zoobank.org/CAD5FB8A-8FF4-47C6-9590-A7E8342C6839

Figs 26A , 28B , 48

##### Holotype worker.

**Madagascar**: Province **Fianarantsoa**: Parc National d’Andringitra, Plateau d’Andohariana, 39.8 km 204° Ambalavao, -22.18767, 46.90083, 2150 m, rubicole thicket at base of cliff, under stone, 16 Apr 2006 (B.L. Fisher et al.) collection code: BLF13773, specimen code: CASENT0217309 (CAS).

##### Paratype.

1 major worker with same data as holotype but with specimen code CASENT0071370 (CAS).

##### Additional material examined.

**Madagascar: Fianarantsoa**: Manandriana I Non Protected Area, 27.11 km SW Ambositra, -20.73194, 47.09413, 1590 m, Savannah grassland (A. Ravelomanana) (CAS); Manandriana III Non Protected Area, 27.25 km SW Ambositra, -20.73333, 47.09391, 1578 m, Savannah grassland (A. Ravelomanana) (CAS); PN Andringitra, Plateau d’Andohariana, 39.8 km 204° Ambalavao, -22.18767, 46.90083, 2150 m, rubicole thicket at base of cliff (B.L. Fisher et al.) (CAS).

##### Diagnosis.

With head in full-face view, lateral margin of head anterior to eye level parallel, lacking erect hairs; clypeus with noticeable anterolateral corner; in profile, petiolar node anteroposteriorly compressed.

##### Description.

**Minor worker.** In full-face view, lateral cephalic margins anterior to level of eye approximately parallel, evenly rounding to posterior margin; eye protruding and large (EL/CS: 0.28±0.01; 0.26–0.30), breaking lateral cephalic margin, level of its posterior margin located at ca. posterior 1/4 of head (PoOc/CL: 0.25±0.01; 0.23–0.26); frontal carinae close to each other, their distance equal to or smaller than smallest distance of one to the eye (FR/CS: 0.28±0.01; 0.28–0.29); clypeus with anterolateral corners, its anteromedian margin broadly triangular or convex; mandible with two apical teeth normally spaced; antennal scape relatively short (SL/CS: 1.19±0.05; 1.08–1.25). Promesonotum weakly convex, mesopropodeum almost flat; mesonotum flat immediately anterior to metanotal groove; metanotal groove weakly visible; dorsal margin of propodeum straight and joining declivity at a noticeable angle, propodeal dorsum < 2 × as long as declivity. Petiolar node scalelike with sharp dorsal margin; femur of hind leg rounded axially and not twisted basally.

First and second gastral tergites without a pair of white spots; erect hairs on lateral margin of head not present; posterior margin of head with two erect hairs; with head in profile, four pairs of erect hairs arranged from level of anterior margin of eye to posterior cephalic margin; antennal scape covered only with appressed hairs; pronotum, mesonotum, and posterodorsal angle of propodeum with a pair of erect hairs.

**Major worker.** Differing from minor worker in the following characters: enlarged head (CS: 2.07±0.18; 1.91–2.40; CWb/CL: 0.95±0.03; 0.90–0.98) with slightly concave to almost straight posterior margin; apical 1/3 of antennal scape extending beyond posterior cephalic margin; robust mesosoma with length of propodeal dorsum approximately the same as height of declivity.

##### Distribution and biology.

**Figure 48.**
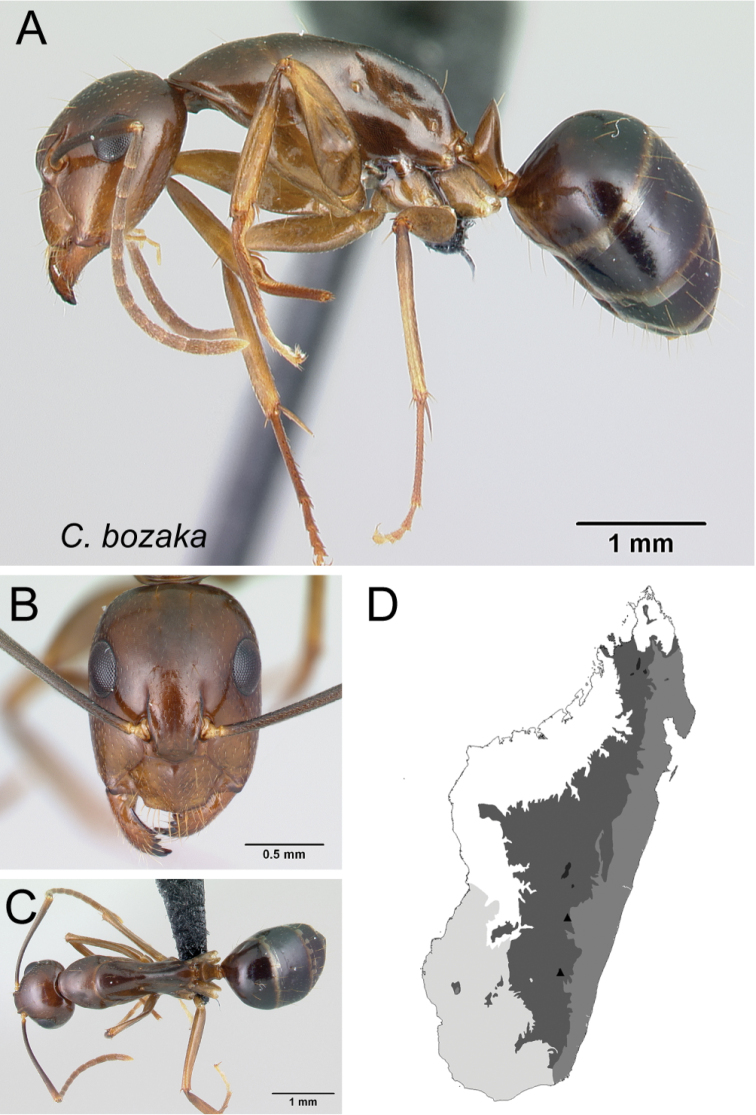
*Camponotusbozaka***A** lateral view **B** head in full-face view **C** dorsal view of holotype minor worker CASENT0217309**D** distribution map.

Known only from the south-central high plateau of Madagascar, *C.bozaka* occupies rubicole thicket, *Uapaca* woodland, savannah grassland, and shrubland habitats (Fig. 48D). Worker specimens have been found foraging through leaf litter and on low vegetation. The nest sites are in the ground or under stones.

##### Discussion.

See discussion under *C.asara*. The taxonomic identity of *C.bozaka* based on conventional qualitative morphology is supported by multivariate morphometric analysis. The grouping shown by the morphometric dendrogram and confirmed by cumulative LDA at 100% success supports the existence of the species.

##### Etymology.

This new name *bozaka* is a singular non-Latin noun used in apposition and is derived from the Malagasy word for “dried grass” in reference to the abundant grassland where this species occurs.

#### ﻿ Camponotus cemeryi

Taxon classificationAnimaliaHymenopteraFormicidae

Özdikmen stat. nov.

5A4A22A6-8C46-564C-8766-31C3C265A413

Figs 21A , 49

Camponotus hova cemeryi Özdikmen, 2010: 34. Syntype major workers, queen and male, Madagascar, Majunga (Voeltzkow); 1 syntype major worker designated as lectotype, by present designation, Madagascar, Majunga (Voeltzkow) AntWeb CASENT0101103 (NHMB) [examined]. Paralectotypes: 2 major workers with the same data as lectotype but with specimen codes CASENT0101104 (NHMB), CASENT0101894 (MHNG); 1 alate queen CASENT0101848 and 1 male CASENT0101911 (MHNG) [examined]. Replacement name of Camponotushovavar.luteolus Emery, 1925: 85. Stat. nov.

##### Note.

Camponotushovavar.luteolus Emery, is a junior secondary homonym of *Camponotusmaculatusluteolus* Emery, 1906:188. As a subspecies of *Camponotusbonariensisluteolus* Emery, 1920a: 233; [First available use of Camponotusmaculatusr.hovavar.luteolus Forel, 1897: 187; unavailable name].

##### Additional material examined.

**Madagascar: Antananarivo**: Navoatra VI Non Protected Area, 7.3 km NW Arivonimamo, -18.97889, 47.12253, 1276 m, *Uapaca* woodland (A. Ravelomanana) (CAS). **Antsiranana**: Forêt de Binara, 7.5 km 230° SW Daraina, -13.255, 49.61667, 375 m, tropical dry forest (B.L. Fisher) (CAS). **Fianarantsoa**: 28 km SSW Ambositra, Ankazomivady, -20.775, 47.16833, 1670 m, grassland (B.L. Fisher) (CAS); Ambalavao, -21.83267, 46.93867, 1020 m, urban/garden (B.L. Fisher et al.) (CAS); Ampandravelo III Non Protected Area, 10.72 km NE Ranohira, -22.53944, 45.51497, 869 m, Shrubland (A. Ravelomanana) (CAS); Ampotoampoto I National Parc, 8.02 km NW Ilakaka, -22.62833, 45.18859, 917 m, savannah woodland (A. Ravelomanana) (CAS); Ampotoampoto IV National Parc, 7.83 km NW Ilakaka, -22.62944, 45.1912, 923 m, savannah woodland (A. Ravelomanana) (CAS); dry wash, 1 km E of PN Isalo Interpretive Center, -22.62667, 45.35817, 885 m, dry wash (M.E. Irwin, F.D. Parker, R. Harin’Hala) (CAS); Forêt d’Analalava, 29.6 km 280° W Ranohira, -22.59167, 45.12833, 700 m, tropical dry forest (Fisher, Griswold et al.) (CAS); Horombe Region, District of Ihosy, PN Isalo, 900 m E of ANGAP Interpretation Center, -22.62667, 45.35817, 701 m, open area near stream (Rin’Ha, Mike) (CAS); Horombe Region, Ihosy Distric, PN Isalo, 1 km E of ANGAP Interpretation Center, -22.62667, 45.35817, 823 m, open area (Rin’Ha, Mike) (CAS); Isalo II National Parc, 12.26 km SW Ranohira, -22.61528, 45.31307, 867 m, *Bismarckia* woodland (A. Ravelomanana) (CAS); Isalo III National Parc, 12.02 km SW Ranohira, -22.61583, 45.31084, 870 m, *Bismarckia* woodland (A. Ravelomanana) (CAS); Isalo IV National Parc, 12 km SW Ranohira, -22.61472, 45.31304, 867 m, *Bismarckia* woodland (A. Ravelomanana) (CAS); RS Manombo, 32 km SE of Farafangana, -23.02183, 47.72, 36 m, Lowland rainforest (Rin’Ha, Mike) (CAS); PN Isalo, Ambovo Springs, 29.3 km 4° N Ranohira, -22.29833, 45.35167, 990 m, *Uapaca* woodland (Fisher, Griswold et al.) (CAS); PN Isalo, Sahanafa River, 29.2 km 351° N Ranohira, -22.31333, 45.29167, 500 m, gallery forest (Fisher, Griswold et al.) (CAS); radio tower, PN Ranomafana, -21.25833, 47.40717, 1130 m, forest edge, mixed tropical forest, open area (M.E. Irwin, F.D. Parker, R. Harin’Hala) (CAS); stream area, 900 m E of PN Isalo Interpretive Center, -22.62667, 45.35817, 750 m, open area near stream (R. Harin’Hala) (CAS). **Mahajanga**: PN Ankarafantsika, Ampijoroa SF, 160 km N Maevatanana, deciduous forest, -16.31944, 46.81333, 43 m, deciduous forest, Mike (Irwin, Rin’ha), Harin’Hala (CAS); Boeny Region, District of Soalala, Beaboaly Bamboo forest Station10 km SW of Soalala, 04 km of Baly village, -16.04533, 48.804, 9 m, Bamboo Forêt (Mike, Rin’ha) (CAS); Boeny Region, District of Marovoay, PN Ankarafantsika, Ampijoroa SF, 160 km North of Maevatanana on RN 04, -16.31933, 46.81333, 42 m, deciduous forest (Mike, Rin’ha) (CAS); Boeny Region, District of Soalala Analamanitra forest, 14 km SW of Mitsinjo, -16.7, 45.7, 19 m, dense dry forest (Mike, Rin’ha) (CAS); Mahavavy River, 6.2 km 145° SE Mitsinjo, -16.05167, 45.90833, 20 m, gallery forest (Fisher, Griswold et al.) (CAS); Melaky Region, District of Mintirano, Ampasy 50 km E of Maintirano, -18.004, 44.452, 85 m, dry forest (Mike, Rin’ha) (CAS); PN Baie de Baly, 12.4 km 337° NNW Soalala, -16.01, 45.265, 10 m, tropical dry forest (Fisher, Griswold et al.) (CAS); PN Namoroka, 17.8 km 329° WNW Vilanandro, -16.37667, 45.32667, 100 m, tropical dry forest (Fisher, Griswold et al.) (CAS); PN Namoroka, 9.8 km 300° WNW Vilanandro, -16.46667, 45.35, 140 m, tropical dry forest (Fisher, Griswold et al.) (CAS); Réserve forestière Beanka, 48.9 km E Maintirano, -18.02472, 44.48788, 250 m, savannah shrubland (B.L. Fisher et al.) (CAS); Réserve forestière Beanka, 50.2 km E Maintirano, -18.02127, 44.49566, 250 m, savannah woodland (B.L. Fisher et al.) (CAS); Réserve forestière Beanka, 50.7 km E Maintirano, -17.8873, 44.47113, 160 m, savannah shrubland (B.L. Fisher et al.) (CAS). **Toliara**: Androy Region, District of Tsihombe, 74 km S of Tsihombe, RS Cap Ste Marie, -25.58767, 45.163, 36 m, spiny bush (Rin’Ha, Mike) (CAS); Androy Region, District of Tsihombe, 74 km S of Tsihombe, RS Cap Ste Marie, -25.58767, 45.163, 36 m, spiny bush (Rin’Ha, Mike) (CAS); Androy Region, District of Tsihombe, 74 km S of Tsihombe, RS Cap Ste Marie, -25.58767, 45.163, 152 m, Bush (Mike, Rin’ha) (CAS); Anosy Region, District of Amboasary, 58 km SW of Fort Dauphin, 08 km NW of Amboasary, Berenty Special Reserve, -25.00667, 46.30333, 85 m, Galery forest (Rin’Ha, Mike) (CAS); Anosy Region, District of Amboasary, 58 km SW of Fort Dauphin, 08 km NW of Amboasary, Berenty Special Reserve, -25.00667, 46.30333, 85 m, Galery forest (Rin’Ha, Mike) (CAS); Anosy Region, District of Amboasary, PN Andohahela, Parcelle III, Ihazofotsy, 32 km NE Amboasary, -24.83083, 46.53617, 58 m, dry forest, spiny forest (Michael Irwin, Frank Parker, Rin’ha) (CAS); Anosy Region, District of Amboasary, 58 km SW of Fort Dauphin, 08 km NW of Amboasary, Berenty Special Reserve, -25.021, 46.3055, 36 m, spiny forest (Mike, Rin’ha) (CAS); Anosy Region, District of Fort-Dauphin, PN Andohahela, Parcelle II, Tsimela, 42 km W of Fort-Dauphin, -24.93683, 46.62667, 176 m, transition forest (Michael Irwin, Frank Parker, Rin’ha) (CAS); Anosy Region, PN Andohahela, Forêt de Manatalinjo, -24.82505, 46.57811, 90 m, spiny forest/thicket (B.L. Fisher, F.A. Esteves et al.) (CAS); Anosy Region, PN Andohahela, Forêt de Manatalinjo, -24.82466, 46.60111, 100 m, spiny forest/thicket (B.L. Fisher, F.A. Esteves et al.) (CAS); Atsimo Andrefana Region, District of Betioky, 30 km E Betioky, RS Beza Mahafaly (Around Research Station), -23.6865, 44.591, 165 m, Galery dry deciduous forest (Rin’Ha, Mike) (CAS); Atsimo Andrefana Region, District of Tulear II, Mikea deciduous dry forest 3 km N Andranomavo village, -22.90367, 43.4755, 30 m, Deciduous dry forest (Rin’Ha, Mike) (CAS); Beza-Mahafaly, 27 km E Betioky, -23.65, 44.63333, 135 m, tropical dry forest (B.L. Fisher) (CAS); Forêt de Beroboka, 5.9 km 131° SE Ankidranoka, -22.23306, 43.36633, 80 m, tropical dry forest (Fisher-Griswold Arthropod Team) (CAS); Forêt de Kirindy, 15.5 km 64° ENE Marofandilia, -20.045, 44.66222, 100 m, tropical dry forest (Fisher-Griswold Arthropod Team) (CAS); Forêt de Tsinjoriaky, 6.2 km 84° E Tsifota, -22.80222, 43.42067, 70 m, spiny forest/thicket (Fisher-Griswold Arthropod Team) (CAS); Makay Mts., -21.29961, 45.12919, 570 m, Dry forest edge and burned savannah (B.L. Fisher et al.) (CAS); Makay Mts., -21.34109, 45.18054, 500 m, Barren rock with sparse vegetation, burned grass (B.L. Fisher et al.) (CAS); Menabe Region, District of Morondava, Beroboka village 45 km NE of Morondava, Antsarongaza galery forest 07 km E of Beroboka, -19.9775, 44.66533, 45 m, Galery forest (M. Irwin, Rin’ha) (CAS); PN Andohahela, Forêt d’Ambohibory, 1.7 km 61° ENE Tsimelahy, 36.1 km 308° NW Tolagnaro, -24.93, 46.6455, 300 m, tropical dry forest (Fisher-Griswold Arthropod Team) (CAS); PN Andohahela, Forêt de Manatalinjo, 33.6 km 63° ENE Amboasary, 7.6 km 99° E Hazofotsy, -24.81694, 46.61, 150 m, spiny forest/thicket (Fisher-Griswold Arthropod Team) (CAS); PN Kirindy Mite, 16.3 km 127° SE Belo sur Mer, -20.79528, 44.147, 80 m, tropical dry forest (Fisher-Griswold Arthropod Team) (CAS); PN Tsimanampetsotsa, Forêt de Bemanateza, 20.7 km 81° E Efoetse, 23.0 km 131° SE Beheloka, -23.99222, 43.88067, 90 m, spiny forest/thicket (Fisher-Griswold Arthropod Team) (CAS); PN Tsimanampetsotsa, Mitoho Cave, 6.4 km 77° ENE Efoetse, 17.4 km 170° S Beheloka, -24.04722, 43.75317, 40 m, spiny forest/thicket (Fisher-Griswold Arthropod Team) (CAS); PN Zombitse, 19.8 km 84° E Sakaraha, -22.84333, 44.71, 770 m, tropical dry forest (Fisher, Griswold et al.) (CAS); RS Beza Mahafaly, Parcel 1, -23.65, 44.63333, 130 m, tropical dry forest (P.S. Ward) (CAS); southern Isoky-Vohimena Forest, 59 km NE Sakaraha, -22.46667, 44.85, 730 m, tropical dry forest (B.L. Fisher) (CAS); 3 km E Itampolo, malaise across path of lower bench of Andrimpano Forest, -24.65783, 43.95617, 45 m, dry forest (M.E. Irwin, Rin’ha) (CAS); 5 km E Itampolo, malaise across path of plateau of Andrimpano Forest, -24.65033, 43.96317, 130 m, dry forest (M.E. Irwin, Rin’ha) (CAS); 5 km N Ampotaka, malaise on trail in Vitambany gallery forest, -24.65033, 43.96317, 86 m, Gallery forest (M.E. Irwin, Rin’ha) (CAS); Berenty Special Reserve, 8 km NW Amboasary, 58 km SW of Fort Dauphin, -25.00667, 46.30333, 85 m, gallery forest (M.E. Irwin, F.D. Parker, R. Harin’Hala) (CAS); PN Andohahela, Ihazofotsy - Parcel III, transition forest, -24.83483, 46.48683, 80 m, tropical dry forest, transition between spiny and dry deciduous forests (M.E. Irwin, F.D. Parker, R. Harin’Hala) (CAS); Mikea Forest, deciduous dry forest, -22.90367, 43.4755, 30 m, deciduous dry forest (R. Harin’Hala) (CAS); Mikea Forest, spiny forest, -22.91333, 43.48222, 37 m, spiny forest (R. Harin’Hala) (CAS); near ANGAP office, PN Zombitse, -22.8865, 44.69217, 840 m, deciduous spiny forest (R. Harin’Hala) (CAS); Parcel I, RS Beza Mahafaly, near research station, -23.6865, 44.591, 165 m, dry deciduous forest (R. Harin’Hala) (CAS); Parcel II, RS Beza Mahafaly, near Bellevue, -23.68983, 44.5755, 180 m, spiny forest (R. Harin’Hala) (CAS); PN Tsimanampetsotsa, Mitoho Forest, malaise on plateau, -24.0485, 43.75233, 150 m, dense dry forest (M.E. Irwin, Rin’ha) (CAS); Tsimelahy - Parcel II, PN Andohahela, transition forest, -24.93683, 46.62667, 180 m, transition forest (M.E. Irwin, F.D. Parker, R. Harin’Hala) (CAS).

##### Diagnosis.

With head in full-face view, lateral cephalic margins anterior to eye level parallel and lacking erect hairs, lateral and anteromedian clypeal margin continuously forming broad convexity; scape covered with erect hairs; mesosoma short and high (MPH/ML: 0.39, 0.46); petiolar node flattened anteroposteriorly; body color yellowish to brown.

##### Description.

**Minor worker.** In full-face view, head sides anterior to level of eye parallel, converging progressively to posterior margin; eye large and convex (EL/CS: 0.33±0.01; 0.31–0.35), breaking lateral cephalic margin, level of its posterior border generally located from posterior 1/3 to 1/4 of head (PoOc/CL: 0.25±0.02; 0.22–0.29); frontal carinae not strongly diverging posteriorly (FR/CS: 0.28±0.01; 0.26–0.30), posteriorly diverging; clypeus with anterolateral angle, its anteromedian margin with blunt or convex angle, two apical teeth of mandible distantly spaced; antennal scape long (SL/CS: 1.49±0.05; 1.41–1.60). Dorsum of mesosoma strongly arched (MPH/ML: 0.42±0.02; 0.39–0.46), outline of promesonotum and dorsum of propodeum evenly convex; metanotal groove weakly visible, dorsal margin and declivity of propodeum connect at blunt angle; propodeal declivity height > 3/4 of dorsum length; petiolar node flattened anteroposteriorly, without noticeable dorsal margin; tibia of hind leg rounded axially and not twisted basally.

First and second gastral tergites without a pair of white spots; whole lateral margin of head covered with erect hairs; posterior margin of head with two pairs of erect hairs; antennal scape covered only with appressed hairs; posterodorsal angle of propodeum with more than four erect hairs.

**Major worker.** Characteristics the same as minor worker, except the enlarged head (CS: 2.41±0.22; 2.19–2.72; CWb/CL: 0.87±0.05; 0.83–0.94); stronger mandible; apical 1/5 of antennal scape surpassing posterior cephalic margin; metanotum visible; propodeal dorsum slightly convex joining a vertically built declivity at rounded angle; petiolar node much higher than long.

##### Distribution and biology.

**Figure 49.**
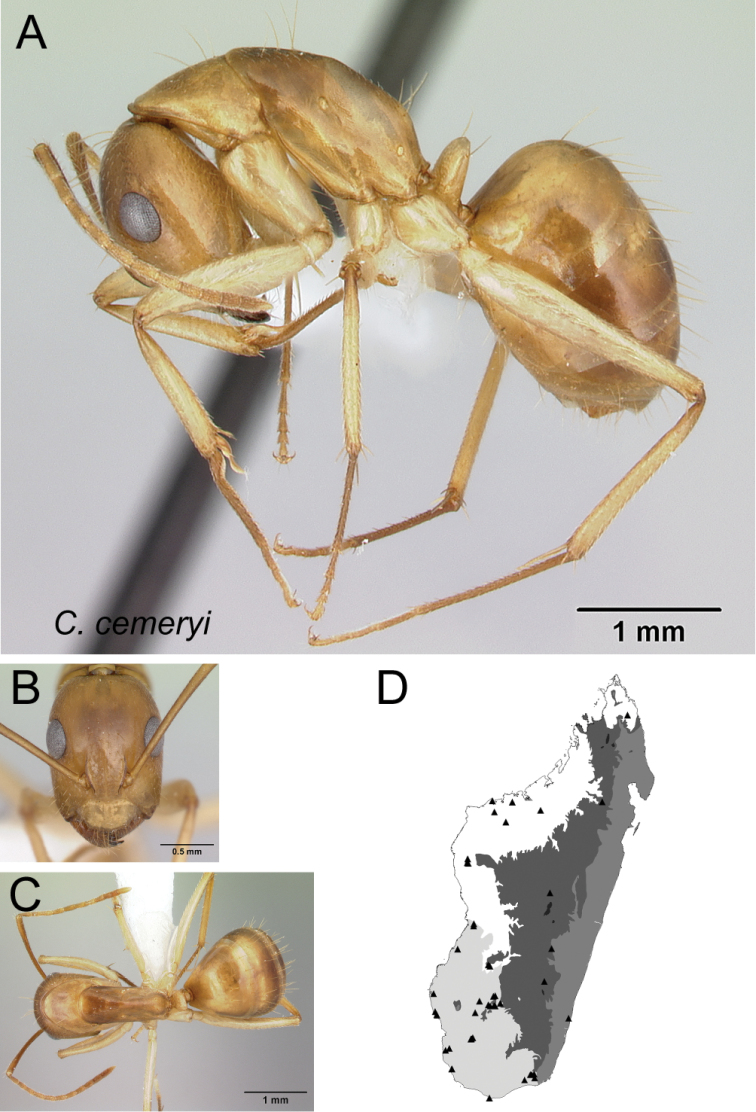
*Camponotuscemeryi***A** lateral view **B** head in full-face view **C** dorsal view of minor worker CASENT0217306**D** distribution map.

*Camponotuscemeryi* occurs in dry forest and spiny forest of west Madagascar and in the savannah shrubland and woodland of the central plateau (Fig. 49D). Along its distribution, members of the species can also be found in gallery forest habitats and in transitional forest between dry and humid forests. It may also establish colonies in human-modified habitats on the high plateau. Specimens have been collected mostly foraging on the forest floor and rarely on lower vegetation. The species nests in rotten logs, in the ground, under stones, and in rotting tree stumps.

##### Discussion.

Members of *C.cemeryi* can be separated from similar species like *C.boivini* by the strongly convex dorsal outline of its short and high mesosoma. It can be differentiated from *C.mahafaly* by the presence of erect to suberect hairs on the antennal scape.

Worker specimens that show the typical form of *C.cemeryi* are generally found across the western dry forest of Madagascar, but specimens have been collected from the mountaintops on the high plateau of the island that present morphological variation in which the lateral margin of head posterior to eye level is covered with erect hairs, three pairs of erect hairs are present on posterior cephalic margin, and body color is generally depigmented yellow or brown with depigmented yellow in some parts of the body. Based on these characters, this variant may constitute a separate species; however, the dendrogram based on quantitative morphological analysis did not support this hypothesis. The variant is nested within the cluster of the typical form of the species according to the NC-clustering method, and all of these samples were correctly classified by LDA at 100% success.

#### ﻿ Camponotus cervicalis

Taxon classificationAnimaliaHymenopteraFormicidae

Roger

A2F2DBE2-3BBE-5A79-804E-35012C841EF4

**Figure 50.**
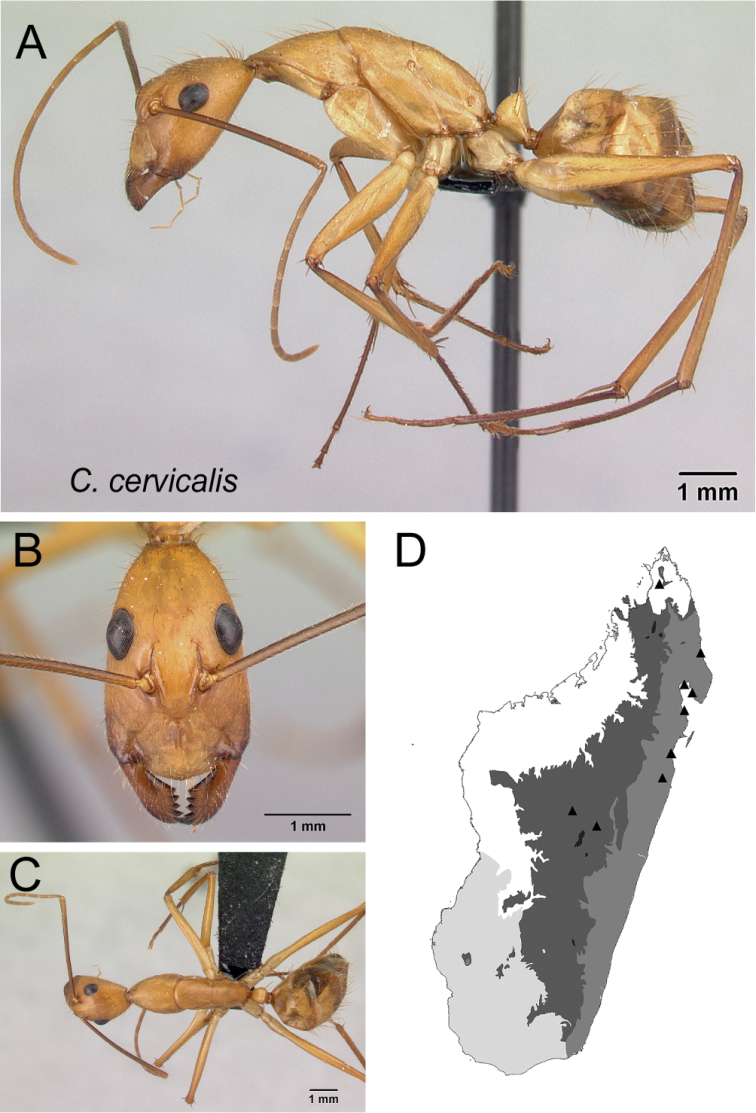
*Camponotuscervicalis***A** lateral view **B** head in full-face view **C** dorsal view of minor worker CASENT0217303**D** distribution map.

Figs 8A , 9B , 10B , 50

Camponotus cervicalis Roger, 1863: 134. Syntype workers, Madagascar (Humboldt) (NHMB); 1 syntype minor worker designated as lectotype, by present designation, AntWeb CASENT0101178 (NHMB) [examined]. Paralectotypes: 3 minor workers and 2 major workers of same data as lectotype but respectively specimen coded as: CASENT0101551, CASENT0101552 (MHNG), CASENT0104634 (ZMHB); CASENT0101704 and CASENT0101779 (MHNG) [examined]. Combination in Camponotus (Dinomyrmex): Forel, 1914: 268; in Camponotus (Tanaemyrmex): Emery, 1925: 84.
Camponotus gaullei Santschi, 1911a: 128. Syntype workers, Madagascar, S. de la Baie d’Antongil, (NHMB); 1 syntype minor worker designated as lectotype, by present designation, AntWeb CASENT0101179 (NHMB) [examined]. [Combination in Camponotus (Dinomyrmex): Wheeler, 1922: 1043; in Camponotus (Tanaemyrmex): Emery, 1925: 84]. As subspecies of Camponotuscervicalis by Emery, 1925: 84. Syn. nov.
Camponotus perroti Forel, 1897: 202. Syntype workers, Madagascar, Sainte Marie (Perrot) (MHNG); 1 syntype minor worker designated as lectotype, by present designation, AntWeb CASENT0101350 (MHNG) [examined]. Paralectotype major worker of same data as lectotype but with specimen code CASENT0101351 (MHNG) [examined]. Syn. nov.
Camponotus perroti aeschylus Forel, 1913: 224. Holotype (by monotypy) alate queen, Madagascar (C. Keller) AntWeb CASENT0101561 (MHNG) [examined]. Syn. nov.
Camponotus gerberti Donisthorpe, 1949: 271. Syntype workers, Madagascar, Sainte Marie (R. Géberti 1917) (NHMUK); 1 syntype minor worker designated as lectotype, by present designation, AntWeb CASENT0102310 (NHMUK) [examined]. Paralectotypes: 2 major workers of the same data as lectotype but with the following specimen codes: CASENT0102309, CASENT0102311 (NHMUK) [examined]. [Combination in Camponotus (Tanaemyrmex): Donisthorpe, 1949: 271]. Syn. nov.

##### Additional material examined.

**Madagascar: Antananarivo**: [Madagascar]; Ambatomanjaka; Miarinarivo, -18.766947, 46.869107, 1343 m (Humblot) (MNHN); Iharanandriana, -19.15823, 47.49702, 1513 m, *Uapaca* woodland (B.L. Fisher et al.) (CAS). **Antsiranana**: Forêt Ambanitaza, 26.1 km 347° Antalaha, -14.67933, 50.18367, 240 m, rainforest (B.L. Fisher) (CAS); RS Ankarana, 7 km SE Matsaborimanga, -12.9, 49.11667, 150 m, rainforest (P.S. Ward) (PSWC). **Toamasina**: S. de la baie d’Antongil. (NHMB); Mananara Avaratra, -16.170555, 49.765208, 18 m (B.L. Fisher et al.) (CAS); Nosy Mangabe, -15.5, 49.76667, <5 m, rainforest edge (P.S. Ward) (PSWC); Nosy Mangabe, 7.43 km S Maroantsetra, -15.4973, 49.76223, 3 m, littoral rainforest (B.L. Fisher et al.) (CAS); PN Masoala, 39.4 km 150° SSE Maroantsetra, -15.71, 49.97, 200 m, rainforest (B.L. Fisher & H.J. Ratsirarson) (CAS); Parcelle K7 Tampolo, -17.28333, 49.41667, 10 m, littoral forest (Malagasy ant team) (CAS); RNI Betampona, 34.1 km 332° Toamasina, -17.91614, 49.20185, 550 m, rainforest (B.L. Fisher et al.) (CAS); SF Tampolo, 10 km NNE Fenoarivo Atn. -17.2825, 49.43, 10 m, littoral rainforest (B.L. Fisher) (CAS); Tampolo, -17.28333, 49.41667, 10 m, littoral forest (Malagasy ant team) (CAS).

##### Diagnosis.

With head in full-face view, lateral cephalic margin converging posteriorly towards eye level, covered with erect hairs from anterior to posterior of eye level; anteromedian margin of clypeus broadly convex; two apical teeth of mandible normally spaced.

##### Description.

**Minor worker.** In full-face view, head sides converging progressively towards short posterior margin; eye protruding and large (EL/CS: 0.28±0.01; 0.26–0.30), breaking lateral cephalic margin, level of posterior margin located at posterior 1/3 of head (PoOc/CL: 0.30±0.01; 0.29–0.31); frontal carinae wide (FR/CS: 0.27±0.01; 0.26–0.28), distance between them larger than smallest distance to eye; clypeus with anterolateral angle and anteromedian margin with blunt angle or convex; mandible with two apical teeth distantly spaced; antennal scape relatively long (SL/CS: 1.81±0.12; 1.57–1.94). Promesonotum weakly convex, mesopropodeum almost flat, joining declivity with rounded angle; metanotal groove weakly visible; propodeal declivity 1/2 length of the dorsum. Petiolar node as long as high, with dorsal margin inclined posteriorly and rounding to anterior margin; anterior face 1/2 height of the posterior; femur of hind leg rounded axially, not twisted basally.

First and second gastral tergites without a pair of white spots; lateral margin of head with erect hairs; more than six erect hairs present near posterior margin of head; antennal scape covered with erect to suberect hairs inclined at ca. 45° as well as appressed hairs; pronotum covered with few erect hairs, mesonotum with a pair of hairs; posterodorsal angle of propodeum with two pairs of erect hairs.

**Major worker.** Characteristics the same as minor worker, except the enlarged head (CS: 4.17±0.35; 3.83–4.63; CWb/CL: 0.91±0.03; 0.85–0.93); the more strongly built mandible; apical 1/4 of antennal scape surpassing posterior cephalic margin; pronotum convex, mesonotum sloping towards metanotum, propodeal dorsum feebly convex and its junction to declivity broadly angulate; petiolar node much higher than long.

##### Distribution and biology.

*Camponotuscervicalis* is only known to occur in littoral forests and lower elevations of the rainforests in the east of Madagascar between the Ambanitaza forest in the north and the RNI Betampona in the south (Fig. 50D). Workers of the species have been found foraging on the ground and through leaf litter, but nest sites range from rotten logs, rotting tree stumps, rot pockets above the ground, live trees, under tree bark, and in moss and leaf litter.

##### Discussion.

*Camponotuscervicalis* and *C.niavo* look similar, but in the latter, the level of the posterior ocular margin is located at posterior 1/4 of length of head and its antennal scape lacks appressed hairs.

In his original description of *Camponotusgaullei*, Santschi (1911a) discussed the resemblance of the species to *C.cervicalis* and *C.dufouri* and no strong separating characters were provided. However, with recent extensive sampling of the Malagasy ant fauna, the observation of the range of worker class sizes in these latter species indicates the closer similarity between *C.cervicalis* and *C.gaullei*. Combined with the close distance between the type localities of both species, this information would be enough to synonymize *C.gaullei* under *C.cervicalis*.

The description of *C.perroti* by Forel (1897) was based on a limited number of worker specimens which had a close similarity to *C.cervicalis* and *C.dufouri*. Careful examinations of more specimens obtained from the recent ant surveys in Madagascar show that *C.perroti* and *C.cervicalis* belong to the same species and that *C.perroti* merits its placement as a junior synonym of *C.cervicalis*.

Forel’s description of *C.perrotiaeschylus* was based only on a single alate queen, and the comparison of this specimen with the queen specimens of *C.cervicalis* collected from its geographical range across Madagascar revealed that this type specimen was a queen of *C.cervicalis*.

Donisthorpe’s description of *Camponotusgerberti* did not compare the characters of the taxa to those of other species, although this description morphologically matches the type specimens of *C.cervicalis*. The data obtained from the recent ant survey in Madagascar and this information are sufficient to reasonably synonymize *C.gerberti* under *C.cervicalis*.

The identity of *C.cervicalis* based on conventional qualitative taxonomy is recognized by multivariate morphometric analysis. The cluster of the samples of the species created by NC-clustering method is confirmed by cumulative LDA with an identification success of 100%.

#### ﻿ Camponotus daraina sp. nov.

Taxon classificationAnimaliaHymenopteraFormicidae

65EBC1CA-8E0B-5A7B-BFA8-5AD02076EACA

http://zoobank.org/10EF7582-8BEC-438F-A757-BE96FF472CDB

Figs 14A , 51

##### Holotype worker.

**Madagascar**: Province **Antsiranana**: Forêt de Binara, 9.1 km 233° SW Daraina, -13.26333, 49.60333, 725 m, rainforest, ex rotten log, 03 Dec 2003 (B.L. Fisher et al.) collection code: BLF09678, specimen code: CASENT0077433 (CAS).

##### Paratypes.

1 major worker of same data as holotype but with specimen code: CASENT0809930 and 5 minor workers of collection code BLF09664 and the following specimen codes: CASENT0077671, CASENT0835653, CASENT0077673, CASENT0077670, CASENT0077673 (NHMUK, MHNG, MSNG, PBZT, CAS).

##### Additional material examined.

**Madagascar: Antsiranana**: Forêt Ambanitaza, 26.1 km 347° Antalaha, -14.67933, 50.18367, 240 m, rainforest (B.L. Fisher) (CAS); Forêt d’Antsahabe, 11.4 km 275° W Daraina, -13.21167, 49.55667, 550 m, tropical dry forest (B.L. Fisher et al.) (CAS); Forêt de Binara, 9.1 km 233° SW Daraina, -13.26333, 49.60333, 650–800 m, rainforest (B.L. Fisher et al.) (CAS); RS Manongarivo, 12.8 km 228° SW Antanambao, -13.97667, 48.42333, 780 m, rainforest (B.L. Fisher) (CAS); PN Montagne d’Ambre [1^st^ campsite], 960 m, rainforest (R. Harin’Hala) (CAS).

##### Diagnosis.

In full-face view, lateral cephalic margins converge posteriorly towards eye level, area posterior to eye level covered with erect hairs; two apical teeth of mandible closely spaced; body color reddish orange.

##### Description.

**Minor worker.** With head in full-face view, lateral borders gradually converge to anterior eye level, strongly converging behind eye level; eyes protruding and large (EL/CS: 0.28±0.01; 0.26–0.30), interrupting lateral cephalic border, level of its anterior margin located at posterior 1/3 of head (PoOc/CL: 0.29±0.01; 0.28–0.30); frontal carinae not widely opened posteriorly (FR/CS: 0.25±0.001; 0.24, 0.26), distance between them larger than smallest distance to eye; clypeus without well-defined anterolateral angle and with anteromedian margin broadly convex; mandible with two apical teeth closely placed; antennal scape relatively long (SL/CS: 1.72±0.06; 1.56–1.82). Promesonotum weakly convex, mesonotum posterior portion flat immediately anterior to weakly visible metanotal groove; propodeal dorsum almost straight, rounding to short declivity 1/2 length of propodeal dorsum. Petiolar node is short and high, with dorsal margin inclining posteriorly and forming a blunt angle with anterior face that has a height ca. 1/2 that of posterior face; femur of hind leg axially rounded, not twisted near base.

First and second gastral tergites without a pair of white spots; head covered with erect hairs on lateral margin, six erect hairs near its posterior margin; antennal scape covered with suberect hairs inclined ca. 30°; pronotum and anterior part of mesonotum covered with erect hairs; posterodorsal angle of propodeum with two pairs of erect hairs.

**Major worker.** Differing from minor worker in the following characters: enlarged head (CS: 13.54±0.25; 3.12–3.76; CWb/CL: 0.90±0.04; 0.85–0.94) with markedly concave posterior margin; apical 1/4 of antennal scape surpassing posterior margin of head; two apical teeth of mandible distantly spaced; robust mesosoma, with promesonotum forming even convexity, metanotum distinctly visible, propodeal dorsum slightly convex immediately behind metanotum, joining declivity surface at a blunt angle, < 2 × as long as declivity; petiolar node much higher and more compressed anteroposteriorly.

##### Distribution and biology.

**Figure 51.**
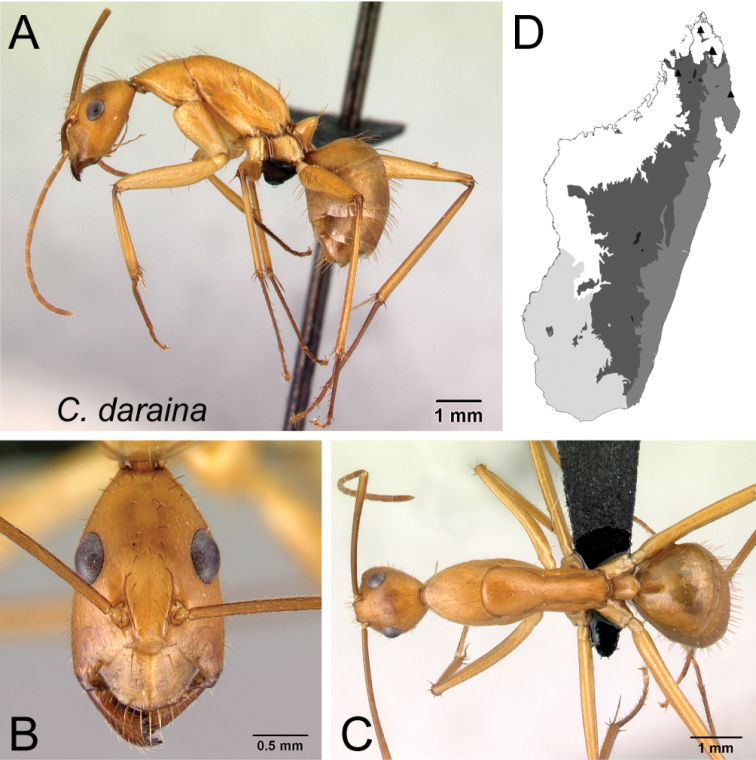
*Camponotusdaraina***A** lateral view **B** head in full-face view **C** dorsal view of holotype minor worker CASENT0077433**D** distribution map.

The distribution of *C.daraina* is generally limited to the northern and western slopes of Madagascar. The species occurs in dry forest habitats, transitional forests, and rainforests of the Daraina region, transitional forests of Montagne d’Ambre and the Ampasindava Peninsula, and rainforests of RS Manongarivo (Fig. 51D). *Camponotusdaraina* is strictly terrestrial; its workers forage mainly on the forest floor and through leaf litter, while its nests have been found in rotten logs and under stones.

##### Discussion.

*Camponotusdaraina* looks similar to *C.bemaheva* and *C.roeseli* but *C.bemaheva* lacks erect hairs from the lateral margin of the head posterior to the eye level and in *C.roeseli* the integument is either entirely reddish black or the head and the gaster are reddish black and the mesosoma is yellowish orange to reddish orange.

The identification of *C.daraina* based on conventional morphology-based taxonomy has been confirmed by multivariate morphometrics. The grouping of the samples of *C.daraina* produced by the NC-clustering method is corroborated by LDA with a classification success of 100%.

##### Etymology.

The species name *daraina* is a singular non-Latin noun used in apposition and refers to the type locality.

#### ﻿ Camponotus dufouri

Taxon classificationAnimaliaHymenopteraFormicidae

Forel

72DF19A0-7D4B-5F32-9064-ADB75E5CC5C3

Figs 8B , 12A , 52

Camponotus dufouri Forel, 1891: 16, 74 (Key). Syntype workers, Madagascar, 30 miles NW Tamatave (O’Swald) (MHNG) [examined]; 1 syntype major worker designated as lectotype, by present designation, Madagascar, AntWeb CASENT0101644 (MHNG) [examined]. Paralectoype: 1 major worker same data as lectotype but with specimen code: CASENT0101669 (MHNG) [examined]. [Combination in Camponotus (Dinomyrmex): Forel, 1914: 268; in Camponotus (Tanaemyrmex): Emery, 1925: 85].
Camponotus dufouri imerinensis Forel, 1891: 18. Syntype workers and queen, Madagascar, Environ d’Antananarivo, Imerina [labeled as “Andrangoloaka”] (Sikora) (MHNG) [examined]; 1 syntype minor worker designated as lectotype, by present designation, AntWeb CASENT0101812 (Sikora) (MHNG) [examined]. Paralectotypes: 1 major worker and 1 alate queen of the same data as lectotype but with the following specimen codes: CASENT0101919 (Sikora) and CASENT0101679 (Camboué) (MHNG) [examined]. Syn. nov.

##### Additional material examined.

**Madagascar: Antananarivo**: [Madagascar], Ambatomanjaka; Miarinarivo, -18.766947, 46.869107, 1343 m (Sicora) (CAS); 3 km 41 °NE Andranomay, 11.5 km 147° SSE Anjozorobe, -18.47333, 47.96, 1300 m, montane rainforest (Fisher, Griswold et al.) (CAS); Région Analamanga, SF Mandraka, -18.9183, 47.91687, 1285 m, montane rainforest (B.L. Fisher, F.A. Esteves et al.) (CAS); [Andrangoloaca]; Mantasoa; Manjakandriana, -19.033333, 47.9166666, 1409 m (MHNG). **Antsiranana**: 9.2 km WSW Befingotra, Réserve Anjanaharibe-Sud, -14.75, 49.46667, 1280 m, montane rainforest (B.L. Fisher) (CAS); 9.2 km WSW Befingotra, Réserve Anjanaharibe-Sud, -14.75, 49.46667, 1200 m, montane rainforest (B.L. Fisher) (CAS); Binara Forest, -13.26392, 49.59919, 1065 m, rainforest (B.L. Fisher et al.) (CAS); Binara Forest, -13.26388, 49.60141, 900 m, rainforest (B.L. Fisher et al.) (CAS); Forêt Ambanitaza, 26.1 km 347° Antalaha, -14.67933, 50.18367, 240 m, rainforest (B.L. Fisher) (CAS); Forêt de Binara, 9.1 km 233° SW Daraina, -13.26333, 49.60333, 650–800 m, rainforest (B.L. Fisher) (CAS); Forêt de Binara, 9.1 km 233° SW Daraina, -13.26333, 49.60333, 800 m, rainforest (B.L. Fisher) (CAS); Makirovana forest, -14.16044, 49.95216, 550 m, rainforest (B.L. Fisher et al.) (CAS); PN Masoala, -15.32331, 50.30751, 60 m, rainforest (B.L. Fisher et al.) (CAS); PN Montagne d’Ambre, Roussette Camp 7 km SW Park entrance, -12.51444, 49.18139, 960 m, rainforest (Mike, Frank, Rin’ha) (CAS); PN Marojejy, Antranohofa, 26.6 km 31° NNE Andapa, 10.7 km 318° NW Manantenina, -14.44333, 49.74333, 1325 m, montane rainforest (B.L. Fisher) (CAS); PN Marojejy, Manantenina River, 27.6 km 35° NE Andapa, 9.6 km 327 °NNW Manantenina, -14.435, 49.76, 775 m, rainforest (B.L. Fisher et al.) (CAS); PN Marojejy, Manantenina River, 28.0 km 38° NE Andapa, 8.2 km 333 °NNW Manantenina, -14.43667, 49.775, 450 m, rainforest (B.L. Fisher) (CAS); PN Montagne d’Ambre, 3.6 km 235° SW Joffreville, -12.53444, 49.1795, 925 m, montane rainforest (Fisher, Griswold et al.) (CAS); PN Montagne d’Ambre, Antomboka, -12.51269, 49.17807, 970 m, montane rainforest (B.L. Fisher et al.) (CAS); PN Montagne d’Ambre, Crête, -12.58132, 49.13368, 1110 m, montane rainforest (B.L. Fisher et al.) (CAS); PN Montagne d’Ambre, Petit lac, -12.53664, 49.17412, 1130 m, montane rainforest (B.L. Fisher et al.) (CAS); PN Montagne d’Ambre, Roussettes, -12.52574, 49.17238, 1025 m, montane rainforest (B.L. Fisher et al.) (CAS); R.N.I. Marojejy, 11 km NW Manantenina, -14.43333, 49.75, 1225 m, montane rainforest (E.L. Quinter) (CAS); RS Manongarivo, 10.8 km 229 °SW Antanambao, -13.96167, 48.43333, 400 m, rainforest (B.L. Fisher) (CAS); RS Manongarivo, 12.8 km 228° SW Antanambao, -13.97667, 48.42333, 780 m, rainforest (B.L. Fisher) (CAS); RS Manongarivo, 14.5 km 220° SW Antanambao, -13.99833, 48.42833, 1175 m, montane rainforest (B.L. Fisher) (CAS); PN Marojejy, 10 km NW Manantenina, -14.43333, 49.76667, 750 m, rainforest (E.L. Quinter) (CAS); PN Marojejy, 8 km NW Manantenina, -14.43333, 49.78333, 450 m, rainforest (E.L. Quinter) (CAS); Sakalava Beach [vegetated beach dunes], -12.26972, 49.39167, 10 m, across sandy trail in dwarf litoral forest (R. Harin’Hala) (CAS); SAVA Region, District of Sambava, PN Marojejy, 5 km W of Manantenina village, 1^st^ Camp site (Mantella), -14.43817, 49.774, 487 m, Low altitude rainforest (Rin’Ha, Mike) (CAS); Montaigne Francais, 150 m, along forested limestone ridge (R. Harin’Hala) (CAS); Montaigne Francais, 150 m, along forested limestone ridge (R. Harin’Hala) (CAS); PN Montagne d’Ambre [1^st^ campsite], 960 m, rainforest (Irwin, Schlinger, Harin’H) (CAS); PN Montagne d’Ambre [lemur trail], 975 m, rainforest (R. Harin’Hala) (CAS); PN Montagne d’Ambre [Petit Lac road], -12.533333, 49.166667, 1125 m, rainforest (Irwin, Schlinger, Harin’H) (CAS). **Fianarantsoa**: 40 km S Ambalavao, PN Andringitra, -22.21667, 46.96667, 1275 m, montane rainforest (B.L. Fisher) (CAS); 45 km S Ambalavao, -22.21667, 47.01667, 720 m, rainforest edge (B.L. Fisher) (MHNG); Andrambovato along river Tatamaly, -21.51082, 47.40992, 1063 m, rainforest (B.L. Fisher et al.) (CAS); Belle Vue trail, PN Ranomafana, -21.2665, 47.42017, 1020 m, mixed tropical forest (M.E. Irwin, F.D. Parker (R. Harin’Hala) (CAS); Vatovavy Fitovinany Region, District of Ifanadiana Belle vue area1.2 km S of PN Ranomafana entrance, -21.2665, 47.42017, 1018 m, rainforest (Rin’Ha, Mike) (CAS); Vatovavy Fitovinany Region, District of Ifanadiana, 12 km W of Ranomafana, -21.25083, 47.40717, 1127 m, forest edge, open area (Rin’Ha, Mike) (CAS); Forêt d’Ambalagoavy Nord, Ikongo, Ambatombe, -21.857068, 47.37849, 625 m (R. Harin’Hala & M.E. Irwin) (CAS); Forêt de Vevembe, 66.6 km 293° Farafangana, -22.791, 47.18183, 600 m, rainforest, transition to montane forest (B.L. Fisher et al.) (CAS); JIRAMA water works near river, PN Ranomafana, -21.2485, 47.45217, 690 m, open area near stream (R. Harin’Hala) (CAS); PN Ranomafana; Ranomafana; Ifanadiana, -21.2161494, 47.4565003, 1087 m (B. Pettersson) (CAS); PN Ranomafana, Vatoharanana River, 4.1 km 231° SW Ranomafana, -21.29, 47.43333, 1100 m, montane rainforest (Fisher, Griswold et al.) (CAS); PN Ranomafana, Talatakely, -21.24833, 47.42667 (CE Griswold, DH Kavanaugh, ND Penny, MJ Raherilalao, JS Ranorianarisoa, J Schweickert) (CAS); RS Ivohibe 8.0 km E Ivohibe, -22.48333, 46.96833, 1200 m, montane rainforest (B.L. Fisher, Sylvain) (CAS); RS Ivohibe, 7.5 km ENE Ivohibe, -22.47, 46.96, 900 m, rainforest (B.L. Fisher, Sylvain) (CAS); radio tower, PN Ranomafana, -21.25833, 47.40717, 1130 m, forest edge, mixed tropical forest, open area (M. Irwin, R. Harin’Hala) (CAS); PN Ranomafana, Talatakely area, 0.4 km WSW of Park Entrance, -21.41667, 47.68333, 900 m, mixed tropical forest (D.H. & K.M. Kavanaugh) (CAS); Réserve Speciale Manombo 24.5 km 228° Farafangana, -23.01583, 47.719, 30 m, rainforest (B.L. Fisher et al.) (CAS); Vohiparara broken bridge, -21.22617, 47.36983, 1110 m, high altitude rainforest (R. Harin’Hala) (CAS). **Toamasina**: [Bois 30 miles NW. Tamatave], Ambodiriana; Toamasina Rural, -17.88832, 49.22849, 373 m (Swald) (CAS); [Tamatave?], Andranobolahy, Toamasina Rural, -18, 49, 370 m (H. O’Swald) (CAS); 5.3 km SSE Ambanizana, Andranobe, -15.66667, 49.96667, 600 m, rainforest (B.L. Fisher) (MHNG); 5.3 km SSE Ambanizana, Andranobe, -15.67133, 49.97395, 425 m, rainforest (B.L. Fisher) (MHNG); 6.2 km SSE Ambanizana, Be Dinta, -15.66667, 49.99806, 600 m, rainforest (V. Razafimahatratra.) (MNHN); 6.3 km S Ambanizana, Andranobe, -15.6813, 49.958, 25 m, rainforest (B.L. Fisher) (MNHN); 6.9 km NE Ambanizana, Ambohitsitondroina, -15.58506, 50.00952, 825 m, rainforest (B.L. Fisher) (MNHN); 7 km SE PN Andasibe Headquarters, -18.969856, 48.465894, 1050 m, tropical forest (M.E. Irwin R. Harin’Hala) (CAS); Analanjirofo Region, District of ToamasinA Mobot Site, Analalava humid dense forest low altitude on the sand 7 km SW of Foulpointe, -17.69333, 49.46028, 75 m, dense humide low alt on the sandy soil (Mike, Rin’ha) (CAS); PN Andasibe, botanic garden near entrance, West of ANGAP office, -18.925172, 48.418651, 1025 m, tropical forest (M.E. Irwin R. Harin’Hala) (CAS); Ankerana, -18.40829, 48.82107, 750 m, rainforest (B.L. Fisher et al.) (CAS); Antsianaka; Ambatosoratra; Ambatondrazaka, -17.58333, 48.5, 752 m (Perrot Freres) (CAS); Corridor Forestier Analamay-Mantadia, Ambatoharanana, -18.80398, 48.40358, 1064 m, rainforest (B.L. Fisher et al.) (CAS); Corridor Forestier Analamay-Mantadia, Ambatoharanana, -18.79944, 48.40375, 1016 m, rainforest (B.L. Fisher et al.) (CAS); Corridor Forestier Analamay-Mantadia, Tsaravoniana, -18.76124, 48.42134, 939 m, rainforest (B.L. Fisher et al.) (CAS); FC Andriantantely, -18.695, 48.81333, 530 m, rainforest (H.J. Ratsirarson) (CAS); FC Didy, -18.19833, 48.57833, 960 m, rainforest (H.J. Ratsirarson) (CAS); FC Sandranantitra, -18.04833, 49.09167, 450 m, rainforest (H.J. Ratsirarson) (CAS); Forêt Ambatovy, 14.3 km 57° Moramanga, -18.85083, 48.32, 1075 m, montane rainforest (Malagasy ant team) (CAS); Ile Sainte Marie, Forêt Ampanihy, 14.4 km 52° Ambodifotatra, -16.91117, 49.93917, 10 m, littoral rainforest (B.L. Fisher et al.) (CAS); Mahavelona (Foulpointe), -17.66667, 49.5 in sandy forest (A. Pauly) (CAS); Manakambahiny Atsinanana, -17.75, 48.71667 Primary forest (A. Pauly) (CAS); Montagne d’Akirindro 7.6 km 341° NNW Ambinanitelo, -15.28833, 49.54833, 600 m, rainforest (Fisher, Griswold et al.) (CAS); Montagne d’Anjanaharibe, 18.0 km 21° NNE Ambinanitelo, -15.18833, 49.615, 470 m, rainforest (Fisher, Griswold et al.) (CAS); Montagne d’Anjanaharibe, 19.5 km 27° NNE Ambinanitelo, -15.17833, 49.635, 1100 m, montane rainforest (Fisher, Griswold et al.) (CAS); PN Mantadia, -18.79167, 48.42667, 895 m, rainforest (H.J. Ratsirarson) (CAS); PN Zahamena, Besaky River, -17.75244, 48.85321, 760 m, rainforest (B.L. Fisher et al.) (CAS); PN Zahamena, Onibe River, -17.75908, 48.85468, 780 m, rainforest (B.L. Fisher et al.) (CAS); PN Mananara-Nord, 7.1 km 261° Antanambe, -16.455, 49.7875, 225 m, rainforest (B.L. Fisher et al.) (CAS); Parcelle K9 Tampolo, -17.175, 49.268, 10 m, littoral forest (Malagasy ant team) (CAS); RNI Betampona, Camp Vohitsivalana, 37.1 km 338° -17.88667, 49.2025, 520 m, rainforest (B.L. Fisher et al.) (CAS); RNI Betampona, 34.08 km 332° Toamasina -17.91977, 49.20039, 525 m, rainforest (B.L. Fisher) (CAS); RNI Betampona, 34.1 km 332° Toamasina -17.91614, 49.20185, 550 m, rainforest (B.L. Fisher) (CAS); SF Tampolo, 10 km NNE Fenoarivo Atn. -17.2825, 49.43, 10 m, littoral rainforest (B.L. Fisher) (CAS); Sahafina forest 11.4 km W Brickaville, -18.81445, 48.96205, 140 m, rainforest (B.L. Fisher et al.) (CAS); Tamatave; Andranobolahy; Toamasina Rural, -18, 49, 370 m (CAS). **Toliara**: 10 km NW Enakara, PN Andohahela, -24.56667, 46.81667, 420 m, rainforest (B.L. Fisher) (PSWC); 13 km NW Enakara, PN Andohahela, -24.55, 46.8, 1250 m, montane rainforest (B.L. Fisher) (CAS); Anosy Region, Anosyenne Mts, 29.33 km NW Manantenina, -24.13993, 47.07418, 540 m, montane rainforest (B.L. Fisher, F.A. Esteves et al.) (CAS); Anosy Region, PN Andohahela, Col de Tanatana, -24.7585, 46.85367, 275 m, rainforest (B.L. Fisher, F.A. Esteves et al.) (CAS); Forêt Mandena 8.5 km N Tolagnaro, -24.95267, 47.0025, 20 m, littoral rainforest (B.L. Fisher et al.) (CAS); Isaka-Ivondro; Taolagnaro, -24.8, 46.866667, 41 m (Remy) (CAS); Ivohibe; Mahabo; Betroka, -23.62778, 46.48861, 1552 m (R. Decary) (CAS); Kirindy Forest, plot I, -20.07354, 44.67194, 60 m, dry forest (E. Lokensgard) (CAS); PN Andohahela, Col du Sedro, 3.8 km 113° ESE Mahamavo, 37.6 km 341° NNW Tolagnaro, -24.76389, 46.75167, 900 m, montane rainforest (Fisher-Griswold Arthropod Team) (CAS); PN Andohahela, Manampanihy River, 5.4 km 113° ESE Mahamavo, 36.7 km 343° NNW Tolagnaro, -24.76389, 46.76683, 650 m, rainforest (Fisher-Griswold Arthropod Team) (CAS); Mikea Forest, deciduous dry forest, -22.90367, 43.4755, 30 m, deciduous dry forest (R. Harin’Hala) (CAS); Mikea Forest, spiny forest, -22.91333, 43.48222, 37 m, spiny forest (R. Harin’Hala) (CAS); Tsimelahy-Parcel II, PN Andohahela, transition forest, -24.93683, 46.62667, 180 m, transition forest (M.E. Irwin, F.D. Parker, R. Harin’Hala) (CAS).

##### Diagnosis.

With head in full-face view, lateral cephalic margins converge posteriorly towards eye level, strongly converging from posterior ocular margin to occipital corner of head; anteromedian margin of clypeus straight; two apical teeth of mandible normally spaced; lateral cephalic margin anterior to eye level covered with erect hairs.

##### Description.

**Minor worker.** In full-face view, lateral cephalic margins converging progressively towards posterior margin or slightly converging to level of anterior ocular margin and strongly converging posteriorly; eye large and convex (EL/CS: 0.28±0.02; 0.25–0.31), breaking lateral cephalic border, located at midlength of head, level of its posterior margin within the posterior 1/3 of head (PoOc/CL; 0.33±0.01; 0.31–0.36); frontal carinae more or less wide, posteriorly parallel (FR/CS: 0.25±0.01; 0.24–0.27), their distance larger than smallest distance to eye; clypeus with anterolateral angle and medially slightly concave anteromedian margin; two apical teeth of mandible distantly spaced; antennal scape relatively long (SL/CS: 1.93±0.08; 1.76–2.12). Mesosoma long and low (MPH/ML: 0.29±0.01; 0.27–0.32), with slightly convex promesonotum, nearly flat mesopropodeum, and feebly visible metanotal groove; junction of propodeal dorsum to declivity bluntly angulate; propodeal declivity 1/3 length of dorsum. Petiole nodiform, tapering dorsally; its dorsal margin inclined posteriorly and forming a blunt angle to anterior face; posterior face 3 × as high as the anterior; femur of hind leg rounded axially, not twisted basally.

First and second gastral tergites without a pair of white spots; lateral margin of head covered with erect hairs; near posterior margin of head with more than six erect hairs; antennal scape covered with erect hairs inclined at ca. 45°; pronotum with numerous erect to suberect hairs; posterodorsal angle of propodeum with a pair of erect hairs.

**Major worker.** With characteristics of minor worker except for the following divergent characters: larger head (CS: 3.93±0.51; 3.46–4.54; CWb/CL: 0.88±0.08; 0.80–0.97), with concavity on posterior margin; anteromedian margin of clypeus slightly concave; apical 1/3 of antennal scape surpassing posterior cephalic margin; promesonotum and propodeum in separate convexity; mesonotum and propodeal dorsum 2 × as long as declivity, with blunt angle junction.

##### Distribution and biology.

**Figure 52.**
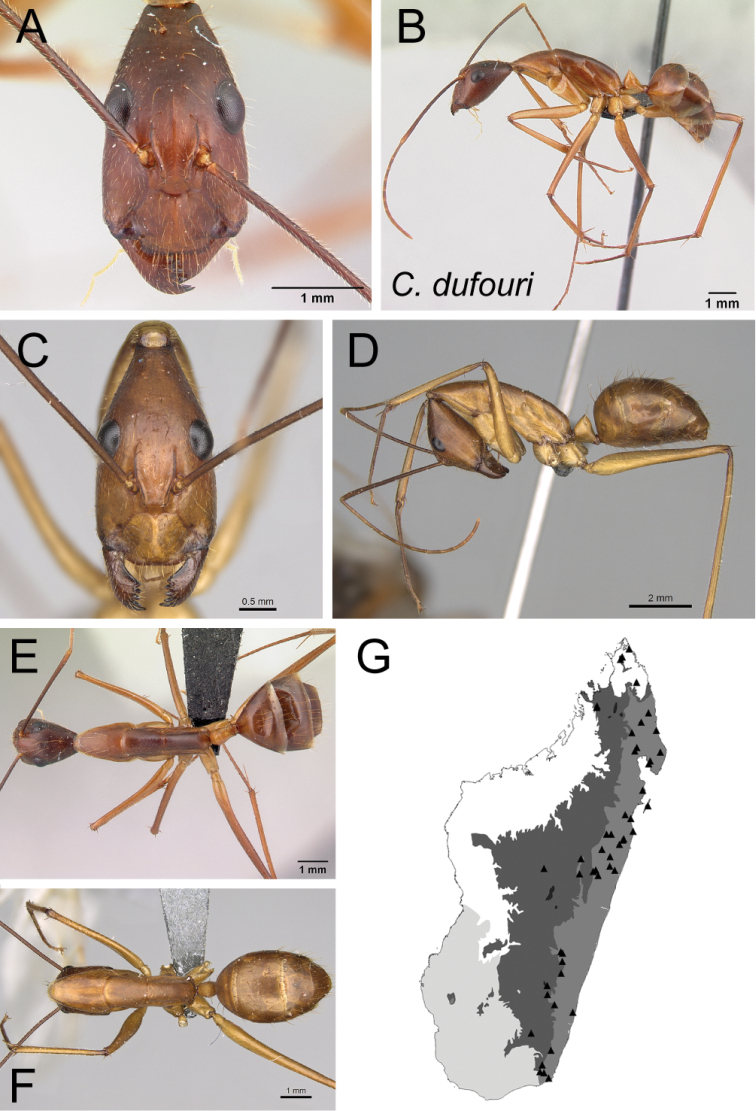
*Camponotusdufouri***A, C** head in full-face view **B, D** lateral view **E, F** dorsal view of minor workers CASENT0060837 and CASENT0274127**G** distribution map.

*Camponotusdufouri* is only known from Madagascar. Its distribution follows that of eastern humid forests ranging from the littoral to the montane rainforest of the island (Fig. 52G). Its colony nests have been found mostly in rotten logs, rotten sticks, and rotting tree stumps, and rarely in dead branches, twigs, and bamboo on the ground. Workers forage frequently on the ground and in leaf litter, rarely on low vegetation.

##### Discussion.

A clypeus with a straight anteromedian margin makes *C.dufouri* easy to separate from *C.lokobe*. Also, in *C.tendryi*, when the head is set in full-face view, the lateral cephalic margin posterior to the eye level is without erect hairs and its body size is smaller (CS: 1.57±0.01; 1.56–1.58), while in *C.dufouri*, erect hairs are present on the lateral cephalic margin posterior to the eye level and its body size is larger (CS: 1.97±0.19; 1.66–2.26).

A verification of the syntype workers of *C.dufouriimerinensis* demonstrates that they were indistinguishable from the syntypes of *C.dufouri*. Moreover, since the geographic distribution of *C.dufouri* is ranging from the littoral to the montane rainforests of Madagascar where the type specimens of the subspecies name were collected, it is consequently correct to synonymize it under *C.dufouri*.

The qualitative morphology-based study of *C.dufouri* agrees with the multivariate statistical analysis and both methods support the taxonomic delimitation for this species. The validity of the species, supported by the grouping obtained from NC-clustering, is confirmed by LDA with an identification success of 100%.

#### ﻿ Camponotus gibber

Taxon classificationAnimaliaHymenopteraFormicidae

Forel

82521528-F8E1-56E9-A714-4B00288E1701

**Figure 53.**
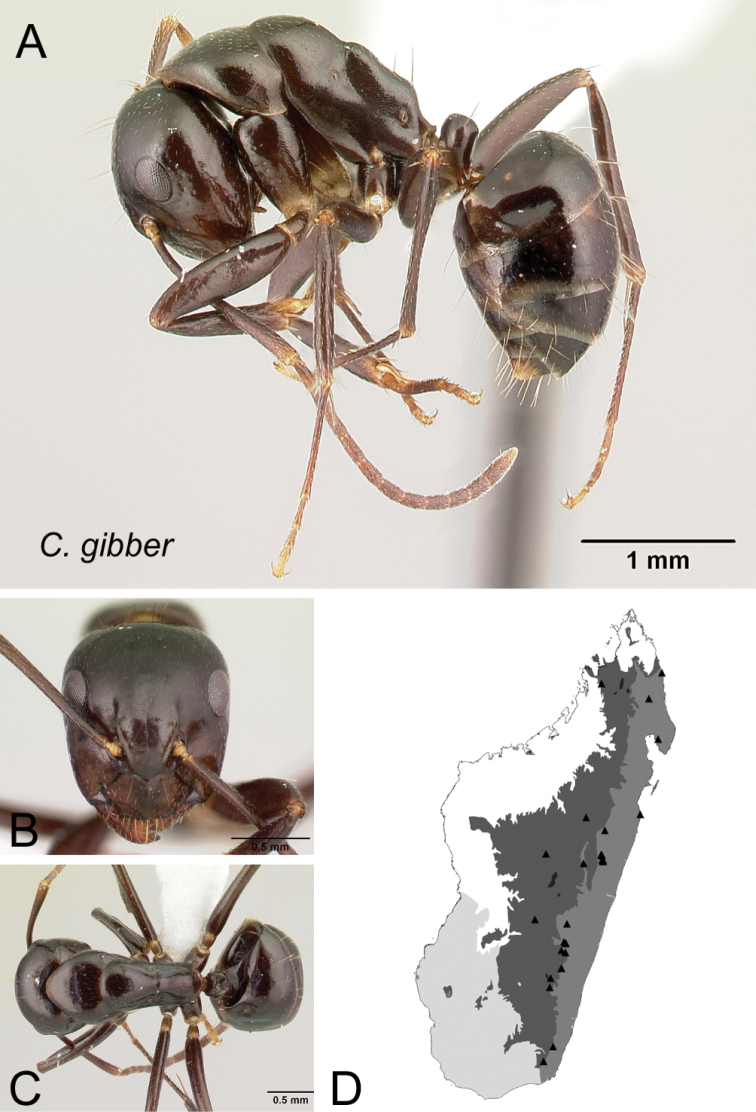
*Camponotusgibber***A** lateral view **B** head in full-face view **C** dorsal view of minor worker CASENT0188619**D** distribution map.

Figs 32B , 35C , 38A , 53

Camponotus quadrimaculatus var. gibber Forel, 1891: 59. Lectotype minor worker, by present designation, Madagascar, Andrangoloaka (Sikora) AntWeb CASENT0101513 (MHNG) [examined]. Paralectotype. 1 minor worker and 1 alate queen of same data as lectotype but with the following specimen codes: CASENT0101528, CASENT0101537 (MHNG) [examined]. Raised to species by Forel 1891: 215; 1892: 232; Dalla Torre 1893: 232; Emery 1896: 374; Wheeler 1922: 1054; Emery 1925: 122; Bolton 1995: 101. [Combination in Camponotus (Myrmosphincta) Forel, 1914: 273; in Camponotus (Mayria) Emery, 1925: 122].

##### Additional material examined.

**Madagascar: Antananarivo**: [(de Diversa); Museum Paris, Grandidier 1899]; Mantasoa; Manjakandriana, -19.033333, 47.9166666, 1409 m (CAS); [Madagascar central]; Ambatomanjaka; Miarinarivo, -18.766947, 46.869107, 1343 m (CAS). **Antsiranana**: Ambondrobe, 41.1 km 175° NW Vohemar, -13.71533, 50.10167, 10 m, littoral rainforest (B.L. Fisher) (CAS); RS Manongarivo 17.3 km 218° SW Antanambao, -14.02167, 48.41833, 1580 m, montane rainforest (B.L. Fisher) (CAS); RS Manongarivo, 14.5 km 220° SW Antanambao, -13.99833, 48.42833, 1175 m, montane rainforest (B.L. Fisher) (CAS); RS Manongarivo, 14.5 km 220° SW Antanambao, -14.00, 48.43167, 1220 m, montane rainforest (B.L. Fisher) (CAS); PN Marojejy, Antranohofa, 26.6 km 31° NNE Andapa, 10.7 km 318° NW Manantenina, -14.44333, 49.74333, 1325 m, montane rainforest (B.L. Fisher) (CAS); RS Manongarivo, 17.3 km 218° SW Antanambao, -14.02167, 48.41833, 1600 m, montane rainforest (B.L. Fisher) (CAS); PN Marojejy, 11 km NW Manantenina, -14.45, 49.73333, 1875 m, montane rainforest (E.L. Quinter) (CAS). **Fianarantsoa**: [Hte Sahandrata; Forét prim. de Tsianovoha]; Ambohimitombo; Ambositra, -20.72, 47.45, 1172 m (P.S. Ward) (PSWC): 2 km W Andrambovato, along river Tatamaly, -21.51167, 47.41, 1075 m, montane rainforest (B.L. Fisher et al.) (CAS); 3 km W Ranomafana, nr. Ifandiana, -21.25, 47.41667, 950 m, rainforest (P.S. Ward) (CAS); 43 km S Ambalavao, PN Andringitra, -22.23333, 47, 825 m, rainforest (B.L. Fisher) (CAS); Forêt d’Atsirakambiaty, 7.6 km 285° WNW Itremo, -20.59333, 46.56333, 1550 m, montane rainforest (Fisher, Griswold et al.) (CAS); Ambinanindranomena Non Protected Area, 39.16 km SE Ambalavao, -21.96007, 47.29125, 1002 m, Savannah grassland (A. Ravelomanana) (CAS); Ambinanindranomena Non Protected Area, 39.45 km SE Ambalavao, -21.95386, 47.29427, 1069 m, montane rainforest (A. Ravelomanana) (CAS); Ampanenitra Non Protected Area, 41.19 km SE Ambalavao, -21.9652, 47.31001, 1010 m, Savannah grassland (A. Ravelomanana) (CAS); Andrambovato along river Tatamaly, -21.50967, 47.40762, 984 m, cultivated land (tavy) (B.L. Fisher et al.) (CAS); Belle Vue trail, PN Ranomafana, -21.2665, 47.42017, 1020 m, mixed tropical forest (R. Harin’Hala) (CAS); Vatovavy Fitovinany Region, District of Ifanadiana Belle vue area1.2 km S of PN Ranomafana entrance, -21.2665, 47.42017, 1018 m, rainforest (Rin’Ha, Mike) (CAS); Vatovavy Fitovinany Region, District of Ifanadiana Belle vue area1.2 km S of PN Ranomafana entrance, -21.2665, 47.42017, 1018 m, rainforest (Rin’Ha, Mike) (CAS); Vatovavy Fitovinany Region, District of Ifanadiana, 12 km W of Ranomafana, -21.25083, 47.40717, 1127 m, forest edge, open area (Rin’Ha, Mike) (CAS); JIRAMA water works near river, PN Ranomafana, -21.2485, 47.45217, 690 m, open area near stream (R. Harin’Hala) (CAS); Namorona River at footbridge, PN Ranomafana, -21.25833, 47.42178, 850 m, mixed tropical forest near river, ME Irwin & EI Schlinger (CAS); PN Ranomafana, Sahamalaotra River, 6.6 km 310° NW Ranomafana, -21.23667, 47.39667, 1150 m, montane rainforest (Fisher, Griswold et al.) (CAS); PN Ranomafana, Vatoharanana River, 4.1 km 231° SW Ranomafana, -21.29, 47.43333, 1100 m, montane rainforest (Fisher, Griswold et al.) (CAS); PN Ranomafana, Talatakely, -21.24833, 47.42667, in bamboo forest (CE Griswold, DH Kavanaugh, ND Penny, MJ Raherilalao, JS Ranorianarisoa, J Schweickert) (CAS); RS Ivohibe 8.0 km E Ivohibe, -22.48333, 46.96833, 1200 m, montane rainforest (B.L. Fisher, Sylvain) (CAS); radio tower, PN Ranomafana, -21.25833, 47.40717, 1130 m, forest edge, mixed tropical forest, open area (M.E. Irwin, F.D. Parker, R. Harin’Hala) (CAS); Ranomafana, -21.25, 47.36667 (A. Pauly) (CAS); Ranomafana Nat. Park, Talatakely forest; Sahambavy; Fianarantsoa Rural, -21.4511792, 47.3023894, 1139 m (V.F. Lee, K.J. Ribardo,) (CAS); PN Ranomafana, Talatakely area, 0.4 km WSW of Park Entrance, -21.41667, 47.68333, 900 m, mixed tropical forest (D.H. & K.M. Kavanaugh) (CAS); Vohiparara, -21.23333, 47.36667 (A. Pauly) (CAS); Vohiparara broken bridge, -21.22617, 47.36983, 1110 m, high altitude rainforest (R. Harin’Hala) (CAS); 23 km E Moramanga, -18.98028, 48.45306, 900 m, tropical dry forest (B.L. Fisher) (CAS). **Toamasina**: 6.9 km NE Ambanizana, Ambohitsitondroina, -15.56667, 50, 1080 m, montane rainforest (B.L. Fisher) (CAS); 6 km ESE Andasibe (= Perinet), -18.95, 48.46667, 900 m, rainforest (P.S. Ward) (CAS); Bevolota 17.1 km N Andasibe, -18.77071, 48.43164, 995 m, montane rainforest (B.L. Fisher et al.) (CAS); Corridor Forestier Analamay-Mantadia, Ambatoharanana, -18.80424, 48.40081, 968 m, rainforest (B.L. Fisher et al.) (CAS); Corridor Forestier Analamay-Mantadia, Ambatoharanana, -18.79956, 48.4028, 1058 m, rainforest (B.L. Fisher et al.) (CAS); forêt Didy, Ambatondrazaka, -18.1111503, 48.5107283, 1029 m, rainforest (A. Pauly) (CAS); Mahavelona (Foulpointe), -17.66667, 49.5, Pandanus marsh (A. Pauly) (CAS); Morarano-Chrome, 25 km W forét I, -17.75, 47.98333 (A. Pauly) (CAS); PN Mantadia, -18.79167, 48.42667, 895 m, rainforest (H.J. Ratsirarson) (CAS); PN Andasibe-Mantadia, Forêt de Mantadia, 25.7 km 248° Moramanga, -18.81402, 48.43028, 1040 m, rainforest (F.N. Raharimalala, B. Blaimer) (CAS); Réserve Perinet-Analamazaotra, -18.93333, 48.43333, 950 m, rainforest (D.M. Olson) (CAS). **Toliara**: 13 km NW Enkara, PN Andohahela, -24.55, 46.8, 1160 m, montane rainforest (B.L. Fisher) (CAS); 13 km NW Enkara, PN Andohahela, -24.56667, 46.81667, 850 m, rainforest (B.L. Fisher) (CAS); Anosy Region, Anosyenne Mts, 31.2 km NW Manantenina, -24.13894, 47.06804, 1125 m, rainforest (B.L. Fisher, F.A. Esteves et al.) (CAS); Forêt Ivohibe 55.6 km N Tolagnaro, -24.56167, 47.20017, 650 m, rainforest (B.L. Fisher et al.) (CAS); Ifaty 22 km N, -23.18333, 43.61667, 30 m, beach dunes (M.E. Irwin and E.I. Schlinger) (CAS); near road, PN Zombitse, -22.8405, 44.73117, 825 m, spiny deciduous forest (R. Harin’Hala) (CAS).

##### Diagnosis.

In full-face view, lateral margins of head anterior to eye level diverging posteriorly; anterior clypeal margin truncate; two pairs of white spots present on second and third abdominal tergites; pronotum, mesonotum, and propodeum forming separate convexities, metanotal groove depressed; level of propodeum lower than that of promesonotum.

##### Description.

**Minor worker.** In full-face view, head sides diverging towards broadly convex posterior margin; eye slightly protruding and small (EL/CS: 0.27±0.01; 0.24–0.30), not breaking lateral cephalic margin, level of its posterior margin located at ca. posterior 1/4 of head (PoOc/CL: 0.25±0.01; 0.23–0.28); frontal carinae wide and diverging posteriorly (FR/CS: 0.35±0.01; 0.33–0.36), distance between them larger than their smallest distance to eye; clypeus with anterolateral angle and straight anteromedian margin; mandible with two apical teeth distant from each other; antennal scape relatively long (SL/CS: 1.17±0.07; 0.93–1.27). Pronotum and mesonotum forming a separate convexity; propodeal dorsum convex anteriorly, concave medially, then flat posteriorly, joining declivity in noticeable angle; metanotal groove weakly visible; propodeal declivity 3/4 length of dorsum. Petiolar node short and high, with dorsal margin straight then rounding to both anterior and posterior faces; anterior face almost 2/3 height of posterior face; femur of hind leg rounded axially, not twisted basally.

First and second gastral tergites with a pair of white spots; lateral margin of head without erect hairs; three pairs of erect hairs present near posterior margin of head; antennal scape only covered with appressed hairs; pronotum with few erect hairs; mesonotum with a pair of erect hairs; posterodorsal corner of propodeum with two pairs of erect hairs. Body color shining brown to dark brown; apical section of appendages lighter in color.

**Major worker.** With characteristics of minor worker except: enlarged head (CS: 2.45±0.21; 2.21–2.82; CWb/CL: 1.05±0.04; 1.01–1.10) with broadly concave posterior margin; anteromedian clypeal margin noticeably excised medially; antennal scape hardly extending beyond posterior cephalic margin; robust mesosoma, pronotum, and mesonotum an even convexity, metanotum distinct, propodeal dorsum sloping straight to declivity, approximately the same length as declivity; petiolar node more flattened anteroposteriorly.

##### Distribution and biology.

*Camponotusgibber* occurs in mid-altitude rainforest, montane rainforest, open areas on the forest edge, and the savannah grassland of the high plateau of Madagascar. Its distribution ranges from the RS Manongarivo and PN Marojejy in the north through the Corridor Forestier Analamay-Mantadia and PN Andringitra in the south-central region to the PN Andohahela and Anosyenne Mountains in the south. It has also colonized the littoral rainforest of Ambondrobe Vohemar, the dry forest of the PN Isalo, and cultivated land in the Andrambovato Forest (Fig. 53D). Individual workers forage mostly on the ground and through leaf litter, and rarely on lower vegetation. Nests are typically in rotten logs and rotting tree stumps, but seldom in the ground, in root mats on the ground, in dead twigs above the ground, in moss and leaf litter on live trees, and under tree bark.

##### Discussion.

*Camponotusgibber* may be difficult to differentiate from *C.rotrae* and *C.quadrimaculatus* in that they have two pairs of white spots on the second and third abdominal tergites. In both latter species, however, the pronotum, mesonotum, and propodeum do not form separate convexities, the metanotal groove is not depressed, and the dorsal face of its petiolar node joins the posterior face at an angle.

There are apparently three forms within *C.gibber*. These are geographically isolated across their distribution along the eastern rainforest of Madagascar due to the presence of high mountain chains in northwestern Madagascar. In the first form, the mesosoma strongly forms separate convexities and the propodeal dorsum is more or less straight. The second form is characterized by a more or less continuous dorsal outline of the mesosoma and a broadly concave propodeal dorsum. The third form constitutes intermediate degrees of these phenotypic variations because workers present separate convexities of mesosoma and a slightly concave propodeal dorsum. The members of the first two forms show morphological variabilities that gradually merge in the third form.

The relatively low 91.89% classification success attained by LDA is due to the misclassification of three minor workers as *C.quadrimaculatus*. This is because the third variant in *C.gibber* and members of *C.quadrimaculatus* species share qualitative morphological traits, and both species display overlapping ranges of quantitative measurements. The grouping of *C.gibber* in the same cluster shown by the dendrogram of multivariate morphometric analysis corroborates the species hypothesized by the taxonomic revision based on qualitative morphology.

#### ﻿ Camponotus gouldi

Taxon classificationAnimaliaHymenopteraFormicidae

Forel

55285B42-4834-5A52-ABFC-0C54A41D1528

Figs 31A , 54 , 55

Camponotus egregius r. gouldi Forel, 1886a: iv. Holotype (by monotypy) major worker, Madagascar (Grandidier) [not examined, type not found]. Combination in Camponotus (Myrmogigas): Forel, 1912: 91; in Camponotus (Dinomyrmex): Forel, 1914: 268; in Camponotus (Tanaemyrmex): Emery, 1925: 85. Raised to species: Dalla Torre 1893: 233; Forel 1897: 201; Forel 1904: 377.

##### Neotype major worker, by present designation.

**Madagascar**: Province **Mahajanga**: PN Ankarafantsika, Forêt de Tsimaloto, 18.3 km 46° NE de Tsaramandroso, -16.22806, 47.14361, 135 m, tropical dry forest, ground nest, 2–8 Apr 2001 (Fisher, Griswold & Malagasy Arthropod Team) collection code: BLF03536, specimen code: CASENT0437531 (CAS).

##### Additional material examined.

**Madagascar: Antananarivo**: Analamanga Region, District of Ankazobe, Ambohitantely, 46 km NE of Ankazobe, -18.198, 47.2815, 701 m, Forêt sclerophylle (Rin’Ha, Mike) (CAS); **Antsiranana**: Ampasindava, Forêt d’Ambilanivy, 3.9 km 181° S Ambaliha, -13.79861, 48.16167, 600 m, rainforest (Fisher, Griswold et al.) (CAS); SAVA Region, District of Vohemar, Antsahabelela Rain Forest, 9 km SW of Daraina, -13.2505, 49.61667, 182 m, humid Forêt (Mike, Rin’ha) (CAS). **Fianarantsoa**: 8.0 km NE Ivohibe, -22.42167, 46.89833, 1200 m, montane rainforest (B.L. Fisher, Sylvain) (CAS); PN Ampotoampoto III, 7.91 km NW Ilakaka, -22.62944, 45.189, 919 m, savannah woodland (A. Ravelomanana) (CAS); Anja Reserve, -21.85358, 46.84903, 1085 m, rupicolous vegetation on granite outcrop (B.L. Fisher et al.) (CAS); 1 km E of PN Isalo Interpretive Center, -22.62667, 45.35817, 885 m, dry wash (R. Harin’Hala) (CAS); Forêt d’Analalava, 29.6 km 280° W Ranohira, -22.59167, 45.12833, 700 m, *Uapaca* woodland (Fisher, Griswold et al.) (CAS); PN Isalo, Sahanafa River, 29.2 km 351° N Ranohira, -22.31333, 45.29167, 500 m, gallery forest (Fisher, Griswold et al.) (CAS); stream area, 900 m E of PN Isalo Interpretive Center, -22.62667, 45.35817, 750 m, open area near stream (R. Harin’Hala) (CAS). **Mahajanga**: PN Ankarafantsika, Ampijoroa SF, 160 km N Maevatanana, deciduous forest, -16.31944, 46.81333, 43 m, deciduous forest (M. Irwin and Rin’ha Harin’hala) (CAS); Boeny Region, District of Marovoay, PN Ankarafantsika, Ampijoroa SF, 160 km North of Maevatanana on RN 04, -16.31933, 46.81333, 42 m, deciduous forest (Rin’Ha, Mike) (CAS); Forêt Ambohimanga, 26.1 km 314° Mampikony, -15.96267, 47.43817, 250 m, tropical dry forest (B.L. Fisher) (CAS); Forêt de Tsimembo, 8.7 km 336° NNW Soatana, -19.02139, 44.44067, 20 m, tropical dry forest (Fisher-Griswold Arthropod Team) (CAS); Melaky Region, District of Besalampy, Marofototra dry forest, 17 km W of Besalampy, -16.72167, 44.42367, 51 m, dry wash in the dry forest (Irwin, Rin’ha) (CAS); Melaky Region, District of Maintirano, Asondrodava dry forest, 15 km N of Maintirano, -17.96533, 44.0355, 6 m, dry forest (Irwin, Rinha) (CAS); PN Ankarafantsika, Ampijoroa SF, 40 km 306° NW Andranofasika, -16.32083, 46.81067, 130 m, tropical dry forest (Fisher, Griswold et al.) (CAS); PN Tsingy de Bemaraha, 10.6 km ESE 123° Antsalova, -18.70944, 44.71817, 150 m, tropical dry forest on Tsingy (Fisher-Griswold Arthropod Team) (CAS); Réserve d’Ankoririka, 10.6 km 13° NE Tsaramandroso, -16.26722, 47.04861, 210 m, tropical dry forest (Fisher, Griswold et al.) (CAS); Station Forestière Ampijoroa, -16.31667, 46.81667, 80 m, tropical dry forest (P.S. Ward) (CAS). **Toliara**: [Androhomana]; Andranobory; Taolagnaro, -25.18333, 46.63333, 35 m (Sikora); 45 km NE Morondava, -20.05, 44.61667, 30 m, tropical dry forest (P.S. Ward) (CAS); 50 km N Morondava, -20.06667, 44.58333, in primary dry forest (A. Pauly) (CAS); 7.0 km 156° SSE Lavanono, -25.47111, 44.9885, 50 m, spiny forest/thicket (Fisher-Griswold Arthropod Team) (CAS); Androy Region, District of Tsihombe, 74 km S of Tsihombe, RS Cap Ste Marie, -25.58767, 45.163, 36 m, spiny bush (Rin’Ha, Mike) (CAS); Anosy Region, Anosyenne Mts, 29.33 km NW Manantenina, -24.13993, 47.07418, 540 m, montane rainforest (B.L. Fisher, F.A. Esteves et al.) (CAS);: Anosy Region, District of Amboasary, 58 km SW of Fort Dauphin, 08 km NW of Amboasary, Berenty Special Reserve, -25.00667, 46.30333, 85 m, Galery forest (Rin’Ha, Mike) (CAS); Anosy Region, District of Amboasary, PN Andohahela, Parcelle III, Ihazofotsy, 32 km NE Amboasary, -24.83083, 46.53617, 58 m, dry forest, spiny forest (Michael Irwin, Frank Parker, Rin’ha) (CAS); Anosy Region, District of Amboasary, 58 km SW of Fort Dauphin, 08 km NW of Amboasary, Berenty Special Reserve, -25.021, 46.3055, 36 m, spiny forest (Mike, Rin’ha) (CAS); Anosy Region, District of Fort-Dauphin, PN Andohahela, Parcelle II, Tsimela, 42 km W of Fort-Dauphin, -24.93683, 46.62667, 176 m, transition forest (Michael Irwin, Frank Parker, Rin’ha) (CAS); Anosy Region, PN Andohahela, Forêt de Manatalinjo, -24.82505, 46.57811, 90 m, spiny forest/thicket (B.L. Fisher, F.A. Esteves et al.) (CAS); Anosy Region, PN Andohahela, Forêt de Manatalinjo, -24.82466, 46.60111, 100 m, spiny forest/thicket (B.L. Fisher, F.A. Esteves et al.) (CAS); Atsimo-Andrefana Region, -23.55275, 43.74471, 45 m, coastal scrub on limestone (B.L. Fisher, F.A. Esteves et al.) (CAS); Atsimo-Andrefana Region, -23.45314, 43.76448, 20 m, coastal spiny bush on sandy soil (B.L. Fisher, F.A. Esteves et al.) (CAS); Atsimo-Andrefana Region, Antsokay Arboretum, -23.41491, 43.75499, 13 m, spiny forest/thicket (B.L. Fisher, F.A. Esteves et al.) (CAS); Fiherenana, -23.17694, 43.96083, 100 m, gallery forest (Frontier Project) (CAS); FC Analavelona, 29.2 km 343° NNW Mahaboboka, -22.675, 44.19, 1100 m, montane rainforest (Fisher, Griswold et al.) (CAS); Forêt de Beroboka, 5.9 km 131° SE Ankidranoka, -22.23306, 43.36633, 80 m, tropical dry forest (Fisher-Griswold Arthropod Team) (CAS); Forét de Kirindy, -20.0671, 44.65723, 50 m (H. Wood & J. Miller) (CAS); Forêt de Kirindy, 15.5 km 64° ENE Marofandilia, -20.06855, 44.65956667, 30 m, tropical dry forest (B.L. Fisher) (CAS); Forêt de Kirindy, 15.5 km 64° ENE Marofandilia, -20.045, 44.66222, 100 m, tropical dry forest (Fisher-Griswold Arthropod Team) (CAS); Forêt de Mahavelo, Isantoria River, -24.75833, 46.15717, 110 m, spiny forest/thicket (Fisher-Griswold Arthropod Team) (CAS); Forêt de Mite, 20.7 km 29° WNW Tongobory, -23.52417, 44.12133, 75 m, gallery forest (Fisher-Griswold Arthropod Team) (CAS); Forêt de Tsinjoriaky, 6.2 km 84° E Tsifota, -22.80222, 43.42067, 70 m, spiny forest/thicket (Fisher-Griswold Arthropod Team) (CAS); Grand Lavasoa, 25.9 km W Tolagnaro, -25.08767, 46.749, 450 m, rainforest edge (B.L. Fisher et al.) (CAS); Mahafaly Plateau, 6.2 km 74° ENE Itampolo, -24.65361, 43.99667, 80 m, spiny forest/thicket (Fisher-Griswold Arthropod Team) (CAS); Makay Mts., -21.26215, 45.17004, 505 m, Barren rock with sparse vegetation, burned grass (B.L. Fisher et al.) (CAS); Makay Mts., -21.34228, 45.18314, 410 m, Gallery forest on sandy soil (B.L. Fisher et al.) (CAS); Makay Mts., -21.20937, 45.25389, 505 m, short scrub, grass, on rocky hill (B.L. Fisher et al.) (CAS); Makay Mts., -21.22284, 45.32477, 490 m, Gallery forest on sandy soil (B.L. Fisher et al.) (CAS); Menabe Region, District of Morondava, Beroboka village 45 km NE of Morondava, Antsarongaza dry forest 07,5 km E of Beroboka, -19.9775, 44.66633, 50 m, dry forest (M. Irwin, Rin’ha) (CAS); Menabe Region, District of Morondava, Beroboka village 45 km NE of Morondava, Antsarongaza galery forest 07 km E of Beroboka, -19.9775, 44.66533, 45 m, Galery forest (M. Irwin, Rin’ha) (CAS); PN Andohahela, Forêt d’Ambohibory, 1.7 km 61° ENE Tsimelahy, 36.1 km 308° NW Tolagnaro, -24.93, 46.6455, 300 m, tropical dry forest (Fisher-Griswold Arthropod Team) (CAS); PN Andohahela, Forêt de Manatalinjo, 33.6 km 63° ENE Amboasary, 7.6 km 99° E Hazofotsy, -24.81694, 46.61, 150 m, spiny forest/thicket (Fisher-Griswold Arthropod Team) (CAS); PN Kirindy Mite, 16.3 km 127° SE Belo sur Mer, -20.79528, 44.147, 80 m, tropical dry forest (Fisher-Griswold Arthropod Team) (CAS); PN Tsimanampetsotsa, Forêt de Bemanateza, 20.7 km 81° E Efoetse, 23.0 km 131° SE Beheloka, -23.99222, 43.88067, 90 m, spiny forest/thicket (Fisher-Griswold Arthropod Team) (CAS); PN Tsimanampetsotsa, Mitoho Cave, 6.4 km 77° ENE Efoetse, 17.4 km 170° S Beheloka, -24.04722, 43.75317, 40 m, spiny forest/thicket (Fisher-Griswold Arthropod Team) (CAS); Ranobe, -23.04642, 43.61015, 20 m, spiny forest/thicket (Frontier Wilderness Project) (CAS); Ranobe-plateau, -23.00283, 43.70367, 30 m, grassland (Frontier Wilderness Project) (CAS); RS Beza Mahafaly, Parcel 1, -23.65, 44.63333, 130 m, tropical dry forest (P.S. Ward) (CAS); Réserve Privé Berenty, Forêt d’Anjapolo, 21.4 km 325° NW Amboasary, -24.92972, 46.20967, 65 m, spiny forest/thicket (Fisher-Griswold Arthropod Team) (CAS); Réserve Privé Berenty, Forêt de Bealoka, Mandraré River, 14.6 km 329° NNW Amboasary, -24.95694, 46.2715, 35 m, gallery forest (Fisher-Griswold Arthropod Team) (CAS); RS Cap Sainte Marie, 12.3 km 262° W Marovato, -25.58167, 45.16833, 200 m, spiny forest/thicket (Fisher-Griswold Arthropod Team) (CAS); 3 km E Itampolo, malaise across path of lower bench of Andrimpano Forest, -24.65783, 43.95617, 45 m, dry forest (M.E. Irwin, Rin’ha) (CAS); 5 km N Ampotaka, malaise on trail in Vitambany gallery forest, -24.65033, 43.96317, 86 m, Gallery forest (M.E. Irwin, Rin’ha) (CAS); 8 km N of Ambohimahavelona village, Ankazomena dry forest, -23.43033, 43.83417, 122 m, dry forest (M.E. Irwin, Rin’ha) (CAS); Ambohimahavelona village 33 km NE of Tulear: Andoharano dry forest, -23.44083, 43.89967, 46 m, dry forest (M.E. Irwin, Rin’ha) (CAS); Ambohimahavelona village 33 km NE of Tulear: private spiny bush, -23.44083, 43.89967, 43 m, spiny bush (M.E. Irwin, Rin’ha) (CAS); PN Andohaela, Tsimelahy, -24.93683, 46.62667, 180 m, transition forest (M.E. Irwin, F.D. Parker, R. Harin’Hala) (CAS); Andranovato, 5 km SE of Manombo, -22.81806, 43.50217, 18 m, Euphorbia forest (Fisher et al.) (CAS); PN Andohahela, Ihazofotsy - Parcel III,-24.83483, 46.48683, 80 m, tropical dry forest, transition between spiny and dry deciduous forests (M.E. Irwin, F.D. Parker, R. Harin’Hala) (CAS); Mikea Forest, -22.90367, 43.4755, 30 m, deciduous dry forest (M.E. Irwin, F.D. Parker, R. Harin’Hala) (CAS); Mikea Forest, -22.91333, 43.48222, 37 m, spiny forest (R. Harin’Hala) (CAS); near ANGAP office, PN Zombitse, -22.8865, 44.69217, 840 m, deciduous spiny forest (R. Harin’Hala) (CAS); near road, PN Zombitse, -22.8405, 44.73117, 825 m, spiny deciduous forest (R. Harin’Hala) (CAS); Parcel I, RS Beza Mahafaly, near research station, -23.6865, 44.591, 165 m, dry deciduous forest (R. Harin’Hala) (CAS); Parcel II, RS Beza Mahafaly, near Bellevue, -23.68983, 44.5755, 180 m, spiny forest (R. Harin’Hala) (CAS); PN Tsimanampetsotsa, Mitoho Forest, malaise on plateau, -24.0485, 43.75233, 150 m, dense dry forest (M.E. Irwin, Rin’ha) (CAS); Tsimelahy - Parcel II, PN Andohahela, transition forest, -24.93683, 46.62667, 180 m, transition forest (M.E. Irwin, F.D. Parker, R. Harin’Hala) (CAS); Makay, -21.35695, 45.19021, 401 m, xerophylic vegetation (J.M. Bichain) (CAS).

##### Diagnosis.

With head in full-face view, lateral margins of head anterior to eye level parallel and lacking erect hairs, posterior portion of head extending into a neck and anteromedian clypeal margin continuously forming broad convexity; dorsal outline of mesosoma broadly convex; body color black to dark brown.

##### Description.

**Minor worker.** In full-face view, head sides anterior to level of eye approximately parallel; lateral cephalic margins posterior to level of eye converging posteriorly and projecting into short neck; eye medium (EL/CS: 0.24±0.01; 0.23–0.26), protruding, and breaking lateral cephalic border, level of posterior margin located approximately at posterior 1/3 of head (PoOc/CL: 0.29±0.02; 0.26–0.32); frontal carinae more or less wide posteriorly (FR/CS: 0.26±0.01; 0.25–0.27), the same width as its smallest distance to eye; clypeus without anterolateral angle, anteromedian margin broadly rounded, mandible with two apical teeth distantly spaced; antennal scape relatively long (SL/CS: 1.56±0.06; 1.44–1.66). Mesosoma presenting even convexity; promesonotum strongly convex, posterior portion of mesonotum flat immediately anterior to weakly visible metanotal groove; propodeal dorsum almost flat, its junction to declivity bluntly angulate; propodeal dorsum 3 × as long as declivity. Petiole nodiform, with dorsal margin inclined posteriorly, forming a blunt angle to anterior margin, medially excised at the junction to posterior margin, anterior face 1/2 height of posterior face, which is inclined anteriorly to the dorsum; femur of hind leg rounded axially, not twisted basally.

**Figure 54.**
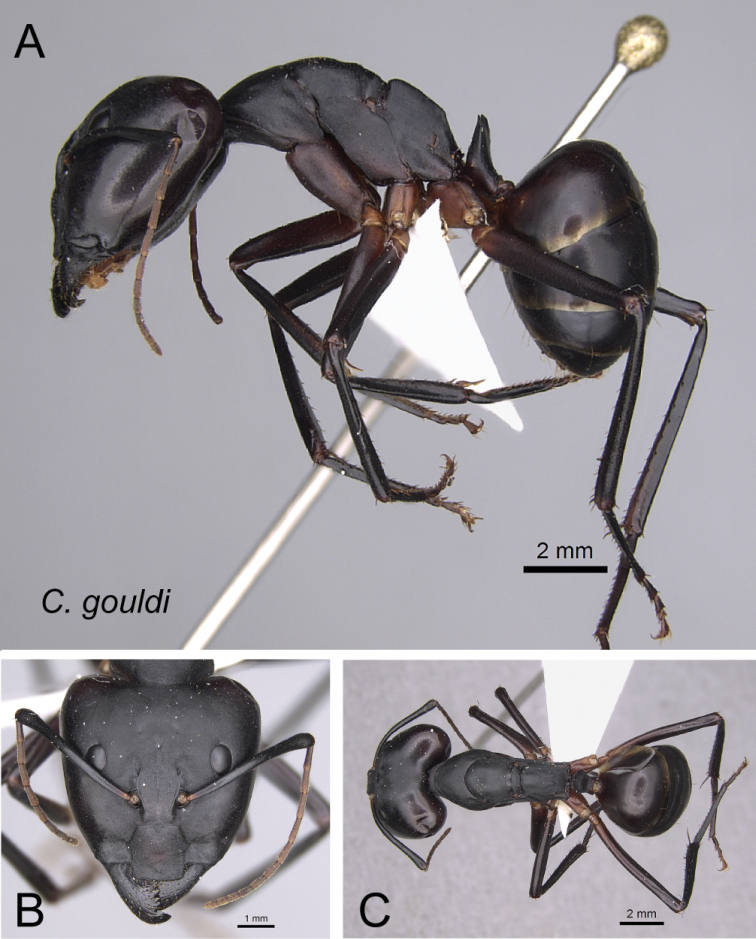
*Camponotusgouldi* (neotype specimen) **A** lateral view **B** head in full-face view **C** dorsal view of major worker CASENT0437531.

First and second gastral tergites without a pair of white spots; lateral margin of head without erect hairs; erect hairs lacking near the end of neck; antennal scape without suberect hairs and covered with short appressed hairs; pronotum with a pair of erect hairs; posterodorsal angle of propodeum without erect hairs.

**Major worker.** Characteristics the same as minor worker, except the enlarged head (CS: 5.19±0.23; 4.89–5.51; CWb/CL: 0.98±0.01; 0.97–1.00), which has no prolonged neck; the more strongly built mandible; antennal scape not extending beyond posterior cephalic margin; promesonotum convex; metanotal groove visible; propodeal dorsum feebly sloping towards declivity, its length the same as the height of declivity; petiolar node compressed anteroposteriorly.

##### Distribution and biology.

**Figure 55.**
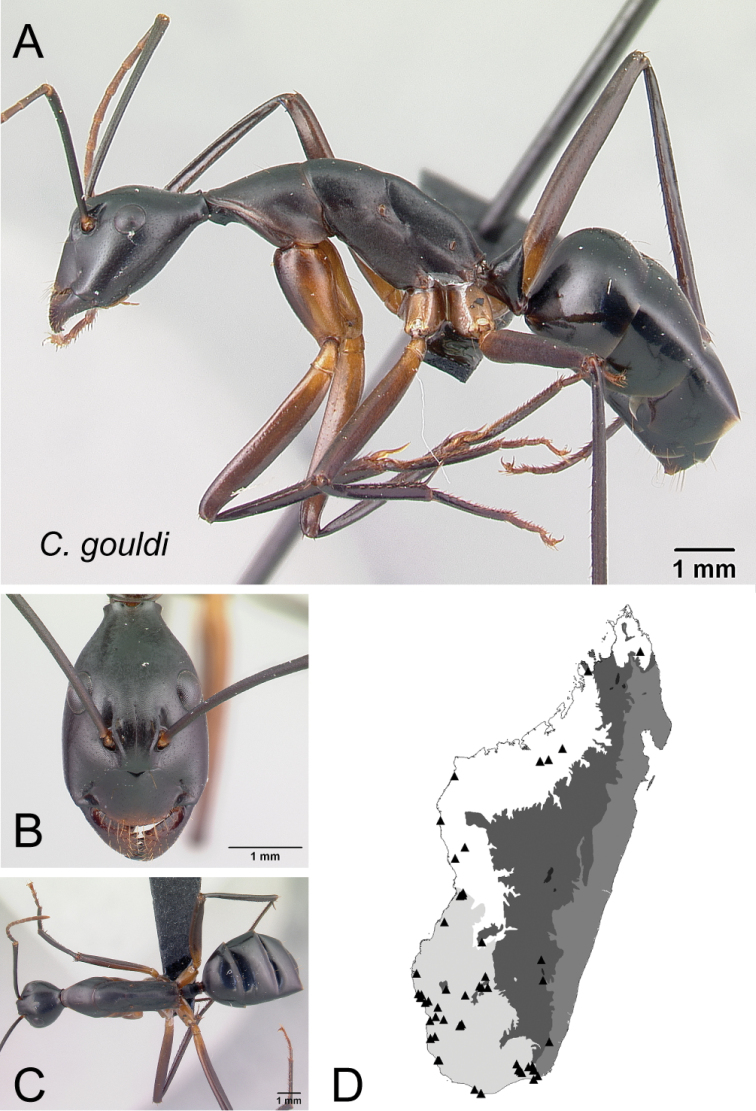
*Camponotusgouldi***A** lateral view **B** head in full-face view **C** dorsal view of minor worker CASENT0121617**D** distribution map.

The dry forest of the west, the spiny forest and thicket of the southwest, the transitional humid forest in the PN Andohahela, the savannah shrubland and woodland, and the *Uapaca* woodland and montane rainforest of the south-central high plateau of Madagascar are all habitats where *C.gouldi* occurs (Fig. 55D). This species nests mostly in rotten logs, in the ground, and under stones, rarely in rotting tree stumps and rotten sticks. Workers forage most often on the ground and seldom on lower vegetation.

##### Discussion.

*Camponotusgouldi* is mostly similar to *C.aro*, but the posterior portion of the head for the latter is normally rounded, not extending into a short neck, and the propodeal dorsum immediately posterior to the metanotal groove is convex, then becomes concave medially and rounds to the declivity surface.

For *C.gouldi*, the decisions drawn from qualitative morphology-based taxonomy agree with the classification hypothesis provided by the exploratory data analysis and the cumulative LDA of the multivariate morphometrics. The combination of this information corroborates the status of *C.gouldi* as a species.

Neotype designation has been made for *C.gouldi* because its type is presumed lost. No type specimen could be found at the renowned museums in Europe that might have held the specimen. One of the author BLF visited the Forel collection at MHNG, MNHN, NHMB, and MSNG and could not find the type for *C.gouldi*. Also, the type specimen appears to be absent from the little-known collection of Forel types at Naturmuseum Solothurn (Germann 2017). Morphological characters that define the designated neotype are in accordance with the original description of the former holotype specimen. Since the original type locality was not precisely located within Madagascar, the new locality type has been chosen on the basis of where the species was most often collected across its geographical distribution.

#### ﻿ Camponotus hagensii

Taxon classificationAnimaliaHymenopteraFormicidae

Forel

42D9CC9F-8094-5062-806D-04DCE1940D8F

Figs 33A , 56

Camponotus rubripes r. hagensii Forel, 1886a: 158. Lectotype minor worker, by present designation, Centre de Madagascar (Hildebrandt) AntWeb CASENT0910112 (MHNG) [examined]. Paralectotypes: 2 minor workers and 3 major workers of same data as lectotype but respectively specimen coded as: FOCOL2423, FOCOL2424 (ZMHB), and CASENT0910111 (MHNG), CASENT0104632 and FOCOL2422 (ZMHB), CASENT0101494 (MNHN) [examined]. As subspecies of Camponotusmaculatus, Forel, 1891: 27; of Camponotusfumidus, Santschi, 1922: 101. Raised to species by Dalla Torre 1893: 233; Forel 1914: 267; Emery 1920b: 6; Wheeler 1922: 1039; Emery 1925: 92.

##### Additional material examined.

**Madagascar: Antananarivo**: Central Madagascar; Ambatomanjaka; Miarinarivo, -18.876091, 46.865775, 1343 m (Hildebrandt) (CAS). **Antsiranana**: 12.2 km WSW Befingotra, Réserve Anjanaharibe-Sud, -14.75, 49.43333, 1960 m, montane rainforest (B.L. Fisher) (CAS); 12.2 km WSW Befingotra, Réserve Anjanaharibe-Sud, -14.75, 49.43333, 1985 m, montane rainforest (B.L. Fisher) (CAS); Nosi-Bé du Majunga; Nosy be; Nosibe, -13.315028, 48.25927, 128 m (P. Carié) (CAS); PN Marojejy, 25.4 km 30° NNE Andapa, 10.9 km 311° NW Manantenina, -14.445, 49.735, 2000 m, montane shrubland (B.L. Fisher) (CAS). **Fianarantsoa**: 36 km S Ambalavao, PN Andringitra, -22.2, 46.96667, 1900 m, montane rainforest (B.L. Fisher) (CAS); 36 km S Ambalavao, PN Andringitra, -22.2, 46.96667, 1975 m, few trees, ericoid thicket (B.L. Fisher) (CAS); 8.5 km SE Antanitotsy, Anjavidilava Forest, -22.16667, 46.96667, 1990 m, Philipia Forest (B.L. Fisher, Sylvain) (CAS); Forest d’Ambalamanakana, -20.73333, 47.2 (A. Pauly) (CAS); PN Andringitra; 8.5 km SE Antanitotsy, -22.16667, 46.96667, 1990 m, montane rainforest (Sylvain) (CAS); Soanierenana IV Non Protected Area, 25.22 km SW Ambositra, -20.72389, 47.10705, 1736 m, savannah grassland (A. Ravelomanana) (CAS); Vohiparara, -21.23333, 47.36667 (A. Pauly) (CAS). **Toliara**: Anosy Region, Anosyenne Mts, 32.5 km NW Manantenina, -24.14098, 47.03689, 1900 m, montane rainforest (B.L. Fisher, F.A. Esteves et al.) (CAS); near ANGAP office, PN Zombitse, -22.8865, 44.69217, 840 m, deciduous spiny forest (R. Harin’Hala) (CAS); near road, PN Zombitse, -22.8405, 44.73117, 825 m, spiny deciduous forest (R. Harin’Hala) (CAS).

##### Diagnosis.

In full-face view, lateral margin of head anterior to eye level diverging posteriorly and covered with erect hairs; anterior clypeal margin truncate; mesosoma in profile low and long; gastral tergites without abundant pubescence; head and gaster black, mesosoma reddish orange to brown.

##### Description.

**Minor worker.** In full-face view, head sides diverging towards broadly convex posterior margin or parallel anterior to eye level and rounding evenly to posterior border; eye protruding and large (EL/CS: 0.30±0.01; 0.28–0.33), breaking lateral cephalic margin; level of its posterior margin located ca. at posterior 1/4 of head (PoOc/CL: 0.25±0.01; 0.23–0.25); frontal carinae wide (FR/CS: 0.34±0.01; 0.31–0.35), posteriorly diverging, distance between them larger than their smallest distance to eye; clypeus without well-defined anterolateral corner, anteromedian margin approximately truncate; mandible with two apical teeth distantly spaced; antennal scape relatively long (SL/CS: 1.24±0.06; 1.09–1.36). Promesonotum weakly convex, mesonotum with posterior portion flat immediately anterior to weakly visible metanotal groove; propodeal dorsum almost straight, its junction to declivity bluntly angulate; height of propodeal declivity 3/4 length of dorsum. Petiolar node short and high; its dorsal margin inclined posteriorly, rounding to anterior margin; height of anterior face 2/3 that of posterior; femur of hind leg rounded axially, not twisted basally.

First and second gastral tergites without a pair of white spots; erect hairs on lateral margin of head lacking; more than six erect hairs present near posterior margin of head; antennal scape covered only with suberect hairs inclined at ca. 30° and appressed hairs; pronotum with few erect hairs, mesonotum bearing a pair of erect hairs; posterodorsal corner of propodeum with two pairs of erect hairs. Head, antenna, and gaster dark brown or reddish brown; mesosoma, petiolar node, and legs yellow to orange-yellow.

**Major worker.** Differing from minor worker in having larger, heart-shaped head (CS: 2.37±0.26; 2.00–2.70; CWb/CL: 0.97±0.05; 0.92–1.04) with broadly concave posterior margin, anteromedian margin of clypeus slightly excised medially; apical 1/4 of antennal scape extending beyond posterior cephalic margin; robust mesosoma with distinct metanotum; propodeal dorsum convex immediately behind metanotum, < 2 × the height of declivity surface; petiolar node much higher than long and tapering dorsally.

##### Distribution and biology.

**Figure 56.**
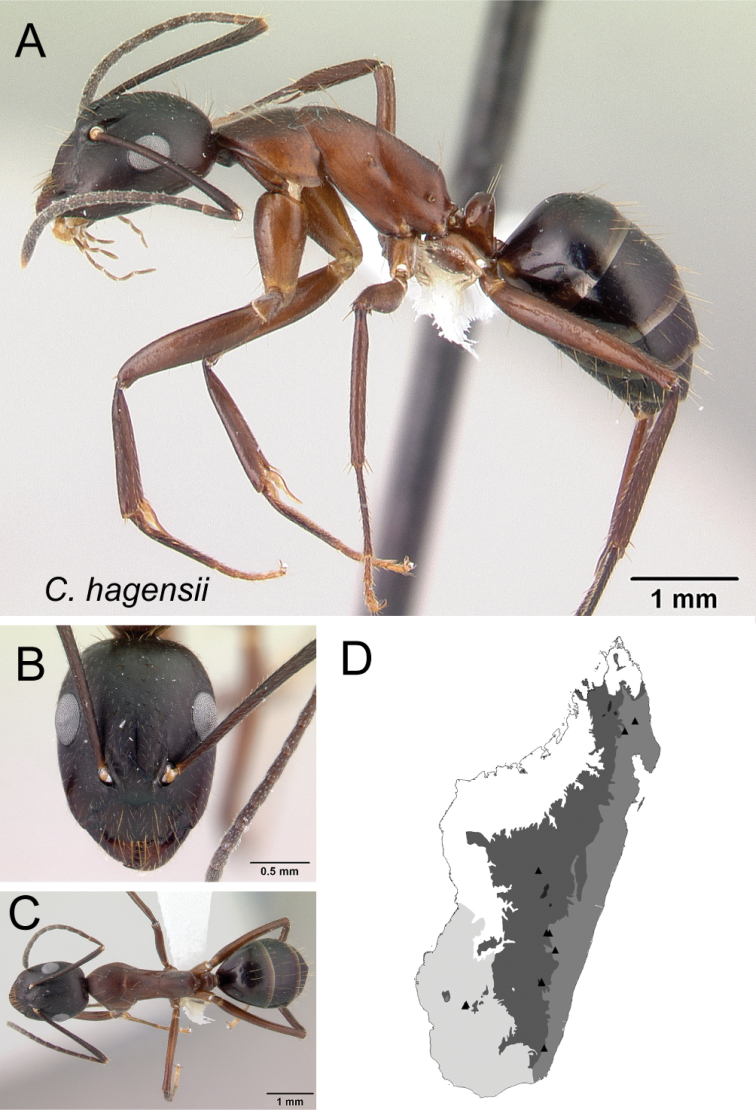
*Camponotushagensii***A** lateral view **B** head in full-face view **C** dorsal view of minor worker CASENT0191568**D** distribution map.

*Camponotushagensii* is known mostly from montane rainforest, montane ericoid thicket, philipia forest, montane shrubland, and savannah grassland of the high plateau ranging from the PN Marojejy in the north to the Anosyenne Mountains in the south (Fig. 56D). The species has been found nesting in the ground, in root mat layers on the ground, and in rotting tree stumps. Workers have been found foraging frequently on the ground and leaf litter, rarely on low vegetation.

##### Discussion.

*Camponotushagensii* is similar to *C.aurosus* with respect to the bicolored body and the presence of erect hairs on the lateral margin of the head anterior to eye level, but in *C.aurosus* the anterior clypeal margin is broadly triangular, the mesosoma is short and high in profile, and the gastral tergites are covered with abundant pubescence.

The cluster of *C.hagensii* samples presented by the dendrogram of multivariate morphometric analysis is supported by the cumulative LDA at 100% identification success. This finding is consistent with the results of the qualitative morphology-based study.

#### ﻿ Camponotus harenarum sp. nov.

Taxon classificationAnimaliaHymenopteraFormicidae

5FB7707A-D087-5FD6-9423-7DB5D7BA5034

http://zoobank.org/98A3E724-B721-4620-AC59-C6246D6E657D

Figs 5B , 57

##### Holotype worker.

**Madagascar**: Province **Antsiranana**: Forêt d’Analabe, 30 km 72° ENE Daraina, -13.08333, 49.90833, 30 m, littoral rainforest, ground forager, 28 Nov 2003 (B.L. Fisher et al.) collection code: BLF09544, specimen code: CASENT0499207 (CAS).

##### Paratype.

1 minor worker of same data as holotype but with collection code: BLF09463 and specimen code: CASENT0499217 (CAS).

##### Additional material examined.

**Madagascar: Antsiranana**: Forêt d’Analabe, 30 km 72° ENE Daraina, -13.08333, 49.90833, 30 m, littoral rainforest (B.L. Fisher) (CAS).

##### Diagnosis.

With head in full-face view, eye not breaking lateral cephalic margin; mesonotum short and lacking constriction; propodeal dorsum transversely medially concave at ca. posterior 1/2; petiolar node ca. as high as long.

##### Description.

**Minor worker.** With head in full-face view, lateral margins anterior to eye level approximately parallel, progressively rounding evenly towards slightly concave rear margin; eye protruding and large (EL/CS: 0.25±0.01; 0.25–0.26), not breaking lateral cephalic margin, location of its posterior margin at posterior 1/3 of head (PoOc/CL: 0.28±0.01; 0.27–0.29); frontal carinae not widely opened posteriorly, (FR/CS: 0.25±0.00; 0.24–0.25); clypeus without anterolateral angle, anteromedian margin broadly convex; mandible with two apical teeth normally spaced; antennal scape relatively long (SL/CS: 1.63±0.05; 1.58–1.70). Promesonotum noticeably convex, metanotal groove noticeably visible; anterior portion of propodeal dorsum convex, then concave and becoming straight with blunt angle to declivity surface. Petiole nodiform, anterior and posterior margins approximately the same height, dorsal margin separated by an angle from the anterior and gently rounding to the posterior.

First and second gastral tergites without a pair of white spots; erect hairs on lateral margin of head present anterior to and behind eye level; posterior margin of head with two elongate, erect hairs; antennal scape covered with erect hairs; junction of propodeal dorsum and declivity with two pairs of erect hairs. Body color orange-brown with darker coxa, junction of segments, abdominal sternites, and appendices.

##### Major worker.

Unknown.

##### Distribution and biology.

**Figure 57.**
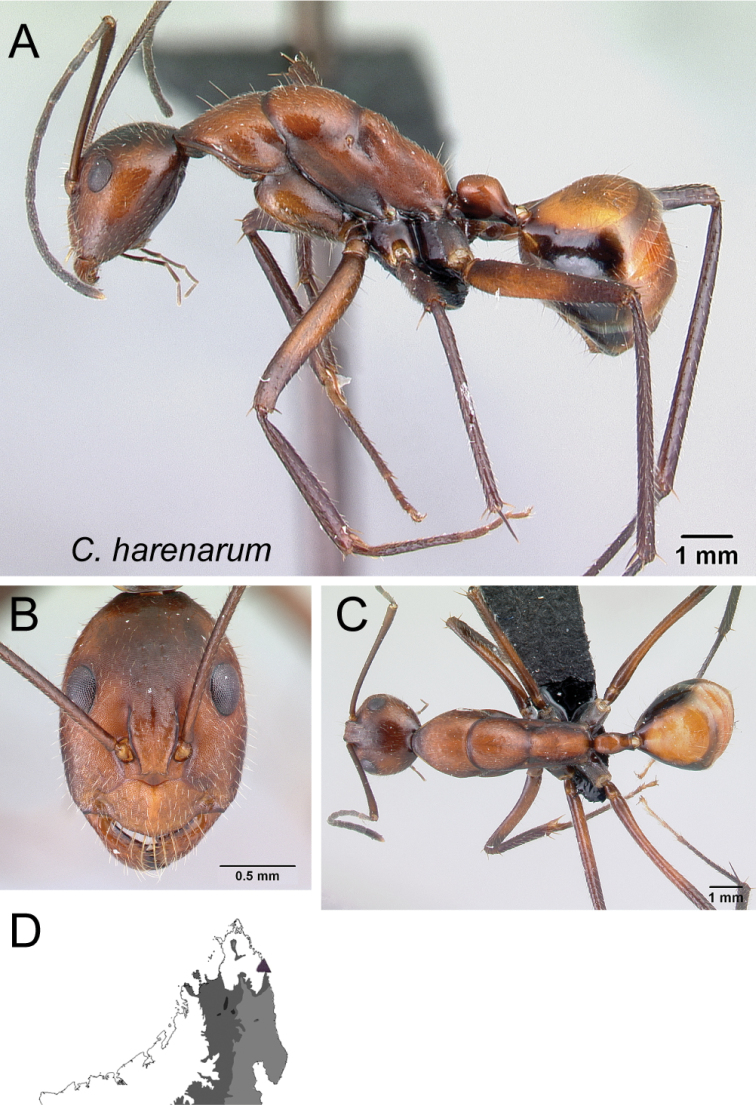
*Camponotusharenarum***A** lateral view **B** head in full-face view **C** dorsal view of holotype minor worker CASENT0499207**D** distribution map.

Endemic to Madagascar, *C.harenarum* is only known from the littoral rainforest of Analabe in Daraina, in the northeastern region of the island (Fig. 57D). This species has been found nesting in rotten logs and foraging on the forest floor and on lower vegetation.

##### Discussion.

*Camponotusharenarum* can be confounded with *C.karsti*, but the latter has a more sloping anterior portion of the propodeum that makes a noticeable angle with the posterior region, and a shorter anterior margin of the petiolar node.

##### Etymology.

The species name *harenarum* is a Latin plural noun in the genitive case of *harena*, which means sand. It is in reference to the littoral rainforest, the only habitat in which it has been found.

#### ﻿ Camponotus hova

Taxon classificationAnimaliaHymenopteraFormicidae

Forel

D25D5593-8F2C-54AB-9DF2-E82F1DBAE539

Figs 18A , 58

Camponotus maculatus r. hova Forel, 1891: 35. Syntype workers and queen, Morondava côte ouest de Madagascar (Grandidier) (MHNG); 1 syntype major worker designated as lectotype, by present designation, AntWeb CASENT0101908 (MHNG) [examined]. [First available use of Camponotusrubripesmaculatushova Forel, 1886b: 150; unavailable name]. Raised to species by Dalla Torre 1893: 235; Emery 1925: 85; Bolton 1995: 104.
Camponotus hova var. obscuratus Emery, 1925: 85. Syntype workers and male, SW Madagascar (Voeltzkow) (NHMB); 1 syntype minor worker designated as lectotype, by present designation, AntWeb CASENT0101110 (NHMB) [examined]. Paracletotype major worker with same data as lectotype but specimen coded as: CASENT0101109 (NHMB) [examined]. [First available use of Camponotusmaculatusradamae obscurata Forel, 1907: 89; Camponotusmaculatushova obscurior Santschi, 1911a: 132; unavailable names]. Syn. nov.

##### Additional material examined.

**Europa Island**: Europa Island (Voeltzkow) (MHNG); -22.34775, 40.37041, 10 m, spiny forest on coral (B.L. Fisher) (CAS); -22.33909, 40.38752, 8 m, coastal dune vegetation (B.L. Fisher) (CAS). **Juan De Nova Island**: -17.04873, 42.71009, 10 m, littoral vegetation (B.L. Fisher) (CAS); -17.06182, 42.72513, 5 m, scrub on coastal karst (B.L. Fisher) (CAS). **Madagascar: Antananarivo**: Analamanga Region, District of Ankazobe, Ambohitantely, 46 km NE of Ankazobe, -18.198, 47.2815, 701 m, Forêt sclerophylle, (Rin’ha, Fanja) (CAS), Beapombo I Non Protected Area, 22.51 km SW Antsirabe, -20.06892, 47.00404, 1663 m, Savannah grassland (A. Ravelomanana) (CAS). **Antsiranana**: Antsiranana II Pref: Antsahampano S.-Pref: Montagne d’Ambre. Site MD1, -12.52765, 49.17235, 1049 m, in *Commelina* regrowth on path next to degraded primary riparian rainforest (D. Lees, R. Ranaivosolo & P. Razafindraibe) (CAS); Montagne des Français, 7.2 km 142° SE Antsiranana (=Diego Suarez), -12.32278, 49.33817, 180 m, tropical dry forest (Fisher, Griswold et al.) (CAS); Nosy Be, RNI Lokobe, 6.3 km 112° ESE Hellville, -13.41933, 48.33117, 30 m, rainforest (Fisher, Griswold et al.) (CAS); Nosy Faly, -13.36435, 48.49137, 40 m, open secondary vegetation (B.L. Fisher et al.) (CAS); Andranomatàna, 15.2 km NW Ambilobe, -13.14965, 48.91765, 28 m, sugar cane plantation (B.L. Fisher et al.) (CAS); Forêt d’Antsahabe, 11.4 km 275° W Daraina, -13.21167, 49.55667, 550 m, tropical dry forest (B.L. Fisher) (CAS); Forêt d’Analabe, 30.0 km 72° ENE Daraina, -13.08333, 49.90833, 30 m, littoral rainforest (B.L. Fisher) (CAS); Forêt de Binara, 7.5 km 230° SW Daraina, -13.255, 49.61667, 375 m, tropical dry forest (B.L. Fisher) (CAS); Réserve Analamerana, 16.7 km 123° Anivorano-Nord, -12.80467, 49.37383, 225 m, tropical dry forest (B.L. Fisher) (CAS); Réserve Analamerana, 28.4 km 99° Anivorano-Nord, -12.74667, 49.49483, 60 m, tropical dry forest (B.L. Fisher) (CAS); RS Ambre, 3.5 km 235° SW Sakaramy, -12.46889, 49.24217, 325 m, cultivated land (Fisher, Griswold et al.) (CAS); Sakaramy, -12.44131, 49.22723, 365 m, tropical dry forest (B.L. Fisher et al.) (CAS); Sakaramy, -12.44071, 49.23061, 350 m, grassland (B.L. Fisher et al.) (CAS); Sakaramy, -12.44275, 49.2326, 313 m, tropical dry forest (B.L. Fisher et al.) (CAS); Sakaramy 07 km N of Joffre Ville, -12.33333, 49.25, 360 m, Low rain forest in open area (Mike, Frank, Rin’ha) (CAS); 7 km N Joffreville [camp 2 of Fisher], -12.33333, 49.25, 360 m, in dry forest (R. Harin’Hala) (CAS); PN Montagne d’Ambre [Petit Lac road], -12.533333, 49.166667, 1125 m, rainforest (R. Harin’Hala) (CAS); Montaigne Francais, 150 m, along forested limestone ridge (R. Harin’Hala) (CAS); PN Montagne d’Ambre [1^st^ campsite], 960 m, rainforest (R. Harin’Hala) (CAS); PN Montagne d’Ambre [lemur trail], 975 m, rainforest (R. Harin’Hala) (CAS); Andilana, Nosy Bé, -13.25, 48.18333, <5 m (D.M. Olson) (CAS). **Mahajanga**: PN Ankarafantsika, Ampijoroa SF, 160 km N Maevatanana, deciduous forest, -16.31944, 46.81333, 43 m, deciduous forest, (Irwin, Rin’ha) (CAS), Boeny Region, District of Soalala, Beaboaly Bamboo forest Station10 km SW of Soalala, 04 km of Baly village, -16.04533, 48.804, 9 m, Bamboo Forêt (Mike, Rin’ha) (CAS); Boeny Region, District of Marovoay, PN Ankarafantsika, Ampijoroa SF, 160 km North of Maevatanana on RN 04, -16.31933, 46.81333, 42 m, Deciduous forest, (Rinha, Mike) (CAS); Melaky Region, District of Maintirano, Asondrodava dry forest against dune 15 km N of Maintirano, -17.96533, 44.0355, 16 m, dry forest (Irwin, Rinha) (CAS); Melaky Region, District of Maintirano, Ampasy 50 km E of Maintirano, -18.004, 44.452, 85 m, dry forest (Mike, Rin’ha) (CAS); Melaky Region; District of Besalampy marofototra palm forest, 17 km W of Besalampy, -16.72167, 44.42367, 10 m, Palm trees on sand (Irwin, Rin’ha) (CAS); PN Baie de Baly, 12.4 km 337° NNW Soalala, -16.01, 45.265, 10 m, tropical dry forest (Fisher, Griswold et al.) (CAS); PN Tsingy de Bemaraha, 3.4 km 93° E Bekopaka, Tombeau Vazimba, -19.14194, 44.828, 50 m, tropical dry forest (Fisher-Griswold Arthropod Team) (CAS); Sofia Region, District of Port-Berger, Ambovomamy 20 km N of Port-Berger, -15.45117, 47.61333, 86 m, secondary forest on white sandy area (Mike, Frank, Rin’ha) (CAS); Boeny Region, District of Soalala, Namoroka village, Befatika Andranovory 7 km NW of Vilanandro village, -16.46967, 45.39133, 120 m, Dense dry forest (Irwin, Rin’ha) (CAS); Boeny Region, District of Soalala, Beaboaly Bamboo forest Station 10 km SW of Soalala, 04 km of Baly village, -16.04533, 48.804, 9 m, Bamboo Forêt (Mike, Rin’ha) (CAS); Boeny Region, District of Marovoay, PN Ankarafantsika, Ampijoroa SF, 160 km North of Maevatanana on RN 04, -16.31933, 46.81333, 42 m, deciduous forest (Mike, Rin’ha) (CAS); Boeny Region, District of Soalala Analamanitra forest, 14 km SW of Mitsinjo, -16.7, 45.7, 19 m, dense dry forest (Mike, Rin’ha) (CAS); Boeny Region; District of Soalala, Anjiaabo.10 km SW of Soalala, 04 km of Baly village, -16.059, 45.27417, 7 m, stabilise dune (Mike, Rin’ha) (CAS); Boeny Region; District of Soalala, Namoroka 53 km of Soalala, Ambatofolaka dry forest 3 km N of Vilanandro villlage, -16.47333, 45.39133, 105 m, dense dry forest in the mud (Mike, Rin’ha) (CAS); Forêt de Tsimembo, 8.7 km 336° NNW Soatana, -19.02139, 44.44067, 20 m, tropical dry forest (Fisher-Griswold Arthropod Team) (CAS); Melaky Region, District of Maintirano, Asondrodava dry forest against dune 15 km N of Maintirano, -17.96533, 44.0355, 16 m, dry forest (Irwin, Rinha) (CAS); Melaky Region, District of Maintirano, Asondrodava dry forest, 15 km N of Maintirano, -17.96533, 44.0355, 6 m, dry forest (Irwin, Rinha) (CAS); Sofia Region, District of Port-Berger, Ambovomamy 20 km N of Port-Berger, -15.45117, 47.61333, 86 m, secondary forest on white sandy area (Mike, Frank, Rin’ha) (CAS). **Toliara**: Androy Region, District of Tsihombe, 74 km S of Tsihombe, RS Cap Ste Marie, -25.58767, 45.163, 36 m, spiny bush (Rin’Ha, Mike) (CAS); Atsimo Andrefana Region, District of Betioky ; RS Beza Mahafaly Parcelle Belle vue 07 km W of Research Station, -23.68983, 44.5755, 177 m, spiny forest, (Rin’ha) (CAS); Atsimo Andrefana Region, District of Betioky, 30 km E Betioky, RS Beza Mahafaly (Around Research Station), -23.6865, 44.591, 165 m, Galery dry deciduous forest (Rin’Ha, Mike) (CAS); Atsimo Andrefana Region, District of Tulear II, Mikea deciduous dry forest 3 km N Andranomavo village, -22.90367, 43.4755, 30 m, Deciduous dry forest (Rin’Ha, Mike) (CAS); Atsimo Andrefana Region, District of Tulear II, Mikea deciduous dry forest 3 km N Andranomavo village, -22.90367, 43.4755, 30 m, Deciduous dry forest (Rin’Ha, Mike) (CAS); Atsimo-Andrefana Region, -23.45314, 43.76448, 20 m, coastal spiny bush on sandy soil (B.L. Fisher, F.A. Esteves et al.) (CAS); Atsimo Andrefana Region, District of Tulear II, Mikea spiny forest 8 km N Andranomavo village, -22.91333, 43.39883, 37 m, spiny forest (Rin’Ha, Mike) (CAS); Atsimo-Andrefana Region, -23.45314, 43.76448, 20 m, coastal spiny bush on sandy soil (B.L. Fisher, F.A. Esteves et al.) (CAS); Atsimo-Andrefana Region, Sarodrano, -23.52243, 43.74031, 15 m, Didiereaceae forest on sand dunes (B.L. Fisher, F.A. Esteves et al.) (CAS); FC Analavelona, 33.2 km 344° NNW Mahaboboka, -22.64333, 44.17167, 1300 m, montane rainforest (Fisher, Griswold et al.) (CAS); Forêt de Beroboka, 5.9 km 131° SE Ankidranoka, -22.23306, 43.36633, 80 m, tropical dry forest (Fisher-Griswold Arthropod Team) (CAS); Mahafaly, near Eloeste, By Lac Tsimanampetsoa, -24.16667, 43.75 (V. & B. Roth) (CAS); Menabe Region, District of Morondava, Beroboka village 45 km NE of Morondava, Antsarongaza dry forest 07.5 km E of Beroboka, -19.9775, 44.66633, 50 m, dry forest (M. Irwin, Rin’ha) (CAS); Menabe Region, District of Morondava, Beroboka village 45 km NE of Morondava, Antsarongaza galery forest 07 km E of Beroboka, -19.9775, 44.66533, 45 m, Galery forest (M. Irwin, Rin’ha) (CAS); PN Tsimanampetsotsa, 6.7 km 130° SE Efoetse, 23.0 km 175° S Beheloka, -24.10056, 43.76, 25 m, spiny forest/thicket (Fisher-Griswold Arthropod Team) (CAS); RS Beza Mahafaly, Parcel 1, -23.65, 44.63333, 130 m, tropical dry forest (P.S. Ward) (CAS); PN Andohaela Tsimelahy, -24.93683, 46.62667, 180 m, transition forest (M.E. Irwin, F.D. Parker, R. Harin’Hala) (CAS); Atsimo Andrefana Region, District of Tulear II,Tsifota 20 km N of Manombo, -22.818, 43.37267, 15 m, spiny forest (M.E. Irwin, Rin’ha) (CAS); Itampolo, Sud A Sud Hotel, malaise in dune vegetation, -24.6905, 43.944, 12 m, littoral bush (M.E. Irwin, Rin’ha) (CAS); Mikea Forest, deciduous dry forest, -22.90367, 43.4755, 30 m, deciduous dry forest (R. Harin’Hala) (CAS); Mikea Forest, spiny forest, -22.91333, 43.48222, 37 m, spiny forest (R. Harin’Hala) (CAS); Parcel I, RS Beza Mahafaly, near research station, -23.6865, 44.591, 165 m, dry deciduous forest (R. Harin’Hala) (CAS); PN Tsimanampetsotsa, Mitoho Forest, malaise across trail at escarpment base, -24.0485, 43.75233, 120 m, dense dry forest (M.E. Irwin, Rin’ha) (CAS); Ambohimahavelona village 33 km NE of Tulear, Andoharano dry forest, -23.44083, 43.89967, 46 m, dry forest (M.E. Irwin, Rin’ha) (CAS); PN Tsimanampetsotsa, Mitoho Forest, malaise across trail at escarpment base, -24.0485, 43.75233, 120 m, dense dry forest (M.E. Irwin, Rin’ha) (CAS).

##### Diagnosis.

Lateral cephalic margins approximately parallel in full-face view; two apical teeth of mandible closely spaced; antennal scape covered with erect hairs.

##### Description.

**Minor worker.** With head in full-face view, lateral margins of head anterior to level of eye parallel and converging progressively to posterior margin; head sides behind eye level ca. 1/4 length of head (PoOc/CL: 0.25±0.02; 0.22–0.28). Eyes protruding and large (EL/CS: 0.30±0.01; 0.28–0.32), breaking lateral cephalic margins; frontal carinae not widely diverging posteriorly (FR/CS: 0.27±0.01; 0.24–0.30), posteriorly parallel; clypeus with anterolateral angle and triangular or convex anteromedian margin; two apical teeth of mandible closely spaced; antennal scape relatively long (SL/CS: 1.53±0.08; 1.41–1.83). Dorsum of mesosoma highly convex, mesonotum with posterior portion flat immediately anterior to metanotal groove; metanotal groove weakly visible; propodeal dorsum almost straight, junction to declivity with blunt angle; declivity height 1/2 length of propodeal dorsum; petiolar node nodiform with dorsal margin inclined posteriorly and forming a blunt angle to anterior face; anterior face of petiolar node 1/3 height of posterior face. Tibia of hind leg rounded axially and not twisted basally.

First and second gastral tergites without a pair of white spots; lateral margin of head with erect hairs; posterior margin of head with more than six erect hairs. Antennal scape covered with suberect hairs inclined ca. 30° and abundant pubescence. Posterodorsal angle of propodeum with a pair of erect hairs.

**Major worker.** Differing from minor worker in the following characters: enlarged head (CS: 3.17±0.16; 2.94–3.48; CWb/CL: 0.94±0.03; 0.89–1.01) with broadly concave posterior margin; two apical teeth of mandible normally spaced; apical 1/4 of antennal scape surpassing posterior cephalic margin; robust mesosoma, metanotum distinctly visible, propodeal dorsum joining declivity in broad angle; dorsal margin of petiolar node inclined posteriorly from shorter anterior face towards much longer posterior face. More pairs of erect hairs on promesonotum, junction of propodeal dorsum and declivity, and posterodorsal margin of petiolar node.

##### Distribution and biology.

**Figure 58.**
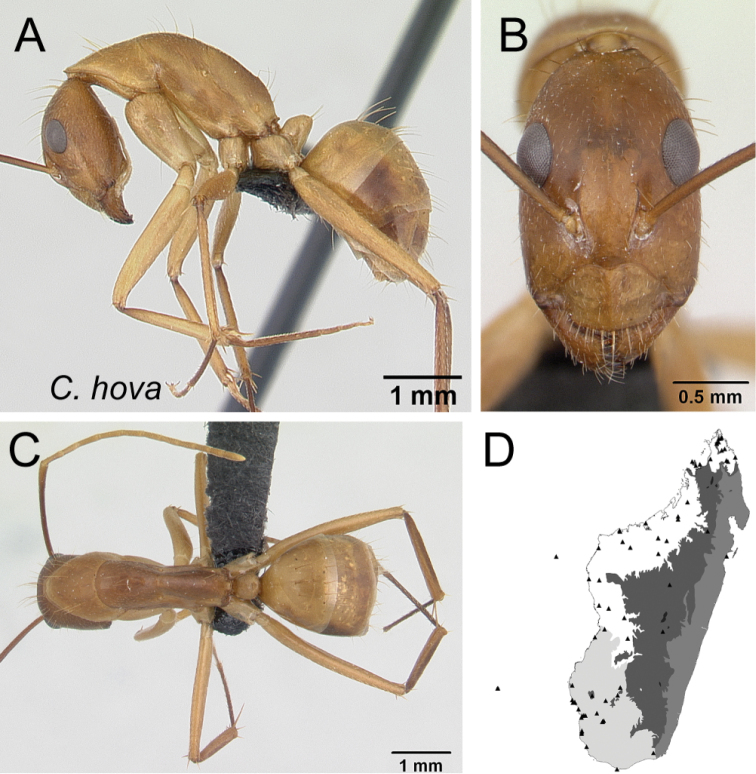
*Camponotushova***A** lateral view **B** head in full-face view **C** dorsal view of minor worker CASENT0055710**D** distribution map.

*Camponotushova* is a widespread species that occurs in dry forests of the west from the north throughout the center and the southwest of Madagascar, and is also known from Juan de Nova, Europa, and Mayotte islands (Fig. 58D). On these small islands, members of this species occupy coastal spiny bush on sand, spiny forest on coral, and coastal dune vegetation. This species is also capable of colonizing human-modified habitats. Workers are found foraging on the ground and nest sites are located in rotten logs, in the ground, and in rot pockets above the ground.

##### Discussion.

*Camponotushova* shows significant morphological variation across its wide distribution in Madagascar and nearby islands. Two variants are recognized according to the forms of the dorsum of the propodeum, but these merge gradually into the typical form through their geographical distribution.

**Variant 1.** Workers of this variant express the typical form by which the posterior 1/2 of the mesonotum to the posterodorsal corner of the propodeum is straight in profile. The petiolar node is characterized by the posterior inclination of the dorsal margin toward the anterior face, which is ca. 1/3 the height of the posterior face. This variant occupies the southwest region of Madagascar and surrounding islands. Integument is brown to dark brown or blackish brown in color.

**Variant 2.** This variant is known from the northwestern part of Madagascar and is characterized by the presence of a slightly broad concavity from the posterior 1/2 of mesonotum to posterodorsal corner of propodeum in lateral view and by the pale yellow to reddish orange color the body. Its petiole is nodiform, and the dorsal margin rounds to the anterior face, which is 1/2 height of the posterior face.

Based on the original description of *C.hova* (Forel, 1891) and *C.hovaobscuratus* (Emery, 1925) and the examination of their respective syntype specimens, there are no distinctive characters that were found to differentiate both taxa. The observation of the collection obtained from the recent survey of the Malagasy ant fauna indicates that the distinctive characters of *C.hovaobscuratus* vary within and across the populations of *C.hova*. Therefore, it is reasonable to place safely *C.hovaobscuratus* in synonymy here.

Although qualitative morphology recognized the two variants in *C.hova*, the exploratory analysis of NC-clustering did not completely detect their distinction. The combination of the two variants is classified by confirmatory LDA at 100% of success.

#### ﻿ Camponotus hovahovoides

Taxon classificationAnimaliaHymenopteraFormicidae

Forel stat. rev.

B744AF47-94B9-5F83-8B34-95C2D20DF665

**Figure 59.**
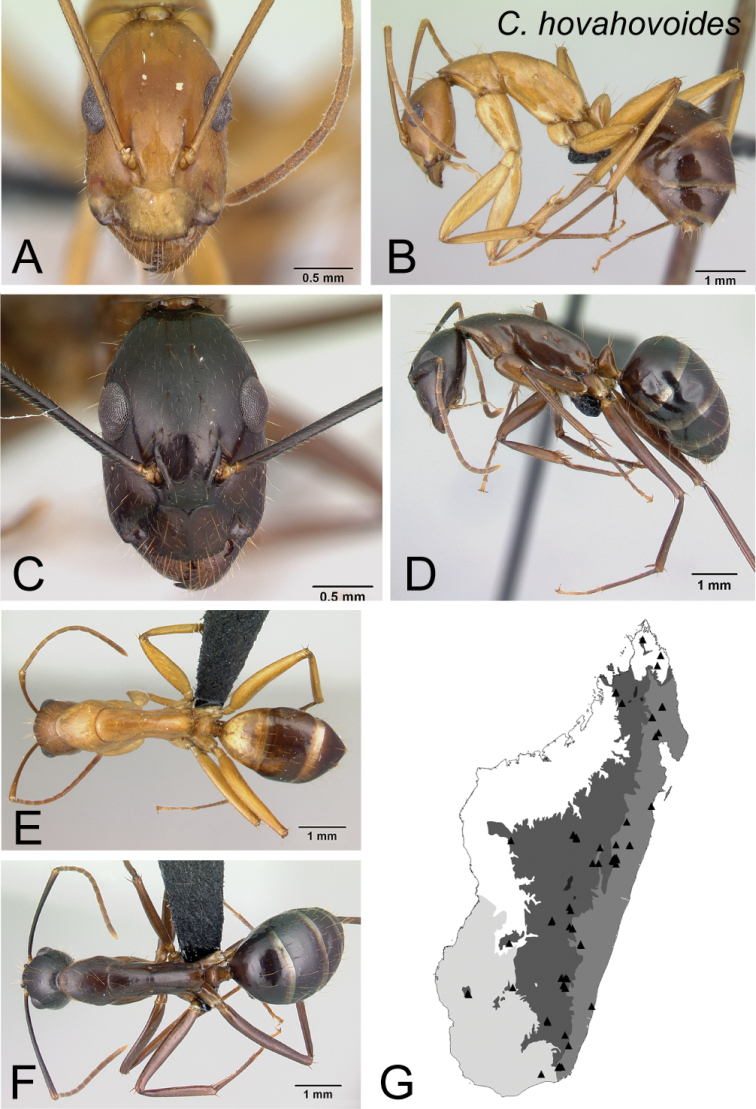
*Camponotushovahovoides***A, C** head in full-face view **B, D** lateral view **E, F** dorsal view of minor workers CASENT0496908 and CASENT0487242**G** distribution map.

Figs 20B , 21B , 22A , 59

Camponotus hovahovoides Forel, 1892: 232. Syntype minor and major workers, queen and male, Madagascar, Andrangoloaka (Sikora) (MHNG) [examined]; 1 syntype minor worker designated as lectotype, by present designation, AntWeb CASENT0101335. Paralectotypes with same data as lectotype but: 1 major worker CASENT0101851 (MHNG), 1 queen CASENT0101629 (MHNG), 1 male CASENT0101759 (MHNG) [examined]. [As subspecies of Camponotushova Dalla Torre, 1893: 235]. Combination in Camponotus (Tanaemyrmex): Emery, 1925: 85. Stat. rev.
Camponotus radamae var. hovoides Dalla Torre, 1893: 249. Syntype minor and major workers, queen and male, Madagascar; 1 syntype minor worker designated as lectotype, by present designation, Antananarivo (Camboué) AntWeb CASENT0101421 (MNHN) [examined]. Paralectotypes: 2 minor workers CASENT0101429 (MNHN), CASENT0101337 (MHNG); 1 queen CASENT0101412 (MNHN); Madagascar (Hildebrandt) 1 major worker CASENT0101954 (MHNG) and 1 male CASENT0101782 (MHNG) [examined]. [First available use of Camponotusmaculatusradamaehovoides Forel, 1891: 33; unavailable name]. Combination in Camponotus (Tanaemyrmex): Emery, 1925: 85. Syn. nov.

##### Additional material examined.

**Madagascar: Antananarivo**: 3 km 41° NE Andranomay, 11.5 km 147° SSE Anjozorobe, -18.47333, 47.96, 1300 m, montane rainforest (Fisher, Griswold et al.) (CAS); Analamanga Region, District of Ankazobe, Ambohitantely, 46 km NE of Ankazobe, -18.198, 47.2815, 701 m, Forêt sclerophylle (Rin’Ha, Mike) (CAS); Forêt de galerie, Andranorovitra, 24.0 km NNE Ankazobe, -18.11243, 47.19757, 1491 m, disturbed gallery montane forest (B.L. Fisher et al.) (CAS); Forêt de galerie, Telomirahavavy, 23.4 km NNE Ankazobe, -18.12167, 47.20627, 1520 m, disturbed gallery montane forest (B.L. Fisher et al.) (CAS); Mandraka, -18.91813, 47.91717, 1312 m, montane rainforest (B.L. Fisher et al.) (CAS); Région Analamanga, SF Mandraka, -18.9183, 47.91687, 1285 m, montane rainforest (B.L. Fisher, F.A. Esteves et al.) (CAS); Réserve Naturelle Sohisika, Sohisika 24.6 km NNE Ankazobe, -18.10322, 47.18692, 1464 m, gallery montane forest (B.L. Fisher et al.) (CAS); RS Ambohitantely, -18.22444, 47.2774, 1490 m, montane forest (B.L. Fisher et al.) (CAS); RS Ambohitantely, -18.18762, 47.28576, 1580 m, montane forest (B.L. Fisher et al.) (CAS); RS Ambohitantely, Forêt d Ambohitantely, 20.9 km 72° NE d Ankazobe, -18.22528, 47.28683, 1410 m, montane rainforest (Fisher, Griswold et al.) (CAS); RS Ambohitantely, Forêt d Ambohitantely, Jardin Botanique, 24.1 km 59° NE d Ankazobe, -18.17139, 47.28182, 1620 m, montane rainforest (Fisher, Griswold et al.) (CAS); SF Angavokely, -18.92207, 47.74157, 1460 m, rainforest (B.L. Fisher et al.) (CAS). **Antsiranana**: PN Montagne d’Ambre, -12.51389, 49.17784, 984 m, montane rainforest (B.L. Fisher et al.) (CAS); 11.0 km WSW Befingotra, Réserve Anjanaharibe-Sud, -14.75, 49.45, 1585 m, montane rainforest (B.L. Fisher) (CAS); 11.0 km WSW Befingotra, Réserve Anjanaharibe-Sud, -14.75, 49.45, 1565 m, montane rainforest (B.L. Fisher) (CAS); 11.0 km WSW Befingotra, Réserve Anjanaharibe-Sud, -14.75, 49.45, 1550 m, montane rainforest (B.L. Fisher) (CAS); 11.0 km WSW Befingotra, Réserve Anjanaharibe-Sud, -14.75, 49.45, 1565 m, montane rainforest (B.L. Fisher) (CAS); 9.2 km WSW Befingotra, Réserve Anjanaharibe-Sud, -14.75, 49.46667, 1280 m, montane rainforest (B.L. Fisher) (CAS); 9.2 km WSW Befingotra, Réserve Anjanaharibe-Sud, -14.75, 49.46667, 1200 m, montane rainforest (B.L. Fisher) (CAS); Forêt d’Ampondrabe, 26.3 km 10° NNE Daraina, -12.97, 49.7, 175 m, tropical dry forest (B.L. Fisher) (CAS); Forêt de Binara, 9.1 km 233° SW Daraina, -13.26333, 49.60333, 800 m, rainforest (B.L. Fisher) (CAS); Forêt de Binara, 9.4 km 235° SW Daraina, -13.26333, 49.6, 1100 m, montane rainforest (B.L. Fisher) (CAS); PN Marojejy, 25.4 km 30° NNE Andapa, 10.9 km 311° NW Manantenina, -14.445, 49.735, 2000 m, montane shrubland (B.L. Fisher) (CAS); PN Marojejy, 25.7 km 32° NNE Andapa, 10.3 km 314° NW Manantenina, -14.445, 49.74167, 1575 m, montane rainforest (B.L. Fisher) (CAS); PN Marojejy, Antranohofa, 26.6 km 31° NNE Andapa, 10.7 km 318° NW Manantenina, -14.44333, 49.74333, 1325 m, montane rainforest (B.L. Fisher) (CAS); RS Manongarivo 17.3 km 218° SW Antanambao, -14.02167, 48.41833, 1580–1600 m, montane rainforest (B.L. Fisher) (CAS); RS Manongarivo, 20.4 km 219° SW Antanambao, -14.04667, 48.40167, 1860 m, montane rainforest (B.L. Fisher) (CAS); PN Marojejy, 10.5 km NW Manantenina, -14.43333, 49.75, 1625 m, montane rainforest (E.L. Quinter) (CAS); PN Marojejy, 11 km NW Manantenina, -14.45, 49.73333, 1875 m, montane rainforest (E.L. Quinter) (CAS). **Fianarantsoa**: 28 km SSW Ambositra, Ankazomivady, -20.775, 47.16833, 1670 m, disturbed montane rainforest (B.L. Fisher) (CAS); 29 km SSW Ambositra, Ankazomivady, -20.77667, 47.165, 1700 m, disturbed montane rainforest (B.L. Fisher) (CAS); 28,5 km SW Ambositra, -20.78414, 47.16699, 1780 m, disturbed montane rainforest along road side (B.L. Fisher et al.) (CAS); 45 km S Ambalavao, -22.21667, 47.01667, 785 m, rainforest (B.L. Fisher) (CAS); 38 km S Ambalavao, PN Andringitra, -22.2, 46.96667, 1680 m, montane rainforest (B.L. Fisher) (CAS); 40 km S Ambalavao, PN Andringitra, -22.21667, 46.96667, 1200–1275 m, rainforest (B.L. Fisher) (CAS); 8.0 km NE Ivohibe, -22.42167, 46.89833, 1200 m, montane rainforest (B.L. Fisher, Sylvain) (CAS); 9.0 km NE Ivohibe, -22.42667, 46.93833, 900 m, rainforest (B.L. Fisher, Sylvain) (CAS); RS Ivohibe, 6.5 km ESE Ivohibe, -22.49667, 46.955, 1575 m, montane rainforest (B.L. Fisher, Sylvain) (CAS); Amoron’i Mania Region, District of Ambositra, Italaviana *Uapaca* forest, 35 km SE of Antsirabe, -20.17333, 47.086, 1359 m, *Uapaca* forest (Rin’Ha, Mike) (CAS); Antohatsahomby II Non Protected Area, 23.38 km NW Itremo, -20.55444, 46.58438, 1640 m, *Uapaca* woodland (A. Ravelomanana) (CAS); Belle Vue trail, PN Ranomafana, -21.2665, 47.42017, 1020 m, mixed tropical forest (R. Harin’Hala) (CAS); Vatovavy Fitovinany Region, District of Ifanadiana, 12 km W of Ranomafana, -21.25083, 47.40717, 1127 m, forest edge, open area (Rin’Ha, Mike) (CAS); Forêt d’Atsirakambiaty, 7.6 km 285 °WNW Itremo, -20.59333, 46.56333, 1550 m, montane rainforest (Fisher, Griswold et al.) (CAS); JIRAMA water works near river, PN Ranomafana, -21.2485, 47.45217, 690 m, open area near stream (R. Harin’Hala) (CAS); Mampiarika IV Non Protected Area, 27.98 km SW Ambositra, -20.73528, 47.08382, 1486 m, *Uapaca* woodland (A. Ravelomanana) (CAS); Miandritsara Forest, 40 km S of Ambositra, -20.79267, 47.17567, 822 m, Low altitude rainforest (Rin’Ha, Mike) (CAS); PN Andringitra, Forêt Ravaro 12.5 km SW Antanifotsy, -22.21167, 46.845, 1500–1800 m, montane rainforest, (S. Razafimandimby) (CAS); PN Befotaka-Midongy, Papango 27.7 km S Midongy-Sud, Mount Papango, -23.83517, 46.96367, 940 m, rainforest (B.L. Fisher et al.) (CAS); PN Befotaka-Midongy, Papango 28.5 km S Midongy-Sud, Mount Papango, -23.84083, 46.9575, 1250 m, montane rainforest (B.L. Fisher et al.) (CAS); PN Ranomafana, Sahamalaotra River, 6.6 km 310° NW Ranomafana, -21.23667, 47.39667, 1150 m, montane rainforest (Fisher, Griswold et al.) (CAS); PN Ranomafana, Sahamalaotra River, 6.6 km 310° NW Ranomafana, -21.23667, 47.39667, 1150 m, montane rainforest (Fisher, Griswold et al.) (CAS); PN Ranomafana, Sahamalaotra River, 6.6 km 310° NW Ranomafana, -21.23667, 47.39667, 1150 m, montane rainforest (Fisher, Griswold et al.) (CAS); PN Ranomafana, Vatoharanana River, 4.1 km 231° SW Ranomafana, -21.29, 47.43333, 1100 m, montane rainforest (Fisher, Griswold et al.) (CAS); PN Isalo, 9.1 km 354° N Ranohira, -22.48167, 45.46167, 725 m, gallery forest (Fisher, Griswold et al.) (CAS); Parc naturel communautaire, radio tower, PN Ranomafana, -21.25833, 47.40717, 1130 m, forest edge, mixed tropical forest, open area (M. Irwin, R. Harin’Hala) (CAS); RS Manombo 24.5 km 228° Farafangana, -23.01583, 47.719, 30 m, rainforest (B.L. Fisher et al.) (CAS); Vohiparara broken bridge, -21.22617, 47.36983, 1110 m, high altitude rainforest (R. Harin’Hala) (CAS); Col des Tapias, -20.26667, 47.11667, 1500 m, *Uapaca* woodland (CAS); PN Ranomafana, Vatoharanana River, 4.1 km 231° SW Ranomafana, -21.29, 47.43333, 1100 m, montane rainforest (Fisher, Griswold et al.) (CAS). **Mahajanga**: Region Sofia, Bemanevika, -14.32826, 48.58406, 1657 m, montane rainforest (B.L. Fisher et al.) (CAS); Region Sofia, Bemanevika, -14.337, 48.58874, 1606 m, montane rainforest (B.L. Fisher et al.) (CAS). **Toamasina**: Ambatovy, 12.4 km NE Moramanga, -18.83937, 48.30842, 1080 m, montane rainforest (B.L. Fisher et al.) (CAS); Analamay, -18.80623, 48.33707, 1068 m, montane rainforest (Malagasy ant team) (CAS); Ankerana, -18.40636, 48.80254, 1108 m, montane forest (B.L. Fisher et al.) (CAS); Ankerana, -18.4104, 48.8189, 855 m, rainforest (B.L. Fisher et al.) (CAS); Corridor Forestier Analamay-Mantadia, Ambatoharanana, -18.80424, 48.40081, 968 m, rainforest (B.L. Fisher et al.) (CAS); Corridor Forestier Analamay-Mantadia, Ambatoharanana, -18.80398, 48.40358, 1064 m, rainforest (B.L. Fisher et al.) (CAS); Corridor Forestier Analamay-Mantadia, Ambatoharanana, -18.80398, 48.40358, 1064 m, rainforest (B.L. Fisher et al.) (CAS); Corridor Forestier Analamay-Mantadia, Ambatoharanana, -18.80388, 48.40506, 1013 m, rainforest (B.L. Fisher et al.) (CAS); Corridor Forestier Analamay-Mantadia, Ambohibolakely, -18.77908, 48.36628, 1014 m, rainforest (B.L. Fisher et al.) (CAS); Corridor Forestier Analamay-Mantadia, Ambohibolakely, -18.76087, 48.37128, 1044 m, rainforest (B.L. Fisher et al.) (CAS); Corridor Forestier Analamay-Mantadia, Tsaravoniana, -18.75641, 48.42195, 1036 m, rainforest (B.L. Fisher et al.) (CAS); District of Moramanga, 29 km E of Moramanga; PN Andasibe, -18.93767, 48.41167, 822 m, rainforest (Mike, Rin’ha) (CAS); Forêt Ambatovy, 14.3 km 57° Moramanga, -18.85083, 48.32, 1075 m, montane rainforest (Malagasy ant team) (CAS); Montagne d’Akirindro 7.6 km 341° NNW Ambinanitelo, -15.28833, 49.54833, 600 m, rainforest (Fisher, Griswold et al.) (CAS); Montagne d’Anjanaharibe, 19.5 km 27° NNE Ambinanitelo, -15.17833, 49.635, 1100 m, montane rainforest (Fisher, Griswold et al.) (CAS); PN Andasibe-Mantadia, Forêt de Mantadia, 25.7 km 248° Moramanga, -18.81402, 48.43028, 1040 m, rainforest (F.N. Raharimalala, B. Blaimer) (CAS); PN Zahamena, -17.73359, 48.72625, 950 m, rainforest (B.L. Fisher et al.) (CAS); PN Zahamena, Tetezambatana forest, near junction of Nosivola and Manakambahiny Rivers, -17.74298, 48.72936, 860 m, rainforest (B.L. Fisher et al.) (CAS); Parcelle E3 Tampolo, -17.28104, 49.43012, 10 m, littoral forest (Malagasy ant team) (CAS); SF Analamazaotra, Analamazaotra 1.3 km S Andasibe, -18.38466, 48.41271, 980 m, montane rainforest (B.L. Fisher et al.) (CAS); Torotorofotsy, -18.87082, 48.34737, 1070 m, montane rainforest, marsh edge (Malagasy ant team) (CAS). **Toliara**: 6 km SSW Eminiminy, Res. Andohahela, -24.73333, 46.8, 330 m, rainforest (P.S. Ward) (CAS); Anosy Region, Anosyenne Mts, 31.2 km NW Manantenina, -24.13632, 47.05485, 1315 m, rainforest (B.L. Fisher, F.A. Esteves et al.) (CAS); Anosy Region, Anosyenne Mts, 31.2 km NW Manantenina, -24.13894, 47.06804, 1125 m, rainforest (B.L. Fisher, F.A. Esteves et al.) (CAS); FC Analavelona, 29.2 km 343° NNW Mahaboboka, -22.675, 44.19, 1100 m, montane rainforest (Fisher, Griswold et al.) (CAS); FC Analavelona, 33.2 km 344° NNW Mahaboboka, -22.64333, 44.17167, 1300 m, montane rainforest (Fisher, Griswold et al.) (CAS); PN Andohahela, Manangotry, 33.8 km NW Tolagnaro, -24.75117, 46.85783, 575 m, rainforest (B.L. Fisher et al.) (CAS); PN Andohahela, Col du Sedro, 3.8 km 113° ESE Mahamavo, 37.6 km 341° NNW Tolagnaro, -24.76389, 46.75167, 900 m, montane rainforest (Fisher-Griswold Arthropod Team) (CAS); RS Ambohijanahary, Forêt d’Ankazotsihitafototra, 35.2 km 312° NW Ambaravaranala, -18.26667, 45.40667, 1050 m, montane rainforest (Fisher, Griswold et al.) (CAS); RS Kalambatritra, Ambinanitelo, -23.4502, 46.45658, 1325 m, montane rainforest (B.L. Fisher et al.) (CAS); RS Kalambatritra, Ampanihy, -23.4635, 46.4631, 1270 m, montane rainforest (B.L. Fisher et al.) (CAS); RS Kalambatritra, Ampanihy, -23.463, 46.47057, 1269 m, montane rainforest (B.L. Fisher et al.) (CAS); RS Kalambatritra, Befarara, -23.4178, 46.4478, 1390 m, montane rainforest (B.L. Fisher et al.) (CAS); RS Kalambatritra, Betanana, -23.4144, 46.459, 1360 m, montane rainforest (B.L. Fisher et al.) (CAS).

##### Diagnosis.

Lateral cephalic margins approximately parallel in full-face view; two apical teeth of mandible normally spaced; antennal scape covered with suberect hairs; in lateral view, dorsum of mesosoma from mid-mesonotum to posterodorsal corner of propodeum approximately straight, propodeal dorsum ca. 3 × as long as the height of declivity surface; petiolar node flattened anteroposteriorly.

##### Description.

**Minor worker.** Head sides anterior to level of eye parallel; lateral margins posterior to level of eye converging progressively to posterior margin; length of posterior portion of head behind eye level 1/3 length of head (PoOc/CL: 0.28±0.01; 0.25–0.31). Eyes protruding and large (EL/CS: 0.30±0.01; 0.27–0.34), breaking lateral cephalic margin. Frontal carinae strongly diverging posteriorly (FR/CS: 0.30±0.01; 0.28–0.33); clypeus projecting into anterolateral angle and medially with blunt angle or convex. Two apical teeth of mandible distantly spaced. Antennal scape relatively long (SL/CS: 1.54±0.08; 1.43–1.75) with erect to suberect hairs inclined to 45°. Promesonotum weakly convex and mesopropodeum almost flat, metanotal groove weakly visible, propodeal dorsum anteriorly convex and posteriorly flat, joining the declivity in a blunt angle, propodeal declivity height 1/2 dorsum length. Petiolar node flattened anteroposteriorly or short and high, its dorsal margin rounding to anterior margin. Tibia of hind leg rounded and not twisted dorsoventrally.

First and second gastral tergites without a pair of white spots. Lateral margin of head covered with erect hairs; posterior margin of head with two or three pairs of erect hairs; posterodorsal angle of propodeum with one or two pairs of erect hairs.

**Major worker.** Characteristics the same as minor worker, except the enlarged head (CS: 2.66±0.20; 2.40–3.07; CWb/CL: 0.77±0.02; 0.72–0.80); the more strongly built mandible; apical 1/5 of antennal scape surpassing posterior cephalic margin; metanotum visible; propodeal dorsum convex and its junction to declivity broadly angulate; petiolar node much higher than long.

##### Distribution and biology.

A widely distributed species, *C.hovahovoides* occurs mainly in the eastern lowland to montane rainforests and montane shrublands of Madagascar (Fig. 59G). Colonies can be found mostly in rotten logs and sticks or in rotting tree stumps; they are seldom established in dead branches or twigs above the ground. Workers forage on the ground or through leaf litter, rarely on lower parts of vegetation.

##### Discussion.

See also discussion under *C.mixtellus*. *Camponotushovahovoides* is one of the more common species of *Myrmosaga* occupying the montane rainforests of Madagascar and shows notable morphological variation. Two variants are recognized based on the shapes of the mesosoma and body colors, but these merge progressively into one typical form across the geographic distribution of the species.

**Variant 1.** In this variant the posterior 1/2 of mesonotum to posterodorsal corner of propodeum is not straight in lateral view and the body is pale yellow to reddish orange in color.

**Variant 2.** This variant is known by a dorsum of mesosoma from mid-mesonotum to posterodorsal corner of propodeum that is approximately straight, and its body is dark brown to black in color or the mesosoma is lighter in color relative to the darker head and gaster. Workers of this variant have been collected from mountaintops.

*Camponotushovahovoides* has a wide distribution across the humid forest in Madagascar, this information combined with the results of the comparison of the syntype specimens of C.radamaevar.hovoides with those of *C.hovahovoides* indicate that there is no strong morphological difference between both taxa. The few specimens of C.radamaevar.hovoides represent a rare variation of *C.hovahovoides* across its geographic distribution.

The NC-Clustering technique was used to reveal the two variants and demonstrates that the quantitative morphological characters did not identify the difference between both variants. The members of each variant are scattered along the cluster of *C.hovahovoides*. More robust molecular phylogenetic information is needed to determine whether the variants constitute separate species and to study the ecological and evolutionary forces underlying these morphological variations.

#### ﻿ Camponotus imitator

Taxon classificationAnimaliaHymenopteraFormicidae

Forel

85799396-A938-58A4-B51B-F96AEEC221A8

Fig. 16A (see Rasoamanana et al. 2017 for the description)

##### Diagnosis.

With head in full-face view, posterior portion of head extending into a neck, and anteromedian clypeal margin continuously forming broad convexity; dorsal outline of mesosoma complex; body color black to dark brown.

##### Distribution and biology.

See the geographic distribution and biology in Rasoamanana et al. (2017).

##### Discussion.

*Camponotusimitator* can be easily separated from the other species of the *Myrmosaga* group in that it is the only species that is characterized by an elongate mesonotum that is constricted at midlength, and its propodeum is broadly convex when the mesosoma is viewed in profile.

See also discussion under the section “Morphological diagnosis of the worker caste of the Malagasy *Myrmosaga*”.

The grouping of *C.imitator* revealed by the NC-clustering method dendrogram is confirmed by the cumulative LDA at 100% identification success, indicating the support of the species hypothesized by qualitative morphology-based taxonomy.

#### ﻿ Camponotus immaculatus

Taxon classificationAnimaliaHymenopteraFormicidae

Forel

19C0A67F-89B8-5B00-B34A-EE4F467C6AF9

Figs 2C , 36A , 60

Camponotus quadrimaculatus var. immaculatus Forel, 1892: 233. Syntype workers, Madagascar Andrangoloaka (Sikora); 1 syntype minor worker designated as lectotype, by present designation, AntWeb CASENT0102430 (MHNG). Paralectotypes: 2 major workers of same data as lectotype but with specimen codes: CASENT0102429, CASENT0102431 (MHNG) (Andrangoloaka). Raised to species: Olson and Ward 1996: 164.
Camponotus quadrimaculatus opacata Emery, 1925: 123. Syntype workers and alate queen, Madagascar, Baie d’Antongil (Mocquerys); 1 syntype minor worker designated as lectotype, by present designation, AntWeb CASENT0102107) (MSNG). Paralectotypes: 2 major worker and one alate queen of same data as lectotype but respectively with the following specimen codes: CASENT0102106 (MSNG), CASENT0102433 (MHNG) and CASENT0102108 (MSNG). [Replacement name for Camponotusquadrimaculatusopaca Emery, 1899: 290. Junior secondary homonym of Formicaopaca (now Camponotusopaca) Nylander, 1856: 55]. Syn. nov.

##### Additional material examined.

**Madagascar: Antananarivo**: [Andrangoloaka]; Mantasoa; Manjakandriana, -19.033333, 47.9166666, 1409 m (Sikora) (MHNG); Kaloy, -18.58998, 47.65102, 1423 m, disturbed montane rainforest (B.L. Fisher et al.) (CAS); Analamanga Region, District of Ankazobe, Ambohitantely, 46 km NE of Ankazobe, -18.198, 47.2815, 701 m, Forêt sclerophylle (Rin’Ha, Mike) (CAS); Ankalalahana, -19.00711, 47.1337, 1350 m, *Uapaca* woodland (B.L. Fisher et al.) (CAS); Antaponimanadala I Non Protected Area, 6.59 km E Manalalondo, -19.25528, 47.1771, 1984 m, Savannah grassland (A. Ravelomanana) (CAS); Antaponimanadala II Non Protected Area, 6.63 km E Manalalondo, -19.255, 47.17684, 1955 m, Savannah grassland (A. Ravelomanana) (CAS); Navoatra I Non Protected Area, 7.64 km NW Arivonimamo, -18.97806, 47.11929, 1373 m, *Uapaca* woodland (A. Ravelomanana) (CAS); SF Manjakatompo, -19.35, 47.31667, 1600 m, montane rainforest (P.S. Ward) (CAS); SF Manjakatompo, -18.91667, 47.53333, 1300–1350 m, park/garden (P.S. Ward) (PSWC); 1 km NE Ambalavao, nr. Antananarivo, -19.08333, 47.53333, 1350 m, roadside (P.S. Ward) (CAS); 42 km S Antsirabe, -20.25, 47.1, 1500 m, *Uapaca* woodland (P.S. Ward) (CAS); Angavokely, -18.93333, 47.75 (B. Pettersson) (CAS). **Antsiranana**: Forêt de Binara, 9.4 km 235° SW Daraina, -13.26333, 49.6, 1100 m, montane rainforest (B.L. Fisher) (CAS); RS Ambre, 3.5 km 235° SW Sakaramy, -12.46889, 49.24217, 325 m, tropical dry forest edge (Fisher, Griswold et al.) (CAS); RS Ambre, 3.5 km 235° SW Sakaramy, -12.46889, 49.24217, 325 m, tropical dry forest, (J.-J. Rafanomezantsoa et al.) (CAS); RS Ankarana, 13.6 km 192° SSW Anivorano Nord, -12.86361, 49.22583, 210 m, open secondary vegetation (Fisher, Griswold et al.) (CAS); PN Marojejy, 11 km NW Manantenina, -14.45, 49.73333, 1875 m, montane rainforest (E.L. Quinter) (CAS); Marojejy, tributary Manantenina River, -14.43333, 49.75, 750 m, (Quinter & Nguyen) (CAS); Galoko chain, Mont Galoko, -13.5888, 48.72864, 980 m, montane forest (B.L. Fisher et al.) (CAS). **Fianarantsoa**: 2 km NE Anjoma-Ramartina, -19.63333, 45.96667, 750 m, grassland (P.S. Ward) (MSNG); 28 km SSW Ambositra, Ankazomivady, -20.775, 47.16833, 1670 m, grassland (B.L. Fisher) (CAS); Ampangabe II Non Protected Area, 21.29 km W Itremo, -20.61139, 46.60809, 1402 m, savannah woodland (A. Ravelomanana) (CAS); Antapia I Non Protected Area, 26.43 km SW Ambositra, -20.71972, 47.08685, 1495 m, *Uapaca* woodland (A. Ravelomanana) (CAS); Forêt d’Atsirakambiaty, 7.6 km 285° WNW Itremo, -20.59333, 46.56333, 1550 m, montane rainforest (Fisher, Griswold et al.) (CAS); Manandriana IV Non Protected Area, 26.96 km SW Ambositra, -20.73111, 47.09512, 1594 m, savannah grassland (A. Ravelomanana) (CAS); Manandriana IV Non Protected Area, 26.96 km SW Ambositra, -20.73111, 47.09512, 1594 m, Savannah grassland (A. Ravelomanana) (CAS); Miandritsara Forest, 40 km S of Ambositra, -20.79267, 47.17567, 822 m, Low altitude rainforest (Rin’Ha, Mike) (CAS); Miandritsara Forest, 40 km S of Ambositra, -20.79267, 47.17567, 822 m, Low altitude rainforest (Rin’Ha, Mike) (CAS); PN Andringitra, Plateau d’Andohariana, 39.8 km 204° Ambalavao, -22.18767, 46.90083, 2150 m, rubicole thicket at base of cliff (B.L. Fisher et al.) (CAS); Fianarantsoa, Soanierenana III Non Protected Area, 25.25 km SW Ambositra, -20.72194, 47.11019, 1707 m, savannah grassland (A. Ravelomanana) (CAS); Amoron’i Mania Region, District of Ambositra, Italaviana *Uapaca* forest, 35 km SE of Antsirabe, -20.17333, 47.086, 1359 m, *Uapaca* forest (Rin’Ha, Mike) (CAS); Amoron’i Mania Region, District of Ambositra, Italaviana *Uapaca* forest, 35 km SE of Antsirabe, -20.17333, 47.086, 1359 m, *Uapaca* forest (Rin’Ha, Mike) (CAS); Col des Tapias, -20.26667, 47.11667, 1500 m, *Uapaca* woodland (P.S. Ward) (PSWC), Vatovavy Fitovinany Region, District of Ifanadiana, 12 km W of Ranomafana, -21.25083, 47.40717, 1127 m, forest edge, open area (Rin’Ha, Mike) (PSWC), Forêt d’Atsirakambiaty, 7.6 km 285° WNW Itremo, -20.59333, 46.56333, 1550 m, grassland (Fisher, Griswold et al.) (CAS); Forêt d’Atsirakambiaty, 7.6 km 285° WNW Itremo, -20.59333, 46.56333, 1550 m, grassland (Fisher, Griswold et al.) (CAS); Manakara, -22.14817, 48.02267, 10 m, urban gardens, coastal *Casuarinaequisetifolia* (B.L. Fisher et al.) (CAS); Miandritsara Forest, 40 km S of Ambositra, -20.79267, 47.17567, 822 m, Low altitude rainforest (Rin’Ha, Mike) (CAS); PN Isalo, Ambovo Springs, 29.3 km 4° N Ranohira, -22.29833, 45.35167, 990 m, *Uapaca* woodland (Fisher, Griswold et al.) (CAS); Tsaranoro, 32.8 km 230° Ambalavao, -22.08317, 46.774, 975 m, savannah woodland (B.L. Fisher et al.) (CAS). **Mahajanga**: PN Ankarafantsika, Ampijoroa SF, 160 km N Maevatanana, deciduous forest, -16.31944, 46.81333, 43 m, deciduous forest, Mike (Irwin, Rin’ha), Harin’Hala (CAS); PN Tsingy de Bemaraha, 10.6 km ESE 123° Antsalova, -18.70944, 44.71817, 150 m, tropical dry forest on Tsingy (Fisher-Griswold Arthropod Team) (CAS); Réserve forestière Beanka, 50.7 km E Maintirano, -17.8873, 44.47113, 160 m, savannah woodland (B.L. Fisher et al.) (CAS); Sofia Region, District of Sofia, Anjiamangirana 45 km S Antsohihy, Analagnambe Galery forest, 5 km W Anjiamangirana, -15.157, 47.73417, 97 m, low degraded dry forest (Mike, Rinha) (CAS). **Toamasina**: [Antongil]; Maroantsetra, -15.43333, 49.75, 12 m, (Mocquerys) (CAS); 6 km ESE Andasibe (=Perinet), -18.95, 48.46667, 900 m, roadside (P.S. Ward) (CAS); Brickaville, -18.82183, 49.07017, 24 m, urban/garden (B.L. Fisher et al.) (CAS); Forêt Ambatovy, 14.3 km 57° Moramanga, -18.85083, 48.32, 1075 m, montane rainforest (B.L. Fisher) (CAS); Manakambahiny Atsinanana, -17.75, 48.71667, Primary forest (A. Pauly) (CAS); Forêt Ambatovy, 14.3 km 57° Moramanga, -18.85083, 48.32, 1075 m, montane shrubland, on rock (B.L. Fisher) (CAS). **Toliara**: Anosy Region, PN Andohahela, Forêt de Manatalinjo, -24.82466, 46.60111, 100 m, spiny forest/thicket (B.L. Fisher, F.A. Esteves et al.) (CAS); Betroka, -23.26558, 46.09716, 800 m, town/park/garden (B.L. Fisher et al.) (CAS). Ivahona, -23.45591, 46.17376, 820 m, village/park/garden (B.L. Fisher et al.) (CAS); 2 km SW Mahamavo, PN Andohahela, -24.78333, 46.7, 320 m, tropical dry forest (P.S. Ward) (MSNG); 34.6 km NW Tsihombe, -25.12983, 45.21033, 150 m, spiny forest/thicket, road side (B.L. Fisher et al.) (MHNG); 45 km NE Morondava, -20.05, 44.61667, 30 m, tropical dry forest (P.S. Ward) (CAS); 48 km ENE Morondava, -20.06667, 44.65, 30 m, tropical dry forest (D.M. Olson) (CAS); 4 km N Isaka-Ivondro, -24.76667, 46.86667, 180 m, roadside (P.S. Ward) (CAS); Anosy Region, District of Amboasary, 58 km SW of Fort Dauphin, 08 km NW of Amboasary, Berenty Special Reserve, -25.00667, 46.30333, 85 m, Galery forest (Rin’Ha, Mike) (CAS); Anosy Region, District of Amboasary, PN Andohahela, Parcelle III, Ihazofotsy, 32 km NE Amboasary, -24.83083, 46.53617, 58 m, dry forest, spiny forest (Michael Irwin, Frank Parker, Rin’ha) (CAS); Anosy Region, District of Amboasary, 58 km SW of Fort Dauphin, 08 km NW of Amboasary, Berenty Special Reserve, -25.021, 46.3055, 36 m, spiny forest (Mike, Rin’ha) (CAS); Anosy Region, District of Fort-Dauphin, PN Andohahela, Parcelle II, Tsimela, 42 km W of Fort-Dauphin, -24.93683, 46.62667, 176 m, transition forest (Michael Irwin, Frank Parker, Rin’ha) (CAS); Atsimo Andrefana Region, District of Betioky, 30 km E Betioky, RS Beza Mahafaly (Around Research Station), -23.6865, 44.591, 165 m, Galery dry deciduous forest (Rin’Ha, Mike) (CAS); FC Analavelona, 29.2 km 343° NNW Mahaboboka, -22.675, 44.19, 1100 m, montane rainforest (Fisher, Griswold et al.) (CAS); Forêt de Kirindy, 15.5 km 64° ENE Marofandilia, -20.045, 44.66222, 100 m, tropical dry forest (Fisher-Griswold Arthropod Team) (CAS); Forêt de Mahavelo, Isantoria River, -24.75833, 46.15717, 110 m, spiny forest/thicket (Fisher-Griswold Arthropod Team) (CAS); Mahafaly Plateau, 6.2 km 74° ENE Itampolo, -24.65361, 43.99667, 80 m, spiny forest/thicket (Fisher-Griswold Arthropod Team) (CAS); Makay Mts., -21.31364, 45.14782, 525 m, Gallery forest on sandy soil (B.L. Fisher et al.) (CAS); Manatantely, 8.9 km NW Tolagnaro, -24.9815, 46.92567, 100 m, rainforest (B.L. Fisher et al.) (CAS); Manderano, -23.5275, 44.08833, 70 m, gallery forest (Frontier Project) (CAS); PN Andohahela, Forêt d’Ambohibory, 1.7 km 61° ENE Tsimelahy, 36.1 km 308° NW Tolagnaro, -24.93, 46.6455, 300 m, tropical dry forest (Fisher-Griswold Arthropod Team) (CAS); PN Andohahela, Forêt de Manatalinjo, 33.6 km 63° ENE Amboasary, 7.6 km 99° E Hazofotsy, -24.81694, 46.61, 150 m, spiny forest/thicket (Fisher-Griswold Arthropod Team) (CAS); PN Tsimanampetsotsa, Forêt de Bemanateza, 20.7 km 81° E Efoetse, 23.0 km 131° SE Beheloka, -23.99222, 43.88067, 90 m, spiny forest/thicket (Fisher-Griswold Arthropod Team) (CAS); PN Zombitse, 19.8 km 84° E Sakaraha, -22.84333, 44.71, 770 m, tropical dry forest (Fisher, Griswold et al.) (CAS); Portuguese Island, nr. Fort Dauphin; Ifarantsa; Taolagnaro, -24.9167, 46.8667, 30 m, lawn, D. Whitacre (CAS); RS Beza Mahafaly, Parcel 1, -23.65, 44.63333, 130 m, tropical dry forest (P.S. Ward) (CAS); Réserve Berenty, -25.01667, 46.3, 25 m, tropical dry forest (P.S. Ward) (CAS); Réserve Privé Berenty, Forêt de Bealoka, Mandraré River, 14.6 km 329° NNW Amboasary, -24.95694, 46.2715, 35 m, gallery forest (Fisher-Griswold Arthropod Team) (CAS); Sakaraha, -22.91233, 44.53283, 470 m, urban/garden (B.L. Fisher et al.) (CAS); Sept Lacs, -23.52083, 44.15972, 120 m, gallery forest (Frontier Project) (CAS); 27 km N Tongobory, Rupelian zone in dry forest, -23.34617, 44.35817, 290 m, spiny scrubland damp wash (M.E. Irwin, Rin’ha) (CAS); PN Andohaela Tsimelahy, -24.93683, 46.62667, 180 m, transition forest (M.E. Irwin, F.D. Parker, R. Harin’Hala) (CAS); Berenty Special Reserve, 8 km NW Amboasary, 58 km SW of Fort Dauphin, -25.00667, 46.30333, 85 m, gallery forest (M.E. Irwin, F.D. Parker, R. Harin’Hala) (CAS); PN Andohahela, Ihazofotsy - Parcel III, transition forest, -24.83483, 46.48683, 80 m, tropical dry forest, transition between spiny and dry deciduous forests (M.E. Irwin, F.D. Parker, R. Harin’Hala) (CAS); Parcel I, RS Beza Mahafaly, near research station, -23.6865, 44.591, 165 m, dry deciduous forest (R. Harin’Hala) (CAS); Parcel II, RS Beza Mahafaly, near Bellevue, -23.68983, 44.5755, 180 m, spiny forest (R. Harin’Hala) (CAS).

##### Diagnosis.

In full-face view, lateral margins of head anterior to eye level diverging posteriorly; anterior clypeal margin truncate; white spots absent on second and third abdominal tergite.

##### Description.

**Minor worker.** With head in full-face view, lateral margins diverging towards almost straight posterior margin; eye slightly convex and small (EL/CS: 0.25±0.01; 0.23–0.28), not interrupting lateral cephalic border, level of its posterior margin located at ca. posterior 1/4 of head (PoOc/CL: 0.26±0.02; 0.22–0.28); frontal carinae wide, posteriorly diverging (FR/CS: 0.32±0.01; 0.30–0.33), distance between them larger than their smallest distance to eye; clypeus with bluntly angulate anterolateral corner and straight anteromedian margin; two apical teeth of mandible normally distant; antennal scape relatively short (SL/CS: 1.28±0.05; 1.19–1.34). Promesonotum evenly convex; posterior portion of mesonotum flat immediately anterior to weakly visible metanotal groove; propodeal dorsum strongly concave medially; junction of dorsal margin of propodeum with declivity bluntly angulate; propodeal dorsum ca. 2 × as long as declivity. Petiole nodiform, its dorsal margin inclined posteriorly and rounding to anterior margin; anterior and posterior faces almost the same height; femur of hind leg rounded axially, not twisted basally.

First and second gastral tergites without a pair of white spots; erect hairs on lateral margin of head absent; two erect hairs present near posterior cephalic margin; antennal scape with appressed hairs only; pronotum and mesonotum with a pair of erect hairs each, posterodorsal angle of propodeum with a pair of erect hairs. Integument shining; body color dark brown; distal portion of leg reddish brown.

**Major worker.** Characteristics the same as minor worker, except the enlarged head (CS: 2.32±0.12; 2.13–2.46; CWb/CL: 1.00±0.03; 0.96–1.04), with slight concavity of posterior margin; anteromedian margin of clypeus noticeably concave; apical 1/4 of antennal scape extending beyond posterior cephalic margin; robust mesosoma, promesonotum convex, propodeal dorsum straight immediately posterior to metanotum, feebly concave towards declivity, length the same as height of declivity; petiolar node tapering dorsally.

##### Distribution and biology.

**Figure 60.**
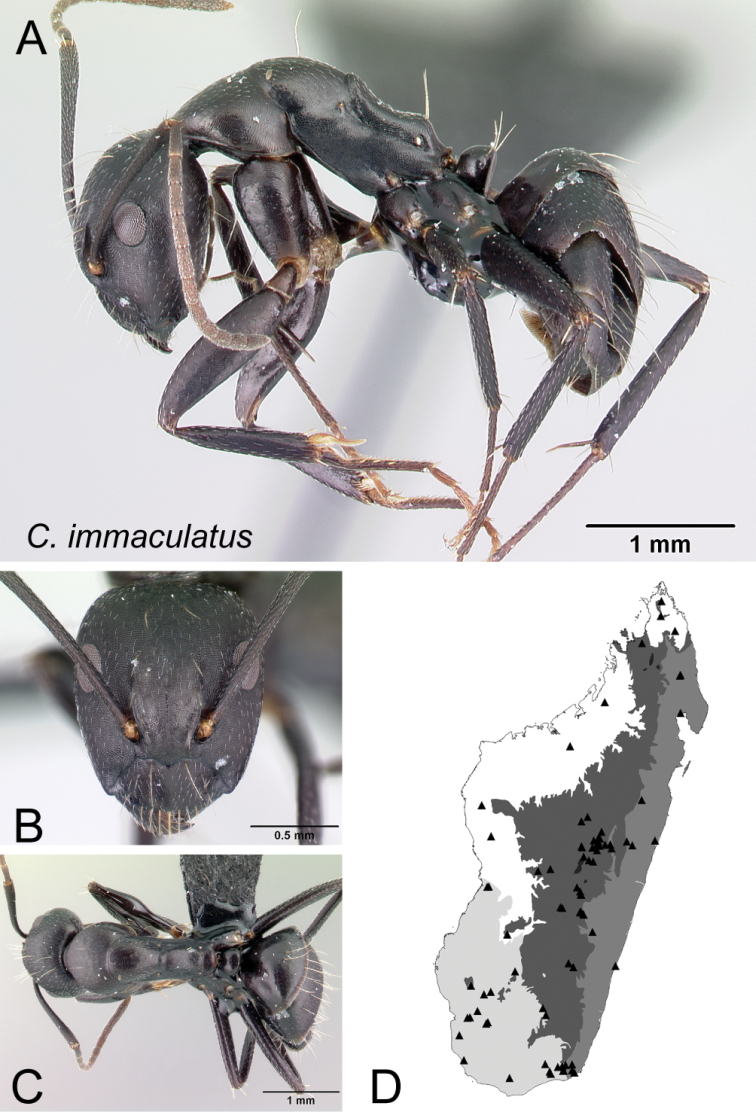
*Camponotusimmaculatus***A** lateral view **B** head in full-face view **C** dorsal view of minor worker CASENT0082438**D** distribution map.

Occupying the high plateau of Madagascar, *C.immaculatus* occurs in shrubland and grassland areas, montane rainforest, savannah grassland, and *Uapaca* woodland habitats (Fig. 60D). It is also known from rainforest and forest transitioning to montane forest in the southeast, and from human-modified habitats in the west, south, and high plateau of the island. Nest sites are typically established in the ground, under stones, and under rotten logs, while foraging is carried out in the leaf litter, on the ground, and on lower vegetation.

##### Discussion.

*Camponotusimmaculatus* can be easily separated from *C.kelimaso* and *C.lubbocki* by the presence of the broad concavity on the propodeal dorsum. It also can be distinguished from other species because the anteromedian margin of its clypeus is truncate and no white spots are visible on the abdominal tergites.

When describing *C.quadrimaculatusopacata*, Emery (1925) did not make any comparative study with *C.immaculatus*. He stated no morphological characters that separate the former species from the later, and our examination of the syntype specimens of both taxa indicates that morphological variation observed in the former is found across the large geographical distribution of the latter. Hence, there is no doubt to make *C.quadrimaculatusopacata* under synonymy here.

The delimitation argument for *C.immaculatus* is strengthened by the congruence between the results of traditional qualitative morphology and the NC-clustering method. However, LDA produces a high 8.4% error rate in the classification of *C.immaculatus*, due to a misidentification of a single minor worker as *C.quadrimaculatus*. The high error rate is due in large part to the small range of minor worker forms of *C.immaculatus* available for this study.

#### ﻿ Camponotus joany sp. nov.

Taxon classificationAnimaliaHymenopteraFormicidae

E49CFDE2-67F6-576A-AF4C-24513692CA8A

http://zoobank.org/77C29D20-D734-49AE-829D-FF228FEDC2D6

Figs 2A , 4B , 61

##### Holotype worker.

**Madagascar**: Province **Antsiranana**: Montagne des Français, 7.2 km 142° SE Antsiranana (=Diego Suarez), -12.32278, 49.33817, 180 m, tropical dry forest, 11 Apr 2009 (Fisher, Griswold et al.) collection code: BLF03132, specimen code: CASENT0408908 (CAS).

##### Paratypes.

2 minor workers with same data as holotype but respectively specimen coded as: CASENT0408907, CASENT0408909 (PBZT, CAS).

##### Additional material examined.

**Madagascar: Antsiranana**: Montagne des Français, 7.2 km 142° SE Antsiranana (=Diego Suarez), -12.32278, 49.33817, 180 m, tropical dry forest (Fisher, Griswold et al.) (CAS).

##### Diagnosis.

In full-face view, lateral margins of head anterior to eye level approximately parallel and covered with erect hairs; two apical teeth of mandible normally spaced; mesonotum short and lacking constriction; propodeal dorsum approximately straight, 2 × as long as declivity; dorsal margin of petiole shorter than posterior margin.

##### Description.

**Minor worker.** In full-face view, lateral margins of head anterior to eye level approximately parallel, rounding progressively towards a slightly concave posterior margin; eye convex and large (EL/CS: 0.27±0.00; 0.27–0.28), not breaking lateral cephalic margin, location of its posterior margin at posterior 1/4 of head (PoOc/CL: 0.26±0.00); frontal carinae parallel, not widely opened posteriorly (FR/CS: 0.27±0.0); clypeus lacking anterolateral angle, anteromedian margin broadly convex; two apical teeth of mandible normally spaced; antennal scape relatively long (SL/CS: 1.62±0.01; 1.61–1.63). Dorsal outline of mesosoma approximately evenly convex; metanotal groove visible; propodeal dorsum straight, ca. 2 × as long as declivity, joining it at a blunt angle. Petiole nodiform, its dorsal margin shorter than anteriorly inclined rear margin and joining the anterior margin at a blunt angle.

First and second gastral tergites without a pair of white spots; short erect hairs on lateral margin of head present; near posterior margin of head with two elongate erect hairs; antennal scape covered with erect hairs; junction of propodeal dorsum and declivity with one pair of erect hairs.

**Major worker.** Unknown.

##### Distribution and biology.

**Figure 61.**
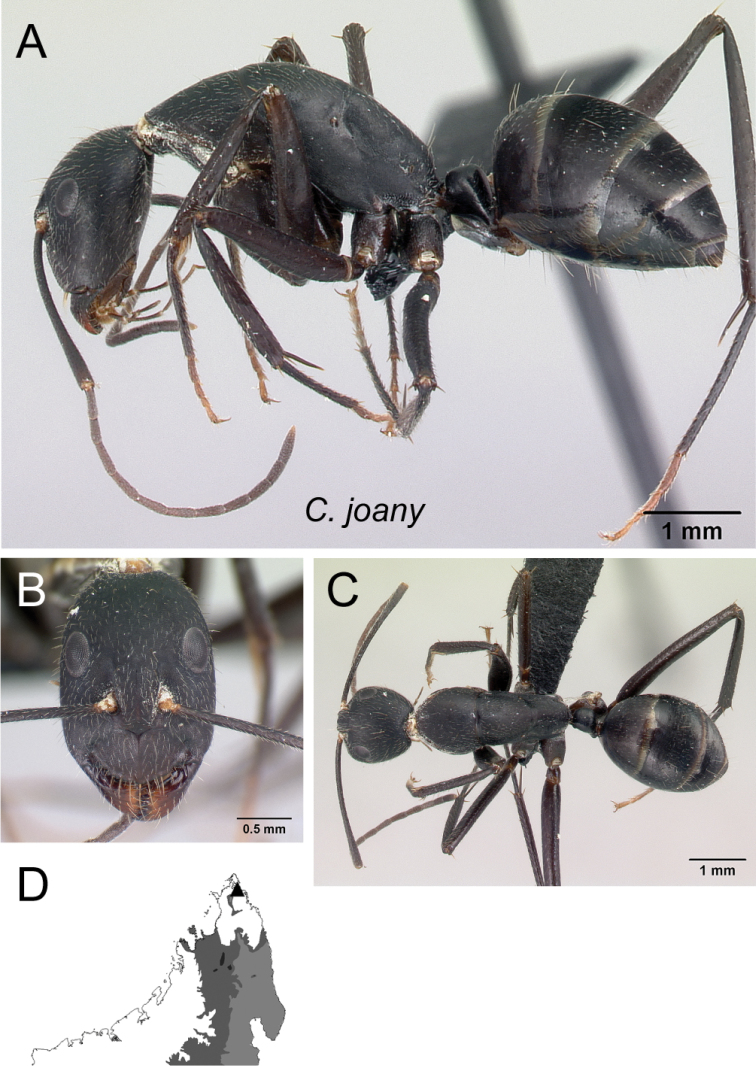
*Camponotusjoany***A** lateral view **B** head in full-face view **C** dorsal view of holotype minor worker CASENT0408908**D** distribution map.

*Camponotusjoany* is geographically restricted to the dry forest of Montagne de Francais in the north of Madagascar (Fig. 61D). Its nest sites are unknown and workers have been found foraging on lower vegetation.

##### Discussion.

See discussion under *C.aina*.

##### Etymology.

The species name *joany* is a non-Latin singular noun used in apposition.

#### ﻿ Camponotus karsti sp. nov.

Taxon classificationAnimaliaHymenopteraFormicidae

FD0748C7-6908-5176-A1B9-770C37040519

http://zoobank.org/A47440D3-8DC7-474C-88A4-9F060DF006C2

Figs 5A , 62

##### Holotype worker.

**Madagascar**: Province **Antsiranana**: RS Ankarana, 7 km SE Matsaborimanga, -12.9, 49.11667, 150 m, tropical dry forest, low vegetation, 27 Nov 1990 (P.S. Ward) collection code: PSW11005, specimen code: CASENT0217292 (CAS).

##### Paratype.

1 minor worker with same data as holotype but respectively specimen coded as: CASENT0837940 (CAS).

##### Additional material examined.

**Madagascar: Antsiranana**: RS Ankarana, 7 km SE Matsaborimanga, -12.9, 49.11667, 150 m, Dry Forest, (P.S. Ward) (CAS).

##### Diagnosis.

With head in full-face view, eye not breaking lateral cephalic margin; mesonotum short and lacking constriction; promesonotum an even convexity; propodeum with blunt angle at ca. posterior 1/2; anterior margin of petiolar node in profile very low, dorsal surface noticeably horizontal, ca. 2 × as long as posterior surface.

##### Description.

**Minor worker.** With head in full-face view, lateral margins anterior to eye level approximately parallel, progressively rounding evenly towards slightly concave rear margin; eye protruding and large (EL/CL: 0.26±0.01; 0.25–0.26), not breaking lateral cephalic margin, location of its posterior margin at posterior 1/4 of head (PoOc/CL: 0.26±0.01; 0.25–0.27); frontal carinae parallel, not widely opened posteriorly (FR/CS: 0.25); clypeus with blunt or poorly defined anterolateral angle, anteromedian margin broadly convex; two apical teeth of mandible normally spaced; antennal scape relatively long (SL/CS: 1.65±0.03; 1.63–1.67). Promesonotum noticeably convex, followed by a slightly visible metanotal groove; anterior portion of propodeal dorsum sloping along its first 1/2 then slightly convex and rounding to declivity surface. Anterior margin of petiolar node ca. 1/2 height of posterior margin, dorsal margin separated by an angle from anterior margin and inclined posteriorly to jointly rounding to posterior margin.

First and second gastral tergites without a pair of white spots; erect hairs on lateral margin of head present on anterior and behind eye level; posterior margin of head with two elongate, erect hairs; antennal scape covered with appressed hairs; junction of propodeal dorsum and declivity with two pairs of erect hairs.

**Major worker.** Unknown.

##### Distribution and biology.

**Figure 62.**
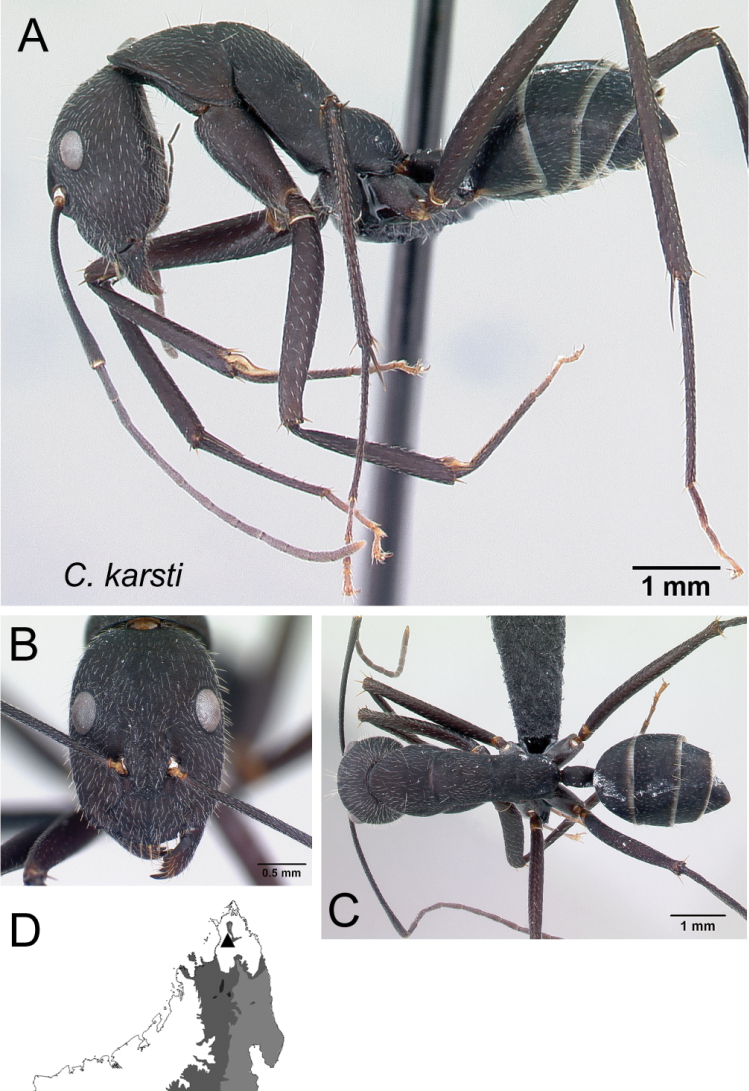
*Camponotuskarsti***A** lateral view **B** head in full-face view **C** dorsal view of holotype minor worker CASENT0217292**D** distribution map.

The distribution of *C.karsti* is limited to the dry forest of the RS Ankarana in the north of Madagascar (Fig. 62D). Nest site for this species is unknown, but foraging is carried out on lower portions of vegetation.

##### Discussion.

*Camponotuskarsti* can be confounded with *C.harenarum* because both species are characterized by the presence of a blunt angle at approximately the posterior 1/2 of propodeal dorsum. However, *C.harenarum* can be distinguished by the separate convexity of its mesonotum and anterior 1/2 of propodeum, and its petiolar node is approximately as high as long.

##### Etymology.

The species name *karsti* is a non-Latin singular noun used in apposition.

#### ﻿ Camponotus kelimaso sp. nov.

Taxon classificationAnimaliaHymenopteraFormicidae

E59160DA-8FA6-5AB8-B878-429A200E7150

http://zoobank.org/BDE5DB12-AFBD-41F7-95F5-966AF8E790F3

**Figure 63.**
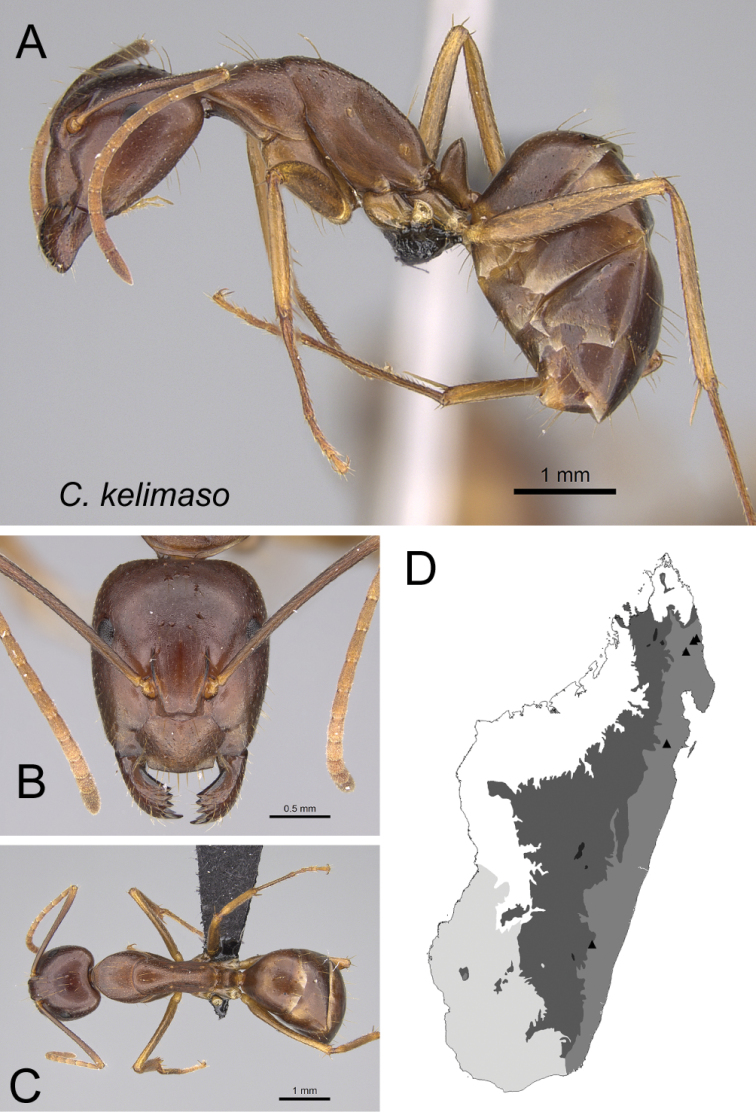
*Camponotuskelimaso***A** lateral view **B** head in full-face view **C** dorsal view of holotype minor worker CASENT0487718**D** distribution map.

Figs 34B , 35A , 37A , 63

##### Holotype worker.

**Madagascar**: Province **Antsiranana**: PN Marojejy, Manantenina River, 27.6 km 35° NE Andapa, 9.6 km 327° NNW Manantenina, -14.435, 49.76, 775 m, rainforest, ex rotten log, 16 Nov 2003 (B.L. Fisher et al.) collection code: BLF09010, specimen code: CASENT0487718 (CAS).

##### Paratypes.

3 minor workers of same data as holotype but specimens coded as: CASENT0837637, CASENT0837636, CASENT0837635 (NHMUK, PBZT, CAS).

##### Additional material examined.

**Madagascar: Antsiranana**: Makirovana forest, -14.16666, 49.95, 715 m, rainforest (B.L. Fisher et al.) (CAS); Makirovana forest, -14.10295, 50.01984, 390 m, rainforest (B.L. Fisher et al.) (CAS); PN Marojejy, Manantenina River, 27.6 km 35° NE Andapa, 9.6 km 327° NNW Manantenina, -14.435, 49.76, 775 m, rainforest (B.L. Fisher) (CAS). **Fianarantsoa**: Forêt d’Ambalagoavy Nord, Ikongo, Ambatombe, -21.857068, 47.37849, 625 m (R. Harin’Hala & M.E. Irwin) (CAS). **Toamasina**: RS Ambatovaky, Sandrangato river, -16.7755, 49.26427, 430 m, rainforest (B.L. Fisher et al.) (CAS) RS Ambatovaky, Sandrangato river, -16.7702, 49.26638, 470 m, rainforest (B.L. Fisher et al.) (CAS).

##### Diagnosis.

In full-face view, lateral margins of head anterior to eye level diverging posteriorly; anterior clypeal margin truncate; dorsum of second and third abdominal tergites lacking white spot; propodeal dorsum straight; body color dark brown; antennal scape without erect hairs.

##### Description.

**Minor worker.** In full-face view, head sides diverging towards anterior to level of eye, evenly rounding to approximately straight posterior margin; head widest at eye level; eyes almost flattened and small (EL/CS: 0.19±0.01; 0.16–0.21), not breaking lateral cephalic margin, level of posterior margin located approximately at posterior 1/3 of head (PoOc/CL: 0.28±0.01; 0.27–0.29); frontal carinae more or less wide (FR/CS: 0.31±0.02; 0.28–0.34), posteriorly diverging, distance between them smaller than their smallest distance to eye; clypeus with anterolateral angle and approximately straight anteromedian margin; mandible with two apical teeth distantly spaced; antennal scape relatively short (SL/CS: 1.19±0.05; 1.11–1.27). Promesonotum weakly convex; mesonotum with posterior portion flat immediately anterior to weakly visible metanotal groove; propodeal dorsum almost straight, its angle to declivity widely rounded; propodeal dorsum ca. 2 × as long as declivity. Petiolar node flattened anteroposteriorly, without obvious dorsal margin; anterior face almost the same height as posterior face; femur of hind leg rounded axially, not twisted basally.

First and second gastral tergites without a pair of white spots; lateral cephalic margin with erect hairs anterior to level of eyes; no erect hairs on lateral margin of head posterior to eye level; two erect hairs present near posterior margin; antennal scape only covered with appressed hairs; pronotum with few erect hairs; mesonotum with a pair of erect hairs; two erect hairs present on anterior to posterodorsal corner of propodeum. Body color reddish brown to pale brown.

**Major worker.** Differing from minor worker in the following characters: enlarged head (CS: 2.83±0.25; 2.35–3.15; CWb/CL: 0.99±0.04; 0.92–1.04) with broadly concave posterior margin; apical 1/4 of antennal scape surpassing posterior cephalic margin; robust mesosoma with separate convexity of promesonotum, lower position of metanotum and propodeum, propodeal dorsum approximately the same length as declivity surface and with rounded junction; petiolar node more flattened anteroposteriorly.

##### Distribution and biology.

The distribution of *C.kelimaso* is limited to the eastern lowland rainforest of Madagascar between the Makirovana forest in the north and the Forêt d’Ambalagoavy Nord in the south (Fig. 63D). It nests mainly in rotten logs and rarely in root mat layers in the ground. Foraging is carried out on the ground and through leaf litter.

##### Discussion.

*Camponotuskelimaso* can be separated from *C.immaculatus* by its straight propodeal dorsum. It can be differentiated from *C.lubbocki* by the approximately straight posterior margin of the head and its small compound eyes.

Based on the information provided by the NC-clustering method, the grouping of *C.kelimaso* supports the distinction of the species by conventional qualitative taxonomy. The confirmatory LDA identified the samples successfully at 100%.

##### Etymology.

The species name *kelimaso* is a non-latin word derived from the Malagasy word for “small eye”. It refers to the fact that its compound eyes are small compared to those of other species in the subgenus Myrmosaga.

#### ﻿ Camponotus liandia

Taxon classificationAnimaliaHymenopteraFormicidae

Rakotonirina & Fisher

B7F12144-798D-5EFC-B31B-45E7A5E21573

**Figure 64.**
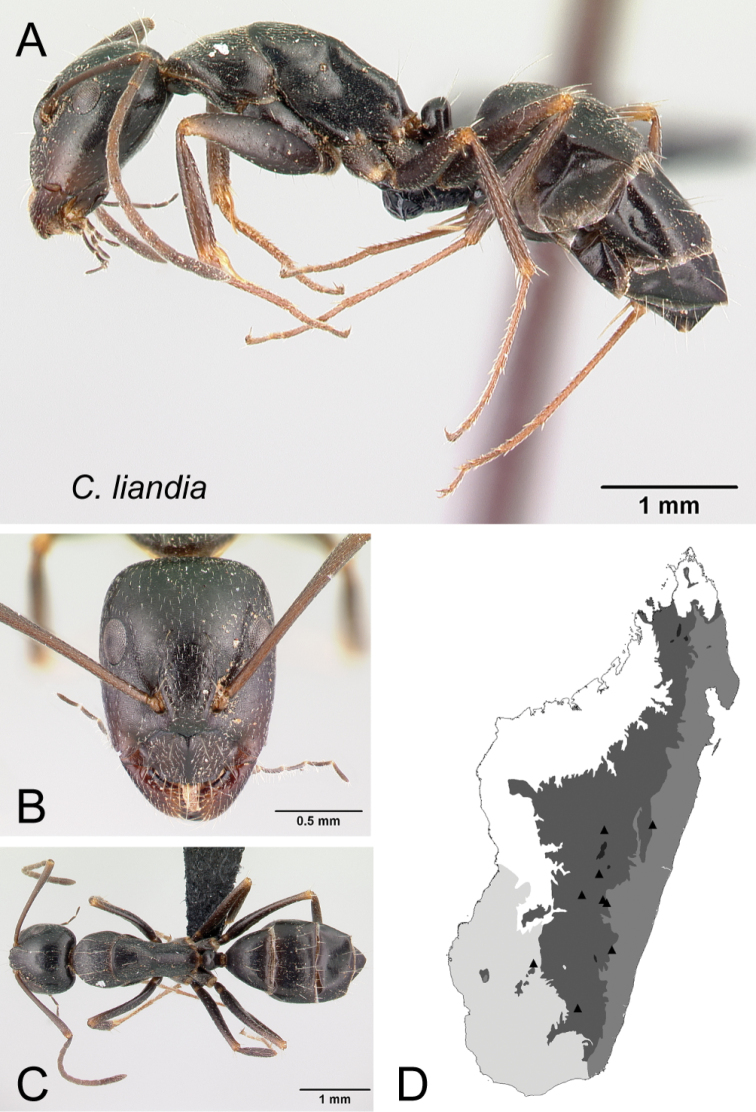
*Camponotusliandia***A** lateral view **B** head in full-face view **C** dorsal view of minor worker CASENT0491145**D** distribution map.

Figs 34A , 64 (See Rakotonirina and Fisher 2018 for the description)

##### Diagnosis.

In full-face view, lateral margins of head anterior to eye level diverging posteriorly; anterior clypeal margin broadly triangular.

##### Distribution and biology.

In the present study, more samples have been attributed to *C.liandia*. The species is generally known from coastal scrub and littoral forest in the east, montane rainforest, grassland, shrubland, savannah grassland, woodland, and *Uapaca* woodland on the central high plateau (Fig. 64D). It has also colonized dry forest on tsingy and burned savannah in the west, degraded forest below granite outcroppings, and montane shrubland on rock in the high plateau. Nests are established in rotten logs, under stones, and in the ground. Workers may forage on the ground and on low vegetation.

##### Discussion.

With the additional samples, *C.liandia* presents two morphological variations: a matte integument covered with microreticulate sculpture, and a smooth and shining body. The first form has been observed from samples collected from the Forêt d’Atsirakambiaty in the grassland of the south-central high plateau. The second form occupies coastal scrub and littoral forest in the east and dry forest on Tsingy and burned savannah in the west.

*Camponotusliandia* was originally described under the subgenus Mayria (Rakotonirina and Fisher 2018) but the presence of a median carina on a broadly projected rectangular clypeus and a dorsally tapering petiolar node that are a few of the strong features characterizing the subgenus Myrmosaga has led to the placement of the species to this latter subgenus. The subgenus Mayria has generally rounded anterior clypeal margin and a nodiform petiole.

The cluster for *C.liandia* is composed of a larger number of worker samples to include the qualitative morphological variation within the species. The results of the exploratory analysis using NC-clustering methods are congruent with the definition of *C.liandia* based on traditional qualitative morphology. Identification of this species is confirmed by LDA with 100% success.

#### ﻿ Camponotus lokobe sp. nov.

Taxon classificationAnimaliaHymenopteraFormicidae

FCC09314-6C22-58F5-ADA3-7FAF9BA816FF

http://zoobank.org/E71343D8-F1F7-43BC-A658-AD47874A36A6

Figs 8C , 11A , 65

##### Holotype worker.

**Madagascar**: Province **Antsiranana**: RNI Lokobe, 6.3 km 112° ESE Hellville, Nosy Be, -13.41933, 48.33117, 30 m, rainforest, ex rotten log, 19–24 Feb 2001, (Fisher, Griswold et al.) collection code: BLF03476, specimen code: CASENT0436584 (CAS).

##### Paratypes.

2 workers of same data as holotype but specimen coded as: CASENT0835595 (PBZT, CAS).

##### Additional material examined.

**Madagascar: Antsiranana**: Nosy Be, RNI Lokobe, 6.3 km 112° ESE Hellville, -13.41933, 48.33117, 30 m, rainforest (Fisher, Griswold et al.) (CAS).

##### Diagnosis.

With head in full-face view, lateral cephalic margins converging posteriorly towards eye level, then rounding to occipital corner of head; anteromedian margin of clypeus noticeably excised medially; two apical teeth of mandible normally spaced; lateral cephalic margin anterior to eye level covered with erect hairs.

##### Description.

**Minor worker.** With head in full-face view, lateral cephalic borders anterior to level of eye parallel to each other, converging strongly towards posterior margin behind eye level; eye large and convex (EL/CS: 0.30±0.01; 0.27–0.31), breaking lateral margin of head, level of its posterior margin located approximately at posterior 1/3 of head (PoOc/CL: 0.29±0.01; 0.28–0.31); frontal carinae not widely opened (FR/CS: 0.26±0.01;0.25–0.27), posteriorly parallel, distance between them larger than smallest distance to eye; clypeus with anterolateral angle and medially slightly concave anteromedian margin; two apical teeth of mandible distantly spaced; antennal scape relatively long (SL/CS: 1.80±0.03; 1.76–1.85). Mesosoma long and low (MPH/ML: 0.29±0.01; 0.29–0.30), with its dorsal outline almost flat; metanotal groove feebly visible; propodeal dorsum feebly convex anteriorly and flat posteriorly; junction of propodeal dorsum to declivity bluntly angulate; propodeal declivity 1/3 length of the dorsum. Petiole nodiform, tapering dorsally; its dorsal margin inclined posteriorly and forming a blunt angle to anterior face; posterior face 2 × as high as the anterior; femur of hind leg rounded axially, without twist near base.

First and second gastral tergites without a pair of white spots; with head in full-face view lateral margin covered with erect hairs; near posterior margin of head with a pair of erect hairs; antennal scape covered with erect hairs inclined at ca. 45°; pronotum with numerous erect to suberect hairs; a pair of erect hairs present on posterodorsal angle of propodeum.

**Major worker.** Unknown.

##### Distribution and biology.

**Figure 65.**
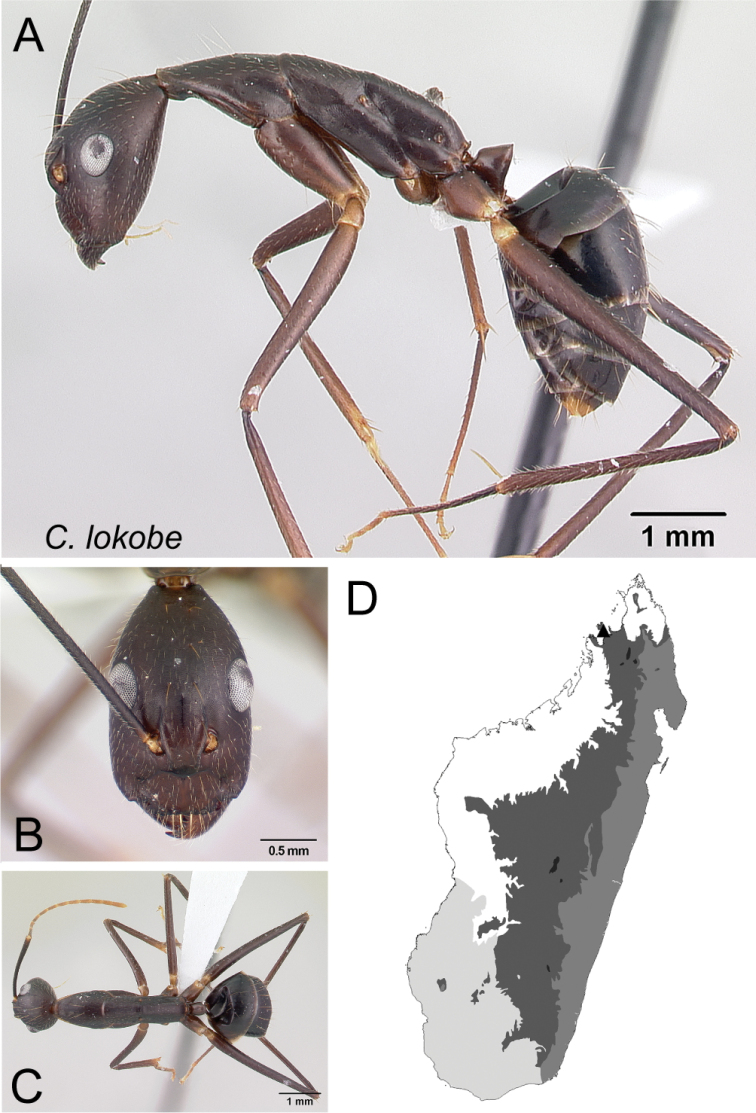
*Camponotuslokobe***A** lateral view **B** head in full-face view **C** dorsal view of holotype minor worker CASENT0436584**D** distribution map.

*Camponotuslokobe* is endemic to Madagascar and restricted to the rainforest of the RNI Lokobe in the north of the island (Fig. 65D). Nest sites have been found in rotten logs.

##### Discussion.

*Camponotuslokobe* can be separated from *C.dufouri* and *C.tendryi* by the fact that its clypeus is characterized by a medially excised anteromedian margin.

The grouping of *C.lokobe* in the same cluster shown by the dendrogram of multivariate morphometric analysis is confirmed by the cumulative LDA at 100% identification success, corroborating the species hypothesized by the taxonomic revision based on qualitative morphology.

##### Etymology.

The species name *lokobe* is a singular non-Latin noun used in apposition and refers to the type locality.

#### ﻿ Camponotus lubbocki

Taxon classificationAnimaliaHymenopteraFormicidae

Forel

FF9302A0-0498-5DD3-9D1D-45B823B1B189

Figs 36B , 37B (See Rakotonirina and Fisher 2018 for the taxonomic history and redescription of the species)

##### Diagnosis.

In full-face view, lateral margin of head anterior to eye level diverging posteriorly; anterior clypeal margin truncate; no white spot on dorsum of second and third abdominal tergites; body color black; antennal scape without erect hairs, propodeal dorsum slightly concave.

##### Distribution and biology.

See the geographic distribution and biology in Rakotonirina and Fisher (2018).

##### Discussion.

*Camponotuslubbocki* might be confused with *C.liandia* but the latter has a broadly convex anteromedian margin of the clypeus. It looks similar to *C.immaculatus* and *C.kelimaso* because they too lack white spots on the second and third abdominal segments, but in *C.immaculatus* the propodeal dorsum is transversely concave and in *C.kelimaso* the eyes are small and the posterior cephalic margin is approximately straight.

Forel (1912) originally placed *C.lubbocki* under the subgenus Myrmosaga, which was later synonymized by Emery (1925) under *Mayria* (see also Rakotonirina and Fisher 2018). Whereas this species was recently redescribed under *Mayria*, it is morphologically similar to those within *Myrmosaga* because of its broadly projected rectangular clypeus that is medially carinate, and a more or less anteroposteriorly flattened petiolar node. The subgenus Mayria is characterized by the clypeus that has no median carina, but with a rounded anterior margin, and a nodiform petiole. Therefore, *C.lubbocki* is moved back to the subgenus Myrmosaga here.

The species delimitation of *C.lubbocki* based on qualitative morphology is sustained by the NC-clustering method. Identification of this species is confirmed by LDA with 100% success (see also Rakotonirina and Fisher 2018).

#### ﻿ Camponotus mahafaly sp. nov.

Taxon classificationAnimaliaHymenopteraFormicidae

A11627C7-4A08-512D-AFE3-B8632BA87703

http://zoobank.org/68F229F9-571D-4B71-8F98-0B89218D660E

**Figure 66.**
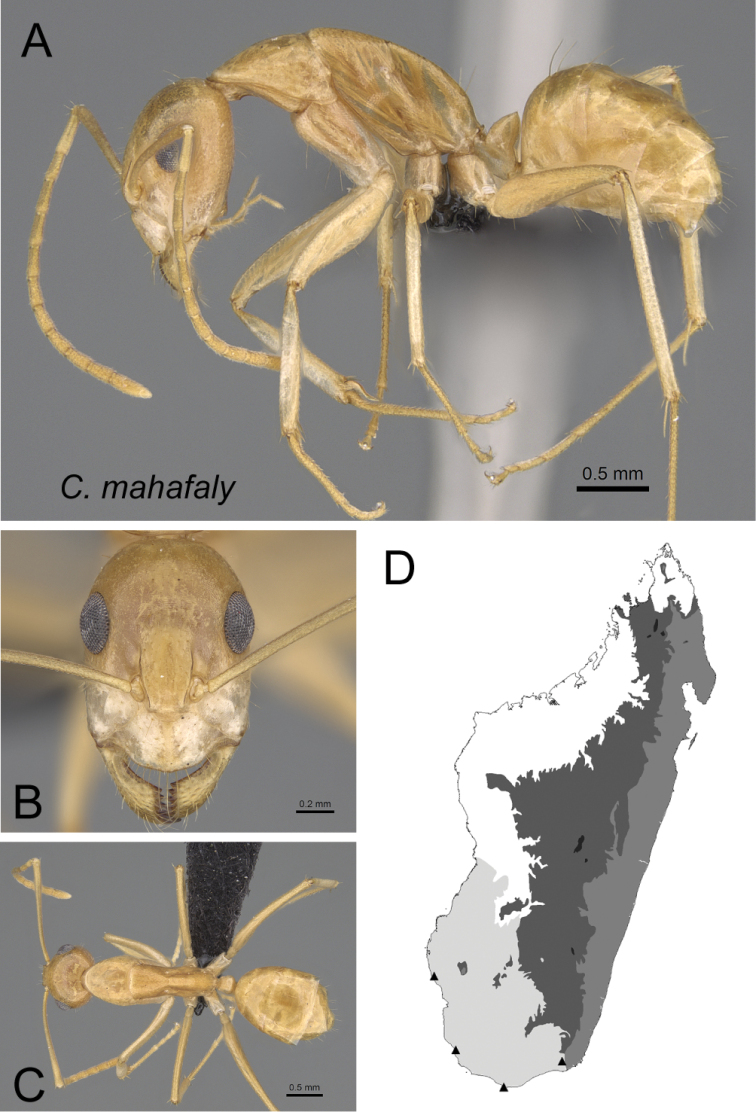
*Camponotusmahafaly***A** lateral view **B** head in full-face view **C** dorsal view of minor worker CASENT0115287**D** distribution map.

Figs 15A , 16B , 20A , 66

##### Holotype worker.

**Madagascar**: Province **Toliara**: Androy Region, District of Tsihombe, 74 km S of Tsihombe, Reserve Speciale Cap Ste Marie, -25.58767, 45.163, 36 m, spiny bush, Malaise trap, 30 Apr–11 May 2003, (Rin’ha, Mike) CASENT0115206 (CAS).

##### Paratypes.

2 minor workers of same data as holotype but respectively with collection codes: MG-23-34 and MG-23-33 and specimen codes: CASENT0115287 and CASENT0115718 (CAS).

##### Additional material examined.

**Madagascar: Toliara**: Androy Region, District of Tsihombe, 74 km S of Tsihombe, RS Cap Ste Marie, -25.58767, 45.163, 36 m, spiny bush, (Mike, Frank Parker, Rin’ha) (CAS); Androy Region, District of Tsihombe, 74 km S of Tsihombe, RS Cap Ste Marie, -25.58767, 45.163, 152 m, Bush (Mike, Rin’ha) (CAS); Anosy Region, District of Fort-Dauphin, PN Andohahela, Parcelle II, Tsimela, 42 km W of Fort-Dauphin, -24.93683, 46.62667, 176 m, transition forest (Michael Irwin, Frank Parker, Rin’ha) (CAS); Forêt de Tsinjoriaky, 6.2 km 84° E Tsifota, -22.80222, 43.42067, 70 m, spiny forest/thicket (Fisher-Griswold Arthropod Team) (CAS); RS Cap Sainte Marie, 12.3 km 262° W Marovato, -25.58167, 45.16833, 200 m, spiny forest/thicket (Fisher-Griswold Arthropod Team) (CAS); 5 km E Itampolo, malaise across path of plateau of Andrimpano Forest, -24.65033, 43.96317, 130 m, dry forest (M.E. Irwin, Rin’ha) (CAS).

##### Diagnosis.

With head in full-face view, lateral margins of head anterior to eye level parallel and lacking erect hairs, lateral and anteromedian clypeal margins continuously forming broad convexity; scape without erect hairs; mesosoma long and low; body color yellowish to brown.

##### Description.

**Minor worker.** In full-face view, head sides anterior to level of eye parallel, rounding evenly to posterior margin behind eye level; eye protruding and large (EL/CS: 0.32±0.01; 0.30–0.35), breaking lateral cephalic margin, level of its posterior margin situated approximately at posterior 1/4 of head (PoOc/CL: 0.23±0.01; 0.20–0.24); frontal carinae close to each other (FR/CS: 0.27±0.01; 0.25–0.28), posteriorly parallel, distance between them smaller than their smallest distance to eye; clypeus without well-defined anterolateral angle, its anteromedian margin broadly convex; mandible with two apical teeth normally spaced; antennal scape relatively long (SL/CS: 1.40±0.04; 1.33–1.45). Promesonotum weakly convex; mesonotum with posterior portion flat immediately anterior to weakly visible metanotal groove; propodeal dorsum almost straight (MPH/ML: 0.37±0.03; 0.34–0.50); rounding to declivity, 2 × as long as declivity. Petiolar node short and high, with dorsal margin inclined posteriorly, rounding to anterior margin; height of anterior face 2/3 of that of posterior face; femur of hind leg rounded axially, without twist near base.

First and second gastral tergites without a pair of white spots; lateral margin of head anterior to eye level, with no erect hairs posterior to eye level; two erect hairs present close to posterior margin; antennal scape covered only with appressed hairs; erect hairs lacking on promesonotum; posterodorsal corner of propodeum with a pair of erect hairs. Integument shining; body color yellowish.

**Major worker.** Unknown.

##### Distribution and biology.

*Camponotusmahafaly* is geographically restricted to dry forest and spiny bush and forest habitats of the southwest and south of Madagascar (Fig. 66D). Nest sites are generally located in rotten logs, and foraging occurs on the surface of the ground.

##### Discussion.

*Camponotusmahafaly* might be confounded with *C.cemeryi* because the petiole of both species is not nodelike, but in the latter species the antennal scape is covered with suberect hairs and the mesosoma is generally short and high, with a dorsal outline that is strongly convex.

The qualitative morphology-based study of this species agrees with the multivariate morphometrics to support the taxonomic determination of *C.mahafaly*. Separation of this species is confirmed by LDA with 100% success.

##### Etymology.

The species name *mahafaly* is a singular non-Latin noun used in apposition and refers to the Mahafaly region in the southwest of Madagascar.

#### ﻿ Camponotus mixtellus

Taxon classificationAnimaliaHymenopteraFormicidae

Forel stat. nov.

3C7C6B62-F27D-5044-A8AA-3EAF06F732A6

Figs 22B , 23A , 67

Camponotus radamae mixtellus Dalla Torre, 1893: 249 Lectotype minor worker, by present designation, Madagascar, Forêt des bords de l’Ivondrona, près de Tamatave (Dr Conrad Keller) AntWeb CASENT0101893 (MHNG) [examined]. Paralectotype major worker, Madagascar (André) CASENT0101924 (MHNG) [examined]. [Camponotus (Myrmoturba) radamae var. mixtellus Dalla Torre, 1893: 249 - First available use of Camponotusmaculatusradamaemixtellus Forel, 1891: 33; unavailable name]. Stat. nov.

##### Additional material examined.

**Comoros: Anjouan**: Mount Ntringui, -12.19865, 44.41866, 740 m, montane forest (B.L. Fisher et al.) (CAS). **Grande Comore**: Karthala, -11.82498, 43.4374, 795 m, disturbed forest (B.L. Fisher et al.) (CAS). **Mohéli**: Lac Boundouni, -12.37915, 43.85165, 25 m, dry forest (B.L. Fisher et al.) (CAS); Ouallah, -12.30353, 43.66827, 500 m, rainforest (B.L. Fisher et al.) (CAS); Ouallah, -12.296, 43.676, 750 m, montane rainforest (B.L. Fisher et al.) (CAS). **Madagascar: Antananarivo**: 3 km 41° NE Andranomay, 11.5 km 147° SSE Anjozorobe, -18.47333, 47.96, 1300 m, montane rainforest (Fisher, Griswold et al.) (CAS); Mandraka Park, -18.9019, 47.90786, 1360 m, montane shrubland (B.L. Fisher et al.) (CAS). **Antsiranana**: 11.0 km WSW Befingotra, Réserve Anjanaharibe-Sud, -14.75, 49.45, 1565 m, montane rainforest (B.L. Fisher) (CAS); 6.5 km SSW Befingotra, Réserve Anjanaharibe-Sud, -14.75, 49.5, 875 m, rainforest (B.L. Fisher) (CAS); 9.2 km WSW Befingotra, Réserve Anjanaharibe-Sud, -14.75, 49.46667, 1200 m, montane rainforest (B.L. Fisher) (CAS); Ambondrobe, 41.1 km 175° NW Vohemar, -13.71533, 50.10167, 10 m, littoral rainforest (B.L. Fisher) (CAS); Ampasindava, Andranomatavy Forest, -13.66296, 47.97936, 543 m, disturbed dry forest (B.L. Fisher et al.) (CAS); Ampasindava, Andranomatavy Forest, -13.6648, 47.98702, 275 m, disturbed dry forest (B.L. Fisher et al.) (CAS); Ampasindava, Andranomatavy Forest, -13.66898, 47.98775, 149 m, disturbed dry forest (B.L. Fisher et al.) (CAS); Ampasindava, Forêt d’Ambilanivy, 3.9 km 181° S Ambaliha, -13.79861, 48.16167, 600 m, rainforest (Fisher, Griswold et al.) (CAS); Antsaraingy, -12.90665, 49.6606, 90 m, littoral forest (B.L. Fisher et al.) (CAS); Binara Forest, -13.26207, 49.60505, 692 m, rainforest (B.L. Fisher et al.) (CAS); Binara Forest, -13.26206, 49.60672, 559 m, degraded rainforest (B.L. Fisher et al.) (CAS); District of Vohemar, Analabe Sahaka 43 km E of Daraina, -13.07933, 49.90233, 30 m, wooded grassland-bushland (Mike, Rin’ha) (CAS); Forêt Ambanitaza, 26.1 km 347° Antalaha, -14.67933, 50.18367, 240 m, rainforest (B.L. Fisher) (CAS); Forêt Ambato, 26.6 km 33° Ambanja, -13.4645, 48.55167, 150 m, rainforest (B.L. Fisher) (CAS); Forêt d’Andavakoera, 21.4 km 75° ENE Ambilobe; 4.6 km 356° N Betsiaka, -13.11833, 49.23, 425 m, rainforest (B.L. Fisher) (CAS); Forêt d’Andavakoera, 21.4 km 75° ENE Ambilobe; 4.6 km 356° N Betsiaka, -13.11833, 49.23, 425 m, rainforest (B.L. Fisher) (CAS); Forêt d’d’Antsahabe, 11.4 km 275° W Daraina, -13.21167, 49.55667, 550 m, tropical dry forest (B.L. Fisher) (CAS); Forêt d’Antsahabe, 11.4 km 275° W Daraina, -13.21167, 49.55667, 550 m, tropical dry forest (B.L. Fisher) (CAS); Forêt d’Ampombofofo, -12.09949, 49.33874, 25 m, littoral forest (B.L. Fisher et al.) (CAS); Forêt d’Ampondrabe, 26.3 km 10° NNE Daraina, -12.97, 49.7, 175 m, tropical dry forest (B.L. Fisher) (CAS); Forêt d’Analabe, 30.0 km 72° ENE Daraina, -13.08333, 49.90833, 30 m, littoral rainforest (B.L. Fisher) (CAS); Forêt de Bekaraoka, 6.8 km 60° ENE Daraina, -13.16667, 49.71, 150 m, tropical dry forest (B.L. Fisher) (CAS); Forêt de Binara, 7.5 km 230° SW Daraina, -13.255, 49.61667, 375 m, tropical dry forest (B.L. Fisher) (CAS); Forêt de Binara, 9.1 km 233° SW Daraina, -13.26333, 49.60333, 650–800 m, rainforest (B.L. Fisher) (CAS); Forêt d’Orangea, 3.6 km 128° SE Remena, -12.25889, 49.37467, 90 m, littoral rainforest (Fisher, Griswold et al.) (CAS); Galoko chain, Mont Galoko, -13.58745, 48.71419, 380 m, rainforest (B.L. Fisher et al.) (CAS); Galoko chain, Mont Galoko, -13.5888, 48.72864, 980 m, montane forest (B.L. Fisher et al.) (CAS); Galoko chain, Mont Kalabenono, -13.64179, 48.67282, 643 m, rainforest (B.L. Fisher et al.) (CAS); Galoko chain, Mont Kalabenono, -13.63999, 48.67374, 498 m, rainforest (B.L. Fisher et al.) (CAS); Galoko chain, Mont Kalabenono, -13.64609, 48.67732, 937 m, rainforest (B.L. Fisher et al.) (CAS); Makirovana forest, -14.104, 50.03574, 225 m, rainforest (B.L. Fisher et al.) (CAS); Masoala, Cap Est, Forêt d’Andranoanala, -15.26158, 50.4758, 15 m, littoral forest on sandy soil (B.L. Fisher et al.) (CAS); Montagne des Français, 7.2 km 142° SE Antsiranana (=Diego Suarez), -12.32278, 49.33817, 180 m, tropical dry forest (Alpert et al.) (CAS); Nosy Be, RNI Lokobe, 6.3 km 112° ESE Hellville, -13.41933, 48.33117, 30 m, rainforest (Fisher, Griswold et al.) (CAS); PN Marojejy, Antranohofa, 26.6 km 31° NNE Andapa, 10.7 km 318° NW Manantenina, -14.44333, 49.74333, 1325 m, montane rainforest (B.L. Fisher) (CAS); PN Marojejy, Manantenina River, 27.6 km 35° NE Andapa, 9.6 km 327° NNW Manantenina, -14.435, 49.76, 775 m, rainforest (B.L. Fisher) (CAS); PN Marojejy, Manantenina River, 28.0 km 38° NE Andapa, 8.2 km 333° NNW Manantenina, -14.43667, 49.775, 450 m, rainforest (B.L. Fisher et al.) (CAS); PN Montagne d’Ambre, 12.2 km 211° SSW Joffreville, -12.59639, 49.1595, 1300 m, montane rainforest (Fisher, Griswold et al.) (CAS); RS Manongarivo, 10.8 km 229° SW Antanambao, -13.96167, 48.43333, 400 m, rainforest (B.L. Fisher) (CAS); RS Manongarivo, 12.8 km 228° SW Antanambao, -13.97667, 48.42333, 780 m, rainforest (B.L. Fisher) (CAS); RS Manongarivo, 14.5 km 220° SW Antanambao, -13.99833, 48.42833, 1175 m, montane rainforest (B.L. Fisher) (CAS); Réserve Analamerana, 16.7 km 123° Anivorano-Nord, -12.80467, 49.37383, 225 m, tropical dry forest (B.L. Fisher) (CAS); Réserve Analamerana, 28.4 km 99° Anivorano-Nord, -12.74667, 49.49483, 60 m, tropical dry forest (B.L. Fisher) (CAS); Réserve Ankarana, 7 km SE Matsaborimanga, -12.9, 49.11667, 150 m, rainforest (P.S. Ward) (CAS); RS Ambre, 3.5 km 235° SW Sakaramy, -12.46889, 49.24217, 325 m, tropical dry forest (Fisher, Griswold et al.) (CAS); RS Ankarana, 13.6 km 192° SSW Anivorano Nord, -12.86361, 49.22583, 210 m, tropical dry forest (Fisher, Griswold et al.) (CAS); RS Ankarana, 22.9 km 224° SW Anivorano Nord, -12.90889, 49.10983, 80 m, tropical dry forest (Fisher, Griswold et al.) (CAS); Sahamalaza Peninsula, Forêt d’Anabohazo, 21.6 km 247° WSW Maromandia, -14.30889, 47.91433, 120 m, tropical dry forest (Fisher, Griswold et al.) (CAS); Sakaramy, -12.44114, 49.23197, 260 m, tropical dry forest (B.L. Fisher et al.) (CAS); 1 km W Sakalava Beach, -12.26639, 49.395, 30 m, cattle trail in sandy brushy area near beach (Irwin, Schlinger, Harin’H) (CAS); 3 km W Sakalava Beach [white dunes site], -12.26972, 49.39167, 40 m, white dunes in littoral forest, (Harin’Hala, Irwin, Schlin) (CAS); 7 km N Joffreville [camp 2 of Fisher], -12.33333, 49.25, 360 m, in dry forest (R. Harin’Hala) (CAS); PN Montagne d’Ambre [1^st^ campsite], 960 m, rainforest (Irwin, Schlinger, Harin’H) (CAS); PN Montagne d’Ambre [1^st^ campsite], 960 m, rainforest (R. Harin’Hala) (CAS); PN Montagne d’Ambre [lemur trail], 975 m, rainforest (R. Harin’Hala) (CAS); PN Montagne d’Ambre [Petit Lac road], -12.533333, 49.166667, 1125 m, rainforest (R. Harin’Hala) (CAS); PN Montagne d’Ambre [Petit Lac road], -12.533333, 49.166667, 1125 m, rainforest (R. Harin’Hala) (CAS); 9.0 km NE Ivohibe, -22.42667, 46.93833, 900 m, rainforest (B.L. Fisher, Sylvain) (CAS). **Fianarantsoa**: Amoron’i Mania Region, District of Ambositra, Italaviana *Uapaca* forest, 35 km SE of Antsirabe, -20.17333, 47.086, 1359 m, *Uapaca* forest (Rin’Ha, Mike) (CAS); Atsimo Atsinanana Region, District of Farafangana, Mahabo Mananivo, 50 km S of Farafangana Ampitavananima, Forest low altitude, Littoral forest on sand 2 km E of Mobot office, -23.12983, 47.717, 33 m, Littoral Low Alt Rain Forest (Mike, Frank, Rin’ha) (CAS); Vatovavy Fitovinany Region, District of Ifanadiana, 12 km W of Ranomafana, -21.25083, 47.40717, 1127 m, forest edge, open area (Rin’Ha, Mike) (CAS); Forêt d’Atsirakambiaty, 7.6 km 285 °WNW Itremo, -20.59333, 46.56333, 1550 m, montane rainforest (Fisher, Griswold et al.) (CAS); Forêt de Vevembe, 66.6 km 293° Farafangana, -22.791, 47.18183, 600 m, rainforest, transition to montane forest (B.L. Fisher et al.) (CAS); Forêt de Vevembe, 66.6 km 293° Farafangana, -22.791, 47.18183, 600 m, rainforest, transition to montane forest (B.L. Fisher et al.) (CAS); Horombe Region, Ihosy Distric, PN Isalo, 1 km E of ANGAP Interpretation Center, -22.62667, 45.35817, 823 m, open area (Rin’Ha, Mike) (CAS); RS Manombo, 32 km SE of Farafangana, -23.02183, 47.72, 36 m, Lowland rainforest (Rin’Ha, Mike) (CAS); Miandritsara Forest, 40 km S of Ambositra, -20.79267, 47.17567, 822 m, Low altitude rainforest (Rin’Ha, Mike) (CAS); PN Isalo, 9.1 km 354° N Ranohira, -22.48167, 45.46167, 725 m, gallery forest (Fisher, Griswold et al.) (CAS); Réserve Forestière d’Agnalazaha, Mahabo, 42.9 km 215° Farafangana, -23.19383, 47.723, 20 m, littoral rainforest (B.L. Fisher et al.) (CAS); Réserve Forestière d’Agnalazaha, Mahabo, 42.9 km 215° Farafangana, -23.19383, 47.723, 20 m, littoral rainforest (B.L. Fisher et al.) (CAS); Réserve Speciale Manombo 24.5 km 228° Farafangana, -23.01583, 47.719, 30 m, rainforest (B.L. Fisher et al.) (CAS); Tsaranoro, 32.8 km 229° Ambalavao, -22.08483, 46.77633, 950 m, rainforest (B.L. Fisher et al.) (CAS). **Mahajanga**: Boeny Region, District of Soalala, Beaboaly Bamboo forest Station10 km SW of Soalala, 04 km of Baly village, -16.04533, 48.804, 9 m, Bamboo Forêt (Mike, Rin’ha) (CAS); Boeny Region, District of Soalala Analamanitra forest, 14 km SW of Mitsinjo, -16.7, 45.7, 19 m, dense dry forest (Mike, Rin’ha) (CAS); Boeny Region, District of Soalala Analamanitra forest, 14 km SW of Mitsinjo, -16.7, 45.7, 19 m, dense dry forest (Mike, Rin’ha) (CAS); Forêt Ambohimanga, 26.1 km 314° Mampikony, -15.96267, 47.43817, 250 m, tropical dry forest (B.L. Fisher) (CAS); Melaky Region, District of Maintirano, Asondrodava dry forest against dune 15 km N of Maintirano, -17.96533, 44.0355, 16 m, dry forest (Irwin, Rinha) (CAS); PN Ankarafantsika, Ampijoroa SF, 40 km 306° NW Andranofasika, -16.32083, 46.81067, 130 m, tropical dry forest (Fisher, Griswold et al.) (CAS); Region Sofia, Bemanevika, -14.337, 48.58874, 1606 m, montane rainforest (B.L. Fisher et al.) (CAS); RS Bemarivo, 23.8 km 223° SW Besalampy, -16.925, 44.36833, 30 m, tropical dry forest (Fisher, Griswold et al.) (CAS); Sofia Region, District of Port-Berger, Ambovomamy 20 km N of Port-Berger, -15.45117, 47.61333, 86 m, secondary forest on white sandy area (Mike, Frank, Rin’ha) (CAS). **Toamasina**: 1 km SSW Andasibe (=Perinet), -18.93333, 48.41667, 920 m, rainforest (P.S. Ward) (CAS); 5.3 km SSE Ambanizana, Andranobe, -15.66667, 49.96667, 600 m, rainforest (B.L. Fisher) (CAS); 5.3 km SSE Ambanizana, Andranobe, -15.67133, 49.97395, 425 m, rainforest (B.L. Fisher) (CAS); 6.2 km SSE Ambanizana, Be Dinta, -15.66667, 49.99806, 600 m, rainforest (V. Razafimahatratra.) (CAS); 6.3 km S Ambanizana, Andranobe, -15.6813, 49.958, 25 m, rainforest (B.L. Fisher) (CAS); 6.3 km S Ambanizana, Andranobe, -15.6813, 49.958, 25 m, rainforest (B.L. Fisher) (CAS); 6.9 km NE Ambanizana, Ambohitsitondroina, -15.58506, 50.00952, 825 m, rainforest (B.L. Fisher) (CAS); Ambatovy, 12.4 km NE Moramanga, -18.84963, 48.2947, 1010 m, montane rainforest (B.L. Fisher et al.) (CAS); Analalava, 7.0 km 255° Mahavelona, -17.7095, 49.454, 50 m, littoral rainforest (B.L. Fisher et al.) (CAS); Analanjirofo Region, District of Toamasina, Mobot Site, Analalava humid dense forest low altitude on the sand 7 km SW of Foulpointe, -17.69333, 49.46028, 75 m, dense humide low alt on the sandy soil (Mike, Rin’ha) (CAS); PN Andasibe, botanic garden near entrance, West of ANGAP office, -18.925172, 48.418651, 1025 m, tropical forest (M.E. Irwin R. Harin’Hala) (CAS); Ankerana, -18.40062, 48.81311, 865 m, rainforest (B.L. Fisher et al.) (CAS); Ankerana, -18.4061, 48.82029, 725 m, rainforest (B.L. Fisher et al.) (CAS); Corridor Forestier Analamay-Mantadia, Ambohibolakely, -18.77898, 48.36375, 918 m, rainforest (B.L. Fisher et al.) (CAS); Corridor Forestier Analamay-Mantadia, Ambohibolakely, -18.76131, 48.36437, 983 m, rainforest (B.L. Fisher et al.) (CAS); Corridor Forestier Analamay-Mantadia, Tsaravoniana, -18.76465, 48.41938, 1039 m, rainforest (B.L. Fisher et al.) (CAS); FC Andriantantely, -18.695, 48.81333, 530 m, rainforest (H.J. Ratsirarson) (CAS); FC Didy, -18.19833, 48.57833, 960 m, rainforest (H.J. Ratsirarson) (CAS); Forêt Ambatovy, 14.3 km 57° Moramanga, -18.85083, 48.32, 1075 m, montane rainforest (B.L. Fisher) (CAS); Ile Sainte Marie, Forêt Kalalao, 9.9 km 34° Ambodifotatra, -16.9225, 49.88733, 100 m, rainforest (B.L. Fisher et al.) (CAS); Mahavelona (Foulpointe), -17.66667, 49.5, in sandy forest (A. Pauly) (CAS); Montagne d’Akirindro 7.6 km 341° NNW Ambinanitelo, -15.28833, 49.54833, 600 m, rainforest (Fisher, Griswold et al.) (CAS); Montagne d’Anjanaharibe, 19.5 km 27° NNE Ambinanitelo, -15.17833, 49.635, 1100 m, montane rainforest (Fisher, Griswold et al.) (CAS); PN Mantadia, -18.79167, 48.42667, 895 m, rainforest (H.J. Ratsirarson) (CAS); RNI Betampona, -17.91132, 49.21167, 400 m, rainforest (E. Lokensgard) (CAS); Res. Ambodiriana, 4.8 km 306° Manompana, along Manompana river, -16.67233, 49.70117, 125 m, rainforest (B.L. Fisher et al.) (CAS); RNI Betampona, Camp Rendrirendry 34.1 km 332° Toamasina, -17.924, 49.19967, 390 m, rainforest (B.L. Fisher et al.) (CAS); RS Ambatovaky, Sandrangato river, -16.81209, 49.29216, 460 m, rainforest (B.L. Fisher et al.) (CAS); Torotorofotsy, -18.87082, 48.34737, 1070 m, montane rainforest, marsh edge (Malagasy ant team) (CAS); Torotorofotsy, -18.77048, 48.43043, 1005 m, montane rainforest (B.L. Fisher et al.) (CAS). **Toliara**: Androy Region, District of Tsihombe, 74 km S of Tsihombe, RS Cap Ste Marie, -25.58767, 45.163, 36 m, spiny bush (Rin’Ha, Mike) (CAS); Androy Region, District of Tsihombe, 74 km S of Tsihombe, RS Cap Ste Marie, -25.58767, 45.163, 152 m, Bush (Mike, Rin’ha) (CAS); Anosy Region, Anosyenne Mts, 29.33 km NW Manantenina, -24.13993, 47.07418, 540 m, montane rainforest (B.L. Fisher, F.A. Esteves et al.) (CAS); Anosy Region, District of Amboasary, PN Andohahela, Parcelle III, Ihazofotsy, 32 km NE Amboasary, -24.83083, 46.53617, 58 m, dry forest, spiny forest (Michael Irwin, Frank Parker, Rin’ha) (CAS); Anosy Region, District of Amboasary, 58 km SW of Fort Dauphin, 08 km NW of Amboasary, Berenty Special Reserve, -25.021, 46.3055, 36 m, spiny forest (Mike, Rin’ha) (CAS); Anosy Region, PN Andohahela, Col de Tanatana, -24.7585, 46.85367, 275 m, rainforest (B.L. Fisher, F.A. Esteves et al.) (CAS); 2.7 km WNW 302° Ste. Luce, -24.77167, 47.17167, 20 m, littoral rainforest (B.L. Fisher) (CAS); Atsimo Andrefana Region, District of Betioky, 30 km E Betioky, RS Beza Mahafaly (Around Research Station), -23.6865, 44.591, 165 m, Galery dry deciduous forest (Rin’Ha, Mike) (CAS); Atsimo-Andrefana Region, -23.55275, 43.74471, 45 m, coastal scrub on limestone (B.L. Fisher, F.A. Esteves et al.) (CAS); FC Analavelona, 29.2 km 343° NNW Mahaboboka, -22.675, 44.19, 1100 m, montane rainforest (Fisher, Griswold et al.) (CAS); FC Analavelona, 33.2 km 344° NNW Mahaboboka, -22.64333, 44.17167, 1300 m, montane rainforest (Fisher, Griswold et al.) (CAS); Forêt de Mite, 20.7 km 29° WNW Tongobory, -23.52417, 44.12133, 75 m, gallery forest (Fisher-Griswold Arthropod Team) (CAS); Forêt de Petriky, 12.5 km W 272° Tolagnaro, -25.06167, 46.87, 10 m, littoral rainforest (B.L. Fisher) (CAS); Forêt Ivohibe 55.0 km N Tolagnaro, -24.569, 47.204, 200 m, rainforest (B.L. Fisher et al.) (CAS); Forêt Vohidava 89.2 km N Amboasary, -24.239, 46.28233, 850 m, tropical dry forest (B.L. Fisher et al.) (CAS); Libanona beach, Tolagnaro, -25.03883, 46.996, 20 m, coastal scrub (B.L. Fisher et al.) (CAS); Manatantely, 8.5 km NW Tolagnaro, -24.9875, 46.92617, 85 m, rainforest (B.L. Fisher et al.) (CAS); Mandena, 8.4 km NNE 30° Tolagnaro, -24.95167, 47.00167, 20 m, littoral rainforest (B.L. Fisher) (CAS); PN Andohahela, Col de Tanatana, 33.3 km NW Tolagnaro, -24.7585, 46.85367, 275 m, rainforest (B.L. Fisher et al.) (CAS); PN Andohahela, Col du Sedro, 3.8 km 113° ESE Mahamavo, 37.6 km 341° NNW Tolagnaro, -24.76389, 46.75167, 900 m, montane rainforest (Fisher-Griswold Arthropod Team) (CAS); PN Andohahela, Forêt d’Ambohibory, 1.7 km 61° ENE Tsimelahy, 36.1 km 308° NW Tolagnaro, -24.93, 46.6455, 300 m, tropical dry forest (Fisher-Griswold Arthropod Team) (CAS); PN Andohahela, Manampanihy River, 5.4 km 113° ESE Mahamavo, 36.7 km 343° NNW Tolagnaro, -24.76389, 46.76683, 650 m, rainforest (Fisher-Griswold Arthropod Team) (CAS); PN Tsimanampetsotsa, Mitoho Cave, 6.4 km 77° ENE Efoetse, 17.4 km 170° S Beheloka, -24.04722, 43.75317, 40 m, spiny forest/thicket (Fisher-Griswold Arthropod Team) (CAS); PN Tsimanampetsotsa, Mitoho Cave, 6.4 km 77° ENE Efoetse, 17.4 km 170° S Beheloka, -24.04722, 43.75317, 40 m, spiny forest/thicket (Fisher-Griswold Arthropod Team) (CAS); PN Zombitse, 17.7 km 98° E Sakaraha, -22.88833, 44.70167, 760 m, tropical dry forest (Fisher, Griswold et al.) (CAS); PN Zombitse, 19.8 km 84° E Sakaraha, -22.84333, 44.71, 770 m, tropical dry forest (Fisher, Griswold et al.) (CAS); Réserve Privé Berenty, Forêt de Bealoka, Mandraré River, 14.6 km 329° NNW Amboasary, -24.95694, 46.2715, 35 m, gallery forest (Fisher-Griswold Arthropod Team) (CAS); RS Ambohijanahary, Forêt d’Ankazotsihitafototra, 34.6 km 314° NW Ambaravaranala, -18.26, 45.41833, 1100 m, montane rainforest (Fisher, Griswold et al.) (CAS); RS Ambohijanahary, Forêt d’Ankazotsihitafototra, 35.2 km 312° NW Ambaravaranala, -18.26667, 45.40667, 1050 m, montane rainforest (Fisher, Griswold et al.) (CAS); RS Cap Sainte Marie, 12.3 km 262° W Marovato, -25.58167, 45.16833, 200 m, spiny forest/thicket (Fisher-Griswold Arthropod Team) (CAS); RS Cap Sainte Marie, 14.9 km 261° W Marovato, -25.59444, 45.14683, 160 m, spiny forest/thicket (Fisher-Griswold Arthropod Team) (CAS); 3 km E Itampolo, malaise across path of lower bench of Andrimpano Forest, -24.65783, 43.95617, 45 m, dry forest (M.E. Irwin, Rin’ha) (CAS); 5 km E Itampolo, malaise across path of plateau of Andrimpano Forest, -24.65033, 43.96317, 130 m, dry forest (M.E. Irwin, Rin’ha) (CAS); Ambohimahavelona village 33 km NE of Tulear, Andoharano dry forest, -23.44083, 43.89967, 46 m, dry forest (M.E. Irwin, Rin’ha) (CAS); Itampolo, Sud A Sud Hotel, malaise in dune vegetation, -24.6905, 43.944, 12 m, littoral bush (M.E. Irwin, Rin’ha) (CAS); Mikea Forest, deciduous dry forest, -22.90367, 43.4755, 30 m, deciduous dry forest (R. Harin’Hala) (CAS); PN Zombitse, near ANGAP office, -22.8865, 44.69217, 840 m, deciduous spiny forest (R. Harin’Hala) (CAS); PN Zombitse, near road, -22.8405, 44.73117, 825 m, spiny deciduous forest (R. Harin’Hala) (CAS); PN Andohahela, Tsimelahy - Parcel II, transition forest, -24.93683, 46.62667, 180 m, transition forest (M.E. Irwin, F.D. Parker, R. Harin’Hala) (CAS). **Mayotte**: Dapani, -12.96279, 45.15037, 135 m, rainforest (B.L. Fisher et al.) (CAS); Dapani, -12.96279, 45.15037, 135 m, rainforest (B.L. Fisher et al.) (CAS); Mont Chongui, -12.95776, 45.13403, 470 m, rainforest (B.L. Fisher et al.) (CAS); Mont Combani, -12.80486, 45.15266, 460 m, rainforest (B.L. Fisher et al.) (CAS); Mt. Choungui, -12.8, 45.1, 360 m, forest near fallen tree (R. Jocqué & G. DeSmet) (CAS); Reserve forestière Sohoa, -12.80586, 45.10054, 20 m, coastal scrub, rainforest (B.L. Fisher et al.) (CAS); Reserve forestière Sohoa, -12.81237, 45.10476, 10 m, coastal dry forest (B.L. Fisher et al.) (CAS).

##### Diagnosis.

Lateral cephalic margins approximately parallel in full-face view; two apical teeth of mandible normally spaced; antennal scape covered with suberect hairs inclined at ca. 30°; in lateral view, posterior 1/2 of mesonotum to posterodorsal corner of propodeum somewhat convex, propodeal dorsum ca. 2 × as long as the height of declivity surface; petiolar node flattened anteroposteriorly.

##### Description.

**Minor worker.** In full-face view, head sides anterior to level of eye parallel; lateral margins posterior to level of eye converging progressively to posterior margin; length of posterior portion of head behind eye level 1/3 length of head (PoOc/CL: 0.30±0.01; 0.28–0.31). Eyes protruding and large (EL/CS: 0.30±0.02; 0.27–0.33), breaking lateral cephalic margin. Frontal carinae not strongly diverging posteriorly (FR/CS: 0.28±0.01; 0.26–0.30) and posteriorly parallel; clypeus with anterolateral angle and its anterior margin with medial blunt angle or convex. Mandible with six teeth, two apical teeth normally placed. Antennal scape relatively long (SL/CS: 1.55±0.07; 1.40–1.73) with suberect hairs inclined 30°. Promesonotum weakly convex and mesopropodeum feebly convex (MPH/ML: 0.39±0.02; 0.36–0.42), posterior portion of mesonotum flat immediately anterior to metanotal groove, metanotal groove weakly visible, propodeal dorsum anteriorly convex and posteriorly flat, junction of dorsal margin and declivity with blunt angle, propodeal dorsum 2 × as long as the declivity. Petiolar node flattened anteroposteriorly or short and high, its dorsal margin rounding to anterior margin. Tibia of hind leg rounded.

First and second gastral tergites without a pair of white spots. Erect hairs on lateral margin of head anterior to level of eyes present, but absent posterior to level of eyes; posterior margin of head with two pairs of erect hairs; posterodorsal angle of propodeum with a pair of erect hairs.

**Major worker.** Differing from minor worker in the following characters: enlarged head (CS: 2.92±0.15; 2.62–3.10; CWb/CL: 0.93±0.03; 0.90–0.99), in full-face view with slightly concave posterior margin, eye not breaking lateral cephalic margin; apical 1/4 of antennal scape surpassing posterior cephalic margin; robust mandibles and mesosoma, metanotum distinctly visible; petiolar node tapering dorsally.

##### Distribution and biology.

**Figure 67.**
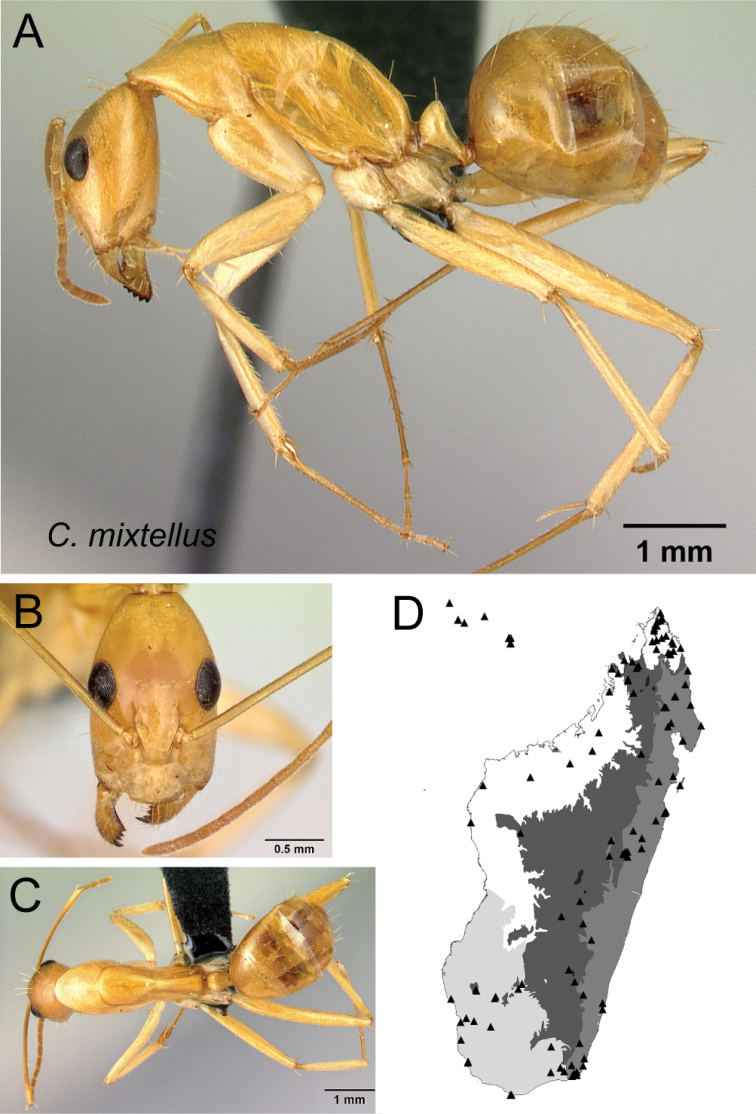
*Camponotusmixtellus***A** lateral view **B** head in full-face view **C** dorsal view of minor worker CASENT0076675**D** distribution map.

*Camponotusmixtellus* is a widespread species of the Malagasy region that occurs in the eastern lowland to montane rainforests and part of the western dry forests of Madagascar; it is known also from secondary rainforest and dry forest habitats of Grand Comore and Moheli of the Comoros islands (Fig. 67D). As this species is both arboreal and terrestrial, its members are commonly found foraging on low vegetation and on the ground, and nest in dead branches and twigs above the ground, in rotten logs and rotting tree stumps, in the ground, or under stones.

##### Discussion.

*Camponotusmixtellus* can be confused with *C.hovahovoides*, but the latter has a longer propodeal dorsum that is ca. 3 × as long as the declivity. It looks similar to *C.boivini* but the latter is characterized by an antennal scape covered with suberect hairs inclined at ca. 45°.

The cluster of *C.mixtellus* shown by the NC-clustering method is confirmed by the cumulative LDA at 100% identification success, indicating support of the species hypothesized by qualitative morphology-based taxonomy.

#### ﻿ Camponotus niavo sp. nov.

Taxon classificationAnimaliaHymenopteraFormicidae

068C5F9C-A615-5A9A-B483-4EC385092ACE

http://zoobank.org/F320A1BF-3E9B-4D78-AF9A-EC548B29F6F6

Figs 10A , 68

##### Holotype worker.

**Madagascar**: Province **Antsiranana**: PN Montagne d’Ambre, Antomboka, -12.50035, 49.175, 885 m, montane rainforest, ex rotten log, 16 Nov 2007 (B.L. Fisher et al.) collection code: BLF18356 specimen code: CASENT0134004 (CAS).

##### Paratype.

1 major worker of same data as holotype but with specimen code: CASENT0134003 (CAS).

##### Additional material examined.

**Madagascar: Antsiranana**: Ampasindava, Forêt d’Ambilanivy, 3.9 km 181° S Ambaliha, -13.79861, 48.16167, 600 m, rainforest (Fisher, Griswold et al.) (CAS); Galoko chain, Mont Galoko, -13.58487, 48.71818, 520 m, rainforest (B.L. Fisher et al.) (CAS); Galoko chain, Mont Kalabenono, -13.64609, 48.67732, 937 m, rainforest (B.L. Fisher et al.) (CAS); PN Montagne d’Ambre, Antomboka, -12.50035, 49.175, 885 m, montane rainforest (B.L. Fisher et al.) (CAS); RS Manongarivo, 10.8 km 229° SW Antanambao, -13.96167, 48.43333, 400 m, rainforest (B.L. Fisher) (CAS); RS Manongarivo, 12.8 km 228° SW Antanambao, -13.97667, 48.42333, 780 m, rainforest (B.L. Fisher) (CAS).

##### Diagnosis.

With head in full-face view, lateral cephalic margin converging posteriorly towards eye level, covered with erect hairs anterior and posterior to eye level; anteromedian margin of clypeus broadly convex; two apical teeth of mandible normally spaced.

##### Description.

**Minor worker.** In full-face view, head widest at midlength, lateral margins posterior to level of eye rounding evenly to posterior margin; eye large, slightly convex (EL/CS: 0.27±0.01; 0.26–0.30), not breaking lateral cephalic margin, level of its posterior margin located approximately at posterior 1/4 of the head (PoOc/CL: 0.28±0.02; 0.25–0.32); frontal carinae wide (FR/CS: 0.25±0.01; 0.24–0.28), distance between them larger than their distance to eye; clypeus with anterolateral angle and anteromedian margin with blunt or convex angle; mandible with two apical teeth distantly spaced; antennal scape relatively long (SL/CS: 1.70±0.11; 1.59–1.88). Promesonotum weakly convex, posterior portion of mesonotum flat immediately anterior to weakly visible metanotal groove; propodeal dorsum almost straight; dorsal margin of propodeum and declivity junction bluntly rounded; propodeal dorsum 2 × as long as declivity. Petiolar node short and high, its dorsal margin inclined posteriorly and joining anterior face in a blunt angle; anterior face 1/3 of height of the posterior; femur of hind leg rounded axially, without torsion near base.

First and second gastral tergites without a pair of white spots; erect hairs present on lateral margin of head, near its posterior margin bearing more than six erect hairs; antennal scape with suberect hairs inclined at ca. 30°, appressed hairs lacking; promesonotum covered with erect hairs; posterodorsal angle of propodeum with more than two erect hairs.

**Major worker.** Differing from minor worker in the following characters: enlarged head (CS: 4.02±0.35; 3.69–4.61; CWb/CL: 0.92±0.08; 0.79–1.01); more robust mandible; apical 1/4 of antennal scape surpassing posterior cephalic margin; pronotum convex, mesonotum sloping towards metanotum, propodeal dorsum feebly convex and joining the declivity at a broad angle; petiolar node higher than long.

##### Distribution and biology.

**Figure 68.**
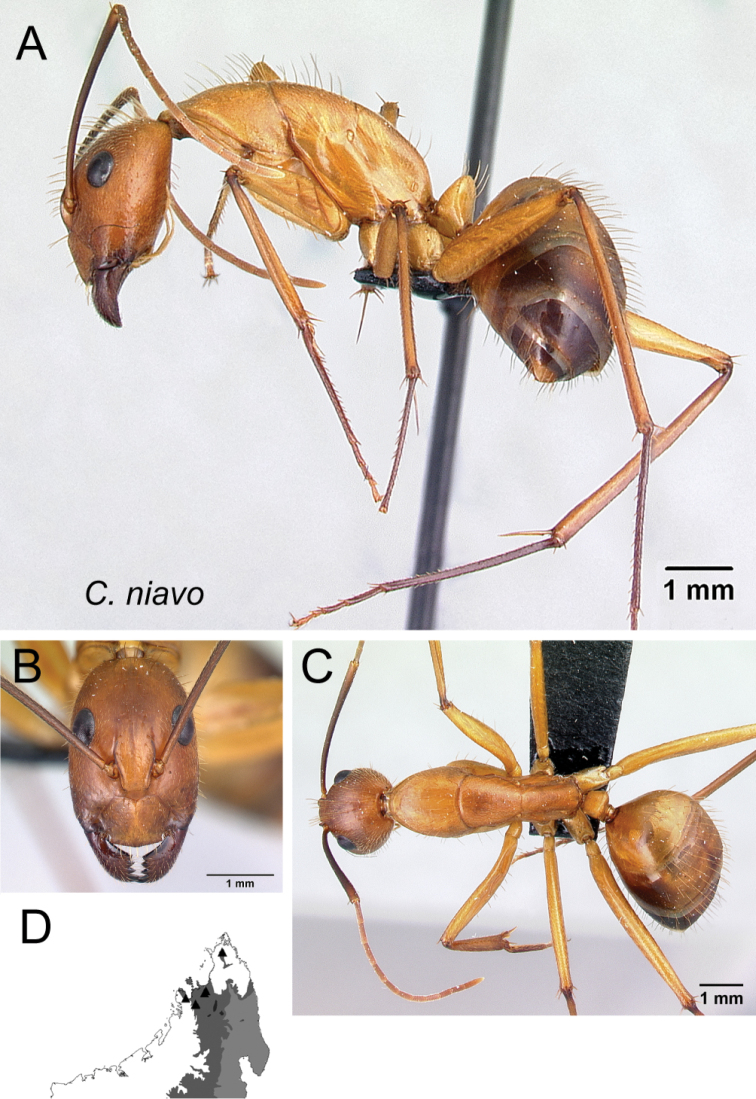
*Camponotusniavo***A** lateral view **B** head in full-face view **C** dorsal view of holotype minor worker CASENT0134004**D** distribution map.

*Camponotusniavo* is geographically restricted to the transitional humid forest of Ampasindava Peninsula, the rainforests of the Galoko chain and RS Manongarivo, and the montane rainforest of the PN Montagne d’Ambre (Fig. 68D). Colony nests are established in rotten logs and rotting tree stumps, while foraging is carried out on the forest floor and through leaf litter.

##### Discussion.

See discussion under *C.cervicalis*.

The qualitative, morphology-based analysis of this species agrees with the multivariate morphometric analysis to provide 100% support for the taxonomic determination of *C.niavo*.

##### Etymology.

The species name *niavo* is a non-Latin singular noun used in apposition and is derived from the Malagasy word that means “from above”. It refers to the high mountains where the species has been collected.

#### ﻿ Camponotus quadrimaculatus

Taxon classificationAnimaliaHymenopteraFormicidae

Forel

40363131-ED8C-5088-8F0B-464124EDC73E

**Figure 69.**
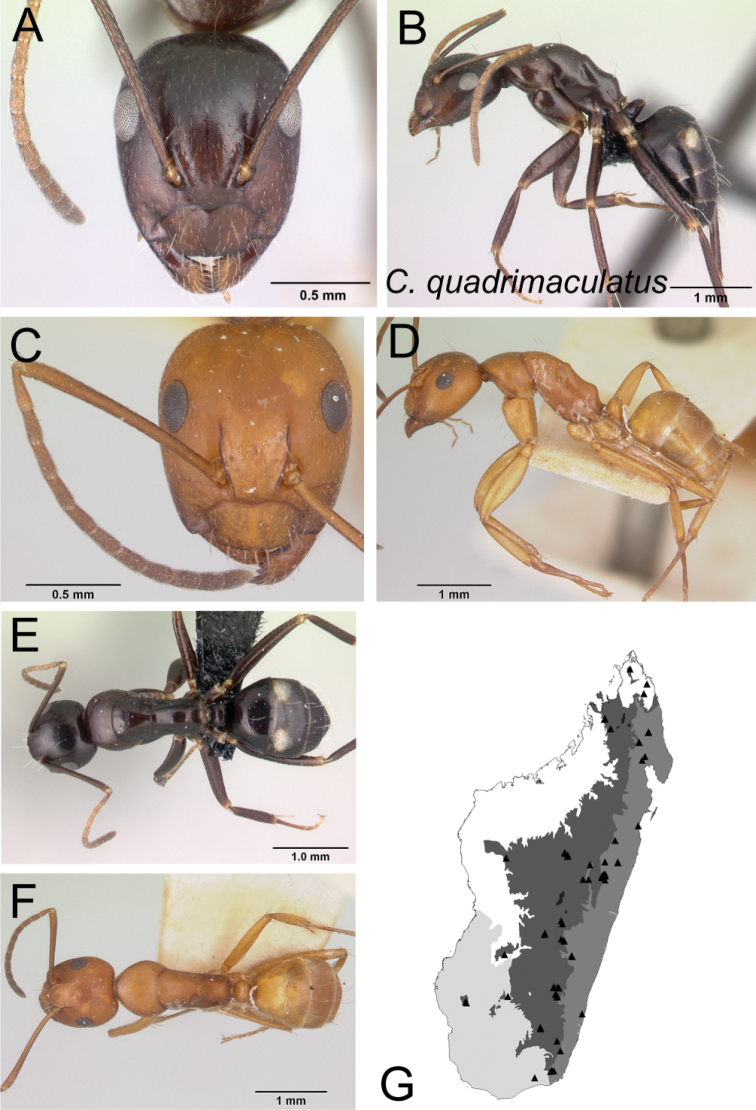
*Camponotusquadrimaculatus***A, C** head in full-face view **B, D** lateral view **E, F** dorsal view of minor workers CASENT0096044 and CASENT0101517**G** distribution map.

Figs 15C , 24B , 35B , 38B , 39B , 69

Camponotus quadrimaculatus Forel, 1886a: cii. Lectotype minor worker, by present designation, Madagascar, Fianarantsoa (Besson) AntWeb CASENT0102426 (MHNG) [examined]. Paralectotypes: 1 minor worker, 2 major workers, 1 alate queen and 2 males of same data as lectotype but respectively specimen coded as: CASENT0102443, CASENT0102427, CASENT0102442CASENT0102424 (queen), CASENT0102425 (MHNG), CASENT0104625 (ZMHB) (male) (Besson). [Camponotusquadrimaculatus Forel, 1879: 115. Nomen nudum.]. Raised to species by Forel 1891: 58 (redescription), 1907: 91; Dalla Torre 1893: 249; Emery 1896: 374, 1925: 123; Wheeler 1922: 1046; Bolton 1995: 119. [Combination in Camponotus (Myrmosaga) Forel, 1912: 92; in Camponotus (Mayria) Emery, 1925: 123].
Camponotus quadrimaculatus sellaris Emery, 1895: 344. Syntype workers, Madagascar, Antsiranana [[Diego Suarez; 7]; Antsahampano; Antsiranana Rural, -12.323135, 49.294285, 67 m] (Alluaud 1893); 1 syntype minor worker designated as lectotype, by present designation, AntWeb CASENT0102109 (MSNG) [examined]. Paralectotypes: 1 minor worker, 3 major workers of same data as lectotype but respectively with the following specimen codes: CASENT0102436 (MHNG); CASENT0102110, CASENT0102111 (MSNG); CASENT0102435 (MHNG) [examined]. [Combination in Camponotus (Mayria) Emery, 1925: 123]. Syn. nov.
Camponotus kelleri Forel, 1886b: 186. Syntype workers, Madagascar, Toamasina (C. Keller); 1 syntype major worker designated as lectotype, by present designation, AntWeb CASENT0101519 (MHNG) [examined]. Paralectotypes: 2 major workers of same data as lectotype but with the following specimen codes: CASENT0101524, CASENT0101525 (MHNG) [examined]. Syn. nov.
Camponotus kelleri var. invalidus Forel, 1897: 200. Syntype workers, Madagascar, Nosibé [Antsiranana, Nosy be, -13.291667, 48.258335, 146 m] (Voeltzkow); 1 syntype minor worker designated as lectotype, by present designation, AntWeb CASENT0101517 (MHNG) [examined]. Paralectotype: 1 major worker of same data as lectotype but specimen coded as: CASENT0101520 (MHNG) [examined]. [Combination in Camponotus (Mayria): Emery, 1925: 123]. Syn. nov.

##### Additional material examined.

**Comoros**: **Anjouan**: Lac Dzialandée, -12.22474, 44.43121, 900 m, disturbed montane rainforest (B.L. Fisher et al.) (CAS). Mount Ntringui, -12.22043, 44.42924, 1225 m, montane forest (B.L. Fisher et al.) (CAS). -12.18771, 44.35929, 65 m, coastal roadside (B.L. Fisher et al.) (CAS). -12.30537, 44.45031, 500 m, along roadside, mango, banana (B.L. Fisher et al.) (CAS). -12.30355, 44.46926, 885 m, along roadside plantation cows (B.L. Fisher et al.) (CAS). -12.29311, 44.5109, 440 m, along roadside, mango (B.L. Fisher et al.) (CAS). **Grande Comore**: Grillé, -11.47578, 43.34669, 995 m, montane rainforest (B.L. Fisher et al.) (CAS); Grillé, -11.47578, 43.34669, 805 m, bananas plantation (B.L. Fisher et al.) (CAS). Itoundzou, -11.63136, 43.30434, 635 m, secondary rainforest along roadside (B.L. Fisher et al.) (CAS). Karthala, -11.82699, 43.4295, 1000 m, montane rainforest (B.L. Fisher et al.) (CAS). Mouadja, -11.47435, 43.3004, 350 m, coastal scrub (B.L. Fisher et al.) (CAS). **Mohéli**: Ouallah, -12.32717, 43.65952, 10 m, coastal scrub (B.L. Fisher et al.) (CAS); Ouallah, -12.30668, 43.66407, 275 m, rainforest (B.L. Fisher et al.) (CAS); Ouallah, -12.29696, 43.67392, 680 m, rainforest (B.L. Fisher et al.) (CAS). **Madagascar: Antananarivo**: Analamanga Region, District of Ankazobe, Ambohitantely, 46 km NE of Ankazobe, -18.198, 47.2815, 701 m, Forêt sclerophylle (Mike, Rinha) (CAS); Ankokoy Forest, 3 km E Ibity, malaise in *Uapaca* forest, -20.0675, 46.9995, 1700 m, *Uapaca* forest (M.E. Irwin, Rin’ha) (CAS). **Antsiranana**: [Nosibé], Nosy be, Nosibe, -13.29166667, 48.25833333, 146 m (MHNG); [Diego Suarez; 7]; Antsahampano; Antsiranana Rural, -12.323135, 49.294283, 67 m (Ch. Alluaud) (MSNG); Ampasindava, Forêt d’Ambilanivy, 3.9 km 181° S Ambaliha, -13.79861, 48.16167, 600 m, rainforest (Fisher, Griswold et al.) (CAS); Ambondrobe, 41.1 km 175° NW Vohemar, -13.71533, 50.10167, 10 m, littoral rainforest (B.L. Fisher) (CAS); Forêt Ambanitaza, 26.1 km 347° Antalaha, -14.67933, 50.18367, 240 m, rainforest (B.L. Fisher) (CAS); Forêt d’Antsahabe, 11.4 km 275° W Daraina, -13.21167, 49.55667, 550 m, tropical dry forest (B.L. Fisher) (CAS); Forêt de Binara, 7.5 km 230° SW Daraina, -13.255, 49.61667, 375 m, tropical dry forest (B.L. Fisher) (CAS); Forêt de Binara, 9.1 km 233° SW Daraina, -13.26333, 49.60333, 650–800 m, rainforest (B.L. Fisher) (CAS); Galoko chain, Mont Galoko, -13.58487, 48.71818, 520 m, rainforest (B.L. Fisher et al.) (CAS); PN Montagne d’Ambre, Pic Bades, -12.5186, 49.18625, 900 m, montane rainforest (B.L. Fisher et al.) (CAS); Antongombato, 2.2 km SW Antsiranana, -12.37277, 49.22888, 74 m, urban/garden (B.L. Fisher et al.) (CAS); RS Manongarivo, 14.5 km 220° SW Antanambao, -13.99833, 48.42833, 1175 m, montane rainforest (B.L. Fisher) (CAS); Réserve Analamerana, 16.7 km 123° Anivorano-Nord, -12.80467, 49.37383, 225 m, tropical dry forest (B.L. Fisher) (CAS); RS Ambre, 3.5 km 235° SW Sakaramy, -12.46889, 49.24217, 325 m, tropical dry forest (Fisher, Griswold et al.) (CAS); RS Ankarana, 13.6 km 192° SSW Anivorano Nord, -12.86361, 49.22583, 210 m, tropical dry forest (Fisher, Griswold et al.) (CAS); Sakaramy 07 km N of Joffre Ville, -12.33333, 49.25, 360 m, Low rain forest in open area (Mike, Frank, Rin’ha) (CAS). **Fianarantsoa**: [Fianarantsoa], 2^eme^ Arrondissement; Fianarantsoa Urban, -21.43333, 47.08333, 1160 m (Dr Besson) (MHNG); Amoron’i Mania Region, District of Ambositra, Italaviana *Uapaca* forest, 35 km SE of Antsirabe, -20.17333, 47.086, 1359 m, *Uapaca* forest (Rin’Ha, Mike) (CAS); Ampangabe V Non Protected Area, 21.37 km W Itremo, -20.61361, 46.60799, 1449 m, Shrubland (A. Ravelomanana) (CAS); Ampangabe VI Non Protected Area, 21.16 km W Itremo, -20.61444, 46.6104, 1379 m, Shrubland (A. Ravelomanana) (CAS); dry wash, 1 km E of PN Isalo Interpretive Center, -22.62667, 45.35817, 885 m, dry wash (M.E. Irwin, F.D. Parker, R. Harin’Hala) (CAS); Forêt d’Analalava, 29.6 km 280° W Ranohira, -22.59167, 45.12833, 700 m, tropical dry forest (Fisher, Griswold et al.) (CAS); Horombe Region, District of Ihosy, Betapia (Border of Fianarantsoa and Tulear): 09 km SW of Ilakaka Saphir town, -22.62883, 45.36117, 1036 m, *Uapaca* forest (Rin’Ha, Mike) (CAS); RS Manombo, 32 km SE of Farafangana, -23.02183, 47.72, 36 m, Lowland rainforest (Rin’Ha, Mike) (CAS); PN Isalo, 9.1 km 354° N Ranohira, -22.48167, 45.46167, 725 m, gallery forest (Fisher, Griswold et al.) (CAS); PN Isalo, Ambovo Springs, 29.3 km 4° N Ranohira, -22.29833, 45.35167, 990 m, *Uapaca* woodland (Fisher, Griswold et al.) (CAS); PN Isalo, Sahanafa River, 29.2 km 351° N Ranohira, -22.31333, 45.29167, 500 m, gallery forest (Fisher, Griswold et al.) (CAS); stream area, 900 m E of PN Isalo Interpretive Center, -22.62667, 45.35817, 750 m, open area near stream (R. Harin’Hala) (CAS); Tsaranoro, 32.8 km 229° Ambalavao, -22.08483, 46.77633, 950 m, rainforest (B.L. Fisher et al.) (CAS); Tsaranoro, 32.8 km 230° Ambalavao, -22.08317, 46.774, 975 m, savannah woodland (B.L. Fisher et al.) (CAS); Vohiparara broken bridge, -21.22617, 47.36983, 1110 m, high altitude rainforest (R. Harin’Hala) (CAS). **Mahajanga**: Boeny Region, District of Marovoay, PN Ankarafantsika, Ampijoroa SF, 160 km North of Maevatanana on RN 04, -16.31933, 46.81333, 42 m, deciduous forest (Rin’Ha, Mike) (CAS); Region Sofia, Bemanevika, -14.33886, 48.58729, 1567 m, montane rainforest (B.L. Fisher et al.) (CAS); Region Sofia, Bemanevika, -14.337, 48.58874, 1606 m, montane rainforest (B.L. Fisher et al.) (CAS); RS Marotandrano, Marotandrano 48.3 km S Mandritsara, -16.28322, 48.81443, 865 m, transition humid forest (B.L. Fisher et al.) (CAS); Sofia Region, District of Port-Berger, Ambovomamy 20 km N of Port-Berger, -15.45117, 47.61333, 86 m, secondary forest on white sandy area (Mike, Frank, Rin’ha) (CAS); PN Ankarafantsika, Ampijoroa SF, 160 km N Maevatanana, deciduous forest, -16.31944, 46.81333, 43 m, deciduous forest (M. Irwin and Rin’ha Harin’hala) (CAS); Boeny Region, District of Marovoay, PN Ankarafantsika, Ampijoroa SF, 160 km North of Maevatanana on RN 04, -16.31933, 46.81333, 42 m, deciduous forest (Rin’Ha, Mike) (CAS); Forêt Ambohimanga, 26.1 km 314° Mampikony, -15.96267, 47.43817, 250 m, tropical dry forest (B.L. Fisher) (CAS); Mahavavy River, 6.2 km 145° SE Mitsinjo, -16.05167, 45.90833, 20 m, gallery forest (Fisher, Griswold et al.) (CAS); PN Ankarafantsika, Ampijoroa SF, 40 km 306° NW Andranofasika, -16.32083, 46.81067, 130 m, tropical dry forest (Fisher, Griswold et al.) (CAS); PN Ankarafantsika, Forêt de Tsimaloto, 18.3 km 46° NE de Tsaramandroso, -16.22806, 47.14361, 135 m, tropical dry forest (Fisher, Griswold et al.) (CAS); PN Namoroka, 16.9 km 317° NW Vilanandro, -16.40667, 45.31, 100 m, tropical dry forest (Fisher, Griswold et al.) (CAS); PN Tsingy de Bemaraha, 3.4 km 93° E Bekopaka, Tombeau Vazimba, -19.14194, 44.828, 50 m, tropical dry forest (Fisher-Griswold Arthropod Team) (CAS); Réserve d’Ankoririka, 10.6 km 13° NE de Tsaramandroso, -16.26722, 47.04861, 210 m, tropical dry forest (Fisher, Griswold et al.) (CAS); RS Bemarivo, 23.8 km 223° SW Besalampy, -16.925, 44.36833, 30 m, tropical dry forest (Fisher, Griswold et al.) (CAS); Sofia Region, District of Port-Berger, Ambovomamy 20 km N of Port-Berger, -15.45117, 47.61333, 86 m, secondary forest on white sandy area (Mike, Frank, Rin’ha) (CAS); Station Forestiere Ampijoroa, -16.31667, 46.81667, 80 m, tropical dry forest (P.S. Ward) (CAS); Mampikony, -16.09323, 47.64278, 49 m, urban/garden (B.L. Fisher et al.) (CAS). **Toamasina**: 25 km W. Morarano-Chrome, -17.75, 47.98333, 1200 m (A. Pauly) (CAS); Besarikata, env. Réserve Zahamena, -17.45, 48.85 (A. Pauly) (CAS). Forêt Ambatovy, 14.3 km 57° Moramanga, -18.85083, 48.32, 1075 m, montane rainforest (B.L. Fisher) (CAS); Nosy Mangabe, -15.5, 49.76667, <5 m, rainforest edge (P.S. Ward) (CAS). **Toliara**: Anosy Region, District of Amboasary, 58 km SW of Fort Dauphin, 8 km NW of Amboasary, Berenty Special Reserve, -25.00667, 46.30333, 85 m, Galery forest (Rin’Ha, Mike) (CAS); Atsimo Andrefana Region, District of Horombe, Vohibasia National Parc, Ambakaka Galery forest, 44 km NE of Sakaraha, -23.38183, 43.71267, 182 m, palm forest (Mike, Rinha) (CAS); Beza-Mahafaly, 27 km E Betioky, -23.65, 44.63333, 135 m, tropical dry forest (B.L. Fisher) (CAS); Makay Mts., -21.21985, 45.32396, 500 m, gallery forest on sandy soil (B.L. Fisher et al.) (CAS); Makay Mts., -21.22336, 45.32628, 480 m, gallery forest on sandy soil (B.L. Fisher et al.) (CAS); Makay Mts., -21.21761, 45.33917, 500 m, gallery forest on sandy soil (B.L. Fisher et al.) (CAS); 3 km NE Ambohitra (=Joffreville), -12.46667, 49.21667, 520 m, rainforest edge (P.S. Ward) (CAS); Makay, -21.31477, 45.12926, 600 m, deciduous dry forest (J.M. Bichain) (CAS); Makay, -21.3133, 45.14788, 520 m, gallery forest (J.M. Bichain) (CAS); 1 km E Mahamavo, PN Andohahela, -24.76667, 46.73333, 600 m, rainforest edge (P.S. Ward) (CAS). **Mayotte: Chembenyoumba**, -12.762222, 45.075278 (R. Jocqué) (CAS); Chembenyoumba, Tsanaraki plage, -12.75, 45.06667, litter of shrubs on mangrove edge (R. Jocqué & G. DeSmet) (CAS); Coconi, DAF Campus, -12.83333, 45.13333 (R. Jocqué) (CAS); Coconi, DAF Campus, -12.83333, 45.13333 (R. Jocqué & G. DeSmet) (CAS); Coconi, SDA (service du develppement agricole), -12.83333, 45.13333, wet area with giant bamboo (R. Jocqué & G. DeSmet) (CAS); Coconi, SDA (service du développement agricole), -12.83333, 45.13333, forested area with giant bamboo (R. Jocqué & G. DeSmet) (CAS); Dziani Karihani, -12.78333, 45.11667, forest (R. Jocqué & G. DeSmet) (CAS); Dziani Karihani, Ilang-Ilang plantation, -12.78333, 45.11667 (R. Jocqué & G. DeSmet) (CAS); entre Combani and Bouyouni, -12.735556, 45.138333, plantation (R. Jocqué) (CAS); Majimbini, -12.765833, 45.188056, in ruins (R. Jocqué) (CAS); Majimbini, -12.765833, 45.188056 (R. Jocqué) (CAS); Majimbini, -12.765833, 45.188056 (R. Jocqué) (CAS); Mlima Choungi, near crossroad GRMT1 & CCT11 (R. Jocqué) (CAS); Mlima Choungi, near crossroad GRMT1 & CCT11 (R. Jocqué) (CAS); Mont Benara, -12.87585, 45.15672, 425 m, rainforest (B.L. Fisher et al.) (CAS); Mont Chongui, -12.95903, 45.13411, 380 m, rainforest (B.L. Fisher et al.) (CAS); Mont Chongui summit, -12.99567, 45.13428, 550 m, rainforest (B.L. Fisher et al.) (CAS); Mont Combani, -12.80632, 45.15314, 370 m, rainforest (B.L. Fisher et al.) (CAS); Mt. Choungui, -12.8, 45.1, 360 m, forest near fallen tree (R. Jocqué & G. DeSmet) (CAS); Reserve Forestière Majimbini, -12.76796, 45.18615, 525 m, rainforest (B.L. Fisher et al.) (CAS); Reserve Forestière Majimbini, -12.76894, 45.19021, 350 m, rainforest (B.L. Fisher et al.) (CAS).

##### Diagnosis.

In full-face view, lateral margins of head anterior to eye level diverging posteriorly; anterior clypeal margin truncate; two pairs of white spots present on second and third abdominal tergites; pronotum, mesonotum, and propodeum not forming separate convexities, metanotal groove not depressed; propodeum immediately in junction with promesonotum; propodeal dorsum concave.

##### Description.

**Minor worker.** In full-face view, head sides diverging towards broadly convex posterior margin; eye convex and large (EL/CS: 0.26±0.02; 0.21–0.30), not breaking lateral cephalic margin, level of its posterior margin located approximately at posterior 1/4 of head (PoOc/CL: 0.25±0.01; 0.22–0.28); frontal carinae widely diverging posteriorly (FR/CS: 0.34±0.01; 0.31–0.37), distance between them larger than their smallest distance to eye; clypeus with anterolateral angle and straight anteromedian margin; mandible with two apical teeth distantly spaced; antennal scape relatively long (SL/CS: 1.23±0.07; 1.01–1.36). Promesonotum slightly convex; posterior portion of mesonotum flat immediately anterior to feebly visible metanotal groove; propodeal dorsum immediately posterior to metanotal groove convex, medially strongly concave, then posteriorly flat, joining declivity at a remarkably visible angle; declivity height 3/4 length of dorsum. Petiolar node short and high, with dorsal margin inclined posteriorly and rounding to anterior margin; anterior face shorter than posterior face; femur of hind leg rounded axially, not twisted basally.

First and second gastral tergites with a pair of white spots; lateral margin of head lacking erect hairs; two erect hairs present close to posterior margin of head; antennal scape covered only with appressed hairs; pronotum with two and mesonotum with one pairs of erect hairs, respectively; posterodorsal corner of propodeum with a pair of erect hairs. Body color shining reddish black, dark brown to orange-yellow; apical portion of appendages pale in color.

**Major worker.** Differing from minor worker in the following characters: enlarged subquadrate head (CS: 2.44±0.14; 2.19–2.66; CWb/CL: 1.04±0.03; 1.00–1.08), with broadly concave posterior margin; anteromedian clypeal margin with median excision; antennal scape barely surpassing posterior cephalic margin; strongly built mandible; robust mesosoma, with distinct metanotum; propodeal dorsum slightly to strongly concave towards declivity surface; petiolar node scalelike.

##### Distribution and biology.

Endemic to the Malagasy region, *C.quadrimaculatus* is known from Madagascar, the Comoros islands (Anjouan, Grand Comore and Moheli), and Mayotte (Fig. 69D). In Madagascar, this species is widely distributed across most of the terrestrial habitats of the island except spiny bush and thicket in the southern region. *Camponotusquadrimaculatus* is typically found from littoral rainforest and dry forest through rainforest and montane rainforests to savannah woodland habitats. It also can colonize human-modified habitats adjacent to its natural areas of distribution. The yellowish orange variant of the species has a range restricted to the northern part of Madagascar, where it occupies not only natural rainforest areas of the RS Manongarivo and the Galoko Montain Chains and the littoral rainforest of Nosy Faly, but also human-modified areas such as coffee plantations, urban garden habitats, and disturbed littoral rainforest habitats.

##### Discussion.

*Camponotusquadrimaculatus* is similar to *C.rotrae* but in the latter the propodeal dorsum is straight.

The minor workers of *C.quadrimaculatus* display a remarkable range of phenotypic diversity. This assertion of the diversity of morphological characters is made on the basis of specimens with similar promesonotum shape, dorsal outline of the propodeal dorsum, and color of the body. However, the geographic range of differences among the observed variants does not follow a simple pattern. One variant of the yellowish orange body color has a restricted geographic boundary in the north of Madagascar, while others, showing variation in the strength of the concavity on the propodeal dorsum, are widely distributed along the rainforests of the island.

Across the wide geographic range of *Camponotusquadrimaculatus*, trait variations in the worker castes of *C.quadrimaculatussellaris*, *Camponotuskelleri* and Camponotuskellerivar.invalidus have been observed. However, these variations do not present any pattern that separates them from the former species. Accordingly, *C.quadrimaculatussellaris*, *Camponotuskelleri* and Camponotuskellerivar.invalidus are synonymyzed under *Camponotusquadrimaculatus* in present study.

Multivariate morphometric analysis through the NC-clustering method was applied to identify the presence of variants detected by the qualitative morphology-based study. However, NC-clustering did not reveal the existence of discrete variants. The samples from each of the variants are spread across the cluster of *C.quadrimaculatus*. Also, its classification success is only 97.2% because one of these samples was identified by LDA as *C.gibber*. This might be due to the fact that one of the variants belonging to this species is very similar to *C.quadrimaculatus* based on the qualitative morphology-based study.

#### ﻿ Camponotus radamae

Taxon classificationAnimaliaHymenopteraFormicidae

Forel stat. rev.

66E9699F-6207-58DC-B676-0AF2BC761E77

**Figure 70.**
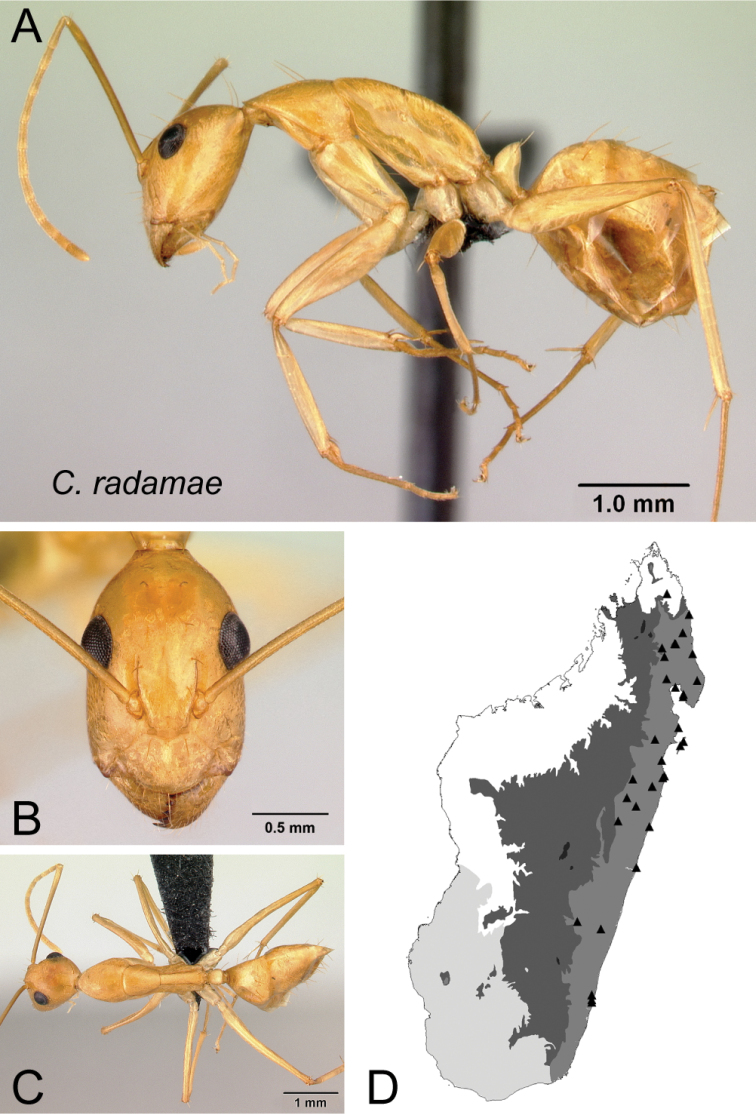
*Camponotusradamae***A** lateral view **B** head in full-face view **C** dorsal view of minor worker CASENT0066777**D** distribution map.

Figs 2B , 6B , 17A , 18B , 19A , 70

Camponotus hova radamae Forel, 1891: 31. Lectotype minor worker, by present designation, Madagascar, forêts du versant NE du grand massif (Humblot) AntWeb CASENT0104639 (ZMHB) [examined]. Paralectotypes: with the following data Forêt des bords de l’Ivondrona, près de Tamatave (Dr Conrad Keller) and forêts du versant NE du grand massif (Humblot): 2 minor workers: 1 from Forêt des bords de l’Ivondrona CASENT0101347 (MHNG); 1 from Forêts du versant NE du grand massif (Humblot) CASENT0101426 (MNHN); 4 major workers: 3 from Forêt des bords de l’Ivondrona CASENT0101346, CASENT0101867 (MHNG), CASENT0101427 (MNHN); 1 from Forêts du versant NE du grand massif (Humblot), CASENT0101432 (MNHN) [examined]. Raised to species by Dalla Torre 1893: 249. As subspecies of Camponotushova by Emery, 1920b: 6; 1925: 86; Bolton, 1995: 119 [Camponotusmaculatusr.radamae Forel, 1891]. Stat. rev.

##### Additional material examined.

**Madagascar: Antsiranana**: 6.5 km SSW Befingotra, Réserve Anjanaharibe-Sud, -14.75, 49.5, 875 m, rainforest (B.L. Fisher) (CAS); Ambondrobe, 41.1 km 175° NW Vohemar, -13.71533, 50.10167, 10 m, littoral rainforest (B.L. Fisher) (CAS); Betaolana Forest, along Bekona River, -14.52996, 49.44039, 880 m, rainforest (B.L. Fisher et al.) (CAS); Forêt Ambanitaza, 26.1 km 347° Antalaha, -14.67933, 50.18367, 240 m, rainforest (B.L. Fisher) (CAS); Forêt d’Antsahabe, 11.4 km 275° W Daraina, -13.21167, 49.55667, 550 m, tropical dry forest (B.L. Fisher) (CAS); Forêt d’Antsahabe, 11.4 km 275° W Daraina, -13.21167, 49.55667, 550 m, tropical dry forest (B.L. Fisher) (CAS); Makirovana forest, -14.16506, 49.9477, 900 m, montane rainforest (B.L. Fisher et al.) (CAS); Marojejy, tributary Manantenina R. -14.43333, 49.75, 750 m, (Quinter & Nguyen) (CAS); PN Masoala, -15.3014, 50.22776, 280 m, rainforest (B.L. Fisher et al.) (CAS); PN Masoala, -15.33058, 50.30279, 250 m, rainforest (B.L. Fisher et al.) (CAS); PN Marojejy, Manantenina River, 27.6 km 35° NE Andapa, 9.6 km 327 °NNW Manantenina, -14.435, 49.76, 775 m, rainforest (B.L. Fisher) (CAS); SAVA Region, District of Sambava, PN Marojejy, 5 km W of Manantenina village, 1^st^ Camp site (Mantella), -14.43817, 49.774, 487 m, Low altitude rainforest (Rin’Ha, Mike) (CAS). **Fianarantsoa**: 7.6 km 122° Kianjavato, FC Vatovavy, -21.4, 47.94, 175 m, rainforest (B.L. Fisher et al.) (CAS); Atsimo Atsinanana Region, District of Farafangana, Mahabo Mananivo, 50 km S of Farafangana Ampitavananima, Forest low altitude, Littoral forest on sand 2 km E of Mobot office, -23.12983, 47.717, 33 m, Littoral Low Alt Rain Forest (Mike, Frank, Rin’ha) (CAS); RS Manombo 24.5 km 228° Farafangana, -23.01583, 47.719, 30 m, rainforest (B.L. Fisher et al.) (CAS); RS Manombo, 32 km SE of Farafangana, -23.02183, 47.72, 36 m, Lowland rainforest (Rin’Ha, Mike) (CAS); Miandritsara Forest, 40 km S of Ambositra, -20.79267, 47.17567, 822 m, Low altitude rainforest (Rin’Ha, Mike) (CAS); Réserve Forestière d’Agnalazaha, Mahabo, 42.9 km 215° Farafangana, -23.19383, 47.723, 20 m, littoral rainforest (B.L. Fisher et al.) (CAS); Vohiparara broken bridge, -21.22617, 47.36983, 1110 m, high altitude rainforest (M.E. Irwin, F.D. Parker, R. Harin’Hala) (CAS). **Toamasina**: 5.3 km SSE Ambanizana, Andranobe, -15.66667, 49.96667, 600 m, rainforest (B.L. Fisher) (CAS); 5.3 km SSE Ambanizana, Andranobe, -15.67133, 49.97395, 425 m, rainforest (B.L. Fisher) (CAS); Analanjirofo Region, District of Toamasina, Mobot Site, Analalava humid dense forest low altitude 7 km SW of Foulpointe, -17.70889, 49.45806, 42 m, dense humide forest low altitude (Mike, Rin’ha) (CAS); Analanjirofo Region, District of Toamasina, Mobot Site, Analalava humid dense forest low altitude on the sand 7 km SW of Foulpointe, -17.69333, 49.46028, 75 m, dense humide low alt on the sandy soil (Mike, Rin’ha) (CAS); Ankerana, -18.40636, 48.80254, 1108 m, montane forest (B.L. Fisher et al.) (CAS); Ankerana, -18.4017, 48.80605, 1035 m, montane forest (B.L. Fisher et al.) (CAS); Corridor Forestier Analamay-Mantadia, Ambohibolakely, -18.76131, 48.36437, 983 m, rainforest (B.L. Fisher et al.) (CAS); Corridor Forestier Analamay-Mantadia, Tsaravoniana, -18.75737, 48.42302, 1018 m, rainforest (B.L. Fisher et al.) (CAS); FC Didy, -18.19833, 48.57833, 960 m, rainforest (H.J. Ratsirarson) (CAS); Forêt d’Analava Mandrisy, 5.9 km 195° Antanambe, -16.48567, 49.847, 10 m, littoral rainforest (B.L. Fisher et al.) (CAS); Ile Sainte Marie, Forêt Ambohidena, 22.8 km 44° Ambodifotatra, -16.82433, 49.96417, 20 m, littoral rainforest (B.L. Fisher et al.) (CAS); Ile Sainte Marie, Forêt Kalalao, 9.9 km 34° Ambodifotatra, -16.9225, 49.88733, 100 m, rainforest (B.L. Fisher et al.) (CAS); Mahanoro, -19.89933, 48.80883, 15 m, urban/garden (B.L. Fisher et al.) (CAS); Mahavelona (Foulpointe), -17.66667, 49.5, in forest (A. Pauly) (CAS); Mahavelona (Foulpointe), -17.66667, 49.5, Forest d’Ambodariamy (A. Pauly) (CAS); Montagne d’Akirindro 7.6 km 341° NNW Ambinanitelo, -15.28833, 49.54833, 600 m, rainforest (Fisher, Griswold et al.) (CAS); Nosy Mangabe, -15.5, 49.76667, <5 m, littoral vegetation (P.S. Ward) (CAS); PN Masoala, 40 km 154° SSE Maroantsetra, -15.72667, 49.95667, 150 m, rainforest (A. Dejean et al.) (CAS); PN Zahamena, Tetezambatana forest, near junction of Nosivola and Manakambahiny Rivers, -17.74298, 48.72936, 860 m, rainforest (B.L. Fisher et al.) (CAS); Parcelle E3 Tampolo, -17.28104, 49.43012, 10 m, littoral forest (Malagasy ant team) (CAS); Parcelle K7 Tampolo, -17.28333, 49.41667, 10 m, littoral forest (Malagasy ant team) (CAS); RNI Betampona, -17.9152, 49.20998, 330–360 m, rainforest, (M.A.Rajaonarivo) (CAS); Réserve Nationale Intégrale Betampona, Betampona 35.1 km NW Toamasina, -17.91801, 49.20074, 500 m, rainforest (B.L. Fisher et al.) (CAS); RNI Betampona, 34.08 km 332° Toamasina, -17.91977, 49.20039, 525 m, rainforest (B.L. Fisher) (CAS); RNI Betampona, 34.1 km 332° Toamasina, -17.91614, 49.20185, 550 m, rainforest (B.L. Fisher) (CAS); RS Ambatovaky, Sandrangato river, -16.77274, 49.26551, 450 m, rainforest (B.L. Fisher et al.) (CAS); RS Ambatovaky, Sandrangato river, -16.7633, 49.26692, 520 m, rainforest (B.L. Fisher et al.) (CAS); RS Ambatovaky, Sandrangato river, -16.8162, 49.29202, 425 m, rainforest (B.L. Fisher et al.) (CAS); SF Tampolo, 10 km NNE Fenoarivo Atn. -17.2825, 49.43, 10 m, littoral rainforest (B.L. Fisher) (CAS); Tampolo, -17.28333, 49.41667, 10 m, littoral forest (Malagasy ant team) (CAS) 11 km SE Ampasimanolotra (=Brickaville), -18.9, 49.13333, 5 m, littoral rainforest (P.S. Ward) (CAS).

##### Diagnosis.

Lateral cephalic margins approximately parallel in full-face view; two apical teeth of mandible closely spaced; lateral cephalic margin posterior to eye level without erect hairs; antennal scape covered with appressed hairs; mesosoma much higher and short, with propodeal dorsum 3 × as long as declivity; petiolar node more or less flattened anteroposteriorly and tapering dorsally.

##### Description.

**Minor worker.** With head in full-face view, lateral margins anterior to eye level parallel, posteriorly rounding evenly towards short-necked rear margin; eye protruding and large (EL/CL: 0.31±0.01; 0.29–0.34), breaking lateral cephalic margin, location of its posterior margin at posterior 1/3 of head (PoOc/CL: 0.29±0.01; 0.27–0.31); frontal carinae widely opened posteriorly (FR/CS: 0.32±0.01; 0.30–0.34); clypeus with blunt or poorly defined anterolateral angle, anteromedian margin broadly convex; mandible with two apical teeth closely spaced; antennal scape relatively long (SL/CS: 1.52±0.07; 1.42–1.64). Mesosoma with weakly convex promesonotum (MPH/ML: 0.34±0.01; 0.31–0.36), posterior portion flat immediately anterior to metanotal groove; metanotal groove weakly visible, propodeal dorsum almost straight, junction of propodeal dorsum and declivity bluntly angulate, propodeal declivity almost 1/2 length of the dorsum. Petiolar node flattened anteroposteriorly, with dorsal margin rounding to anterior margin; tibia of hind leg rounded axially, without basal twist.

First and second gastral tergites without a pair of white spots; erect hairs on lateral margin of head anterior to level of eyes present but absent behind eye level; antennal scape covered with appressed hairs; posterior margin of head with two erect hairs; posterodorsal angle of propodeum with a pair of erect hairs.

**Major worker.** Differing from minor worker in the following characters: enlarged head (CS: 2.68±0.31; 2.29–3.02; CWb/CL: 0.95±0.04; 0.89–0.99) with broadly concave posterior margin; two apical teeth of mandible normally spaced; antennal scape barely surpassing posterior cephalic margin; robust mesosoma, metanotum distinctly visible, propodeal dorsum almost as long as the height of much more vertical declivity, their junction forming a broad angle; more pairs of erect hairs on promesonotum, junction of propodeal dorsum, declivity, and posterodorsal margin of petiolar node.

##### Distribution and biology.

Endemic to Madagascar, *Camponotusradamae* normally occurs in eastern humid forests ranging from the littoral region to mountaintops (Fig. 70D). The species is arboreal and terrestrial, and nests in rotten logs, tree stumps, dead branches, twigs, and rot pockets above the ground, while its workers rarely forage on the ground and on lower vegetation.

##### Discussion.

*Camponotusradamae* is similar to *C.vano* in that the two apical teeth of their mandible are closely spaced, but *C.vano* has a more elongate body in which the mesosoma is very low and long, and its propodeal dorsum is at least four times as long as the declivity surface. In *C.radamae* the propodeal dorsum is 3 × as long as the declivity.

In *Camponotusradamae* the species definition is supported by the clustering shown by the dendrogram and confirmed by cumulative LDA at 100% classification success.

#### ﻿ Camponotus roeseli

Taxon classificationAnimaliaHymenopteraFormicidae

Forel

F4FB97B2-25AD-503E-B251-990BAE353C48

Figs 13B , 14B , 71

Camponotus roeseli Forel, 1910: 20. Syntype major workers, Madagascar, Montagne d’Ambre (Rolle); 1 syntype major worker designated as lectotype, by present designation, AntWeb CASENT0101602 (MHNG) [examined]. Paralectotype major worker of same data as lectotype but with specimen code: CASENT0101788 (MHNG) [examined]. [Combination in Camponotus (Myrmoturba): Forel, 1914A 267; in Camponotus (Tanaemyrmex): Emery, 1925: 89].
Camponotus maculatus st. legionarium Santschi, 1911b: 283. Holotype (by monotypy) major worker, Madagascar, Diego Suarez (Légion Étrangère 1903) AntWeb CASENT0101413 (MNHN) [examined]. Raised to species as Camponotuslegionarius by Baroni Urbani, 1972: 132. [Combination in Camponotus (Tanaemyrmex): Emery, 1925: 89]. Syn. nov.

##### Additional material examined.

**Madagascar: Antsiranana**: [Diego-Suarez]; Museum Paris, Légion Étrangére 1903]; Bevohotra; Sambava, -14, 49.5, 797 m (MNHN); [Nd. Madagascar, Amber Gebirge Nd. Madagascar H. Rolle, Berlin, S.W.11.]; Mosorolava; Antsiranana Rural, -12.616667, 49.15, 1113 m (MHNG); Antsaraingy, -12.90362, 49.65921, 66 m, littoral forest (B.L. Fisher et al.) (CAS); Antsaraingy, -12.90665, 49.6606, 90 m, littoral forest (B.L. Fisher et al.) (CAS); Diana Region, Diego II Region, Orangea dry forest, Baie de Dune 900 m E of Camps Minier, -12.23283, 49.3665, 152 m, dry forest (Mike, Rinha) (CAS); DIANA, PN Montagne d’Ambre, Roussette Camp 7 km SW Park entrance, -12.51444, 49.18139, 975 m, rainforest, (Mike, Ev, Rin’ha) (CAS); Forêt Ambato, 26.6 km 33° Ambanja, -13.4645, 48.55167, 150 m, rainforest (B.L. Fisher) (CAS); Forêt d’Andavakoera, 21.4 km 75° ENE Ambilobe; 4.6 km 356° N Betsiaka, -13.11833, 49.23, 425 m, rainforest (B.L. Fisher) (CAS); Forêt d’Ampondrabe, 26.3 km 10° NNE Daraina, -12.97, 49.7, 175 m, tropical dry forest (B.L. Fisher) (CAS); Forêt de Bekaraoka, 6.8 km 60° ENE Daraina, -13.16667, 49.71, 150 m, tropical dry forest (B.L. Fisher) (CAS); Forêt de Binara, 7.5 km 230° SW Daraina, -13.255, 49.61667, 375 m, tropical dry forest (B.L. Fisher) (CAS); Forêt d’Orangea, 3.6 km 128° SE Remena, -12.25889, 49.37467, 90 m, littoral rainforest (Fisher, Griswold et al.) (CAS); Massif du Tsaratanana (versant Sud), Andohanambatoafo; Marovato; Ambanja, -13.998127, 48.981754, 2714 m, (P. Soga) (CAS); Nosi-Bé du Majunga; Nosy be; Nosibe, -13.315028, 48.25927, 128 m (T. Carié) (CAS); Réserve Analamerana, 16.7 km 123° Anivorano-Nord, -12.80467, 49.37383, 225 m, tropical dry forest (B.L. Fisher) (CAS); Réserve Analamerana, 28.4 km 99° Anivorano-Nord, -12.74667, 49.49483, 60 m, tropical dry forest (B.L. Fisher) (CAS); Réserve Ankarana, 7 km SE Matsaborimanga, -12.9, 49.11667, 150 m, tropical dry forest (P.S. Ward) (CAS); RS Ambre, 3.5 km 235° SW Sakaramy, -12.46889, 49.24217, 325 m, tropical dry forest (Fisher, Griswold et al.) (CAS); RS Ankarana, 13.6 km 192° SSW Anivorano Nord, -12.86361, 49.22583, 210 m, tropical dry forest (Fisher, Griswold et al.) (CAS); RS Ankarana, 22.9 km 224° SW Anivorano Nord, -12.90889, 49.10983, 80 m, tropical dry forest (Fisher, Griswold et al.) (CAS); Sahamalaza Peninsula, Forêt d’Anabohazo, 21.6 km 247° WSW Maromandia, -14.30889, 47.91433, 120 m, tropical dry forest (Fisher, Griswold et al.) (CAS); SAVA Region District of Vohemar, Andranotsimaty Dry forest, 9 km NE of Daraina, -13.1695, 49.70067, 90 m, dry dense forest (Mike, Rinha) (CAS); 1 km W Sakalava Beach [cattle trail near beach], -12.26639, 49.395, 30 m, cattle trail in sandy brushy area near beach (Irwin, Schlinger, Harin’H) (CAS), 7 km N Joffreville [camp 2 of Fisher], -12.33333, 49.25, 360 m, in dry forest (R. Harin’Hala) (CAS), PN Montagne d’Ambre [1^st^ campsite], 960 m, rainforest (Irwin, Schlinger, Harin’H) (CAS); PN Montagne d’Ambre [Petit Lac road], -12.533333, 49.166667, 1125 m, rainforest (R. Harin’Hala) (CAS); SAVA Region, District of Vohemar, Antsahabelela Rain Forest, 9 km SW of Daraina, -13.2505, 49.61667, 182 m, humid Forêt (Mike, Rin’ha) (CAS). **Fianarantsoa**: dry wash, 1 km E of PN Isalo Interpretive Center, -22.62667, 45.35817, 885 m, dry wash (R. Harin’Hala) (CAS); dry wash, 1 km E of PN Isalo Interpretive Center, -22.62667, 45.35817, 885 m, dry wash (R. Harin’Hala) (CAS); Forêt d’Analalava, 29.6 km 280° W Ranohira, -22.59167, 45.12833, 700 m, tropical dry forest (Fisher, Griswold et al.) (CAS); Horombe Region, District of Ihosy, PN Isalo, 900 m E of ANGAP Interpretation Center, -22.62667, 45.35817, 701 m, open area near stream (Rin’Ha, Mike) (CAS); Horombe Region, District of Ihosy, Betapia (Border of Fianarantsoa and Tulear): 09 km SW of Ilakaka Saphir town, -22.62883, 45.36117, 1036 m, *Uapaca* forest (Rin’Ha, Mike) (CAS); PN Isalo, 9.1 km 354° N Ranohira, -22.48167, 45.46167, 725 m, gallery forest (Fisher, Griswold et al.) (CAS); PN Isalo, Sahanafa River, 29.2 km 351° N Ranohira, -22.31333, 45.29167, 500 m, gallery forest (Fisher, Griswold et al.) (CAS); stream area, 900 m E of PN Isalo Interpretive Center, -22.62667, 45.35817, 750 m, open area near stream (M.E. Irwin, F.D. Parker, R. Harin’Hala) (CAS); Tsaranoro, 32.8 km 230° Ambalavao, -22.08317, 46.774, 975 m, savannah woodland (B.L. Fisher et al.) (CAS). **Mahajanga**: Ambolomaiky, 48.1 km SE Mahajanga, -15.8541, 46.74663, 77 m, disturbed dry forest (B.L. Fisher et al.) (CAS); Bemeraha, 9 km E. Antsalova, -18.65, 44.71667 (D.C. Lees) (CAS); Boeny Region, District of Marovoay, PN Ankarafantsika, Ampijoroa SF, 160 km North of Maevatanana on RN 04, -16.31933, 46.81333, 42 m, deciduous forest (Mike, Rin’ha) (CAS); Boeny Region, District of Soalala Analamanitra forest, 14 km SW of Mitsinjo, -16.7, 45.7, 19 m, dense dry forest (Mike, Rin’ha) (CAS); Boeny Region, PN Ankarafantsika, Ambodimanga, -16.32294, 46.82602, 75 m, dry forest, Canopy Bootcamp, (P.C.Willadsen and A. Karunakaran) (CAS); Boeny Region, District of Soalala, Namoroka 53 km of Soalala, Ambatofolaka dry forest 3 km N of Vilanandro villlage, -16.47333, 45.39133, 105 m, dense dry forest in the mud (Mike, Rin’ha) (CAS); Forêt Ambohimanga, 26.1 km 314° Mampikony, -15.96267, 47.43817, 250 m, tropical dry forest (B.L. Fisher) (CAS); Forêt de Tsimembo, 8.7 km 336° NNW Soatana, -19.02139, 44.44067, 20 m, tropical dry forest (Fisher-Griswold Arthropod Team) (CAS); Mahavavy River, 10.6 km 148° SSE Mitsinjo, -16.08167, 45.935, 50 m, tropical dry forest (Fisher, Griswold et al.) (CAS); Mahavavy River, 6.2 km 145° SE Mitsinjo, -16.05167, 45.90833, 20 m, gallery forest (Fisher, Griswold et al.) (CAS); Melaky Region, District of Besalampy, Marofototra dry forest, 17 km W of Besalampy, -16.72167, 44.42367, 51 m, dry wash in the dry forest (Irwin, Rin’ha) (CAS); Melaky Region, District of Besalampy, Analangidro dry forest, 07 km NE of Besalampy, -16.6915, 44.5235, 25 m, dry forest on sand (Irwin, Rin’ha) (CAS); Réserve forestière Beanka, 54.3 km E Maintirano, -18.06009, 44.54086, 262 m, tropical dry forest on tsingy (B.L. Fisher et al.) (CAS); PN Ankarafantsika, Ampijoroa SF, 40 km 306° NW Andranofasika, -16.32083, 46.81067, 130 m, tropical dry forest (Fisher, Griswold et al.) (CAS); PN Ankarafantsika, Forêt de Tsimaloto, 18.3 km 46° NE de Tsaramandroso, -16.22806, 47.14361, 135 m, tropical dry forest (Fisher, Griswold et al.) (CAS); Station Forestiere Ampijoroa, -16.31667, 46.81667, 80 m, tropical dry forest (P.S. Ward) (CAS); PN Baie de Baly, 12.4 km 337° NNW Soalala, -16.01, 45.265, 10 m, tropical dry forest (Fisher, Griswold et al.) (CAS); PN Namoroka, 16.9 km 317° NW Vilanandro, -16.40667, 45.31, 100 m, tropical dry forest (Fisher, Griswold et al.) (CAS); PN Namoroka, 9.8 km 300° WNW Vilanandro, -16.46667, 45.35, 140 m, tropical dry forest (Fisher, Griswold et al.) (CAS); PN Tsingy de Bemaraha, 10.6 km ESE 123° Antsalova, -18.70944, 44.71817, 150 m, tropical dry forest on Tsingy (Fisher-Griswold Arthropod Team) (CAS); PN Tsingy de Bemaraha, 10.6 km ESE 123° Antsalova, -18.70944, 44.71817, 150 m, tropical dry forest on Tsingy (Fisher-Griswold Arthropod Team) (CAS); PN Tsingy de Bemaraha, 10.6 km ESE 123° Antsalova, -18.70944, 44.71817, 150 m, tropical dry forest on Tsingy (Fisher-Griswold Arthropod Team) (CAS); PN Tsingy de Bemaraha, 2.5 km 62° ENE Bekopaka, Ankidrodroa River, -19.13222, 44.81467, 100 m, tropical dry forest on Tsingy (Fisher-Griswold Arthropod Team) (CAS); PN Tsingy de Bemaraha, 2.5 km 62° ENE Bekopaka, Ankidrodroa River, -19.13222, 44.81467, 100 m, tropical dry forest on Tsingy (Fisher-Griswold Arthropod Team) (CAS); PN Tsingy de Bemaraha, 3.4 km 93° E Bekopaka, Tombeau Vazimba, -19.14194, 44.828, 50 m, tropical dry forest (Fisher-Griswold Arthropod Team) (CAS); PN Tsingy de Bemaraha, 3.4 km 93° E Bekopaka, Tombeau Vazimba, -19.14194, 44.828, 50 m, tropical dry forest (Fisher-Griswold Arthropod Team) (CAS); PN Tsingy de Bemaraha, 3.4 km 93° E Bekopaka, Tombeau Vazimba, -19.14194, 44.828, 50 m, tropical dry forest (Fisher-Griswold Arthropod Team) (CAS); Réserve d’Ankoririka, 10.6 km 13° NE de Tsaramandroso, -16.26722, 47.04861, 210 m, tropical dry forest (Fisher, Griswold et al.) (CAS); RS Bemarivo, 23.8 km 223° SW Besalampy, -16.925, 44.36833, 30 m, tropical dry forest (Fisher, Griswold et al.) (CAS); Sofia Region, District of Sofia, Anjiamangirana 45 km S Antsohihy, Analagnambe Galery forest, 5 km W Anjiamangirana, -15.157, 47.73417, 97 m, low degraded dry forest (Mike, Rinha) (CAS). **Toliara**: 48 km ENE Morondava, -20.06667, 44.65, 30 m, tropical dry forest (D.M. Olson) (CAS); 50 kms N Morondava, -20.06667, 44.58333, in primary dry forest (A. Pauly) (CAS), Androy Region, District of Tsihombe, 74 km S of Tsihombe, RS Cap Ste Marie, -25.58767, 45.163, 36 m, spiny bush (Rin’Ha, Mike) (CAS); Anosy Region, Anosyenne Mts, 29.33 km NW Manantenina, -24.13993, 47.07418, 540 m, montane rainforest (B.L. Fisher, F.A. Esteves et al.) (CAS); Anosy Region, Anosyenne Mts, 32.5 km NW Manantenina, -24.14098, 47.03689, 1900 m, montane rainforest (B.L. Fisher, F.A. Esteves et al.) (CAS); Anosy Region, District of Amboasary, 58 km SW of Fort Dauphin, 08 km NW of Amboasary, Berenty Special Reserve, -25.00667, 46.30333, 85 m, Galery forest (Rin’Ha, Mike) (CAS); Anosy Region, District of Amboasary, 58 km SW of Fort Dauphin, 08 km NW of Amboasary, Berenty Special Reserve, -25.021, 46.3055, 36 m, spiny forest (Mike, Rin’ha) (CAS); Anosy Region, District of Fort-Dauphin, PN Andohahela, Parcelle II, Tsimela, 42 km W of Fort-Dauphin, -24.93683, 46.62667, 176 m, transition forest (Michael Irwin, Frank Parker, Rin’ha) (CAS); Anosy Region, District of Fort-Dauphin, PN Andohahela, Parcelle II, Tsimela, 42 km W of Fort-Dauphin, -24.93683, 46.62667, 176 m, transition forest (Michael Irwin, Frank Parker, Rin’ha) (CAS); Anosy Region, District of Fort-Dauphin, PN Andohahela, Parcelle II, Tsimela, 42 km W of Fort-Dauphin, -24.93683, 46.62667, 176 m, transition forest (Michael Irwin, Frank Parker, Rin’ha) (CAS); Anosy Region, District of Fort-Dauphin, PN Andohahela, Parcelle II, Tsimela, 42 km W of Fort-Dauphin, -24.93683, 46.62667, 176 m, transition forest (Michael Irwin, Frank Parker, Rin’ha) (CAS); Anosy Region, District of Fort-Dauphin, PN Andohahela, Parcelle II, Tsimela, 42 km W of Fort-Dauphin, -24.93683, 46.62667, 176 m, transition forest (Michael Irwin, Frank Parker, Rin’ha) (CAS); Anosy Region, District of Fort-Dauphin, PN Andohahela, Parcelle II, Tsimela, 42 km W of Fort-Dauphin, -24.93683, 46.62667, 176 m, transition forest (Michael Irwin, Frank Parker, Rin’ha) (CAS); Anosy Region, District of Fort-Dauphin, PN Andohahela, Parcelle II, Tsimela, 42 km W of Fort-Dauphin, -24.93683, 46.62667, 176 m, transition forest (Michael Irwin, Frank Parker, Rin’ha) (CAS); Anosy Region, District of Fort-Dauphin, PN Andohahela, Parcelle II, Tsimela, 42 km W of Fort-Dauphin, -24.93683, 46.62667, 176 m, transition forest (Michael Irwin, Frank Parker, Rin’ha) (CAS); Anosy Region, District of Fort-Dauphin, PN Andohahela, Parcelle II, Tsimela, 42 km W of Fort-Dauphin, -24.93683, 46.62667, 176 m, transition forest (Michael Irwin, Frank Parker, Rin’ha) (CAS); Anosy Region, District of Fort-Dauphin, PN Andohahela, Parcelle II, Tsimela, 42 km W of Fort-Dauphin, -24.93683, 46.62667, 176 m, transition forest (Michael Irwin, Frank Parker, Rin’ha) (CAS); Anosy Region, District of Fort-Dauphin, PN Andohahela, Parcelle II, Tsimela, 42 km W of Fort-Dauphin, -24.93683, 46.62667, 176 m, transition forest (Michael Irwin, Frank Parker, Rin’ha) (CAS); Anosy Region, District of Fort-Dauphin, PN Andohahela, Parcelle II, Tsimela, 42 km W of Fort-Dauphin, -24.93683, 46.62667, 176 m, transition forest (Michael Irwin, Frank Parker, Rin’ha) (CAS); Anosy Region, District of Fort-Dauphin, PN Andohahela, Parcelle II, Tsimela, 42 km W of Fort-Dauphin, -24.93683, 46.62667, 176 m, transition forest (Michael Irwin, Frank Parker, Rin’ha) (CAS); Anosy Region, PN Andohahela, Forêt de Manatalinjo, -24.82466, 46.60111, 100 m, spiny forest/thicket (B.L. Fisher, F.A. Esteves et al.) (CAS); Anosy Region, PN Andohahela, Forêt de Manatalinjo, -24.82505, 46.57811, 90 m, spiny forest/thicket (B.L. Fisher, F.A. Esteves et al.) (CAS); Atsimo Andrefana Region, District of Betioky ; RS Beza Mahafaly Parcelle Belle vue 07 km W of Research Station, -23.68983, 44.5755, 177 m, spiny forest, (Rin’ha) (CAS); Berenty Reserve, -24.98333, 46.3, 30 m, gallery forest (D.M. Olson) (CAS); Forêt de Kirindy, 15.5 km 64° ENE Marofandilia, -20.045, 44.66222, 100 m, tropical dry forest (Fisher-Griswold Arthropod Team) (CAS); Forêt de Mahavelo, Isantoria River, -24.75833, 46.15717, 110 m, spiny forest/thicket (Fisher-Griswold Arthropod Team) (CAS); Forêt de Mite, 20.7 km 29° WNW Tongobory, -23.52417, 44.12133, 75 m, gallery forest (Fisher-Griswold Arthropod Team) (CAS); Forêt Vohidava 88.9 km N Amboasary, -24.24067, 46.28783, 500 m, spiny forest/dry forest transition (B.L. Fisher et al.) (CAS); Mahafaly Plateau, 6.2 km 74° ENE Itampolo, -24.65361, 43.99667, 80 m, spiny forest/thicket (Fisher-Griswold Arthropod Team) (CAS); Makay Mts., -21.34228, 45.18314, 410 m, Gallery forest on sandy soil (B.L. Fisher et al.) (CAS); Makay Mts., -21.21836, 45.3106, 510 m, Gallery forest on sandy soil (B.L. Fisher et al.) (CAS); Makay Mts., -21.22284, 45.32477, 490 m, Gallery forest on sandy soil (B.L. Fisher et al.) (CAS); Manderano, -23.5275, 44.08833, 70 m, gallery forest (Frontier Project) (CAS); Manombo, -22.807, 43.76273, 218 m, gallery forest, TS1 (Frontier Wilderness Project) (CAS); Menabe Region, District of Morondava, Beroboka village 45 km NE of Morondava, Antsarongaza dry forest 07,5 km E of Beroboka, -19.9775, 44.66633, 50 m, dry forest (M. Irwin, Rin’ha) (CAS); Menabe Region, District of Morondava, Beroboka village 45 km NE of Morondava, Antsarongaza galery forest 07 km E of Beroboka, -19.9775, 44.66533, 45 m, Galery forest (M. Irwin, Rin’ha) (CAS); PN Andohahela, Forêt d’Ambohibory, 1.7 km 61° ENE Tsimelahy, 36.1 km 308° NW Tolagnaro, -24.93, 46.6455, 300 m, tropical dry forest (Fisher-Griswold Arthropod Team) (CAS); PN Kirindy Mite, 16.3 km 127° SE Belo sur Mer, -20.79528, 44.147, 80 m, tropical dry forest (Fisher-Griswold Arthropod Team) (CAS); Ranobe, -23.03952, 43.61015, 20 m, gallery forest (Frontier Wilderness Project) (CAS); RS Beza Mahafaly, Parcel 1, -23.65, 44.63333, 130 m, tropical dry forest (P.S. Ward) (CAS); Réserve Berenty, -25.01667, 46.3, 25 m, tropical dry forest (P. S. Ward) (CAS); Réserve Privé Berenty, Forêt de Bealoka, Mandraré River, 14.6 km 329° NNW Amboasary, -24.95694, 46.2715, 35 m, gallery forest (Fisher-Griswold Arthropod Team) (CAS); Réserve Privé Berenty, Forêt de Malaza, Mandraré River, 8.6 km 314° NW Amboasary, -25.00778, 46.306, 40 m, gallery forest (Fisher-Griswold Arthropod Team) (CAS); RS Ambohijanahary, Forêt d’Ankazotsihitafototra, 35.2 km 312 °NW Ambaravaranala, -18.26667, 45.40667, 1050 m, montane rainforest (Fisher, Griswold et al.) (CAS); Sept Lacs, -23.52083, 44.15972, 120 m, gallery forest (Frontier Project) (CAS); Southern Isoky-Vohimena Forest, -22.68333, 44.83333, 730 m (Sylvain) (CAS); 5 km N Ampotaka, malaise on trail in Vitambany gallery forest, -24.65033, 43.96317, 86 m, Gallery forest (M.E. Irwin, Rin’ha) (CAS), Ambohimahavelona village 33 km NE of Tulear, Andoharano dry forest, -23.44083, 43.89967, 46 m, dry forest (M.E. Irwin, Rin’ha) (CAS); PN Andohahela Tsimelahy, -24.93683, 46.62667, 180 m, transition forest (M.E. Irwin, F.D. Parker, R. Harin’Hala) (CAS); Berenty Special Reserve, 8 km NW Amboasary, 58 km SW of Fort Dauphin, -25.00667, 46.30333, 85 m, gallery forest (M.E. Irwin, F.D. Parker, R. Harin’Hala) (CAS); near ANGAP office, PN Zombitse, -22.8865, 44.69217, 840 m, deciduous spiny forest (R. Harin’Hala) (CAS); near road, PN Zombitse, -22.8405, 44.73117, 825 m, spiny deciduous forest (R. Harin’Hala) (CAS); Parcel I, RS Beza Mahafaly, near research station, -23.6865, 44.591, 165 m, dry deciduous forest (R. Harin’Hala) (CAS); Parcel II, RS Beza Mahafaly, near Bellevue, -23.68983, 44.5755, 180 m, spiny forest (R. Harin’Hala) (CAS); Tsimelahy - Parcel II, PN Andohahela, transition forest, -24.93683, 46.62667, 180 m, transition forest (M.E. Irwin, F.D. Parker, R. Harin’Hala) (CAS); Makay, -21.3133, 45.14788, 520 m, gallery forest (J.M. Bichain) (CAS); Makay, -21.35695, 45.19021, 401 m, xerophylic vegetation (J.M. Bichain) (CAS); Makay, -21.22283, 45.32453, 525 m, gallery forest (J.M. Bichain) (CAS); Makay, -21.21882, 45.33289, 542 m, gallery forest (J.M. Bichain) (CAS); Kirindy Forest, plot II, -20.07212, 44.65531, 50 m, dry forest (B.B. Blaimer) (CAS).

##### Diagnosis.

In full-face view, lateral cephalic margins converging posteriorly towards eye level, posterior to eye level covered with erect hairs; two apical teeth of mandible closely spaced; head and gaster reddish black and mesosoma reddish orange or integument entirely reddish black.

##### Description.

**Minor worker.** In full-face view, lateral margins slightly converging progressively towards level of anterior border of eye, strongly converging to a short posterior margin; eye large and broadly convex (EL/CS: 0.27±0.01; 0.25–0.29), breaking lateral cephalic margin, level of its posterior margin located at ca. posterior 1/3 of head (PoOc/CL: 0.28±0.01; 0.26–0.29); frontal carinae medially diverging (FR/CS: 0.24±0.01; 0.23–0.27), posteriorly parallel; clypeus without well-defined anterolateral angle, its anteromedian margin broadly convex, with anterolateral angle and anteromedian margin medially slightly concave; mandible with two apical teeth closely placed; antennal scape relatively long (SL/CS: 1.70±0.08; 1.50–1.81). Pronotum and anterior section of mesonotum weakly convex, posterior portion of mesonotum flat immediately anterior to a barely visible metanotal groove; propodeal dorsum anteriorly convex and posteriorly flat, rounding to declivity; propodeal dorsum ca. 3 × as long as declivity. Petiolar node approximately conical or with short anterior face and posteriorly inclined dorsal margin; femur of hind leg axially rounded, not twisted basally.

First and second gastral tergites without a pair of white spots; head with numerous erect hairs on lateral margin, more than six erect hairs present near its posterior margin; antennal scape covered in suberect hairs inclined at ca. 30°; promesonotum, posterior portion of propodeum, and higher level of declivity covered with more or less long, erect hairs.

**Major worker.** With characteristics of minor worker except: broader head (CS: 14.07±0.19; 3.81–4.40; CWb/CL: 0.89±0.04; 0.85–0.96), apical 1/3 of antennal scape extending beyond posterior cephalic margin; two apical teeth of mandible normally spaced, usually area ranging from third to basal teeth of mandible divided into two teeth or denticles; mesosoma very robust, promesonotum and metanotum forming even convexity, propodeal dorsum forming separate convexity and rounding to a short declivity surface; petiolar node more flattened anteroposteriorly. Hairs numerous and shorter on body dorsum.

##### Distribution and biology.

**Figure 71.**
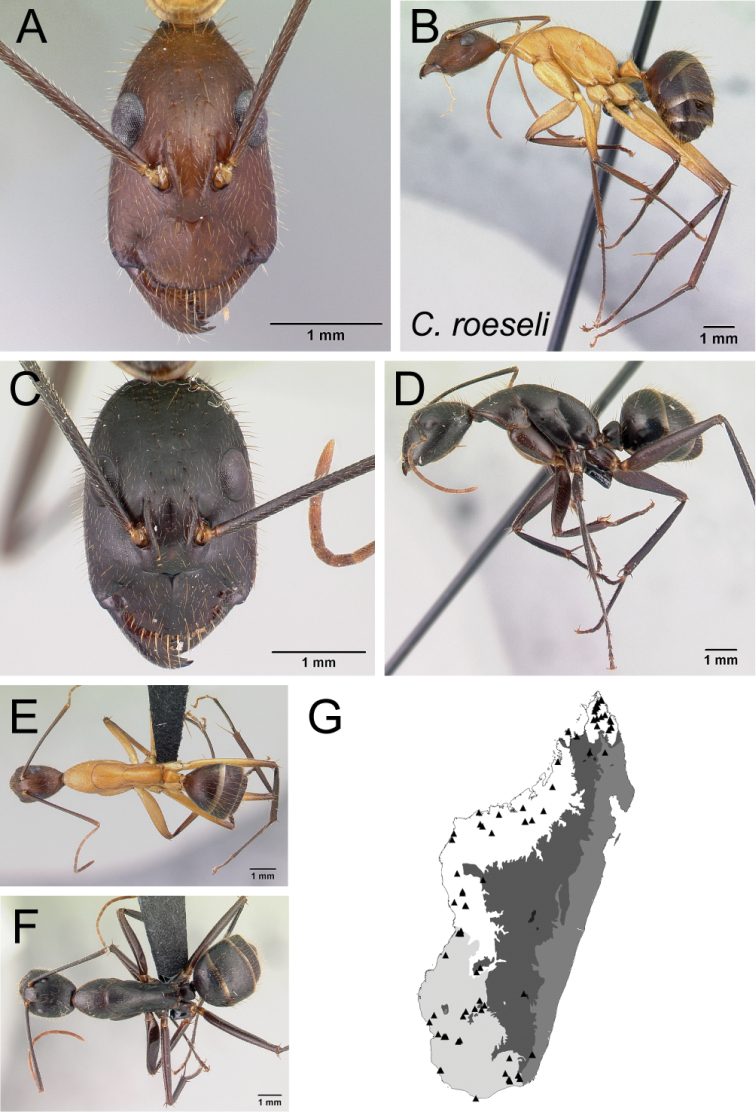
*Camponotusroeseli***A, C** head in full-face view **B, D** lateral view **E, F** dorsal view of minor workers CASENT0492473 and CASENT0179449**G** distribution map.

As an endemic and widespread species of Madagascar, *C.roeseli* generally occurs in the western portion of the island in areas ranging from littoral and rainforest habitats in the north to dry forest areas in the west-central region through spiny forest and thicket in the south (Fig. 71G). This species is also known to occupy savannah, *Uapaca* woodland, montane rainforests, and human-modified habitats of the south-central high plateau. Foraging behavior is mostly carried out on the ground surface and infrequently on low vegetation, while nests are located mainly in rotten logs, in the ground, and under stones, and rarely in rotting tree stumps and under root mat-litter on rocks.

##### Discussion.

*Camponotusroeseli* might be confused with *C.daraina* because of the presence of erect hairs on the lateral margin of the head posterior to the eye level; however, the latter species can be distinguished by the reddish orange color of the entire body.

The species name *C.roeseli* and the subspecies name *C.maculatuslegionarium* were created and described by Forel (1910) and Santschi (1911b), respectively, based on major worker specimens. However, observations of the type specimens of the species and subspecies reveal no robust morphological characters to distinguish them. Samples of the minor workers for *C.roeseli* show striking morphological variation along the western dry forest of Madagascar, especially in body coloration that ranges from dark brown to black through the head; brown gaster; and mesosoma, petiolar node, and the basal portion of the legs yellowish orange. This indicates morphological variation of major workers within the species is likely. Therefore, *C.maculatuslegionarium* is presently synonymized under *C.roeseli*.

The taxonomic identity of *C.roeseli* established by conventional qualitative morphology is supported by multivariate statistical methods. The grouping of samples of this species achieved by the NC-clustering method is confirmed by LDA with an identification success of 100%.

#### ﻿ Camponotus rotrae sp. nov.

Taxon classificationAnimaliaHymenopteraFormicidae

F3D1B794-ADC0-529E-B5FA-6F0DBE7BB716

http://zoobank.org/1E46205A-A4FB-4D92-9B33-2B89217369B1

Figs 39A , 72

##### Holotype worker.

**Madagascar**: Province **Fianarantsoa**: PN Isalo, 9.1 km 354° N Ranohira, -22.48167, 45.46167, 725 m, gallery forest, ex rotten log, 27–31 Jan 2003 (Fisher, Griswold et al.) collection code: BLF07331, specimen code: CASENT0485625 (CAS).

##### Paratype.

1 major worker of same data as holotype but specimen coded as: CASENT0485628 (CAS).

##### Additional material examined.

**Madagascar: Fianarantsoa**: dry wash, 1 km E of PN Isalo Interpretive Center, -22.62667, 45.35817, 885 m, dry wash (R. Harin’Hala) (CAS); Horombe Region, District of Ihosy, PN Isalo, 900 m E of ANGAP Interpretation Center, -22.62667, 45.35817, 701 m, open area near stream (Rin’Ha, Mike) (CAS); PN Andringitra, Forêt Ravaro 12.5 km SW Antanifotsy, -22.21167, 46.845, 1500–1800 m, montane rainforest, (S. Razafimandimby) (CAS); PN Isalo, 9.1 km 354° N Ranohira, -22.48167, 45.46167, 725 m, gallery forest (Fisher, Griswold et al.) (CAS); stream area, 900 m E of PN Isalo Interpretive Center, -22.62667, 45.35817, 750 m, open area near stream (R. Harin’Hala) (CAS). **Mahajanga**: PN Ankarafantsika, Forêt de Tsimaloto, 18.3 km 46° NE de Tsaramandroso, -16.22806, 47.14361, 135 m, tropical dry forest (Fisher, Griswold et al.) (CAS). **Toliara**: FC Analavelona, 29.2 km 343° NNW Mahaboboka, -22.675, 44.19, 1100 m, montane rainforest (Fisher, Griswold et al.) (CAS).

##### Diagnosis.

In full-face view, lateral margins of head anterior to eye level diverging posteriorly; anterior clypeal margin truncate; two pairs of white spots present on second and third abdominal tergites; pronotum, mesonotum, and propodeum not forming separate convexities, metanotal groove not depressed; propodeum immediately in junction with promesonotum; propodeal dorsum straight.

##### Description.

**Minor worker.** In full-face view, lateral margin of head diverging posteriorly towards level of eye, rounding evenly to posterior margin behind eye level; eye protruding and small (EL/CS: 0.24±0.01; 0.22–0.26), not interrupting lateral cephalic border, level of its posterior margin located approximately at posterior 1/4 of head (PoOc/CL: 0.25±0.01; 0.23–0.25); frontal carinae wide (FR/CS: 0.31±0.01; 0.29–0.33), posteriorly diverging, distance between them larger than their smallest distance to eye; clypeus with anterolateral angle and straight anteromedian margin; two apical teeth of mandible normally distant; antennal scape relatively long (SL/CS: 1.19±0.05; 1.08–1.27). Promesonotum weakly convex, mesopropodeum almost flat; metanotal groove barely visible or indistinct; propodeal dorsum nearly straight, its junction to declivity bluntly angulate; propodeal dorsum two and a 1/2 times as long as declivity. Petiolar node short and high, with dorsal margin inclined posteriorly and rounding to anterior margin; anterior and posterior faces at almost the same height; femur of hind leg rounded axially, not twisted near base.

First and second gastral tergites with a pair of white spots; lateral margin of head without erect hairs; two erect hairs present near posterior margin of head; antennal scape covered only with appressed hairs; pronotum with few erect hairs; mesonotum with one pair of erect hairs; posterodorsal corner of propodeum with two pairs of erect hairs. Body color shining brown to dark-brown; apical portion of appendages with lighter color.

**Major worker.** Differing from minor worker in the following characters: larger head (CS: 2.29±0.17; 2.03–2.42; CWb/CL: 1.03±0.01; 1.02–1.04) with more or less straight posterior margin; apical 1/5 of antennal scape surpassing posterior cephalic margin; in lateral view, mesosoma characterized by even convexity of pronotum and mesonotum, separated from propodeum by distinct metanotum; propodeal dorsum strongly sloping to declivity surface and two and a 1/2 times as long as declivity.

##### Distribution and biology.

**Figure 72.**
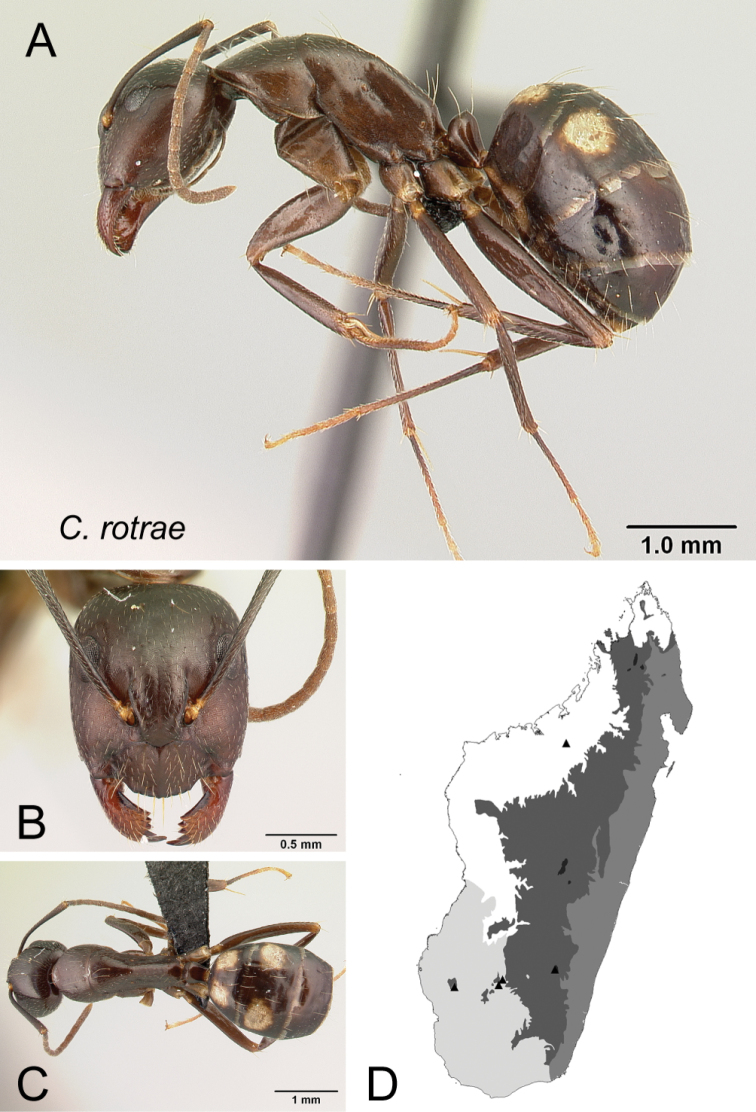
*Camponotusrotrae***A** lateral view **B** head in full-face view **C** dorsal view of holotype minor worker CASENT0485625**D** distribution map.

Endemic to Madagascar, *C.rotrae* is geographically limited to the south-central high plateau of Madagascar. The species occupies montane rainforests of the PN Andringitra, gallery forest and grassland of the PN Isalo, savannah woodland of Itremo, and relict montane rainforest of the Forêt Classée d’Analavelona (Fig. 72D). The species nests mainly in rotten logs and under stones. Based on collection data, workers most often forage on the ground and rarely on lower vegetation.

##### Discussion.

See discussion under *C.gibber*. The qualitative morphological distinction of *C.rotrae* from other species has been confirmed by multivariate analyses of quantitative morphology. The cluster of *C.rotrae* samples in the dendrogram of the NC-clustering is supported by LDA with a classification success of 100%.

##### Etymology.

The species name *rotrae* is a non-Latin singular noun used in apposition.

#### ﻿ Camponotus sambiranoensis sp. nov.

Taxon classificationAnimaliaHymenopteraFormicidae

FA389A21-4FB5-5C6D-9B67-7EFD2420F1A8

http://zoobank.org/5A84A1E2-D64E-4665-BACA-83CEAC5DB8BF

Figs 6A , 9A , 73

##### Holotype worker.

**Madagascar**: Province **Antsiranana**: RS Ankarana, 22.9 km 224° SW Anivorano Nord, -12.90889, 49.10983, 80 m, tropical dry forest, ex rotten log, 10–16 Feb 2001, (Fisher, Griswold et al.) collection code: BLF02880, collection code: CASENT0428077 (CAS).

##### Paratypes.

3 minor workers of the same data as holotype but with the following specimen codes: CASENT0428078, CASENT0428080, CASENT0428079 (NHMUK, MHNG, CAS).

##### Additional material examined.

**Madagascar: Antsiranana**: Ampasindava, Forêt d’Ambilanivy, 3.9 km 181° S Ambaliha, -13.79861, 48.16167, 600 m, rainforest (Fisher, Griswold et al.) (CAS); Binara Forest, -13.26388, 49.60141, 900 m, rainforest (B.L. Fisher et al.) (CAS); District of Vohemar, Analabe Sahaka 43 km E of Daraina, -13.07933, 49.90233, 30 m, wooded grassland-bushland (Mike, Rin’ha) (CAS); Forêt de Binara, 7.5 km 230° SW Daraina, -13.255, 49.61667, 375 m, tropical dry forest (B.L. Fisher et al.) (CAS); Makirovana forest, -14.17066, 49.95409, 415 m, rainforest (B.L. Fisher et al.) (CAS); Nosy-Be Pref., Antsirambazaha, Hell-Ville S.-Pref.: Nosy Be, Site 3, -13.41345, 48.3113, 143 m, in degraded primary rainforest on slope with Nastus (D. Lees & R. Ranaivosolo) (CAS); RS Manongarivo, 10.8 km 229° SW Antanambao, -13.96167, 48.43333, 400 m, rainforest (B.L. Fisher) (CAS); RS Manongarivo, 14.5 km 220° SW Antanambao, -14, 48.43167, 1220 m, montane rainforest (B.L. Fisher) (CAS); RS Ambre, 3.5 km 235° SW Sakaramy, -12.46889, 49.24217, 325 m, tropical dry forest edge (Fisher, Griswold et al.) (CAS); RS Ankarana, 22.9 km 224° SW Anivorano Nord, -12.90889, 49.10983, 80 m, tropical dry forest (Fisher, Griswold et al.) (CAS). SAVA Region, District of Vohemar, Antsahabelela Rain Forest, 9 km SW of Daraina, -13.2505, 49.61667, 182 m, humid Forêt (Mike, Rin’ha) (CAS).

##### Diagnosis.

With head in full-face view, lateral cephalic margins converging posteriorly towards eye level; anteromedian margin of clypeus broadly convex; two apical teeth of mandible normally spaced; lateral cephalic margin anterior to eye level without erect hairs.

##### Description.

**Minor worker.** In full-face view, lateral margins of head converging progressively to level of anterior ocular margin, strongly converging to a short posterior margin behind eye level; eye protruding and large (EL/CS: 0.28±0.01; 0.27–0.29), breaking lateral cephalic border, level of its posterior margin at least at posterior 1/3 of head (PoOc/CL; 0.32±0.04; 0.30–0.42); frontal carinae wide (FR/CS: 0.22±0.0; 0.21–0.23), posteriorly parallel, distance between them larger than smallest distance to eye; clypeus without well-defined anterolateral angle, its anteromedian margin broadly convex or triangular; mandible with two apical teeth closely spaced; antennal scape relatively long (SL/CS: 2.04±0.07; 1.97–2.18). Mesosoma low and long (MPH/ML: 0.33±0.03; 0.28–0.36), with weakly convex promesonotum and approximately flat mesopropodeum; metanotal groove barely visible; propodeal dorsum almost straight or anteriorly slightly convex and posteriorly flat; dorsal margin of propodeum and declivity joined at blunt angle; propodeal declivity 1/3 of length of dorsum. Petiole nodiform, dorsal margin inclined posteriorly, its junction to anterior face bluntly angulate; anterior face of petiolar node 1/2 height of posterior face; femur of hind leg rounded axially, not twisted basally.

First and second gastral tergites without a pair of white spots; lateral margin of head without erect hairs; two erect hairs present near posterior cephalic margin; antennal scape without erect hairs but covered with numerous appressed hairs; pronotum with a pair of erect hairs; posterodorsal angle of propodeum without erect hairs.

**Major worker.** Differing from minor worker in the following characters: larger head (CS: 4.45; CWb/CL: 0.98); apical 1/4 of antennal scape extending beyond posterior cephalic margin; very robust, short, and high mesosoma, with promesonotum and metanotum forming an even convexity; and propodeal dorsum sloping and joining declivity with blunt angle. Petiolar node much compressed anteroposteriorly, anterior face shorter than posterior.

##### Distribution and biology.

**Figure 73.**
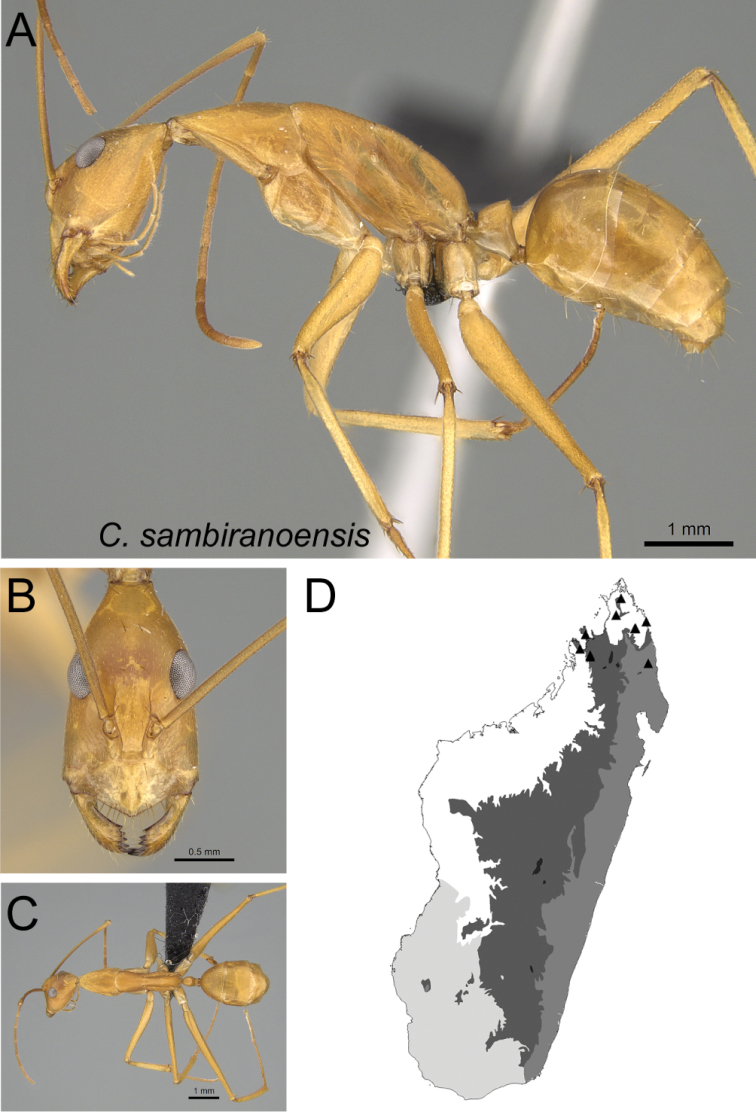
*Camponotussambiranoensis***A** lateral view **B** head in full-face view **C** dorsal view of minor worker CASENT0231845**D** distribution map.

*Camponotussambiranoensis* is only known to occur in the north of Madagascar. It colonizes the dry forests of Binara, RS Ambre, and RS Ankarana, the transitional forests of Ampasindava and Binara, the rainforests of Makirovana and RS Manongarivo, and the montane forest of RS Manongarivo (Fig. 73D). This species nests in rotten logs and in the ground, and forages on the ground, in leaf litter, and on low vegetation.

##### Discussion.

*Camponotussambiranoensis* shares a broadly convex anteromedian margin of clypeus with *C.niavo*, and *C.cervicalis*, but in the two latter species the lateral cephalic margin anterior to the eye level is covered with erect hairs.

The qualitative, morphology-based delimitation of *C.sambiranoensis* is confirmed by the multivariate morphometric technique. The validity of the species is also confirmed by LDA at 100% classification success.

##### Etymology.

The species name *sambiranoensis* is a Latin singular adjective in the nominative case of masculine gender. This species name refers to the region of Sambirano where the species was found.

#### ﻿ Camponotus strangulatus

Taxon classificationAnimaliaHymenopteraFormicidae

Santschi

2D09D46F-7B27-5B47-8699-E9E028733DB3

Figs 29A , 74

Camponotus maculatus st. strangulatus Santschi, 1911a: 129. Lectotype major worker, by present designation, Madagascar, Vitikampy; Morondava, AntWeb CASENT0101097 (NHMB) [examined]. Raised to species by Olson and Ward 1996: 164.
Camponotus hova maculatoides Emery, 1920b: 6. Lectotype minor worker, by present designation, Madagascar, Nosibe, Antsiranana (Voeltzkow) AntWeb CASENT0101095 (NHMB) [examined]. Paralectotypes: 1 minor worker and 3 major workers of same data as lectotype, but minor and majors respectively specimen coded as: CASENT0101339, CASENT0101338, CASENT0101747 (MHNG) and CASENT0101095 (NHMB) [examined]. [First available use of Camponotusmaculatusr.hovavar.maculatoides Forel, 1897: 200]. Syn. nov.
Note: As junior synonym by Emery 1920: 6. [Emery established Camponotusstrangulatus as the junior synonym of Camponotusmaculatoides but the former name has priority: Bolton 1995: 125].

##### Additional material examined.

**Comoros: Grande Comore**: Domani, -11.51778, 43.28, 25 m, coastal scrub (B.L. Fisher et al.) (CAS); Pidjani, -11.75447, 43.45148, 35 m, coastal scrub (B.L. Fisher et al.) (CAS). **Mohéli**: Madahali 50, -12.37421, 43.86857, 50 m, coastal dry forest scrub (B.L. Fisher et al.) (CAS); Ouallah, -12.34234, 43.66851, 1 m, mangrove (B.L. Fisher et al.) (CAS). **Juan De Nova Island**: -17.04891, 42.72102, 10 m, littoral vegetation (B.L. Fisher) (CAS). **Madagascar: Antananarivo**: Analamanga Region, District of Ankazobe, Ambohitantely, 46 km NE of Ankazobe, -18.198, 47.2815, 701 m, Forêt sclerophylle (Mike, Rinha) (CAS); RS Ambohitantely, Forêt d’Ambohitantely, 20.9 km 72° NE d’Ankazobe, -18.22528, 47.28683, 1410 m, montane rainforest (Fisher, Griswold et al.) (CAS). **Antsiranana**: [Loucoubé auf Nossi-Bé]; Nosy be; Nosibe (Carl Bosse) (MHNG); District of Vohemar, Analabe Sahaka 43 km E of Daraina, -13.07933, 49.90233, 30 m, wooded grassland-bushland (Mike, Rin’ha) (CAS); Forêt d’Orangea, 3.6 km 128° SE Remena, -12.25889, 49.37467, 90 m, littoral rainforest (Fisher, Griswold et al.) (CAS); Galoko chain, Mont Kalabenono, -13.64179, 48.67282, 643 m, rainforest (B.L. Fisher et al.) (CAS); Galoko chain, Mont Kalabenono, -13.63999, 48.67374, 498 m, rainforest (B.L. Fisher et al.) (CAS); Nosy Be, 4 km ESE Andoany (=Hellville), -13.41667, 48.3, <5 m, littoral vegetation (P.S. Ward) (CAS); Nosy Be, RNI Lokobe, 6.3 km 112° ESE Hellville, -13.41933, 48.33117, 30 m, rainforest (Fisher, Griswold et al.) (CAS); PN Montagne d’Ambre, Fozalanana, -12.46637, 49.22157, 475 m, dry forest (B.L. Fisher et al.) (CAS); Réserve Analamerana, 16.7 km 123° Anivorano-Nord, -12.80467, 49.37383, 225 m, tropical dry forest (B.L. Fisher) (CAS); SAVA Region District of Vohemar, Andranotsimaty, Dry forest, 9 km NE of Daraina, -13.1695, 49.70067, 90 m, dry dense forest (Mike, Rinha) (CAS); Taizambato, 22.9 km NE Ambanja, -13.5092, 48.56722, 16 m, coconut plantation (B.L. Fisher et al.) (CAS); PN Montagne d’Ambre [1^st^ campsite], 960 m, rainforest (R. Harin’Hala) (CAS); PN Montagne d’Ambre [Petit Lac road], -12.533333, 49.166667, 1125 m, rainforest (R. Harin’Hala) (CAS). **Fianarantsoa**: Amoron’i Mania Region, District of Ambositra, Italaviana *Uapaca* forest, 35 km SE of Antsirabe, -20.17333, 47.086, 1359 m, *Uapaca* forest (Rin’Ha, Mike) (CAS); Atsimo Atsinanana Region, District of Farafangana, Mahabo Mananivo, 50 km S of Farafangana Ampitavananima, Forest low altitude, Littoral forest on sand 2 km E of Mobot office, -23.12983, 47.717, 33 m, Littoral Low Alt Rain Forest (Mike, Frank, Rin’ha) (CAS); Vatovavy Fitovinany Region, District of Ifanadiana, 12 km W of Ranomafana, -21.25083, 47.40717, 1127 m, forest edge, open area (Rin’Ha, Mike) (CAS); RS Manombo, 32 km SE of Farafangana, -23.02183, 47.72, 36 m, Lowland rainforest (Rin’Ha, Mike) (CAS). Miandritsara Forest, 40 km S of Ambositra, -20.79267, 47.17567, 822 m, Low altitude rainforest (Rin’Ha, Mike) (CAS); PN Andringitra, Forêt Ravaro 12.5 km SW Antanifotsy, -22.21167, 46.845, 1500–1800 m, montane rainforest, (S. Razafimandimby) (CAS); radio tower, PN Ranomafana, -21.25833, 47.40717, 1130 m, forest edge, mixed tropical forest, open area (M. Irwin, R. Harin’Hala) (CAS); Vohiparara broken bridge, -21.22617, 47.36983, 1110 m, high altitude rainforest (R. Harin’Hala) (CAS). **Mahajanga**: Ambalahonko, 5.69 km S Antsohihy, -14.8439, 48.01421, 21 m, urban garden (B.L. Fisher et al.) (CAS); Boeny Region, District of Soalala, Namoroka village, Befatika Andranovory 7 km NW of Vilanandro village, -16.46967, 45.39133, 120 m, Dense dry forest (Irwin, Rin’ha) (CAS); Boeny Region: PN Ankarafantsika, Ambodimanga, -16.32342, 46.82443, 75 m, dry forest, Canopy Bootcamp, (A. Karunakaran) (CAS); Boeny Region; District of Soalala, Namoroka 53 km of Soalala, Ambatofolaka dry forest 3 km N of Vilanandro villlage, -16.47333, 45.39133, 105 m, dense dry forest in the mud (Mike, Rin’ha) (CAS); Forêt de Tsimembo, 8.7 km 336° NNW Soatana, -19.02139, 44.44067, 20 m, tropical dry forest (Fisher-Griswold Arthropod Team) (CAS); Melaky Region, District of Maintirano, Asondrodava dry forest against dune 15 km N of Maintirano, -17.96533, 44.0355, 16 m, dry forest (Irwin, Rinha) (CAS); Melaky Region, District of Besalampy, Marofototra dry forest, 17 km W of Besalampy, -16.72167, 44.42367, 51 m, dry wash in the dry forest (Irwin, Rin’ha) (CAS); Melaky Region, District of Besalampy, Analangidro dry forest, 07 km NE of Besalampy, -16.6915, 44.5235, 25 m, dry forest on sand (Irwin, Rin’ha) (CAS); Melaky Region, District of Maintirano, Asondrodava dry forest, 15 km N of Maintirano, -17.96533, 44.0355, 6 m, dry forest (Irwin, Rinha) (CAS); Melaky Region, District of Mintirano, Ampasy 50 km E of Maintirano, -18.004, 44.452, 85 m, dry forest (Mike, Rin’ha) (CAS); Melaky Region; District of Besalampy marofototra palm forest, 17 km W of Besalampy, -16.72167, 44.42367, 10 m, Palm trees on sand (Irwin, Rin’ha) (CAS); PN Ankarafantsika, Forêt de Tsimaloto, 18.3 km 46° NE de Tsaramandroso, -16.22806, 47.14361, 135 m, tropical dry forest (Fisher, Griswold et al.) (CAS); PN Baie de Baly, 12.4 km 337° NNW Soalala, -16.01, 45.265, 10 m, tropical dry forest (Fisher, Griswold et al.) (CAS); PN Namoroka, 9.8 km 300° WNW Vilanandro, -16.46667, 45.35, 140 m, tropical dry forest (Fisher, Griswold et al.) (CAS); PN Tsingy de Bemaraha, 2.5 km 62° ENE Bekopaka, Ankidrodroa River, -19.13222, 44.81467, 100 m, tropical dry forest on Tsingy (Fisher-Griswold Arthropod Team) (CAS); PN Tsingy de Bemaraha, 3.4 km 93° E Bekopaka, Tombeau Vazimba, -19.14194, 44.828, 50 m, tropical dry forest (Fisher-Griswold Arthropod Team) (CAS); Réserve forestière Beanka, 50.2 km E Maintirano, -17.88756, 44.47265, 153 m, tropical dry forest on tsingy (B.L. Fisher et al.) (CAS); RS Bemarivo, 23.8 km 223° SW Besalampy, -16.925, 44.36833, 30 m, tropical dry forest (Fisher, Griswold et al.) (CAS). **Toliara**: [Vitikampy; Morondava; Museum Paris, G. Grandidier 1899]; Morondava, -20.290419, 44.299996, 6 m (CAS); 48 km ENE Morondava, Kirindy, -20.06667, 44.65, 30 m, tropical dry forest (D.M. Olson) (CAS); Ambovombe, -25.1775, 46.09283, 25 m, urban/garden (B.L. Fisher et al.) (CAS); Ampanihy; Behompy, -23.209835, 43.9457622, 268 m, urban/garden (B.L. Fisher et al.) (CAS); Anosy Region, District of Amboasary, 58 km SW of Fort Dauphin, 08 km NW of Amboasary, Berenty Special Reserve, -25.021, 46.3055, 36 m, spiny forest (Mike, Rin’ha) (CAS); Atsimo Andrefana Region, District of Tulear II, Mikea deciduous dry forest 3 km N Andranomavo village, -22.90367, 43.4755, 30 m, Deciduous dry forest (Rin’Ha, Mike) (CAS); Atsimo Andrefana Region, District of Tulear II, Mikea spiny forest 8 km N Andranomavo village, -22.91333, 43.39883, 37 m, spiny forest (Rin’Ha, Mike) (CAS); Atsimo Andrefana Region, District of Tulear II, Private dry forest, Ifaty 30 km N of Tulear, -23.17967, 43.61683, 9 m, vegetation on sandy area (Mike, Rin’ha) (CAS); Betioky, -23.72117, 44.38017, 270 m, urban/garden (B.L. Fisher et al.) (CAS); Beza-Mahafaly, 27 km E Betioky, -23.65, 44.63333, 135 m, tropical dry forest (B.L. Fisher) (CAS); Col du Manangotry, -24.75, 46.8, 600 m, rainforest edge (B.L. Fisher) (CAS); Ehazoara Canyon, 26 km E Betioky, -23.68333, 44.63333, 175 m, tropical dry forest (B.L. Fisher) (CAS); Ejeda, -24.3505, 44.516, 250 m, urban/garden (B.L. Fisher et al.) (CAS); Fiherenana, -23.23528, 43.87083, 50 m, degraded gallery forest (Frontier Project) (CAS); Fiherenana, -23.22252, 43.88088, 65 m, gallery forest, degraded (Frontier Wilderness Project) (CAS); Forêt de Beroboka, 5.9 km 131° SE Ankidranoka, -22.23306, 43.36633, 80 m, tropical dry forest (Fisher-Griswold Arthropod Team) (CAS); Forêt de Mahavelo, Isantoria River, -24.75833, 46.15717, 110 m, spiny forest/thicket (Fisher-Griswold Arthropod Team) (CAS); Forêt de Mite, 20.7 km 29° WNW Tongobory, -23.52417, 44.12133, 75 m, gallery forest (Fisher-Griswold Arthropod Team) (CAS); Forêt de Tsinjoriaky, 6.2 km 84° E Tsifota, -22.80222, 43.42067, 70 m, spiny forest/thicket (Fisher-Griswold Arthropod Team) (CAS); Kirindy Forest, plot I, -20.07434, 44.67247, 60 m, dry forest (E. Lokensgard) (CAS); Mahafaly Plateau, 6.2 km 74° ENE Itampolo, -24.65361, 43.99667, 80 m, spiny forest/thicket (Fisher-Griswold Arthropod Team) (CAS); Mahafaly, near Eloeste, By Lac Tsimanampetsoa, -24.16667, 43.75, (V. & B. Roth) (CAS); Manderano, -23.5275, 44.08833, 70 m, gallery forest (Frontier Project) (CAS); Menabe Region, District of Morondava, Beroboka village 45 km NE of Morondava, Antsarongaza dry forest 07,5 km E of Beroboka, -19.9775, 44.66633, 50 m, dry forest (M. Irwin, Rin’ha) (CAS); Menabe Region, District of Morondava, Beroboka village 45 km NE of Morondava, Antsarongaza galery forest 07 km E of Beroboka, -19.9775, 44.66533, 45 m, Galery forest (M. Irwin, Rin’ha) (CAS); PN Andohahela, Forêt de Manatalinjo, 33.6 km 63° ENE Amboasary, 7.6 km 99° E Hazofotsy, -24.81694, 46.61, 150 m, spiny forest/thicket (Fisher-Griswold Arthropod Team) (CAS); PN Kirindy Mite, 16.3 km 127° SE Belo sur Mer, -20.79528, 44.147, 80 m, tropical dry forest (Fisher-Griswold Arthropod Team) (CAS); PN Tsimanampetsotsa, 6.7 km 130° SE Efoetse, 23.0 km 175° S Beheloka, -24.10056, 43.76, 25 m, spiny forest/thicket (Fisher-Griswold Arthropod Team) (CAS); PN Tsimanampetsotsa, Mitoho Cave, 6.4 km 77° ENE Efoetse, 17.4 km 170° S Beheloka, -24.04722, 43.75317, 40 m, spiny forest/thicket (Fisher-Griswold Arthropod Team) (CAS); Ranobe, -23.04055, 43.6096, 30 m, spiny forest/thicket (Frontier Project) (CAS); Ranobe, -23.04067, 43.60973, 20 m, gallery forest, degraded (Frontier Wilderness Project) (CAS); Ranobe, -23.03883, 43.60982, 30 m, gallery forest (Frontier Wilderness Project) (CAS); Ranobe, -23.03952, 43.61015, 20 m, gallery forest (Frontier Wilderness Project) (CAS); Ranobe, -23.0342, 43.61185, 30 m, spiny forest/thicket (Frontier Project) (CAS); Ranobe, -23.04457, 43.61532, 20 m, spiny forest/thicket (Frontier Wilderness Project) (CAS); RS Beza-Mahafaly, Parcel 1, -23.65, 44.63333, 130 m, tropical dry forest (P.S. Ward) (CAS); Réserve Privé Berenty, Forêt de Bealoka, Mandraré River, 14.6 km 329° NNW Amboasary, -24.95694, 46.2715, 35 m, gallery forest (Fisher-Griswold Arthropod Team) (CAS); Sept Lacs, -23.5275, 44.15444, 70 m, Gallery forest (Frontier Project) (CAS); 3 km E Itampolo, malaise across path of lower bench of Andrimpano Forest, -24.65783, 43.95617, 45 m, dry forest (M.E. Irwin, Rin’ha) (CAS); 5 km E Itampolo, malaise across path of plateau of Andrimpano Forest, -24.65033, 43.96317, 130 m, dry forest (M.E. Irwin, Rin’ha) (CAS); 5 km N Ampotaka, malaise on trail in Vitambany gallery forest, -24.65033, 43.96317, 86 m, Gallery forest (M.E. Irwin, Rin’ha) (CAS); Ambohimahavelona village 33 km NE of Tulear, Andoharano dry forest, -23.44083, 43.89967, 46 m, dry forest (M.E. Irwin, Rin’ha) (CAS); Ambohimahavelona village 33 km NE of Tulear, private spiny bush, -23.44083, 43.89967, 43 m, spiny bush (M.E. Irwin, Rin’ha) (CAS). Andranovato, Euphorbia forest 5 km SE of Manombo, -22.81806, 43.50217, 18 m, Ephorbia forest (CAS); Atsimo Andrefana Region, District of Tulear II,Tsifota 20 km N of Manombo, -22.818, 43.37267, 15 m, spiny forest (M.E. Irwin, Rin’ha) (CAS); Itampolo, Sud A Sud Hotel, malaise in dune vegetation, -24.6905, 43.944, 12 m, littoral bush (M.E. Irwin, Rin’ha) (CAS); Mikea Forest, deciduous dry forest, -22.90367, 43.4755, 30 m, deciduous dry forest (M.E. Irwin, F.D. Parker, R. Harin’Hala) (CAS); Mikea Forest, spiny forest, -22.91333, 43.48222, 37 m, spiny forest (R. Harin’Hala) (CAS); Parcel I, RS Beza Mahafaly, near research station, -23.6865, 44.591, 165 m, dry deciduous forest (R. Harin’Hala) (CAS); Parcel II, RS Beza Mahafaly, near Bellevue, -23.68983, 44.5755, 180 m, spiny forest (R. Harin’Hala) (CAS); PN Tsimanampetsotsa, Mitoho Forest, malaise across trail at escarpment base, -24.0485, 43.75233, 120 m, dense dry forest (M.E. Irwin, Rin’ha) (CAS); Tsimelahy - Parcel II, PN Andohahela, transition forest, -24.93683, 46.62667, 180 m, transition forest (M.E. Irwin, F.D. Parker, R. Harin’Hala) (CAS). **Mayotte**: Baie de Tsingoni, -12.7926, 45.10764, 5 m, mangrove, coastal scrub (B.L. Fisher et al.) (CAS); Coconi, DAF Campus, -12.83333, 45.13333 (R. Jocqué & G. DeSmet) (CAS); Tanaraki, -12.75754, 45.0678, 10 m, coastal scrub (B.L. Fisher et al.) (CAS). **Seychelles**: Aldabra Atoll, Grande Terre Island, -9.43453, 46.45767, 7 m, Takamaka forest, S.M. Goodman (CAS); Aldabra Island, Picard, old settlement, -9.39606, 46.20465, 2 m, coastal scrub (B.L. Fisher) (NHMB).

##### Diagnosis.

With head in full-face view, lateral cephalic margins anterior to eye level parallel, lacking erect hairs; in oblique profile, three pairs of erect hairs arranged successively from level of anterior ocular margin towards posterior cephalic margin; clypeus with distinct anterolateral corner; in profile, junction of propodeal dorsum to declivity rounded; petiole nodelike and not anteroposteriorly compressed.

##### Description.

**Minor worker.** In full-face view, head sides anterior to eye level parallel, converging strongly towards posterior margin behind eye level; eye convex (EL/CS: 0.28±0.01; 0.26–0.30), breaking lateral cephalic margin, level of its posterior margin located approximately at posterior 1/3 of head (PoOc/CL: 0.27±0.01; 0.26–0.29); frontal carinae close, distance between them equal to or smaller than their smallest distance to eye (FR/CS: 0.25±0.01; 0.24–0.27); clypeus with anterolateral angle and an almost straight or bluntly angulate anteromedian margin; two apical teeth of mandible normally spaced; antennal scape relatively long (SL/CS: 1.58±0.05; 1.50–1.66). Mesosoma low and long (MPH/ML: 0.33±0.01; 0.31–0.36); promesonotum weakly convex, mesonotum and propodeum nearly flat, separated by weakly visible metanotal groove; propodeal dorsum rounding to declivity; propodeal dorsum roughly 2 × as long as declivity. Petiolar node tapering dorsally, its dorsal margin inclined posteriorly, rounding to anterior margin; anterior face 1/2 height of posterior face; femur of hind leg rounded axially, not twisted near base.

First and second gastral tergites without a pair of white spots; lateral margin of head without erect hairs; a pair of erect hairs present near posterior cephalic margin; with head in profile, three pairs of erect hairs arranged from level of anterior margin of eye to posterior cephalic margin; antennal scape without erect hairs, covered with distantly spaced pubescence; pronotum with two pairs of erect hairs; mesonotum and propodeum without erect hairs.

**Major worker.** With characteristics of minor worker, except for the following divergent features: enlarged head (CS: 3.22±0.36; 2.73–3.74; CWb/CL: 0.91±0.06; 0.80–0.98), apical 1/4 of antennal scape surpassing posterior cephalic margin, much more robust mesosoma, promesonotum forming a convexity, separated from propodeum by a distinct metanotum; propodeal dorsum convex immediately behind metanotum, length almost equal to height of declivity, their junction at a rounded angle; petiolar node tapering dorsally.

##### Distribution and biology.

**Figure 74.**
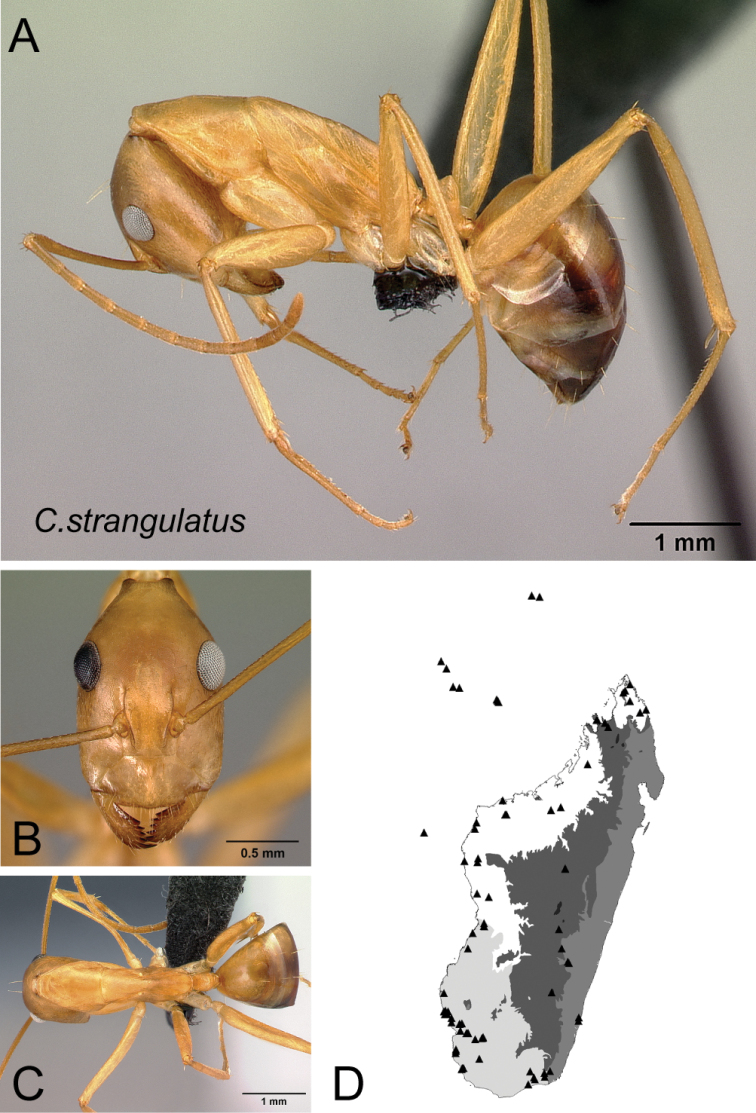
*Camponotusstrangulatus***A** lateral view **B** head in full-face view **C** dorsal view of minor worker CASENT0132660**D** distribution map.

Endemic to the Malagasy region, *C.strangulatus* occurs in Grand Comore and Moheli of the Comoros islands, in Mayotte, in Aldabra of the Seychelles, and in Madagascar (Fig. 74D). In the Comoros and Mayotte this species occupies mangrove and coastal dry forest scrub, while in the Seychelles it also inhabits forest habitats. Regarding the distribution of *C.strangulatus* in Madagascar, it is found in rainforest and littoral rainforest in the north, in dry forest in the west, in spiny forest and thicket in the south, in montane rainforest and *Uapaca* woodland on the south-central high plateau, and in human-modified habitats in its southern range. It also occurs in littoral vegetation on Juan de Nova Island. Due to its capacity to colonize different types of habitats, *C.strangulatus* also builds nests in a large array of microhabitats such as rotten logs and rotten sticks on the ground, dead branches and dead twigs above the ground, under stones, and in the ground. Workers forage on the forest floor, in leaf litter, and on lower vegetation.

##### Discussion.

*Camponotusstrangulatus* may be difficult to distinguish from *C.tapia* and *C.atimo* but the dorsum of the head in the latter species bears four or more pairs of erect hairs arranged successively from the level of the anterior ocular margin towards the posterior cephalic margin.

As generally known, the genus *Camponotus* displays polymorphism in the worker castes and this feature is amplified by the phenotypic variation within the castes when the species has specifically a large geographic distribution. This is especially true for *C.strangulatus* which inhabits most of the terrestrial landscapes, but not the rainforests in the east of Madagascar. *Camponotushovamaculatoides*, created by Emery (1920b) was found in Nosibe, north of Madagascar, an area within the range of *C.strangulatus*. Observations of the type specimens of the species and subspecies prove the absence of strong morphological traits to differentiate them. The comparison of the type specimens of *C.hovamaculatoides* with the samples of workers for *C.strangulatus* did not show striking morphological difference. Consequently, *C.hovamaculatoides* is placed under synonymy here.

Species delimitation of *C.strangulatus* on the basis of traditional taxonomic study is congruent with the results of the NC-clustering method. The species was also classified correctly at 100% by confirmatory LDA.

#### ﻿ Camponotus tapia sp. nov.

Taxon classificationAnimaliaHymenopteraFormicidae

6EF02710-6092-5773-AB46-26B8152E3F55

http://zoobank.org/C050A383-5A8A-4437-A01A-8AC5746E3F70

**Figure 75.**
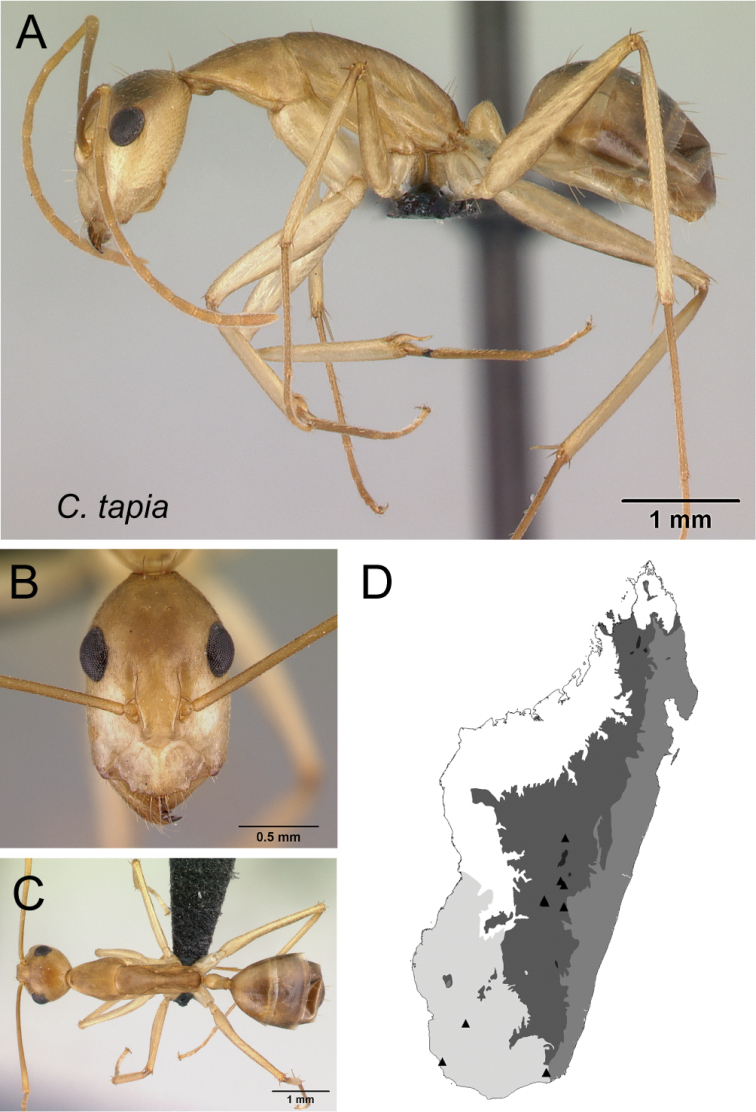
*Camponotustapia***A** lateral view **B** head in full-face view **C** dorsal view of minor worker CASENT0217310**D** distribution map.

Figs 15B , 25A , 29B , 30B , 75

##### Holotype worker.

**Madagascar**: Province **Fianarantsoa**: Forêt d’Atsirakambiaty, 7.6 km 285° WNW Itremo, -20.59333, 46.56333, 1550 m, grassland, under stone, 22–26 Jan 2003 (Fisher, Griswold et al.) collection code: BLF07214, specimen code: CASENT0493939 (CAS).

##### Paratypes.

5 minor workers and 1 major worker with same data as holotype but respectively with specimen codes: CASENT0826114, CASENT0826115, CASENT0493938, CASENT0837568, CASENT0837569, CASENT0493942 (NHMUK, MHNG, MSNG, PBZT, CAS).

##### Additional material examined.

**Madagascar: Antananarivo**: Andohony IV Non Protected Area, 22.51 km SW Antsirabe, -20.06718, 46.99274, 1526 m, *Uapaca* woodland (A. Ravelomanana) (CAS); Beapombo I Non Protected Area, 22.51 km SW Antsirabe, -20.06892, 47.00404, 1663 m, Savannah grassland (A. Ravelomanana) (CAS); Beapombo II Non Protected Area, 22.65 km SW Antsirabe, -20.07022, 47.00555, 1689 m, Savannah grassland (A. Ravelomanana) (CAS); Navoatra III Non Protected Area, 7.39 km NW Arivonimamo, -18.98028, 47.12071, 1321 m, *Uapaca* woodland (A. Ravelomanana) (CAS); Navoatra V Non Protected Area, 7.76 km NW Arivonimamo, -18.97667, 47.11889, 1316 m, *Uapaca* woodland (A. Ravelomanana) (CAS). **Fianarantsoa**: Forêt d’Atsirakambiaty, 7.6 km 285° WNW Itremo, -20.59333, 46.56333, 1550 m, grassland (Fisher, Griswold et al.) (CAS); Amoron’i Mania Region, District of Ambositra, Italaviana *Uapaca* forest, 35 km SE of Antsirabe, -20.17333, 47.086, 1359 m, *Uapaca* forest (Rin’Ha, Mike) (CAS); Ampangabe III Non Protected Area, 21.26 km W Itremo, -20.6125, 46.60883, 1412 m, savannah woodland (A. Ravelomanana) (CAS); Ampangabe VII Non Protected Area, 21.2 km W Itremo, -20.61417, 46.60989, 1420 m, Shrubland (A. Ravelomanana) (CAS); Antapia IV Non Protected Area, 26.42 km SW Ambositra, -20.71917, 47.0868, 1494 m, *Uapaca* woodland (A. Ravelomanana) (CAS); Antohatsahomby III Non Protected Area, 22.79 km NW Itremo, -20.54806, 46.58599, 1499 m, *Uapaca* woodland (A. Ravelomanana) (CAS); Mampiarika III Non Protected Area, 28.93 km SW Ambositra, -20.73583, 47.08399, 1487 m, *Uapaca* woodland (A. Ravelomanana) (CAS).

##### Diagnosis.

With head in full-face view, lateral margins of head anterior to eye level parallel, lacking erect hairs; clypeus with distinct anterolateral corner; in oblique profile, four or more pairs of erect hairs arranged successively from level of anterior ocular margin towards posterior cephalic margin; in profile, junction of propodeal dorsum to declivity surface broadly angulate; petiole nodelike and not anteroposteriorly compressed.

##### Description.

**Minor worker.** In full-face view, head sides anterior to level of eye approximately parallel, strongly converging to form a short area posterior to level of eye; eye protruding and large (EL/CS: 0.29±0.01; 0.28–0.31), breaking lateral cephalic margin, level of its posterior margin located at ca. posterior 1/4 of head (PoOc/CL: 0.24±0.01; 0.23–0.25); frontal carinae close to each other, their distance equal to or smaller than their smallest distance to the eye (FR/CS: 0.25±0.01; 0.23–0.27); clypeus with anterolateral angle, its anteromedian margin broadly triangular or convex; mandible with six teeth, its two apical teeth distantly spaced; antennal scape relatively long (SL/CS: 1.61±0.08; 1.48–1.71). Promesonotum weakly convex, mesopropodeum almost flat; mesonotum flat immediately anterior to metanotal groove; metanotal groove weakly visible; propodeal dorsum anteriorly slightly convex and posteriorly flat, joining declivity at a blunt angle; propodeal declivity 1/3 length of dorsum. Petiole nodiform, its dorsal margin rounding to anterior face, posterior face higher than anterior; femur of hind leg rounded axially, without twist basally.

First and second gastral tergites without a pair of white spots; erect hairs on lateral margin of head absent; posterior margin of head with two erect hairs; with head in profile, four pairs of erect hairs arranged from level of anterior margin of eye to posterior cephalic margin; antennal scape covered only with appressed hairs; pronotum with a pair of erect hairs; posterodorsal angle of propodeum with 2–4 erect hairs.

**Major worker.** Differing from minor worker in the following characters: enlarged head (CS: 3.20±0.21; 2.84–3.38; CWb/CL: 0.99±0.03; 0.94–1.01) with noticeably concave posterior margin; apical 1/3 of antennal scape surpassing posterior cephalic margin; robust mesosoma, propodeal dorsum convex immediately posterior to metanotum, propodeal dorsum < 2 × as long as height of declivity, rounded at junction; petiolar node tapering dorsally.

##### Distribution and biology.

The distribution of *C.tapia* is limited to the high central plateau of Madagascar (Fig. 75D). The species inhabits grassland, savannah woodland, shrubland, and *Uapaca* woodland in the region. Across these areas, this species nests most often under stones and in rotting tree stumps, and forages mainly on the ground.

##### Discussion.

See discussion under *C.atimo*.

In the present study, the definition of *C.tapia* is based on both qualitative morphological analysis and morphometrics, which are congruent. The grouping of the samples of this species obtained from the NC-clustering is confirmed by LDA with an identification success of 100%.

##### Etymology.

The species name *tapia* is non-Latin singular noun used in apposition and refers to the Malagasy name for *Uapaca* in reference to the type of habitat where the ant was most frequently found.

#### ﻿ Camponotus tendryi sp. nov.

Taxon classificationAnimaliaHymenopteraFormicidae

32357F97-D421-5DCB-876A-C539624B5770

http://zoobank.org/19D46B05-61F9-4423-BD96-0F41A74DA3E9

Figs 11B , 12B , 76

##### Holotype worker.

**Madagascar**: Province **Toamasina**: RNI Betampona, 34.08 km 332° Toamasina, -17.91977, 49.20039, 525 m, rainforest, Malaise trap, 06 Apr 2008–01 Feb 2009 (B.L. Fisher et al.) collection code: BLF19594, specimen code: CASENT0145284 (CAS).

##### Paratypes.

3 minor workers with same data as holotype but specimen coded as: CASENT0138778, CASENT0138799, CASENT0138800 (NHMUK, PBZT, CAS).

##### Additional material examined.

**Madagascar: Antsiranana**: Forêt de Binara, 9.1 km 233° SW Daraina, -13.26333, 49.60333, 650–800 m, rainforest (B.L. Fisher et al.) (CAS). **Toamasina**: RNI Betampona, 34.08 km 332° Toamasina, -17.91977, 49.20039, 525 m, rainforest (B.L. Fisher et al.) (CAS).

##### Diagnosis.

With head in full-face view, lateral cephalic margins converging posteriorly towards eye level and covered with erect hairs; erect hairs lacking posterior to eye level; anteromedian margin of clypeus broadly convex; two apical teeth of mandible normally spaced.

##### Description.

**Minor worker.** In full-face view, head sides anterior to level of eye parallel; lateral cephalic margins posterior to level of eye converging progressively to posterior margin; eye large (EL/CS: 0.28±0.01; 0.27–0.29), protruding, and breaking lateral cephalic border, level of posterior margin located at posterior 1/3 of head (PoOc/CL: 0.31±0.02; 0.30–0.33); frontal carinae slightly diverging posteriorly (FR/CS: 0.25±0.01; 0.24–0.26), larger than smallest distance to eye; clypeus with rounded anterolateral corner and broadly angulate anteromedian margin; mandible with two apical teeth distantly spaced; antennal scape relatively long (SL/CS: 1.95±0.00; 1.95–1.96). Mesosoma low and long (MPH/ML: 0.30); dorsum of mesosoma more or less flat, promesonotum weakly convex, posterior portion of mesonotum flat immediately anterior to barely visible metanotal groove; propodeal dorsum anteriorly slightly convex and posteriorly flat, its junction with declivity bluntly angulate; propodeal dorsum 3 × as long as declivity. Petiole nodiform, with dorsal margin inclined posteriorly and forming a blunt angle to anterior margin; anterior face 1/2 height of posterior face, which inclines anteriorly to the dorsal face; femur of hind leg rounded axially, not twisted basally.

First and second gastral tergites without a pair of white spots; lateral margin of head anterior to level of eyes covered with erect hairs, which are lacking posterior to level of eyes; two erect hairs present near posterior cephalic margin; antennal scape covered with suberect hairs inclined at ca. 30° as well as appressed hairs; pronotum with a pair of erect hairs; posterodorsal angle of propodeum with a pair of erect hairs.

**Major worker.** Unknown.

##### Distribution and biology.

**Figure 76.**
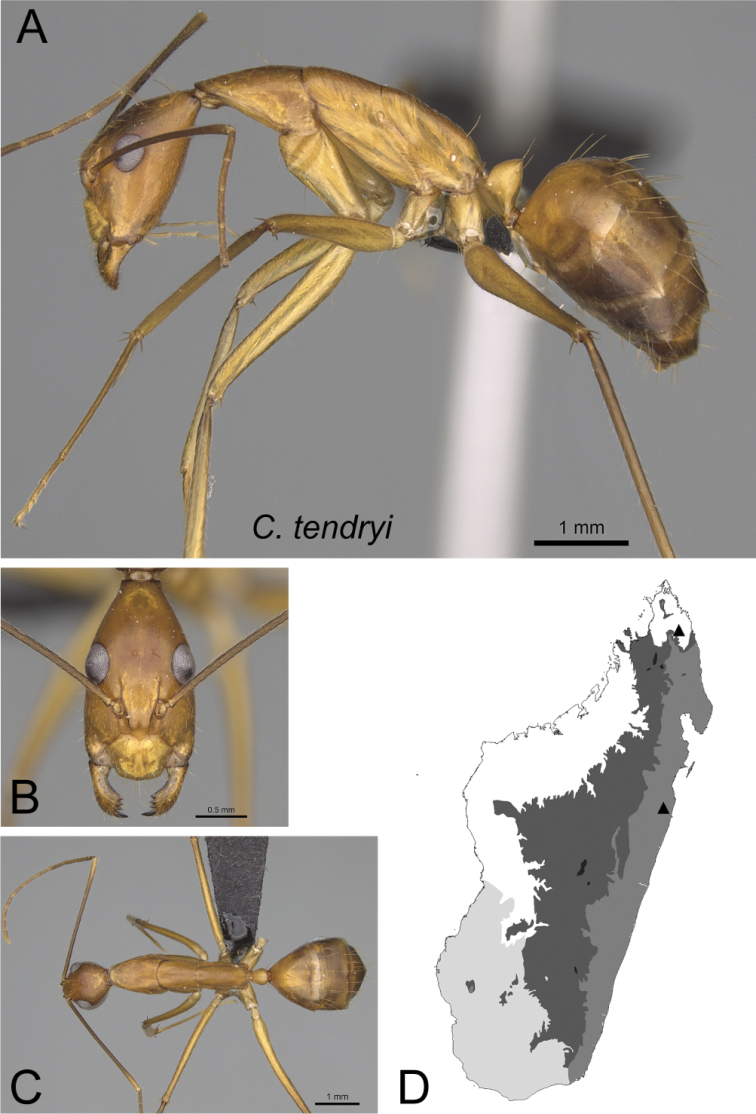
*Camponotustendryi***A** lateral view **B** head in full-face view **C** dorsal view of holotype minor worker CASENT0145284**D** distribution map.

The distribution of *C.tendryi* is limited to Madagascar, in rainforest habitats of the RNI Betampona and RS Ambatovaky in the east, and the transitional humid forest of Binara in the northeast (Fig. 76D). Specimens have been collected foraging on lower vegetation, but the species nests in rotten logs.

##### Discussion.

See discussion under *C.dufouri*.

*Camponotustendryi* were not included in the morphometric analysis due to the lack of sufficient minor worker samples.

##### Etymology.

The species name *tendryi* is a Latin noun in the genitive case derived from the first name of a male person “Tendry”.

#### ﻿ Camponotus vano sp. nov.

Taxon classificationAnimaliaHymenopteraFormicidae

C8C9E39E-B37D-5272-8007-2BCDCD26CEC8

http://zoobank.org/E3794FA9-9ED3-4AD3-811A-744DC7630861

Figs 19B , 77

##### Holotype worker.

**Madagascar**: Province **Toamasina**: PN Zahamena, Onibe River, -17.75908, 48.85468, 780 m, rainforest, beating low vegetation, 22 Feb 2009 (B.L. Fisher, Malagasy Arthropod Team) collection code: BLF22341, specimen code: CASENT0217316 (CAS).

##### Paratypes.

2 minor workers and 1 major worker with same data as holotype but respectively specimen coded as: CASENT0077651, CASENT0840785, CASENT0152085 (PBZT, CAS).

##### Additional material examined.

**Madagascar: Toamasina**: PN Zahamena, Onibe River, -17.75908, 48.85468, 780 m, rainforest (B.L. Fisher, Malagasy Arthropod Team) (CAS); Corridor Forestier Analamay-Mantadia, Ambatoharanana, -18.80388, 48.40506, 1013 m, rainforest (B.L. Fisher et al.) (CAS).

##### Diagnosis.

Lateral cephalic margins approximately parallel in full-face view; two apical teeth of mandibles closely spaced; lateral cephalic margin posterior to eye level without erect hairs; antennal scape covered with appressed hairs; mesosoma very low and long; propodeal dorsum at least four times as long as declivity; petiole nodelike.

##### Description.

**Minor worker.** With head in full-face view, lateral margins anterior to eye level parallel, posteriorly rounding evenly towards a rear margin that is somewhat necked; eye protruding and large (EL/CL: 0.29), breaking lateral cephalic margin, location of its posterior margin at posterior 1/3 of head (PoOc/CL: 0.34); frontal carinae widely opened (FR/CS: 0.29±0.00; 0.29–0.30), posteriorly parallel; clypeus with blunt or poorly defined anterolateral angle, anteromedian margin broadly convex; two apical teeth of mandible closely spaced; antennal scape relatively long (SL/CS: 1.68±0.03; 1.66–1.70). Mesosoma low and long (MPH/ML: 0.29±0.02; 0.28–0.31), promesonotum slightly convex, mesonotum and propodeum more or less flat; metanotal groove feebly visible, propodeal dorsum rounding to declivity, propodeal dorsum four times as long as declivity. Petiole nodelike, with dorsal margin inclined posteriorly and rounding to anterior margin; tibia of hind leg rounded axially, without basal twist.

First and second gastral tergites without a pair of white spots; erect hairs on lateral margin of head present anterior to eye level and absent behind eye level; posterior margin of head with two erect hairs; antennal scape covered with appressed hairs; junction of propodeal dorsum and declivity with a pair of erect hairs.

**Major worker.** Differing from minor worker in the following characters: enlarged, elongate, and rectangular head (CS: 1.67; CWb/CL: 0.64) with slightly concave posterior margin; two apical teeth of mandible normally spaced; distal 1/3 of antennal scape surpassing posterior cephalic margin; robust, short, and high mesosoma, metanotum distinctly visible, propodeal dorsum almost 3 × as long as more vertical declivity, their junction forming a blunt angle; with more pairs of erect hairs. Head dark brown, body color pale brown, propodeum much darker.

##### Distribution and biology.

**Figure 77.**
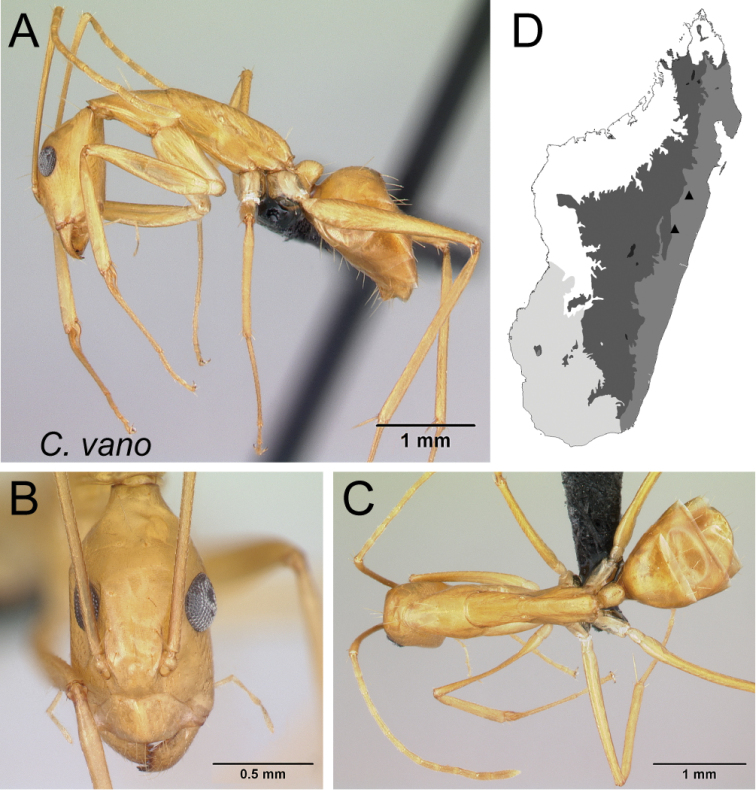
*Camponotusvano***A** lateral view **B** head in full-face view **C** dorsal view of holotype minor worker CASENT0217316**D** distribution map.

A species endemic to Madagascar, *C.vano* is geographically restricted to the PN Zahamena and the Corridor Forestier Analamay-Mantadia in humid forests in the east of the island (Fig. 77D). Collection data indicate that the species is arboreal; nests have been found in dead bamboo on the ground, while workers have been found foraging on lower vegetation.

##### Discussion.

See discussion under *C.radamae*.

*Camponotusvano* was not included in the NC-clustering analysis due to an inadequate number of minor workers. However, its qualitative morphological traits are sufficient to supports its status as a real species.

##### Etymology.

The species name *vano* is a non-Latin singular noun used in apposition and refers to the Malagasy name for “heron”. It refers to the elongate body form of the species.

## Supplementary Material

XML Treatment for Camponotus aina

XML Treatment for Camponotus aro

XML Treatment for Camponotus asara

XML Treatment for Camponotus atimo

XML Treatment for Camponotus aurosus

XML Treatment for Camponotus becki

XML Treatment for Camponotus bemaheva

XML Treatment for Camponotus boivini

XML Treatment for Camponotus bozaka

XML Treatment for Camponotus cemeryi

XML Treatment for Camponotus cervicalis

XML Treatment for Camponotus daraina

XML Treatment for Camponotus dufouri

XML Treatment for Camponotus gibber

XML Treatment for Camponotus gouldi

XML Treatment for Camponotus hagensii

XML Treatment for Camponotus harenarum

XML Treatment for Camponotus hova

XML Treatment for Camponotus hovahovoides

XML Treatment for Camponotus imitator

XML Treatment for Camponotus immaculatus

XML Treatment for Camponotus joany

XML Treatment for Camponotus karsti

XML Treatment for Camponotus kelimaso

XML Treatment for Camponotus liandia

XML Treatment for Camponotus lokobe

XML Treatment for Camponotus lubbocki

XML Treatment for Camponotus mahafaly

XML Treatment for Camponotus mixtellus

XML Treatment for Camponotus niavo

XML Treatment for Camponotus quadrimaculatus

XML Treatment for Camponotus radamae

XML Treatment for Camponotus roeseli

XML Treatment for Camponotus rotrae

XML Treatment for Camponotus sambiranoensis

XML Treatment for Camponotus strangulatus

XML Treatment for Camponotus tapia

XML Treatment for Camponotus tendryi

XML Treatment for Camponotus vano

## Supplementary materials

Supplementary material 1 Table S1 Data type Morphological measurements Explanation note Table S1. Basic measurements of individual specimens arranged by species code, collection code, and specimen code (unique identification number). See text for abbreviations. https://binary.pensoft.net/file/678137This dataset is made available under the Open Database License (http://opendatacommons.org/licenses/odbl/1.0/). The Open Database License (ODbL) is a license agreement intended to allow users to freely share, modify, and use this Dataset while maintaining this same freedom for others, provided that the original source and author(s) are credited.Jean Claude Rakotonirina, Brian L. Fisher
